# NMR of nuclei bound to a paramagnetic metal centre: the case of *S* = 1 square-planar ruthenium(ii)†

**DOI:** 10.1039/d6sc04475a

**Published:** 2026-08-19

**Authors:** Leon K. Paschai Darian, Charlotte S. V. Weiß, Kim Warth, Tim Bruckhoff, Joachim Ballmann, Markus Enders, Lutz H. Gade

**Affiliations:** a Anorganisch-Chemisches Institut, Universität Heidelberg Im Neuenheimer Feld 270 69120 Heidelberg Germany lutz.gade@uni-heidelberg.de

## Abstract

A series of ruthenium(ii) halide complexes, with a carbazole based PNP pincer ligand, featuring an intermediate-spin (*S* = 1) ground state, seldomly adopted by 4d metals such as ruthenium, have been synthesized. This has been achieved using the planar tridentate ligand, which combines hard and soft donor atoms, giving rise to a very large zero-field splitting (*D* > 200 cm^−1^). The electronic structure and systematic influence of the fourth (halogenido) ligand have been explored theoretically *via* DFT and CASSCF calculations as well as experimentally *via* UV-vis spectroscopy and SQUID magnetometry. Analysis of the strikingly well-resolved pNMR spectra allowed the detection of the directly bound ^31^P nuclei at around −3900 ppm for all discussed complexes, as well as the ^19^F resonance of the fluorido complex at over +3900 ppm chemical shift, which are a direct probe for the sign and magnitude of the hyperfine coupling constants *A*_iso_. Simulation of the room temperature spectra based on DFT and CASSCF calculations allowed for full assignment of the observed resonances and experimental determination of the axial zero-field splitting parameter *D* from VT-NMR experiments.

## Abstract 2

For a series of ruthenium(ii) halide complexes pNMR spectra allowed the detection of the directly bound ^31^P nuclei at −3900 ppm, as well as the ^19^F resonance at over +3900 ppm, which are a direct probe for the sign and magnitude of *A*_iso_.

## Graphical abstract

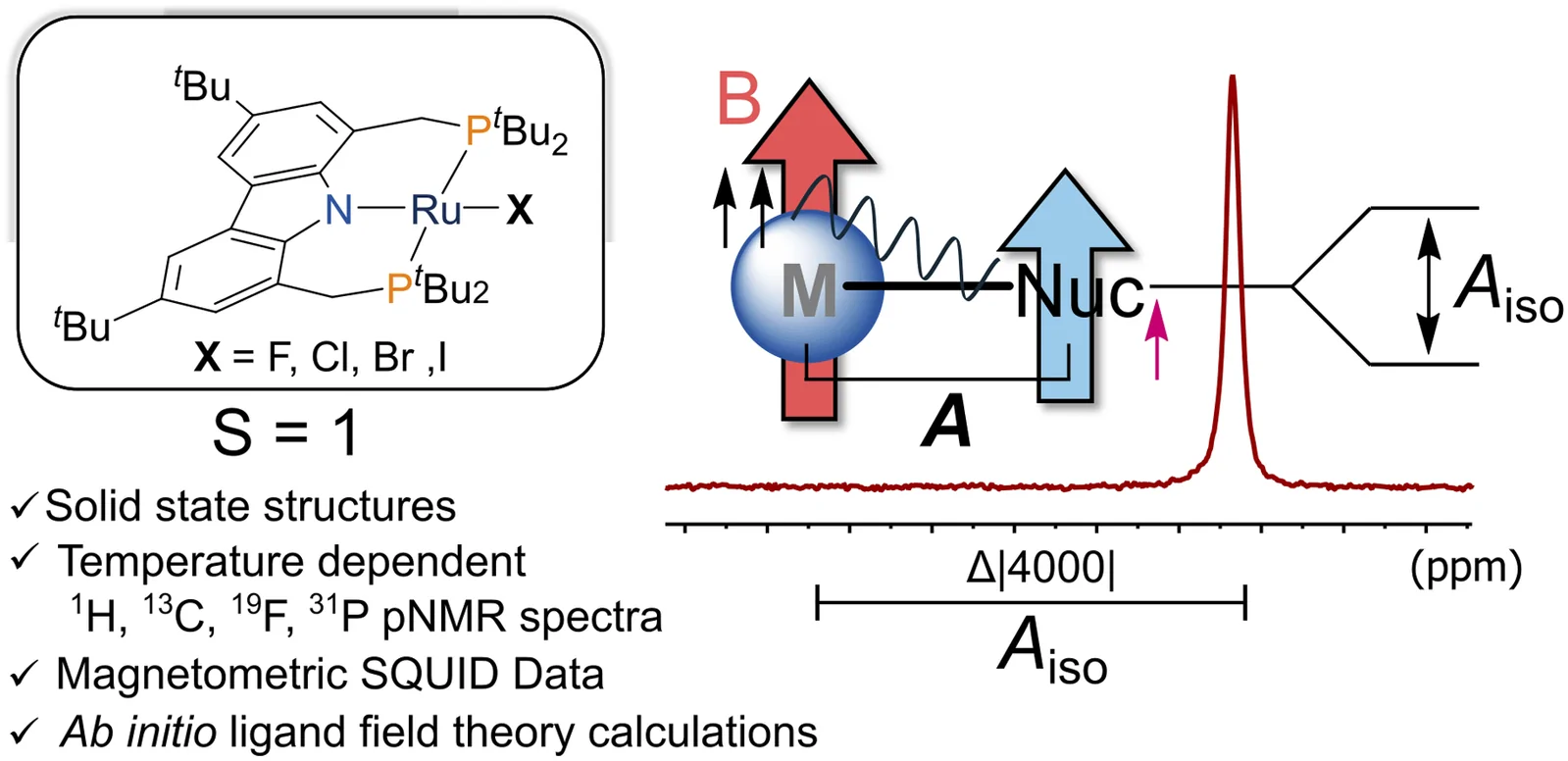

## Introduction

Paramagnetic NMR (pNMR) spectroscopy was first developed in the late 1950s^1^ but was underexploited for a long time as an analytical technique due to the challenges posed by the interpretation and analysis of the spectra.^2^ With the advent of powerful theoretical modelling tools,^3^ combined with advances in spectrometer hardware, there has been a revival of the method in more recent times.^4^ Nowadays, its applications range from drug development^5^ to the analysis of single molecule magnets^6^ and paramagnetic transition metal catalysts and even catalysis intermediates.^7^ This is accompanied by ever refined computational and theoretical prediction methods, which continue to be developed and provide the foundation for further developments in the field.^8^

For solutions of open-shell transition metal compounds adopting an *S* = 1 ground state, pNMR spectra with sharp resonances and, in some cases, resolved spin–spin couplings are occasionally observed. Since the late 1970s, pNMR spectra of intermediate-spin Fe(ii) porphyrins have been used to elucidate the electronic structure of the compounds.^9^ Of particular note is the work by Collmann and Ibers *et al.* (Fig. 1A)^10^ on a porphyrin Ru(ii) dimer with an overall spin of *S* = 1, in which ^1^H-pNMR spectroscopy was used to elucidate the structural composition of the compound as a dimer with a Ru–Ru single bond, a result which was subsequently confirmed by single crystal X-ray diffraction.

**Fig. 1:**
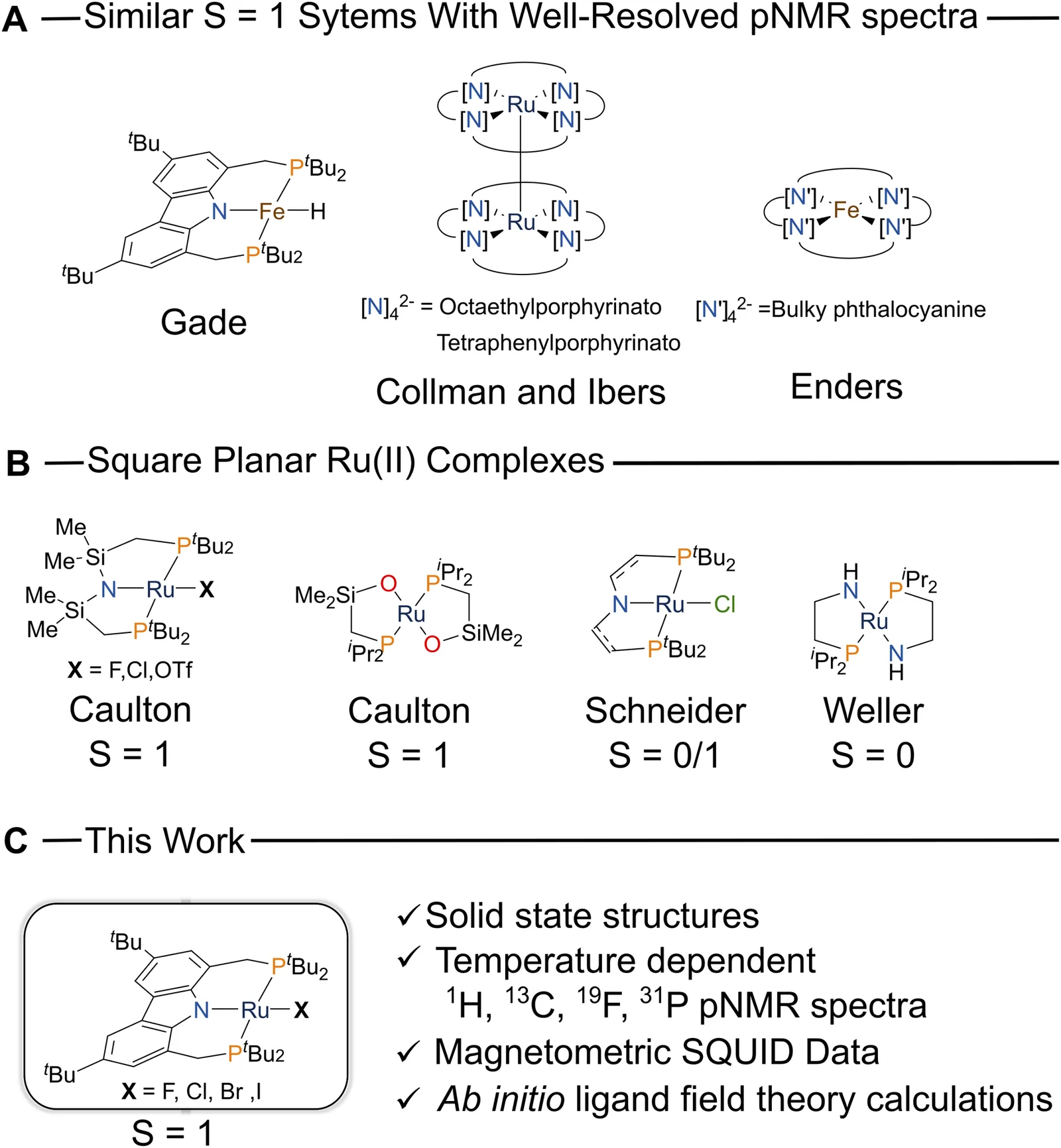
(A) Examples of *S* = 1 complexes with well resolved pNMR spectra; (B) examples in the literature of four coordinate Ru(ii) complexes; (C) the series of complexes studied in this work.

Since then, a range of pNMR studies on *S* = 1 systems have been published, including the use of residual quadrupolar couplings (RQCs) and pseudo contact shift (PCS) contributions to determine the axial zero-field splitting parameter *D*.^11^ In a study of a PNP coordinated Fe(ii) hydride reported by our group, it was even possible to observe the directly bound ^31^P and hydride ^1^H nuclei at giant chemical shifts (Fig. 1A; left).^12^

For 3d transition metals, such as iron, intermediate-spin (IS) complexes are commonly found for non-octahedral coordination geometries.^13^ On the other hand, the coordination chemistry of Ru(ii) is dominated by six-coordinate octahedral complexes as, for example, those commonly used as photoredox catalysts.^14^ Reports about four-coordinate Ru(ii) compounds remain comparatively scarce, with early examples adopting a sawhorse/*cis*-divacant-octahedral geometry. In combination with strong field ligands this leads to low-spin (LS) diamagnetic complexes.^15^

The first examples of square-planar Ru(ii) complexes were published by Caulton *et al.* using a PNP pincer ligand scaffold (Fig. 1B; far left side).^16^ The complexes were found to adopt an intermediate-spin triplet ground state, which is also observed in a related homoleptic bis-P,O complex (Fig. 1B; half left side).^17^ Subsequent work by Schneider *et al.* (Fig. 1B; half right side) on a similar PNP ligand system revealed that the spin state of the system is dependent on the degree of π-bonding of the amide donor. Small changes in the ligand backbone were thus found to induce a change in the ground-state spin multiplicity.^18^ In ideal *C*_2v_ symmetry, with the *x*-axis along the Ru–N bond, the *y*-axis along the P–Ru–P bond and the *z*-axis orthogonal to the xy plane, the d orbital ordering of d_*yz*_, d_*xy*_/d_*z*^2^_, d_*xz*_, d_*x*^2^−*y*^2^_ is obtained. In either case (LS or IS) the highest energy d_*x*^2^−*y*^2^_ orbital is unoccupied, but the degree of π-donation from the N-donor atom can influence the energy of the d_*xz*_ orbital, leading to an unoccupied high energy d_*xz*_ orbital and doubly occupied d_*xy*_ and d_*z*^2^_ orbitals for a stronger donor, resulting in a LS complex, or a lower energy singly occupied d_*xz*_ and one singly and one doubly occupied d_*xy*_ or d_*z*^2^_ orbital for a weaker donor, leading to an IS complex.

Recently, the family of square-planar complexes was expanded with a homoleptic bis-P,N complex with a singlet ground state, reported by Weller and coworkers, which displayed high activity in catalytic amine-borane dehydropolymerization (Fig. 1B; right side).^19^

In the present study, we report a series of Ru(ii) halide complexes, coordinated by the carbazole based PNP (^cbz^PNP) pincer ligand previously used to coordinate low-valent 3d/4d metals.^20,21^ For these new complexes intermediate-spin (*S* = 1) ground states have been identified and all have given rise to well-resolved pNMR spectra. This has not only allowed full assignment of all resonances based on a combination of theoretical modelling, 2D-NMR and residual dipolar couplings (RDCs), but also the detection of NMR active nuclei directly bonded to the paramagnetic centre at extreme chemical shifts (Fig. 2). Moreover, a detailed analysis of the temperature dependence of the pNMR resonances has allowed quantitative estimates of the zero-field parameter *D* by a method which is complementary to the conventional analytical magnetic techniques.

**Fig. 2:**
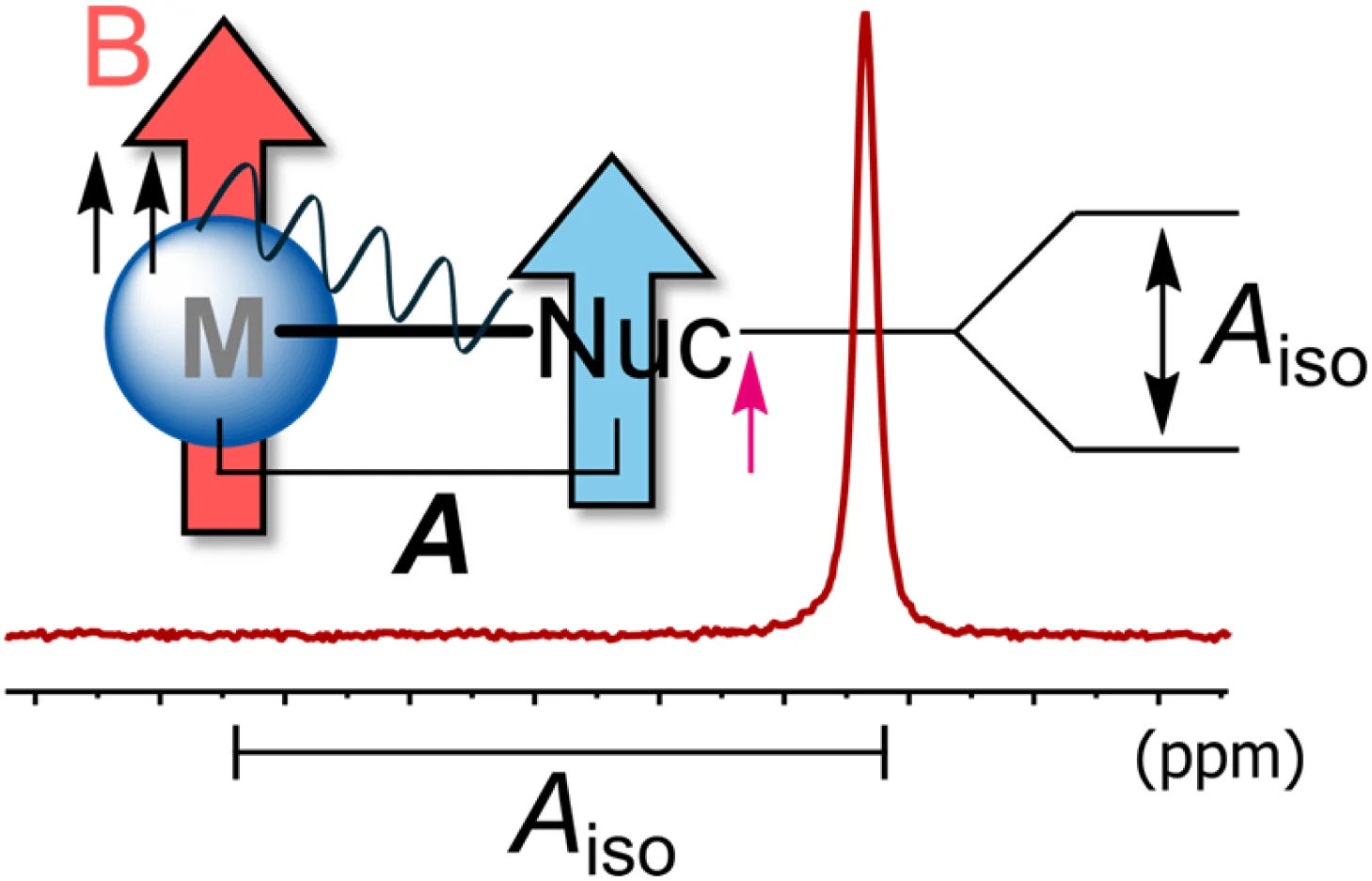
Directly bonded NMR nuclei at paramagnetic metal centres as NMR probes with extremely large chemical shift dispersion. Interaction of a metal centre with two unpaired electrons (black arrows) in a magnetic field (light red) and an NMR active nucleus (nuclear spin in pink). Fast relaxation of the unpaired electrons (curved line) leads to the energy-weighted average of the electron interacting with the nuclear spin *via* hyperfine coupling *A*. If the coupling is sufficiently large, the energy difference of the NMR energy levels becomes dominated by *A*_iso_, making the observed chemical shift a direct sign-dependent probe for *A*_iso_.

## Results and discussion

### Synthesis and molecular structures of a series of square-planar Ru(ii) complexes

Similar to previous protocols for the synthesis of square-planar metal complexes with the ^cbz^PNP ligand, the protio ligand 1 was deprotonated *in situ* with LiN(SiMe_3_)_2_ and reacted with the Ru(ii) precursor [Ru(cymene)Cl_2_]_2_ to give the Ru(ii) complex 2, which was isolated as a deep-blue solid (Scheme 1).

**Scheme 1:**
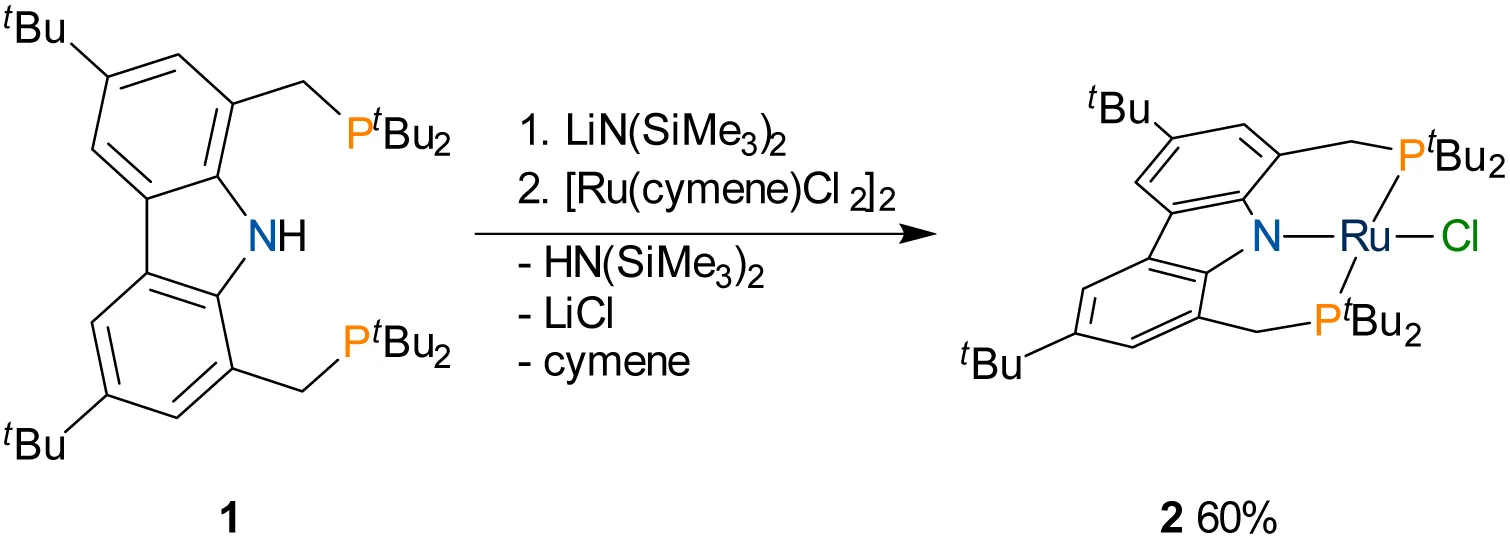
Synthesis of the ruthenium(ii) chlorido pincer complexes 2.

The chlorido complex 2 provided the starting point for the preparation of the other halide complexes 3–5 in one or two steps. The bromido and iodido complexes 4 and 5 were synthesised by reaction with TMS-Br and TMS-I in toluene or benzene, respectively, and isolated in good yields (83% for 4 and 54% for 5, see Scheme 2). For the fluorido complex, direct synthesis starting from 2 with metal fluoride salts, such as CsF, proved unsuccessful, while reaction with TlF at elevated temperatures, even with a large excess of the thallium reagent, gave only incomplete conversion to 3 and was accompanied by non-selective decomposition of the target complex during synthesis. The preparation of the fluorido complex 3 was achieved by reaction of the iodido complex 5 with PPh_4_F (60% see Scheme 2).

**Scheme 2:**
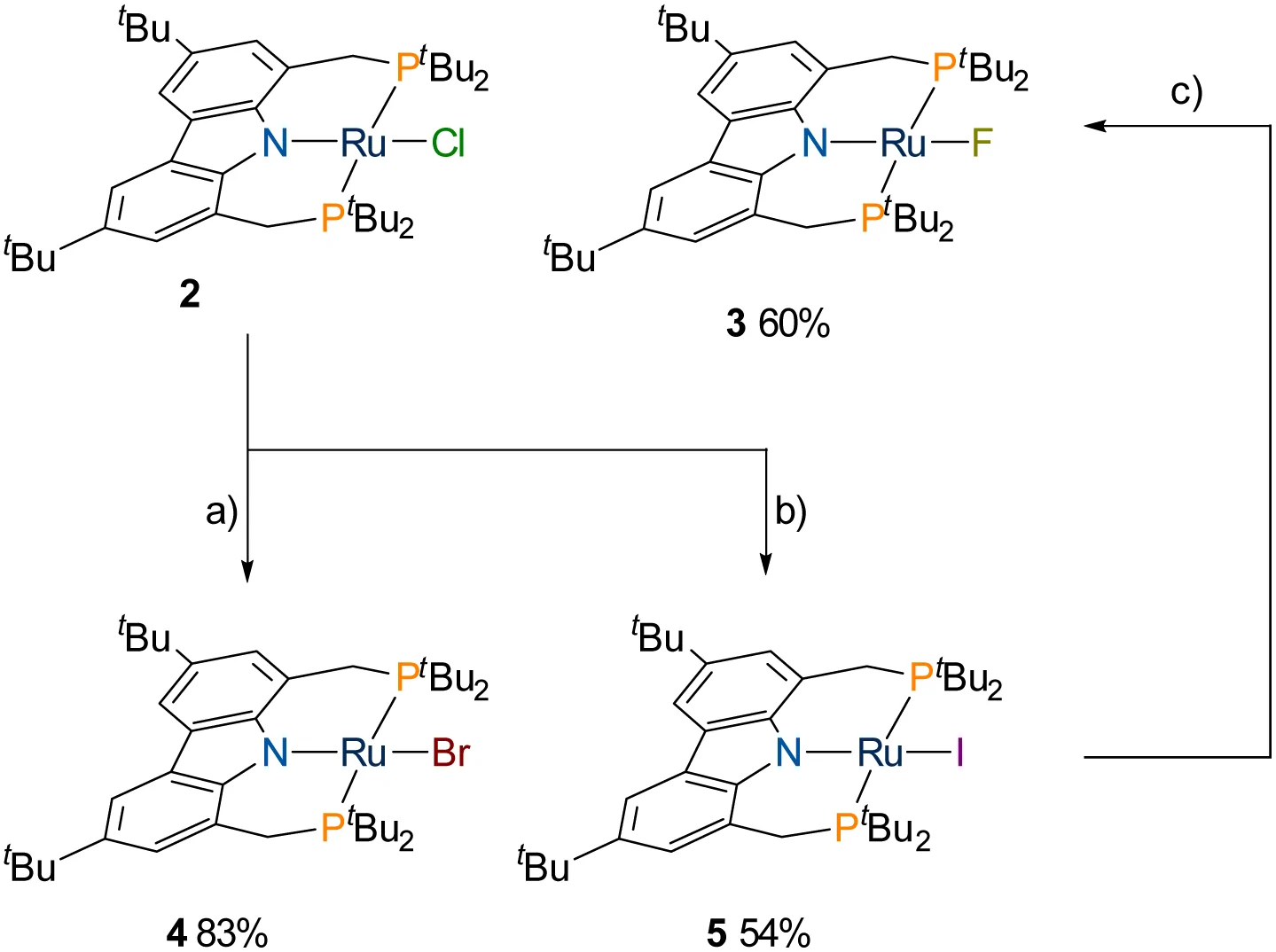
Synthesis of the ruthenium(ii) halogenido pincer complexes 3–5: (a) reaction of 2 with TMS-Br in toluene to afford 4; (b) reaction of 2 with TMS-I in toluene to afford 5; (c) reaction of 5 with PPh_4_F in benzene to afford 3.

Although 3 proved to be thermally unstable in solution over time, complete conversion within a few minutes and rapid workup made it possible to isolate the target complex. Single crystals suitable for X-ray diffraction were grown for complexes 2–5 from saturated pentane solutions at −40 °C (Fig. 3). All the halide complexes adopt a slightly distorted square-planar geometry (2*τ*_4_ = 0.15; 3*τ*_4_ = 0.18; 4*τ*_4_ = 0.24; 5*τ*_4_ = 0.29),^22^ a distortion which increases for heavier halides. The fluorido complex 3 appears to be a notable exception, slightly less planar compared to the chlorido complex 2, although examination of the residual electron density confirms the presence of a superposition of two different molecules as minor components, exhibiting C–H bond activation of one Me group on the left and right P-^*t*^Bu group respectively, leading to bond insertion *via* cyclometallation in accordance with the decomposition pathway suggested below (*vide infra*), which precludes a more detailed discussion of that effect. The Ru–X bond lengths increase with the atomic number of the halide [Ru–F: 2.088(4) Å, Ru–Cl 2.3532(7) Å, Ru–Br 2.4856(7) Å, and Ru–I 2.6700(4) Å] and are comparable to the range of values reported in the literature.^16*b*,23^ In particular, the Ru–Cl bond is slightly shorter compared to the intermediate-spin complexes by Caulton *et al.* (2.361(1) Å)^16*a*^ and Schneider *et al.* (2.3720(5) Å).^18*b*^ The Ru–N bond is contracted upon going from the fluorido to the bromido complex [F: Ru–N 2.078(3) Å, Cl: Ru–N 2.0728(19) Å, and Br: Ru–N 2.037(4) Å], reflecting reduced competition between the *trans*-disposed π-donors. This trend is discontinued for the iodido complex which features a slightly longer Ru–N bond compared to 4 (Ru–N 2.057(4) Å), possibly resulting from increased inter-ligand steric repulsion.

**Fig. 3:**
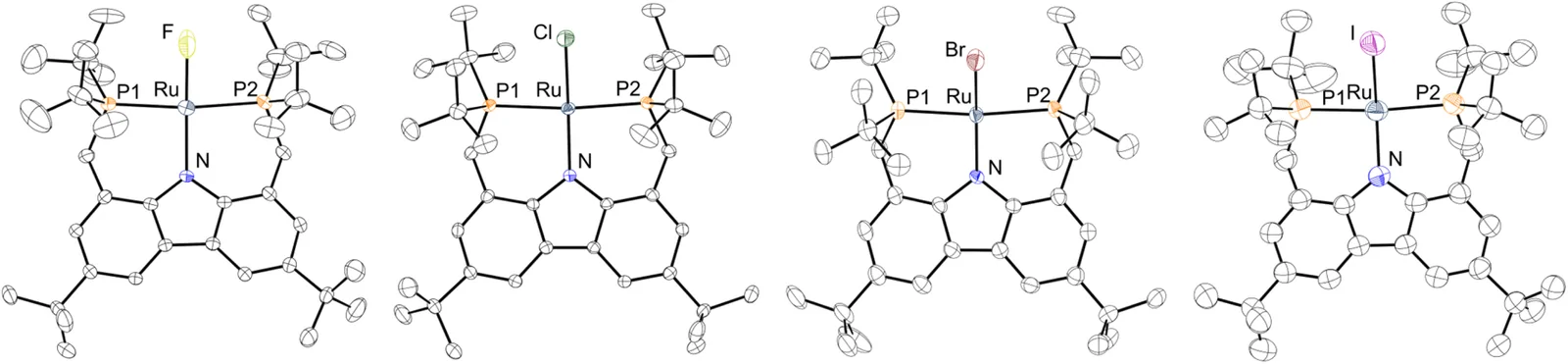
Molecular structures of 2–5 (thermal ellipsoids are shown at 50% probability, and hydrogen atoms are omitted; in 3 and 2, disordered *t*-butyl groups on the P atoms are omitted for clarity). Selected bond lengths (Å) and angles (deg) for 3: Ru–P1 2.3544(10), Ru–P2 2.3573(11), Ru–F 2.088(4), Ru–N 2.078(3), and P1–Ru–P2 170.98(4) N–Ru–F 163.73(17); for 2: Ru–Cl 2.3532(7), Ru–P1 2.3793(6), Ru–P2 2.3789(6), Ru–N 2.0728(19), P2–Ru–P1 173.02(2), and N–Ru–Cl 166.25(6); for 4: Ru–Br 2.4856(7), Ru–P1 2.3723(12), Ru–P2 2.3737(12), Ru–N 2.037(4), P1–Ru–P2 171.36(5), and N–Ru–Br 155.16(13); for 5: Ru–I 2.6700(4), Ru–P1 2.3781(12), Ru–P2 2.3878(12), Ru–N 2.057(4), P1–Ru–P2 166.55(5), and N–Ru–I 152.16(11).

### The electronic structures of complexes 2–5: photophysics and magnetism

To gain insights into the electronic structure of the square-planar complexes, their spectroscopic and magnetic properties were investigated by a combination of experimental and theoretical methods. Measurements of the effective magnetic moments in solution *via* Evans' method^24^ led to the assignment as intermediate-spin complexes: *µ*_eff_: 2.37 *µ*_B_ (Ru–F, 3); 2.76 ± 0.16 *µ*_B_ (Ru–Cl, 2); 2.75 ± 0.09 *µ*_B_ (Ru–Br, 4); 2.91 ± 0.22 *µ*_B_ (Ru–I, 5). These values are close to the expected spin-only value for an *S* = 1 system of 2.83 *µ*_B_ with the exception of the fluorido complex 3. While the lower *µ*_eff_ of the latter compared to the other complexes fits the general trend of heavier halogens giving rise to higher *µ*_eff_ values, we additionally attribute the drastic deviation to a small degree of decomposition in solution.

In solution, compound 3 is stable over the course of several hours, which allowed for its characterisation by NMR spectroscopy, because signals of 3 can be identified even in the presence of decomposition products. However, in SQUID magnetometry the bulk properties are measured, so that impurities hinder a reliable data analysis. For UV-vis spectroscopy, high dilution is necessary which led to rapid decomposition of the complex, presumably due to residual N_2_, which was shown to catalyse the decomposition of a similar Ru-OTf complex.^16*c*^ We note that these data are also in accordance with reported *µ*_eff_ values of the chlorido complexes reported by Caulton and Schneider.^16*a*,18*b*^ A computational study based on DFT modeling of all four complexes has also established a more stable IS (*S* = 1) ground state, compared to the LS (*S* = 0) configuration (SMD(benzene); PWPB95-D3BJ/SARC-ZORA-Def2-TZVP(Ru/I)/ZORA-Def2-TZVP(others)//r^2^scan-3c/Def2-mSVP level of theory): Δ(*E*^T^ − *E*^S^)[kcal mol^−1^] = −7.00(Ru–F, 3); −7.62(Ru–Cl, 2); −7.68(Ru–Br, 4); −8.05(Ru–I, 5) (see the SI). UV-vis spectra of the Ru–Cl, Ru–Br and Ru–I complexes 2, 4 and 5 are displayed in Fig. 4 along with the respective computationally modeled spectra for the intermediate-spin (*S* = 1) ground state.

**Fig. 4:**
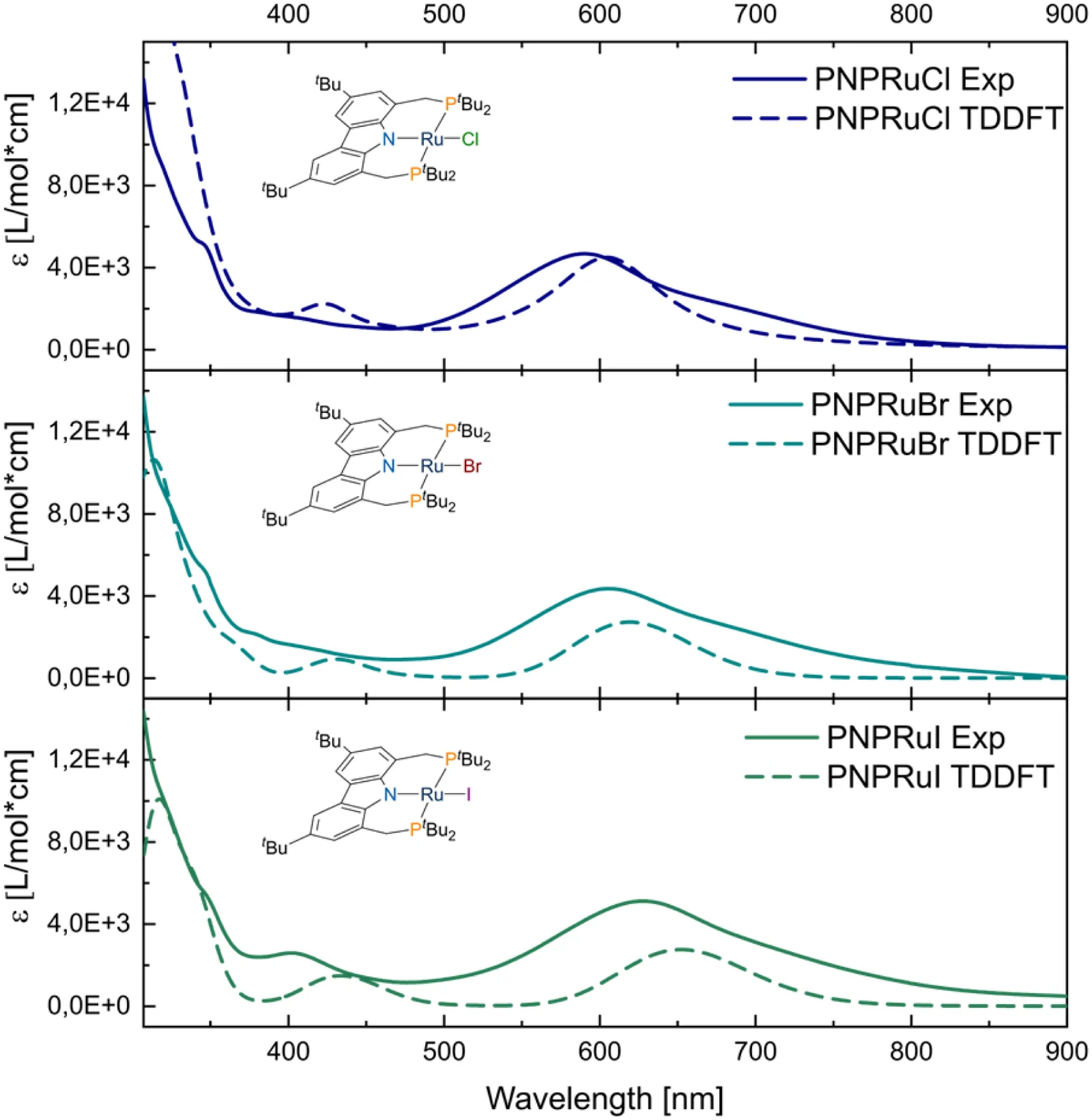
Comparison of the TD-DFT (dotted lines) (*S* = 1 SMD(pentane); TPSSh-D3BJ/ZORA-Def2-TZVP//r^2^scan-3c/Def2-mSVP level of theory) and experimental (solid lines) UV-vis spectra in pentane of complexes 2, 4, and 5.

As shown, the computed spectra modeled using time dependent (TD)-DFT reproduce the general shape as well as the observed trends for all halide complexes (for Ru–F 3, see SI, Fig. S25). All spectra display strong absorption below 300 nm, attributed to excitations within the ligand framework, as well as a weaker absorption band at around 500–650 nm. This band is attributed to an excitation with LMCT character from the carbazole backbone based π-system into the metal d_*xz*_ orbital, as reflected in the natural transition orbitals displayed in Fig. 5 (see SI, Table S11).

**Fig. 5:**
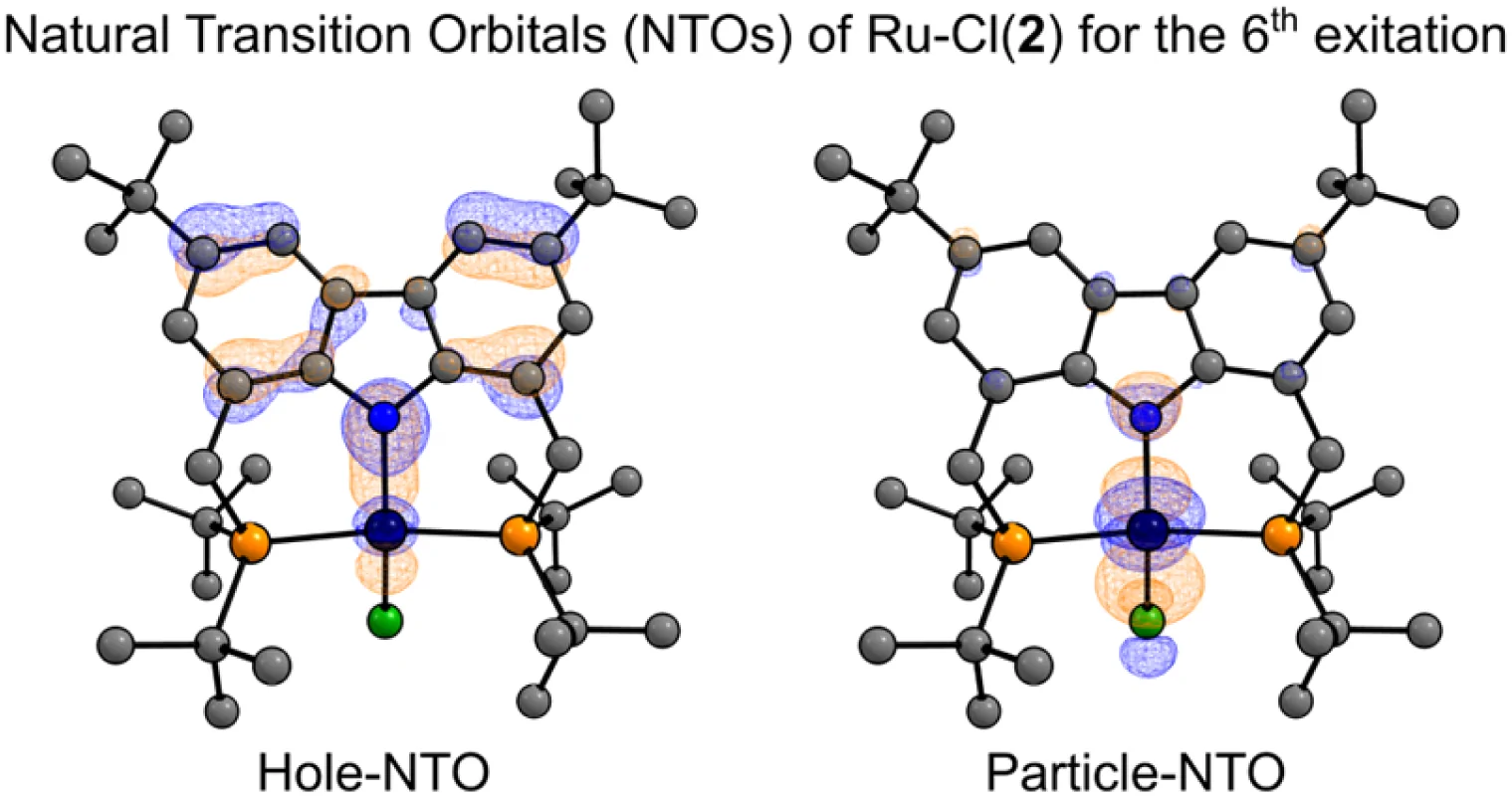
NTOs of the 6th excitation, responsible for absorption in the VIS region of Ru–Cl(2) calculated at the TPSSh-D3BJ/SARC-ZORA-Def2-TZVP(Ru)/ZORA-Def2-TZVP(rest)//r^2^scan-3c/Def2-mSVP level of theory.

A red shift of this band along the series of complexes is observed (Table 1), suggesting a lowering of the respective d_*xz*_ orbital energy level along the series (*vide infra*). As the fluorido complex 3 is not stable in solution (*vide supra*) and underwent a significant amount of decomposition (see SI, Fig. S25), its properties are not discussed here. However, the major band in the visible region is still recognisable at 550 nm and matches nearly exactly the TD-DFT predicted band (553 nm) in the visible region for an intermediate-spin (*S* = 1) ground state.

**Table 1:** Comparison of the calculated (*S* = 1; SMD; (pentane) TPSSh-D3BJ/SARC-ZORA-Def2-TZVP(Ru/I)//r^2^scan-3c/Def2-mSVP level of theory) and experimental absorption bands in the visible region of complexes 2–5

[nm]	Ru–F (3)	Ru–Cl (2)	Ru–Br (4)	Ru–I (5)
*λ*_max_ (exp)	550	589	605	628
*λ*_max_ (calc)	553	608	624	657

To obtain further details of the magnetic characteristics and electronic structure of complexes 2, 4 and 5 and to compare them with those of related compounds, SQUID magnetometric data were collected (see SI, Section 4) and accompanied by theoretical modeling based on complete active space self-consistent field CASSCF (6,5) calculations (see SI, Section 6.2). For powder samples of the Ru–Cl, Ru–Br and Ru–I complexes 2, 4 and 5, the product of the molar susceptibility and temperature obtained from SQUID data was simulated (see the SI for more information) and the parameters, listed in Table 2, were obtained. Generally, the complexes display very similar values for the zero-field splitting parameters *D* and |*E*/*D*|, with an overall upward trend of *D* and *E* with increasing atomic number of the halogen.

**Table 2:** Values for *g*_iso_, and zero-field splitting parameters *D* (cm^−1^) and |*D*/*E*| obtained experimentally from SQUID magnetometric data

(Exp)	Ru–Cl (2)	Ru–Br (4)	Ru–I (5)
*g*_iso_	1.94	1.87	1.91
*D* [cm^−1^]	229	238	257
|*E*/*D*|	0.31	0.33	0.33

The general results are in accordance with previously reported data for similar systems.^16*a*,18*b*^ A large positive axial zero-field splitting (*D*) combined with a high rhombicity (|*E*/*D*| ≈ 1/3) leads to an |*S* = 1, *M*_*S*_ = 0〉 ground state below the |*S* = 1, *M*_*S*_ = ±1〉 states, at a non-zero magnetic field, and leads to a significant decrease in *χT* at low temperatures.^18*b*^ The *g* values obtained specifically from *χT* fittings tend to be associated with significant errors and vary by only a small degree for all three complexes.

Essentially identical *g*_iso_ values are also obtained from CASSCF(6,5)/NEVPT2 calculations for complexes 2–5 (Table 3), while being generally somewhat higher compared to the experimentally obtained values displaying a small positive Δ*g* shift in contrast to *g* values obtained from *χT* values. This however is most likely a result of the significant errors associated with the experimentally obtained values and the overall absolute small Δ*g* shift. On the other hand, the calculated *D* and |*E*/*D*| values are very close to the experimental results.

**Table 3:** Calculated (CASSCF/NEVPT2(6,5), QDPT effective Hamiltonian) values for *g*_iso_, and zero-field splitting parameters *D* (cm^−1^) and |*E*/*D*|

(Calc.)	Ru–F (3)	Ru–Cl (2)	Ru–Br (4)	Ru–I (5)
*g*_iso_	2.22	2.22	2.23	2.23
*D* [cm^−1^]	223	217	215	214
|*E*/*D*|	0.27	0.26	0.27	0.28

The high zero-field splitting parameters, in combination with the *S* = 1 ground state, preclude the use of standard EPR spectroscopy on these complexes. For integer spin systems (such as *S* = 1) no Kramers doublet exists and EPR resonances are expected at |*D*| − |*E*|; |*D*| + |*E*| and |2*E*|, far outside the spectral range of conventional EPR spectrometers in the case of *D* values around the 200 cm^−1^ range. It should however be noted that in principle frequency-domain Fourier-transform THz-EPR spectroscopy could be used to determine zero-field splitting parameters *D* and |*E*/*D*| as demonstrated previously.^3*d*,4*h*,4*j*,6*b*,12*b*,25^

The computational study also sheds light on the highly anisotropic magnetic susceptibilities ***χ***. For the latter, the main magnetic axes *χ*_*x*_ are aligned approximately along the Ru–N bonds as shown in Fig. 6, for the Ru–Cl complex 2.

**Fig. 6:**
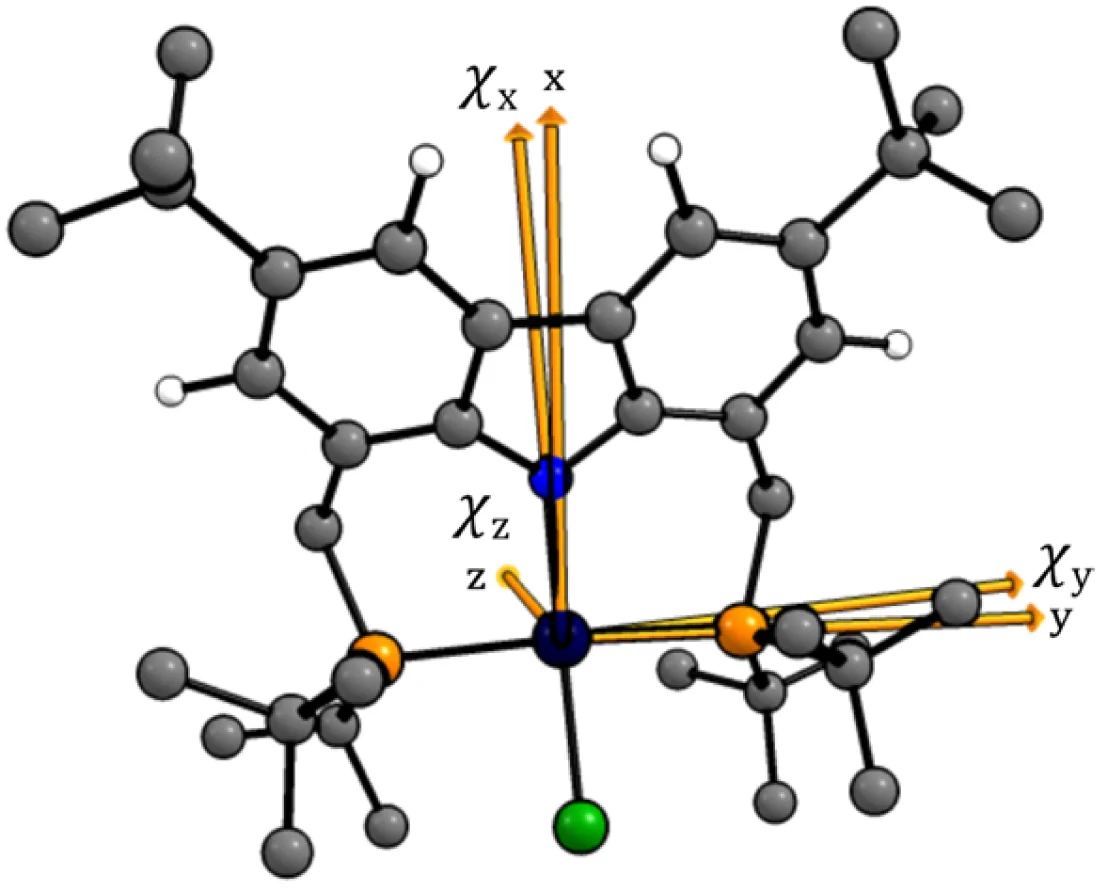
Magnetic axes *χ*_*x*_, *χ*_*y*_ and *χ*_*z*_ of 2 compared to the reference coordinate system *x*, *y* and *z*.

The ligand field splittings obtained from *ab initio* ligand field theory (AILFT) calculations are displayed in Fig. 7. As expected, the ligand field splitting decreases with heavier halogens, but all complexes feature a d_*x*^2^−*y*^2^_ level which is unoccupied and considerably higher in energy compared to the other orbitals. The d_*xz*_ orbital is 0.61–0.58 eV higher in energy compared to the nearly degenerate d_*z*^2^_ and d_*xy*_ orbitals. This results in the intermediate-spin ground state with a singly occupied d_*xz*_ and either d_*z*^2^_ or d_*xy*_ orbitals, in accordance with the ordering described in the Introduction. On a qualitative level, the ordering can be rationalized by the d_*yz*_ orbital being lowest in energy as a result of the π-acceptor interaction with the P-atoms and the d_*xz*_ orbital being destabilized by a π-donor interaction from the N- or X-atom. The d_*z*^2^_ and d_*xy*_ only have weak interaction and are therefore similar in energy to the low d_*yz*_ and high d_*xz*_ orbtials. We note that the DFT calculations suggest a singly occupied d_*z*^2^_ orbital, based on Mulliken reduced spin populations, but definitive assignment only from DFT is not possible. In general, the energy splitting of the d orbitals from AILFT is too small to allow for a definitive assignment based on this approach. The result of this electronic structure is a low degree of direct spin delocalisation to the ligand atoms as the magnetic orbitals are orthogonal to the plane spanned by the ligand atoms, and spin polarisation is only possible from π-interactions, a point which will be relevant to the pNMR studies discussed below.

**Fig. 7:**
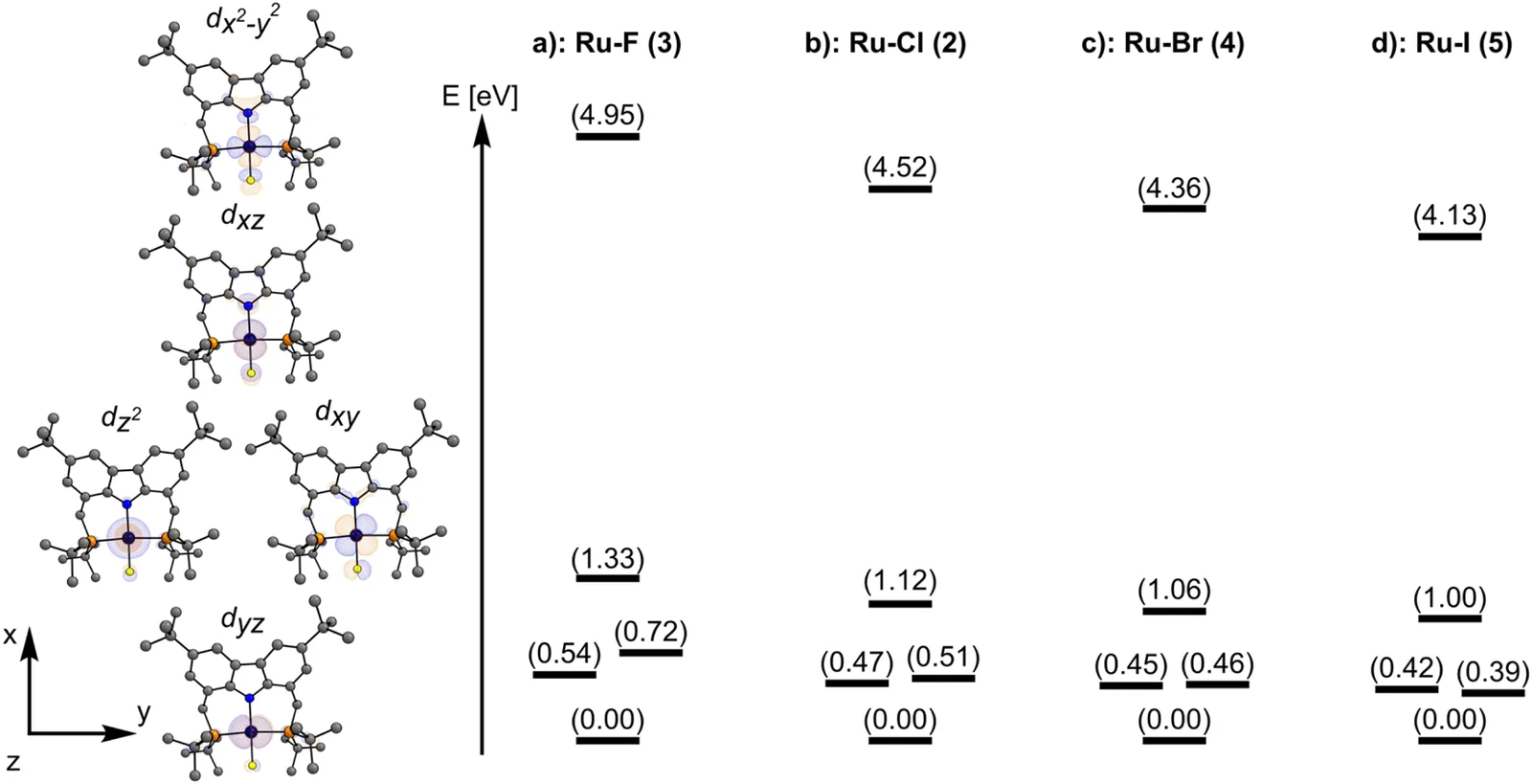
AILFT derived orbitals of the fluoride complex 3 (left side), (a–d) computed splitting of the ligand field of the PNP-pincer ligand and the halogenido ligand for the square-planar complexes 2–5 (energy difference of the orbitals relative to the lowest orbital given in eV in parentheses). All complexes feature a doubly occupied d_*yz*_ orbital, either one singly and one doubly occupied d_*z*^2^_ or d_*xy*_ orbital, a singly occupied d_*xz*_ orbital and an empty d_*x*^2^−*y*^2^_ orbital.

### Prediction and characterisation of pNMR spectra, including directly bonded heteronuclei

As mentioned above, pNMR spectroscopy is an established technique, which, in combination with theoretical modelling tools, may provide insights into the electronic structure and magnetic properties of open-shell transition metal compounds. However, in this context, the detection of nuclei close to the paramagnetic centre, as well as explicit assignment of all detected signals, remains very challenging. Before applying the method to the paramagnetic ruthenium complexes in this study, we therefore briefly recapitulate some of the underlying principles. The NMR shift (*δ*_calc_) in paramagnetic compounds in solution can be understood as a function of different contributions:^26^

1 *δ*_calc_ = *δ*_orb_ + *δ*_HF_

These include the orbital contribution (*δ*_orb_) which is also present in diamagnetic compounds and a hyperfine term (*δ*_HF_), which arises from the presence of unpaired electrons. The latter is often separated into two components, an isotropic Fermi-contact term (*δ*_FC_) and a dipolar pseudo-contact term (*δ*_PC_):

2 *δ*_HF_ = *δ*_FC_ + *δ*_PC_

The Fermi-contact (FC) contribution may be approximated using the following relation:^26^3
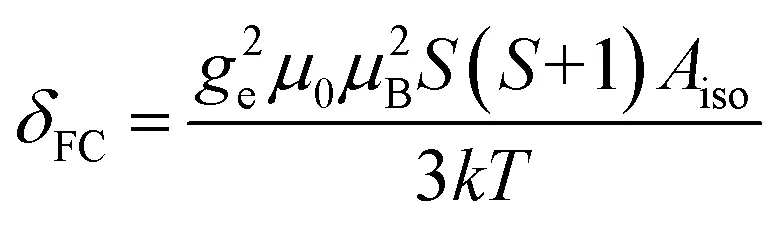
which predicts that the FC shift is proportional to the isotropic hyperfine coupling constant *A*_iso_ at a given nucleus and inversely proportional to the temperature.

The pseudo-contact contribution, on the other hand, is most often modelled *via* the point-dipole approximation:^26^4
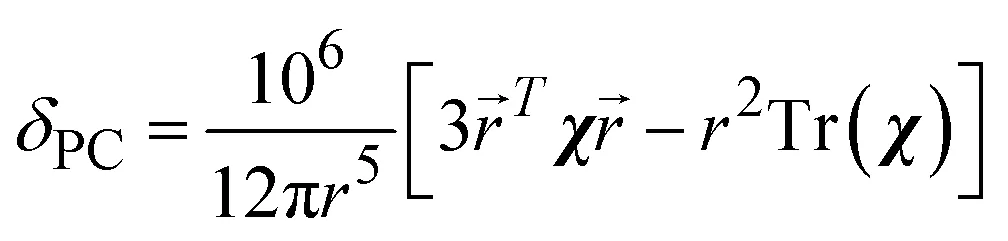
where *r* is the length of the vector between the given nucleus and the paramagnetic centre and ***χ*** is the magnetic susceptibility tensor. The values for the tensor can be obtained experimentally as *χ*_iso_, Δ*χ*_ax_ and Δ*χ*_rh_ or computationally or from an explicit expression of the magnetic susceptibility tensor. A different approach adopted by Kuprov and recently applied for pNMR of a wide range of complexes is the prediction of *δ*_HF_ based on the relation^3*c*,8*b*,12*b*,20*g*,21*d*,21*e*,27^5
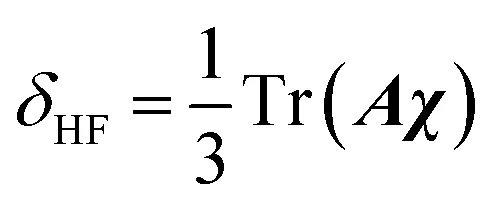
In this approach, the hyperfine tensor (***A***) and *δ*_dia_ are computed *via* DFT and the susceptibility tensor (***χ***) is iteratively optimised to minimise the root mean square deviation (RMSD) between experimental and calculated signals. For this approach, the contribution to the overall chemical shift can still be divided into a contact term, dependent on the diagonal elements of the ***A*** tensor, and a dipolar term, dependent on the off-diagonal elements of the ***A*** tensor (see SI Section 6.4).

Remarkably well-resolved ^1^H and ^13^C pNMR resonances are observed for complexes 2–5, and even resonances of directly bonded heteronuclei, such as ^31^P and ^19^F, at very large chemical shifts could be detected (*vide infra*). ^1^H and ^13^C pNMR spectra of 2–5 are displayed in Fig. 8 (red lines) and span a wide chemical shift window (*ca.* +30 to −10 ppm for ^1^H-NMR spectra and +500 to −500 ppm for ^13^C-NMR spectra). In solution at room temperature, the complexes adopt an effective *C*_2v_ symmetry based on the ^1^H and ^13^C spectra due to rapid interconversion of the carbazole backbone. For the fluorido complex 3, all expected resonances are observed and could be assigned. For the other complexes, some resonances (notably, the nuclei of the conformationally flexible CH_2_ (for 2, 4, and 5) as well as certain nuclei of the P-^*t*^Bu groups (4 and 5)) are not observed at 295 K.

**Fig. 8:**
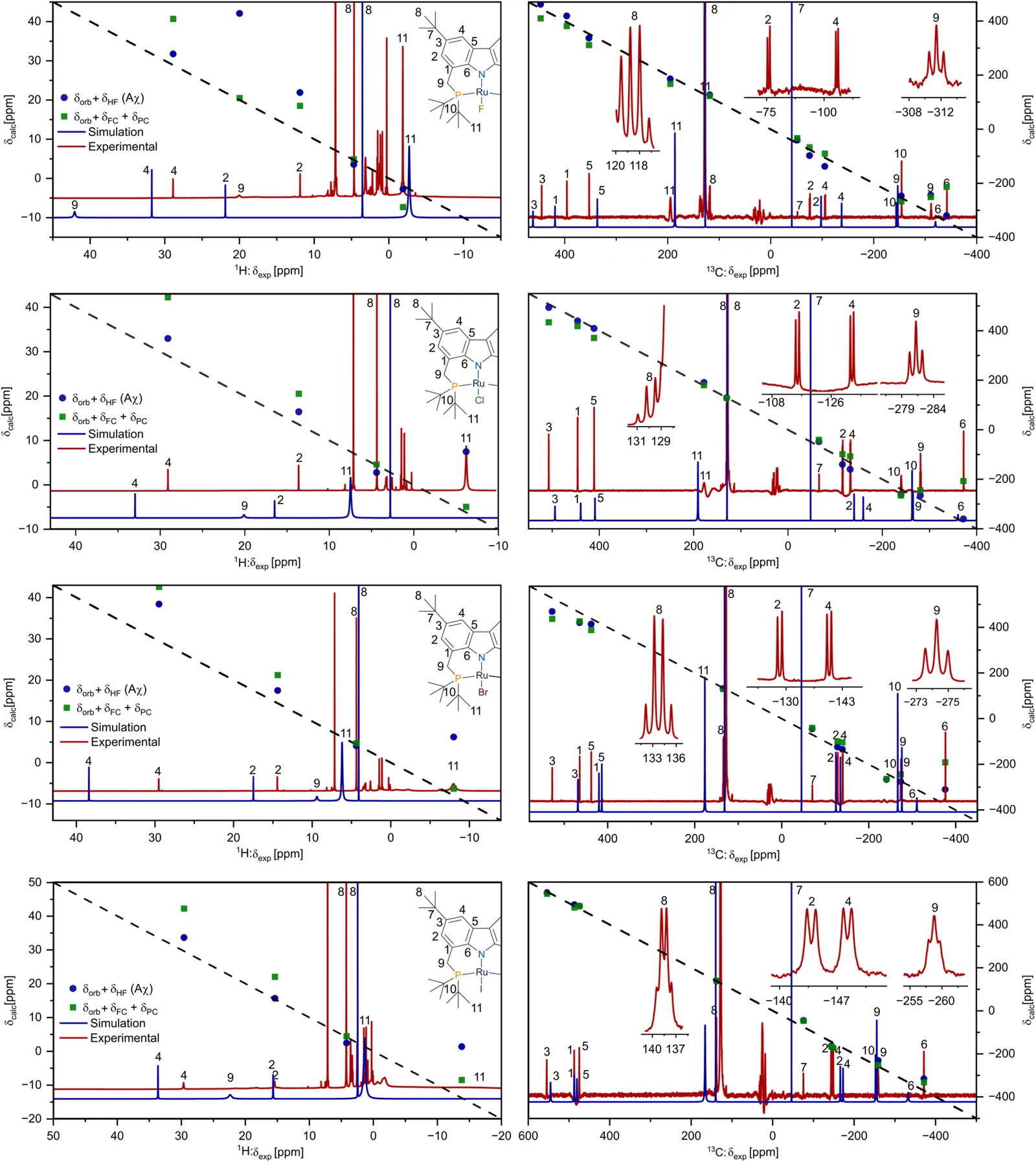
Correlation between the experimental (red line) and simulated, *via*eqn (5) (blue line), resonances of the halide complexes 2–5; ^1^H-NMR spectra (C_6_D_6_, 600 MHz, 295 K) (left side) and ^13^C-NMR spectra (C_6_D_6_, 151 MHz, 295 K, with expansions of signals which display resolved *J*_CH_ couplings) (right side). Predicted resonances *via* both methods (eqn (3) and (4), green squares; eqn (5), blue dots) additionally displayed as points; the black dotted line represents a perfect correlation.

This is in part attributed to dynamic broadening effects in solution but, more importantly, to broadening due to paramagnetic relaxation enhancement which is stronger for nuclei in close proximity to the paramagnetic metal centre. A general trend can be observed along the series of the complexes: both the magnitude of the chemical shift and the line width increase with increasing halogen mass. This is consistent with a greater localisation of the unpaired spin density on the halide for the lighter congeners, resulting in reduced spin density on the ligand framework, as indicated in DFT calculations (see SI Section 6.1.2). Additionally, the influence of the N–Ru–X angle on the chemical shift has been investigated for the Ru–Cl complex 2. For this purpose, *A*_iso_ values of nuclei C^1^–C^6^ as well as P have been calculated for different N–Ru–Cl angles (see SI Table S53). As expected, a smaller angle leads to higher *A*_iso_ values for C^1^–C^6^ while an angle of 180° results in smaller *A*_iso_ values. A clear trend however cannot be observed for the P atoms.

Complete assignment of all ^1^H and ^13^C resonances was achieved by predicting the chemical shifts of the respective nuclei using the two methods outlined above, as well as the resolved *J*_CH_ couplings for several carbon nuclei (see Fig. 8 expansions) in combination with 2D-NMR spectra. Only the assignment of H/C^2/4^ was not unambiguously possible by this method, and in this case the resonances were unambiguously assigned through analysis of the respective residual dipolar couplings (RDCs).^4*c*^ RDCs of paramagnetic complexes in solution NMR spectroscopy are the result of partial self-alignment in the magnetic field due to an anisotropic magnetic susceptibility of the molecule. They add to the scalar spin–spin *J*-coupling and can be evaluated using the observed total signal splittings:

6 ^1^*T* = ^1^*J* + Δ*v*^rdc^

The RDC of a paramagnetic molecule in solution in the approximation of axial magnetic susceptibility is given by^26^7
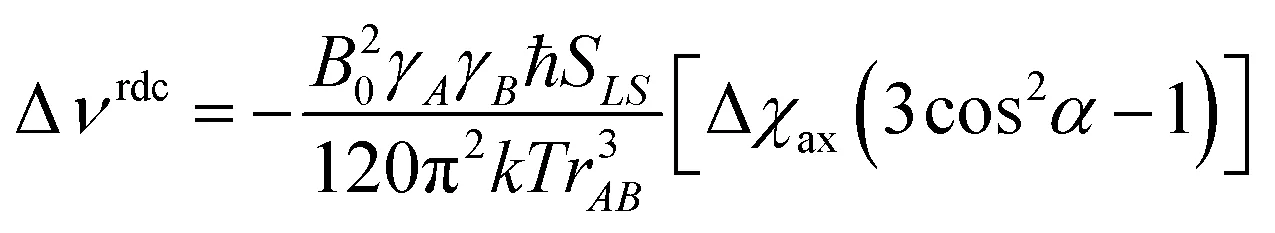
where *r*_*AB*_ is the vector between the two coupling nuclei *A* and *B* and *α* is the angle between the *AB* vector and the main magnetic axis (for further information see SI Section 6.4.2). As can be seen in eqn (7), the absolute sign of the Δ*v*_rdc_ is dependent on the angle of the coupling nuclei and the main magnetic axis and changes above and below the magic angle of approximately 54.74°. For the C^2^–H^2^ (85.3°) and the C^4^–H^4^ (49.3°) vectors of the Ru–Cl complex 2, a different absolute sign of the Δ*v*_rdc_ is thus expected. For C^2^–H^2^, a positive RDC and thus increased splitting are expected (in the given reference frame where the main magnetic axis is defined as the axis with the largest contribution to *χ*_iso_, making Δ*χ*_ax_ positive by definition, and when assuming the ^1^*J*_CH_ coupling as positive), while for C^4^–H^4^, a negative RDC is expected based on eqn (7).

On this basis, the resonances in the ^13^C-NMR spectra could be assigned unambiguously together with the ^1^H-NMR resonances by means of 2D-NMR. Thus, the ^13^C(−115.7 ppm), ^1^H (13.6 ppm), and ^1^*T*_CH_ = 158.5 Hz resonances were assigned to C^2^–H^2^ with positive RDC and the ^13^C(−132.3 ppm), ^1^H (29.1 ppm), and ^1^*T*_CH_ = 149.9 Hz resonances were assigned to C^4^–H^4^ with negative RDC (see Fig. 8). These results were further confirmed by investigating the temperature dependence of the observed splitting (see SI Table S56). The observed splitting of the resonance assigned to C^2^ decreases with temperature consistent with a positive RDC, while the observed coupling of the resonance assigned to C^4^ increases with temperature consistent with a negative RDC. The assignments of the C^2/4^/H^2/4^ nuclei of complexes 3–5 could be deduced from these results.

As part of a method validation to identify the optimal DFT functional for the computation of the hyperfine tensor ***A***, a screening was conducted based on calculated chemical shifts derived from a chosen set of *δ*_orb_ values and eqn (3) and (4) (see SI Section 6.4.1). These computed data were compared to the experimental resonances of the chlorido complex as a benchmark. The Fermi-contact contributions (*δ*_FC_) were calculated from the isotropic hyperfine coupling constants *A*_iso_ thus obtained, while the pseudo-contact shift contribution (*δ*_PC_) was calculated from the CASSCF calculated ***χ*** tensor.

Altogether, this enabled a complete theoretical modelling of *δ*_calc_ and a comparison to the experimentally measured chemical shifts, with the ωB97X-D3(BJ) functional, a semi-empirical range separated generalized gradient approximation hybrid functional developed by Head-Gordon,^28^ giving rise to the best agreement.

Based on these results, paramagnetic chemical shift contributions (*δ*_HF_) (including ^31^P and ^19^F when applicable; *vide infra*) were simulated after iterative optimisation of the ***χ*** tensor with the full ***A*** tensor according to eqn (5) in combination with DFT calculated *δ*_orb_ values at the same level of theory as the ***A*** tensor. The simulated spectra (blue lines) as well as a comparison of the predicted values for both methods *versus* the experimental results (blue dots and green squares *versus* dotted lines) are depicted in Fig. 8. For the prediction of the NMR resonances using both methods, CUVNMR, developed by one of the co-authors of this manuscript, was employed.^29^

Both methods perform well in the prediction of the ^1^H and ^13^C resonances for all four complexes, with the *δ*_orb_ + *δ*_FC_ + *δ*_PC_ method generally underestimating the paramagnetic contribution for resonances with large chemical shifts in ^13^C spectra and overestimating the highly shifted ^1^H resonances and the *δ*_orb_ + *δ*_HF_ approach leading to a more accurate prediction overall. In general, prediction of the ^13^C resonance tends to be more accurate with both methods compared to the modelled ^1^H spectra. In particular, resonances of the conformationally flexible H^9^ and H^11^ are predicted with less accuracy, which is thought to result from equilibria between rapidly exchanging low-energy conformers, contributing to an average molecular structure in solution. In particular, the dipolar interactions (represented in eqn (4) for the first method or the off-diagonal components of the ***A*** tensor elements in case of the latter) depend sensitively on the orientation of the molecule and thus cannot accurately be described based on a single conformer.

Given the precise prediction of the ^1^H and ^13^C-NMR chemical shifts, as well as previous success at detecting directly bonded nuclei in solution NMR, we aimed to observe the resonances of the directly bonded ^31^P (2–5) and ^19^F (3) nuclei based on the predicted chemical shift. Indeed, all ^31^P NMR resonances, as well as the fluoride resonance in 3, could be observed experimentally and are depicted in Fig. 9. As can be seen, a large chemical shift of *ca.* −3900 ppm is observed with a trend towards more negative shifts upon increasing the atomic number of the halide, with the iodido complex 5 exhibiting the most upfield shifted resonance. Interestingly, the ^19^F-NMR signal of 3 was observed at a similar absolute magnitude of chemical shift (*ca.* 3900 ppm) but with an opposite sign.

**Fig. 9:**
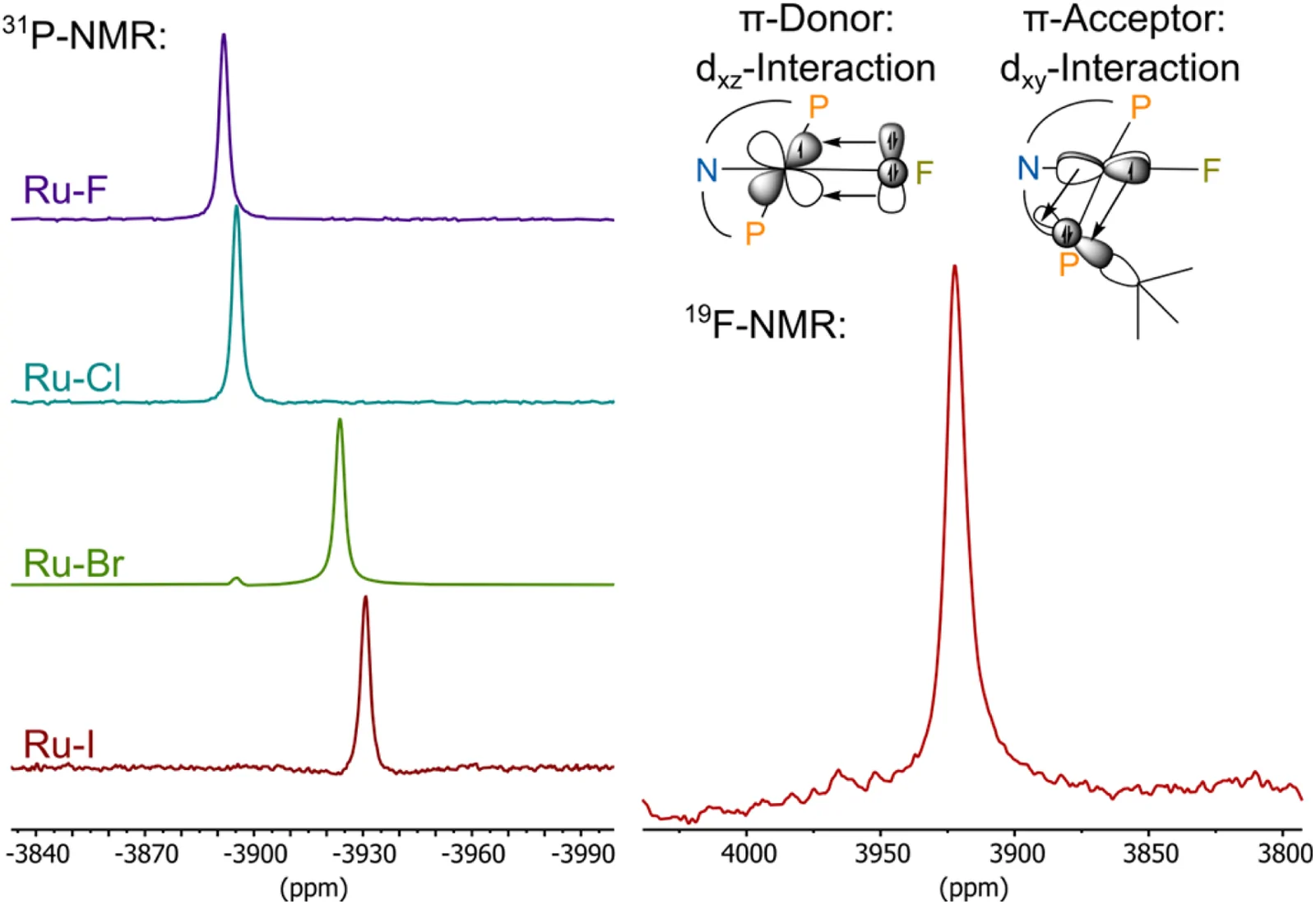
^31^P-NMR spectra of complexes 2–5 (C_6_D_6_, 242 MHz, 295 K) (left side), ^19^F-NMR spectra of complex 3 (C_6_D_6_, 567 MHz, 295 K) (right side bottom), and schematic representation of the π-interaction of the phosphorus and fluoride atoms with the unpaired electron located at the metal centre, as well as the filled s-orbital through which spin polarisation is mediated.

For nuclei in close proximity to the paragenetic centre, the contact term should provide the dominant contribution to the chemical shift. A different sign in the pNMR shift of two different nuclei with the same absolute sign in the gyromagnetic ratio implies a different absolute sign of *A*_iso_ for both nuclei, which is corroborated using the DFT calculations. Under this special circumstance (very large *δ*_FC_ contribution, which makes it possible to disregard *δ*_orb_ and *δ*_PC_) *A*_iso_ can be directly obtained from the chemical shift of the nucleus from eqn (3). Remarkably, the values obtained from the experiments (
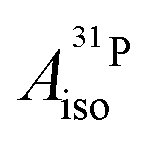
 = −22.1 MHz; 
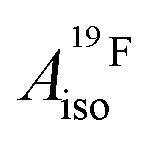
 = 52.8 MHz) are in very good agreement with the values obtained from the DFT calculations (
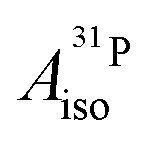
 = −21.5 MHz; 
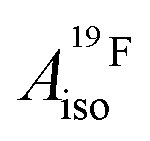
 = 50.6 MHz).

At a qualitative level, this can be understood from the interaction of the unpaired electron spin of the metal centred d-orbitals with the ligand centred frontier orbitals. Assuming that the dominant interaction arises from the unpaired electron occupying the d_*xz*_ or d_*xy*_ orbital, both of which have π-symmetry with regard to the metal–ligand axes, the fluoride can act as a π-donor ligand, *i.e.* unpaired spin density from a singly occupied d_*xz*_ orbital can be induced at the s-orbital of the fluorine (which is responsible for the hyperfine interaction) through a double spin-polarization mechanism.^30^ The unpaired electron in the d orbital induces, through spin polarization, spin density of opposite sign in the lone pairs of the fluoride atom. From there, the s-orbital can be spin polarized which induces unpaired spin density with the same orientation as the unpaired electron on the metal center, which results in a positive *A*_iso_.

On the other hand, the phosphorus atoms act as π-acceptor ligands. Direct spin delocalization from a singly occupied d_*xy*_ orbital into the antibonding P–R acceptor orbital is possible, which induces excess spin density with the same orientation to the unpaired electron. From there spin polarization of the electrons in the phosphorus s-orbital occurs which induces spin density with the opposite sign of spin compared to the unpaired electrons, resulting in a negative *A*_iso_ value (see Fig. 9). DFT calculated Mulliken spin populations, while to be treated with care in this context, also qualitatively support this explanation with negative spin populations on the P-atoms and a positive spin population of similar magnitude on the F-atom (see SI Table S11).

However, this would suggest a singly occupied d_*xy*_ orbital, apparently in contrast to results of the DFT calculated reduced spin populations, but generally consistent with the AILFT results presented above. The near degeneracy of the d_π_-orbitals derived from the computational studies supports the general consistency of this qualitative interpretation.

In summary, the prediction of the chemical shifts of the ^19^F and ^31^P NMR resonances with the two approaches outlined above gives acceptable agreement (see Table 4), while in the case of the classical approach *δ*_orb_ + *δ*_FC_ + *δ*_PC_, larger deviations of several 100 ppm are observed. This is to be expected as the point dipole approximation is known to fail for nuclei in close proximity to the paramagnetic centre. The dominant contribution from Fermi-contact coupling leads to a reasonable agreement overall, while the exact shift cannot be reproduced *via* this method. Simulation *via* the second method, with *δ*_HF_ prediction from ***χ*** tensor fitting, on the other hand, leads to excellent agreement with experimental results and an error of less than 75 ppm. For the exact prediction of the directly bound heteronuclei, this method is clearly shown to be more accurate and thus to be applied preferentially.

**Table 4:** Comparison between experimentally observed ^19^F and ^31^P resonances and the prediction based on both methods: (*δ*_FC_ + *δ*_PC_) eqn (3) +4 and (*δ*_HF_) eqn (5)

Nucleus	*δ*_exp_ [ppm]	*δ*_FC_ + *δ*_PC_ [ppm]	*δ*_HF_ [ppm]
^19^F(Ru–F)	3922.5	4475.3	3970.7
^31^P(Ru–F)	−3891.7	−3981.0	−3896.2
^31^P(Ru–Cl)	−3896.5	−4118.4	−3871.2
^31^P(Ru–Br)	−3923.7	−4153.1	−3888.9
^31^P(Ru–I)	−3930.8	−4225.2	−3998.5

### Temperature dependence of the chemical shifts and extraction of the axial zero-field splitting parameter *D* directly from solution pNMR

The temperature dependence of paramagnetically shifted NMR signals provides valuable insight into the magnetic properties of a given compound. To this end, variable temperature ^1^H–, ^13^C–, ^31^P-(2–5) and ^19^F-(3) pNMR experiments were performed.

Within the measured temperature range (235–353 K), all complexes and resonances exhibit a temperature dependent shift that is linear with respect to the inverse temperature (see SI Section 5.1). This is in accordance with the measured SQUID data, as a significant divergence from the Curie-type behaviour would usually only be expected if a significant population of a different spin isomer is achieved in the case of transition metals.^6*f*^ In particular, the ^31^P and ^19^F NMR resonances display a pronounced dependence on temperature over the range of 118 K, with the observed chemical shift changes spanning over 1150 ppm (Fig. 10a and b), while the simple inverse temperature dependence is retained.

**Fig. 10:**
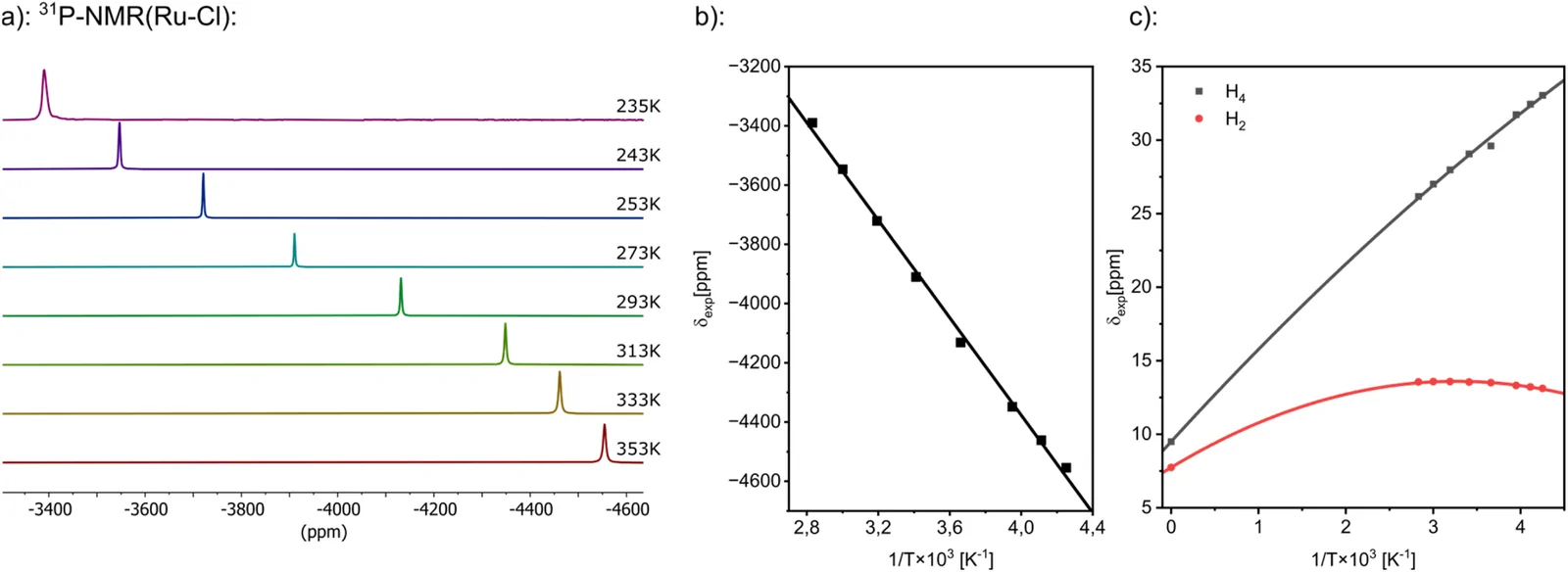
(a) ^31^P-NMR spectra of complex 2 in the temperature range between 235 and 353 K; (b) linear temperature dependence of the chemical shift of the ^31^P-NMR resonance of 3 against inverse temperature; (c) temperature dependence of the chemical shift of H_2_ and H_4_ resonance with added resonance in the high temperature limit.

In principle, the temperature dependence of the chemical shift of paramagnetic transition metal complexes is complicated and dependent on multiple orders of inverse temperature,^6*f*^ although in the analysed temperature range between 235 and 353 K, the *T*^−2^ and higher order dependences are not noticeable.

In fact, eqn (3) is obtained through a simplified substitution of ***χ*** expressed through a *T*^−1^ dependence only. The same holds true for eqn (4) if the ***χ*** components are expressed through the van Vleck equation, although in the case of the pseudo-contact contribution, an additional *T*^−2^ dependent term is usually included. In principle, even higher order dependencies can be considered, although these are seldomly included. In any case, at the high temperature limit, all paramagnetic contributions (*δ*_HF_) should vanish, and the chemical shift can be described solely by *δ*_orb_.

If the observed shifts are truly dominated by *T*^−1^ dependence, the intercept of the linear regression should be very close to the calculated *δ*_orb_ values. In particular, for the ^1^H NMR resonances, a significant deviation from the calculated *δ*_orb_ values is observed (Fig. 10c), implying some degree of higher order dependence of the chemical shift. In an approach by Autschbach *et al.*^31^ the temperature dependence of the chemical shift was investigated using the EPR pseudo-spin Hamiltonian. In their approach, the *T*^−2^ part is dependent on the axial zero-field splitting parameter *D* as well as *A*_iso_, Δ*A*, *g*, and Δ*g* (see SI Section 5.2).

By adding the calculated *δ*_orb_ values as the high temperature limit and fitting the *δ*_exp_ temperature dependence with a second-order polynomial, the degree of *T*^−2^ dependence can be determined. Based on calculated values for *A*_iso_, Δ*A* (obtained from DFT calculations), *g,* and Δ*g* (obtained from CASSCF calculations), *D* can be extracted from the *T*^−2^ dependence using the following relation:8
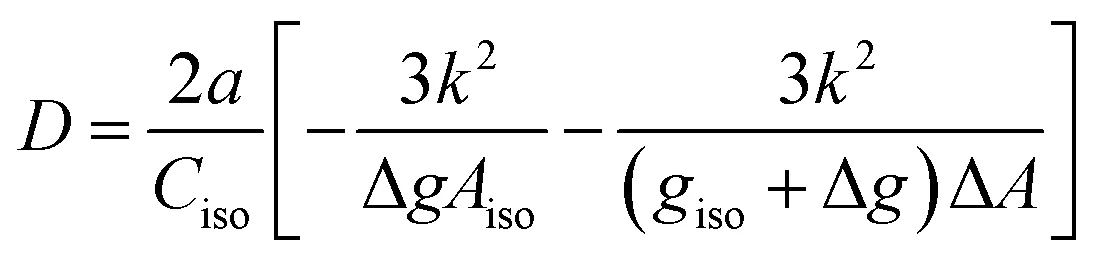
where *C*_iso_ is a constant pre-factor and *a* is the factor obtained from the polynomial fit for the second-order dependence from experimental values.

Since the signals of protons H^2^ and H^4^ displayed pronounced *T*^−2^ dependence as well as a relatively high degree of conformational rigidity and could be modelled well by the approaches mentioned above, these were chosen as a basis for the calculation of *D*. The obtained values for complexes 2–5 are given in Table 5. *D* values estimated from VT-NMR are in reasonable agreement with values obtained experimentally from SQUID magnetometry as well as values obtained from CASSCF calculations. Deviations likely arise as a result of the interconversion of the ligand backbone in the pNMR experiment time scale leading to an effective *C*_2v_ symmetry in solution and therefore to an averaged interaction over multiple conformers. From VT-NMR data, it was also possible to obtain *D* values for the fluorido complex 3, for which the limited stability of the complex made it impossible to obtain them from SQUID measurements.

**Table 5:** Calculated *D* values from *T*^−2^ dependence in comparison to values obtained from SQUID and CASSCF calculations

[cm^−1^]	*D* (NMR)	*D* (SQUID)	*D* (CASSCF)
Ru–F (3)	134 ± 80	—	223
Ru–Cl (2)	182 ± 63	229	217
Ru–Br (4)	173 ± 66	238	215
Ru–I (5)	181 ± 62	257	214

Notably, the absolute sign of *D* in the presence of a magnetic field can be assigned unambiguously from this method. This is of particular value because it is often unreliable when obtained only from SQUID magnetometry, as well as CASSCF calculations when rhombicity is close to the physical limit of |*E*/*D*| ≈ 1/3, as is the case for the ruthenium complexes of this study.

## Conclusion

In this study the synthesis and characterisation of a series of square-planar Ru(ii) halide complexes with an intermediate-spin *S* = 1 ground state are reported. Their electronic and magnetic properties were investigated by UV-VIS spectroscopy as well as SQUID magnetometry. Special attention was given to the assignment of the pNMR spectra as the complexes displayed very well resolved spectra. This even allowed the observation of the directly bound ^31^P and ^19^F NMR resonances at very large chemical shifts (over −3900 ppm for ^31^P and over +3900 ppm for ^19^F).

Using two approaches, one fully computational and one semi-empirical involving the fitting of the magnetic susceptibility tensor ***χ***, it has been possible to fully assign and quantitatively predict the experimental spectra and simulate them, which has led to good agreement with experimental results over the whole series of complexes. Additionally, the temperature dependence of the paramagnetic shifts was investigated and allowed estimation of the values of the axial zero-field splitting parameter (*D* > 130 cm^−1^). These were found to be in good agreement with values obtained from SQUID magnetometry as well as CASSCF calculations. Such findings may contribute to expanding the role of solution pNMR spectroscopy as a useful tool for the analysis of transition metal complexes. In combination with modern computational methods, which are able to predict and simulate the NMR spectra in a generalisable approach, this allows deeper insights into the magnetoelectronic properties of paramagnetic substances complementary to more established methods such as SQUID magnetometry or EPR.

## Author contributions

L. K. P. D.: conceptualization (equal), investigation (lead), methodology (lead), data curation (equal) visualization (lead), writing, review and editing (equal). C. S. V. W.: investigation (supporting). K. W.: investigation (supporting). T. B.: data curation (supporting). M. E.: data curation (supporting), review and editing (supporting) J. B.: data curation (supporting) L. H. G.: conceptualization (equal), project administration (lead), resources (lead), supervision (lead), writing, review and editing (equal). The manuscript was written through contributions of all authors.

## Conflicts of interest

There are no conflicts to declare.

## Supplementary Material

SC-017-D6SC04475A-s001

SC-017-D6SC04475A-s002

## Data Availability

CCDC 2557324 (Ru-Cl 2), 2557325 (Ru-I 5), 2557326 (Ru-F 3) and 2557327 (Ru-Br 4) contain the supplementary crystallographic data for this paper.^32*a*–*d*^ Spectroscopic data as well as key computational files are accessible from heiDATA: https://doi.org/10.11588/DATA/ROEKDF. Supplementary information (SI): experimental and computational details, including Cartesian coordinates as well as NMR spectra, UV-vis spectra, SQUID data and crystallographic data. See DOI: https://doi.org/10.1039/d6sc04475a.
