# Supplementary material for: NMR of nuclei bound to a paramagnetic metal centre: the case of S = 1 square-planar ruthenium(ii)
Source: Chem Sci. 2026 Aug 19;17(40):19364–75. doi: 10.1039/d6sc04475a (PMC13523777; doi:10.1039/d6sc04475a)
Supplement: Supplementary file 1 [file SC-017-D6SC04475A-s001.pdf]

Supplementary Information (SI) for Chemical Science.
This journal is © The Royal Society of Chemistry 2026

Supplementary Information

NMR of Nuclei Bound to a Paramagnetic Metal Centre: The Case of *S* = 1 Square Planar Ruthenium(II)

Leon K. Paschai Darian, Charlotte S. V. Weiß, Kim Warth, Tim Bruckhoff, Joachim Ballmann, Markus Enders, and Lutz H. Gade *

Table of Contents

1. Experimental Procedures ... 3

1.1 General Remarks ... 3

1.2 Synthesis of the $PNP^{tBu}RuX$ Halide Complexes ... 4

1.2.1 Synthesis of $PNP^{tBu}RuCl$ **2** ... 4

1.2.2 Synthesis of Synthesis of $PNP^{tBu}RuBr$ **4** ... 4

1.2.3 Synthesis of Synthesis of $PNP^{tBu}RuI$ **5** ... 4

1.2.4 Synthesis of Synthesis of $PNP^{tBu}RuF$ **3** ... 5

2. NMR Spectra ... 6

2.1 Room Temperature NMR Spectra of the Ru Complexes ... 6

2.2 General Acquisition procedure ... 16

3. UV-VIS Spectra ... 17

4. SQUID Magnetometry ... 19

5. Variable Temperature NMR experiments ... 21

5.1 VT-NMR ^{1}H, ^{13}C, ^{19}F and ^{31}P spectra of the halide complexes ... 21

5.2 Estimation of the T^{-2} dependence for ^{1}H resonances of H^2 and H^4 and calculation of *D* ... 36

6. Computational Section ... 39

6.1 TD-DFT calculations ... 40

6.1.1 Excitations energies and Oscillator Strength of the Halide complexes ... 40

6.1.2 NTOs of Transition 6, Spin density and frontier MOs of **2** ... 43

6.2 CASSCF Results ... 45

6.3 Computation of HCF Tensors ... 64

6.4 Prediction and Simulation of the NMR spectra ... 73

6.4.1 Calculation of chemical shift with two approaches ... 73

6.4.2 Investigation of the influence of the N-Ru-X angle on A, for the Ru-Cl complex **2** ... 80

6.4.3 Assignment of the H^2/C^2 and H^4/C^4 resonances based on residual dipolar couplings (RDCs) ... 80

6.5 Cartesian coordinates of the Complexes ... 82

7. X-Ray Crystal Structure Determinations ... 93

References ... 96

1. Experimental Procedures

1.1 General Remarks

All experiments were conducted under an atmosphere of dry and oxygen-free argon by using standard Schlenk or glovebox techniques. Argon 5.0 was used and further dried by passing over a column of phosphorus pentoxide. Glassware was heated to 130°C overnight and evacuated while still hot. Diethylether, toluene, and pentane were purified using an MBraun Solvent Purification System (SPS). Benzene was dried over potassium and distilled prior to use. Deuterated solvents were dried over sodium/potassium (C_6D_6, toluene-d_8,). Furthermore, solvents not purified by the (SPS) were degassed by freeze-pump-thaw cycles. The protioligand $PNP^{tBu}H$ (**1**)[1] and PPh_4F[2] were prepared according to literature procedures. All other chemicals were purchased from commercial suppliers and used as received.

Samples for NMR spectroscopy were prepared under argon in 5 mm Wilmad J. Young Tubes or in NewEra 5 mm Pyrex tubes. One and two dimensional ^1H, ^{13}C, ^{19}F and ^{31}P NMR spectra were recorded on a Bruker Avance II 400 MHz or on a Bruker Avance 600 III spectrometer. Residual (undeuterated) solvent served as reference for ^1H and ^{13}C NMR spectra. ^{31}P NMR spectra were referenced to external $P(OMe)_3$ (141.0 ppm with respect to 85 % H3PO4 at 0.0 ppm). ^{19}F NMR spectra were referenced to external $CFCl_3$ ($C^{19}F(^{35}Cl_2)(^{37}Cl)$ isotopologue at 0.0 ppm). Chemical shifts δ are given in parts per million (ppm), coupling constants J (for simplicity and consistency the observed total couplings are referred to, in the experimental part, as J instead of T) in Hertz (Hz). Signal multiplicities are stated by common abbreviations (e.g. s – singlet, d – doublet, dd – doublet of doublets). Measurements of magnetic susceptibility in solution via Evans method were conducted with an NMR tube containing the sample in a known concentration in the solvent, and a inner capillary containing only the solvent. Values obtained with this method were corrected with the aid of pascal constants.[3]

Mass spectra were recorded by the department of Organic Chemistry of the University of Heidelberg under the direction of Dr. J. Gross on a JEOL JMS-700 magnetic sector by liquid injection FD ionization (LIFDI) technique.

UV-VIS spectra were recorded on a Varian Cary 5000 spectrometer in cuvettes fitted with an airtight ampoule adapter.

Magnetometry measurements of powder samples were carried out in polypropylene capsules using a Quantum Design MPMS3 SQUID-Magnetometer. Background measurements were subtracted from the data to obtain the spectra.

X-ray analyses were performed by.Prof. Dr. Joachim Ballmann in the X-ray laboratory of the Department of Inorganic Chemistry at the University of Heidelberg with an Agilent Supernova E diffractometer. The obtained structures were solved and refined by Prof. Dr. Joachim Ballmann.

1.2 Synthesis of the PNP^{tBu}RuX Halide Complexes

*1.2.1 Synthesis of PNP^{tBu}*RuCl **2**

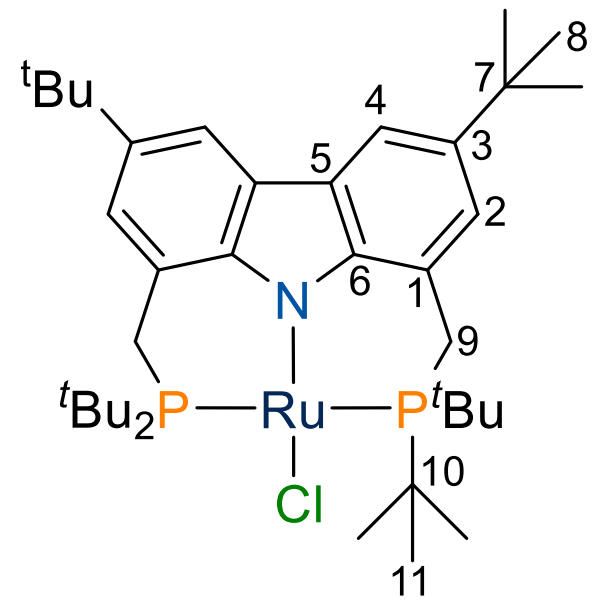

To a solution of 3,6-di-tert-butyl-1,8-bis((di-tert-butylphosphaneyl)methyl)-9H-carbazole (750 mg, 1.26 mmol, 1.00 eq) in toluene (50 mL) lithium-bis(trimethylsilyl)-amide (271 mg, 1.39 mmol, 1.10 eq) dissolved in toluene (10 mL) was added dropwise, changing the colour of the solution from light yellow to bright orange. After stirring for 30 minutes dichloro(p-cymene)ruthenium (413 mg, 1.32 mmol, 1.05 eq) was added turning the solution dark blue. The mixture was allowed to stir for three hours, after which the solution was filtered over a Celite pad, and the filter pad was washed with toluene (2 × 5 mL). The solvent was removed *in vacuo* and the remaining solid was washed with cold pentane (2 × 10 mL), and a mixture of cold pentane and diethyl ether (1:1, 10 mL). The solid was dried *in vacuo* affording PNP^{tBu}RuCl **2** in 60 % yield (550 mg, 752 µmol) as a dark-blue solid. **^{1}H NMR** (600 MHz, C_6D_6) δ [ppm] = 29.1 (s. 2H, H4), 13.6 (s, 2H. H2), 4.4 (s, 18H, H8), −6.2 (s, 36H, H11). **^{13}C NMR** (151 MHz, C_6D_6) δ [ppm] = 507.2 (s, 2C, C3), 446.0 (s, 2C, C1), 411.1 (s, 2C, C5), 178.0 (s, 12C, C11), 129.8 (q, J_{CH} = 125.7 Hz 6C, C8), −65.9 (s, 2C, C7), −115.7 (d, J_{CH} = 158.5 Hz, 2C, C2), −132.3 (d, J_{CH} = 149.9 Hz, 2C, C4), -240.0 (s, 4C, C10), −281.0 (t, J_{CH} = 133.7 Hz, 2C, C9), −372.4 (s, 2C. C6). **^{31}P NMR** (161 MHz, C_6D_6) δ [ppm] = −3896.5. **UV-VIS** [nm] (pentane) = 589 (ε = 4620 $M^{-1}cm^{-1}$), 401 (ε = 1400 $M^{-1}cm^{-1}$), 344 (ε = 4660 $M^{-1}cm^{-1}$), 299 (ε = 17110 $M^{-1}cm^{-1}$), 282 (ε = 16950 $M^{-1}cm^{-1}$). **MS** (LIFDI, toluene): calc for $[C_{38}H_{62}ClNP_2Ru]^+$ 731.31 found: 731.35. **SQUID:** D = 256 cm^{-1}, $|E/D|$ = 0.33, g_{iso} = 2.03. **Magnetic Susceptibility**: μ_{eff} = 2.76±0.16 μ_B (Evans, C_6D_6, 295 K).

*1.2.2 Synthesis of Synthesis of PNP^{tBu}*RuBr **4**

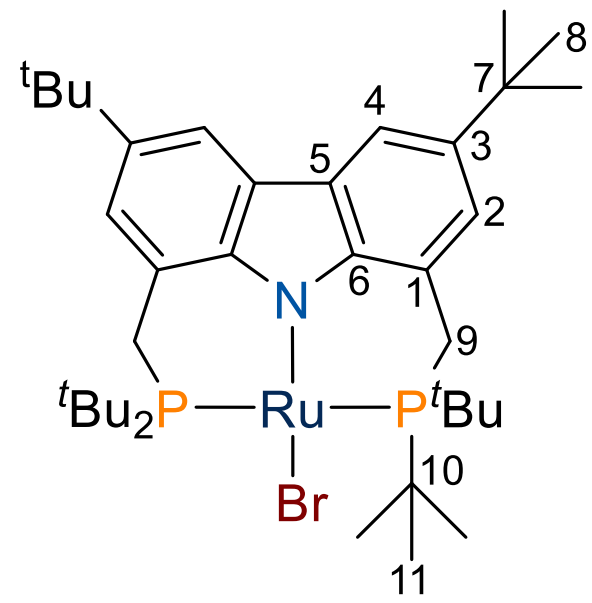

2 (60.0 mg, 82.0 µmol, 1.0 eq) was dissolved in toluene (5 ml) and TMS-Br (16.2 µl, 123 µmol, 1.5 eq) was added. The resulting solution was stirred for 5 h upon which the solution changed from dark blue to a dark turquoise. Subsequently the solvent was removed *in vacuo* and the remaining solid was washed with cold pentane (2 × 5 mL). After removing residual solvent *in vacuo* the product PNP^{tBu}RuBr **4** was afforded as a dark-turquoise solid in 83% yield (53.0 mg, 68.3 µmol). **^{1}H NMR** (600 MHz, C_6D_6) δ [ppm] = 29.5 (s. 2H, H4), 14.4 (s, 2H. H2), 4.4 (s, 18H, H8), −8.0 (s, 36H, H11). **^{13}C NMR** (151 MHz, C_6D_6) δ [ppm] = 527.6 (s, 2C, C3), 464.5 (s, 2C, C1), 437.9 (s, 2C, C5), 134.0 (q, J_{CH} = 125.4 Hz 6C, C8), −70.4 (s, 2C, C7), −128.6 (d, J_{CH} = 158.8 Hz, 2C, C2), −140.0 (d, J_{CH} = 150.4 Hz, 2C, C4), -241.4 (s, 4C, C10), −274.1 (t, J_{CH} = 133.6 Hz, 2C, C9), −376.4 (s, 2C. C6). **^{31}P** NMR (161 MHz, C_6D_6) δ [ppm] = −3923.7. **UV-VIS** [nm] (pentane) = 605 (ε = 4970 $M^{-1}cm^{-1}$), 401 (ε = 1970 $M^{-1}cm^{-1}$), 346 (ε = 5390 $M^{-1}cm^{-1}$), 300 (ε = 16570 $M^{-1}cm^{-1}$), 284 (ε = 17390 $M^{-1}cm^{-1}$). **MS** (LIFDI, toluene): calc for $[C_{38}H_{62}BrNP_2Ru]^+$ 775.26 found: 775.38. **SQUID:** D = 234 cm^{-1}, $|E/D|$ = 0.33, g_{iso} = 1.65. **Magnetic Susceptibility**: μ_{eff} = 2.75±0.16 μ_B (Evans, C_6D_6, 295 K).

*1.2.3 Synthesis of Synthesis of PNP^{tBu}*RuI **5**

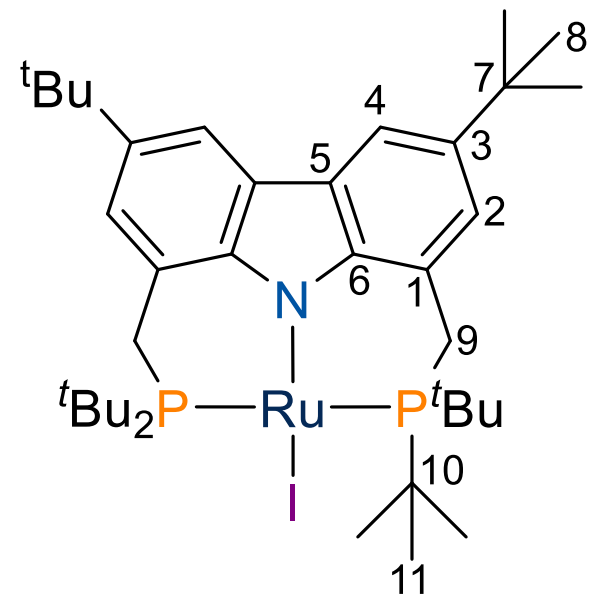

2 (60.0 mg, 82.0 µmol, 1.0 eq) was dissolved in toluene (5 ml) and TMS-I (16.8 µl, 123 µmol, 1.5 eq) was added. The resulting solution was stirred for 5 h upon which the solution changed from dark blue to dark green. Subsequently the solvent was removed *in vacuo* and the remaining solid was washed with cold pentane (2 × 5 mL). After removing residual solvent *in vacuo* the product PNP^{tBu}RuI **5** was afforded as a dark-green solid in 54% yield (36.5 mg, 44.4 µmol). **^{1}H NMR** (600 MHz, C_6D_6) δ [ppm] = 29.6 (s. 2H, H4), 15.4 (s, 2H. H2), 4.2 (s, 18H, H8), −13.8 (s, 36H, H11). **^{13}C NMR** (151 MHz, C_6D_6) δ [ppm] = 554.6 (s, 2C, C3), 487.2 (s, 2C, C1), 474.8 (s, 2C, C5), 138.5 (q, J_{CH} = 123.7 Hz 6C, C8), −75.4 (s, 2C, C7), −143.9 (d, J_{CH} = 157.8 Hz, 2C, C2), −148.3 (d, J_{CH} = 147.5 Hz, 2C, C4), -241.4 (s, 4C, C10), −258.8 (t, J_{CH} = 132.0 Hz, 2C, C9), −371.2 (s, 2C. C6). **^{31}P NMR** (161 MHz, C_6D_6) δ [ppm] = −3930.8. **UV-VIS** [nm] (pentane) = 628 (ε = 5000 $M^{-1}cm^{-1}$), 403 (ε = 2480 $M^{-1}cm^{-1}$), 348 (ε = 4720 $M^{-1}cm^{-1}$), 300 (ε = 15880 $M^{-1}cm^{-1}$), 284 (ε = 17250 $M^{-1}cm^{-1}$). **MS** (LIFDI, toluene): calc for $[C_{38}H_{62}INP_2Ru]^+$ 823.24 found: 823.29. **SQUID:** D = 247 cm^{-1}, $|E/D|$ = 0.33, g_{iso} = 1.91. **Magnetic Susceptibility**: μ_{eff} = 2.91±0.22 μ_B (Evans, C_6D_6, 295 K).

1.2.4 Synthesis of Synthesis of PNP^{tBu}RuF **3**

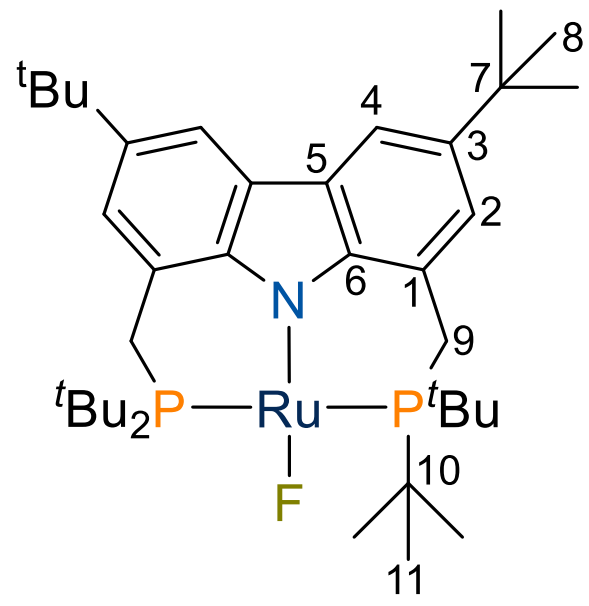

To a solution of **5** (60 mg, 79.9 µmol, 1.00 eq) in benzene (5 mL) fluorotetraphenylphosphorane (28.8 mg, 80.2 µmol, 1.10 eq) was added and the resulting solution was stirred for 5 minutes upon which the color changed from a deep turquoise to a deep purple and a white precipitate formed. The solution was filtered through a syringe filter and placed in a freezer at −40°C. The frozen solution was lyophilized and the remaining solid was washed with cold pentane (1 × 5 mL). The solid was dried *in vacuo* affording PNP^{tBu}RuF **3** in 60 % yield (53.0 mg, 43.9 µmol) as a dark-purple solid. **^{1}H NMR** (600 MHz, C_6D_6) δ [ppm] = 28.9 (s. 2H, H4), 20.0 (s. 4H, H9),11.9 (s, 2H. H2), 4.7 (s, 18H, H8), −1.9 (s, 36H, H11). **^{13}C NMR** (151 MHz, C_6D_6) δ [ppm] = 444.6 (s, 2C, C3), 395.7 (s, 2C, C1), 352.5 (s, 2C, C5), 194.6 (s, 12C, C11), 118.0 (q, J_{CH} = 125.4 Hz 6C, C8), −52.1 (s, 2C, C7), −76.0 (d, J_{CH} = 157.6 Hz, 2C, C2), −105.6 (d, J_{CH} = 148.5 Hz, 2C, C4), -254.1 (s, 4C, C10), −311.4 (t, J_{CH} = 131.8 Hz, 2C, C9), −342.1 (s, 2C. C6). **^{19}F NMR** (567 MHz, C_6D_6) δ [ppm] = 3922.5. **^{31}P NMR** (161 MHz, C_6D_6) δ [ppm] = −3891.7. **MS** (LIFDI, toluene): calc for $[C_{38}H_{62}FNP_2Ru]^+$ 715.34 found: 715.36. **Magnetic Susceptibility**: μ_{eff} = 2.37 μ_B (Evans, C_6D_6, 295 K).

2. NMR Spectra

2.1 Room Temperature NMR Spectra of the Ru Complexes

Compound **2:**

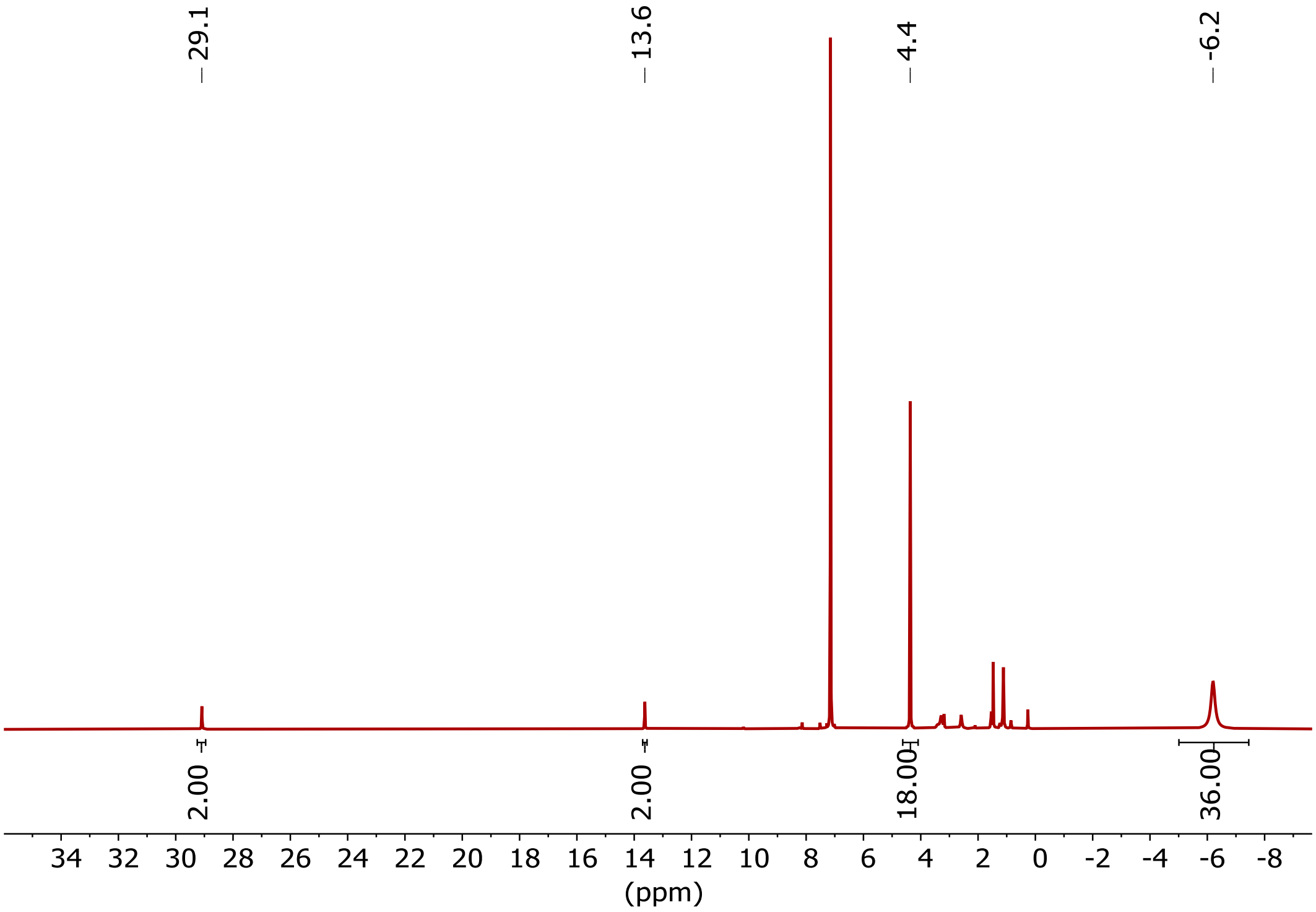

Figure S 1: ^{1}H NMR spectrum of **2** (C_6D_6, 600 MHz, 295 K).

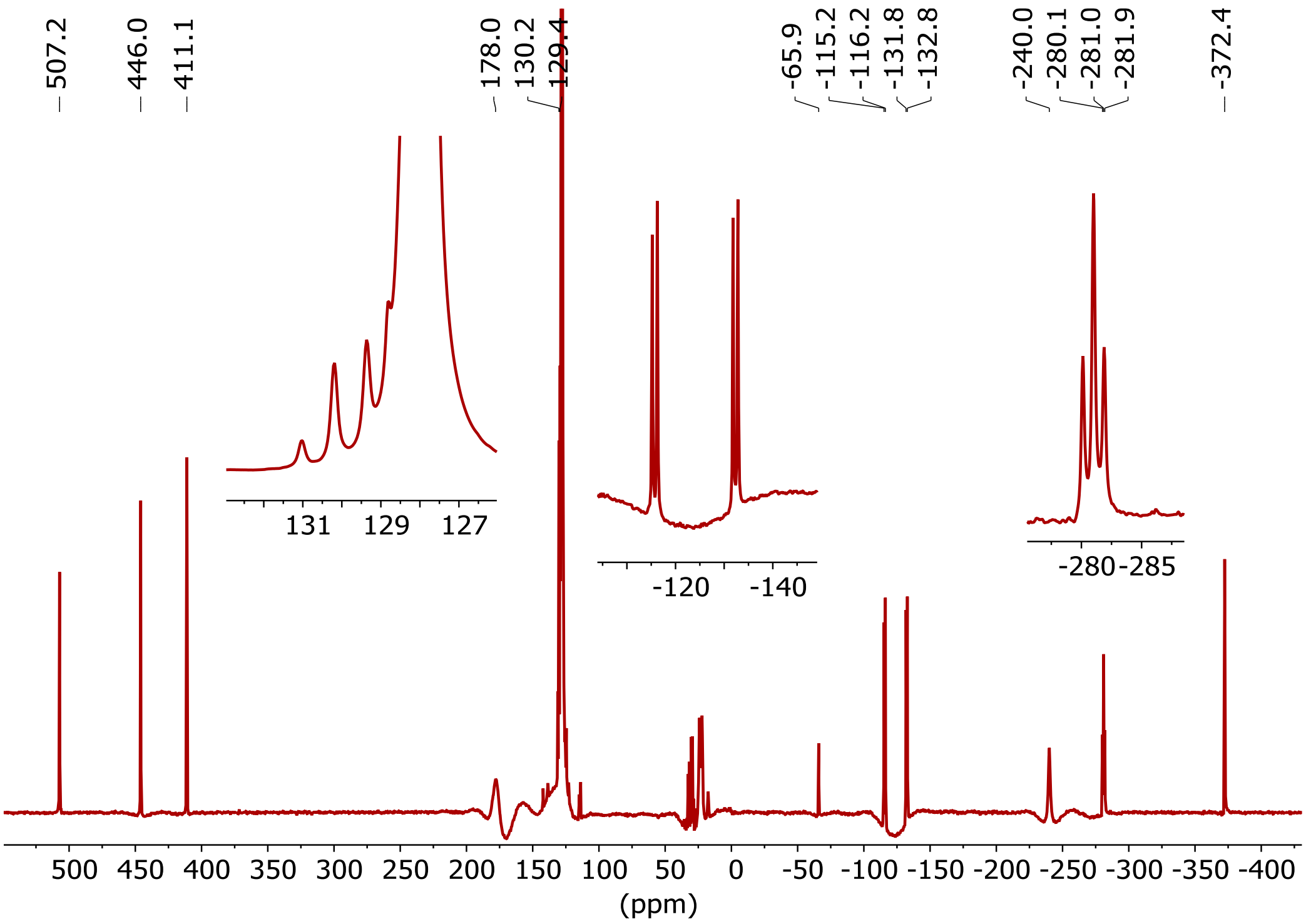

Figure S 2: ^{13}C NMR spectrum of **2** (C_6D_6, 151 MHz, 295 K).

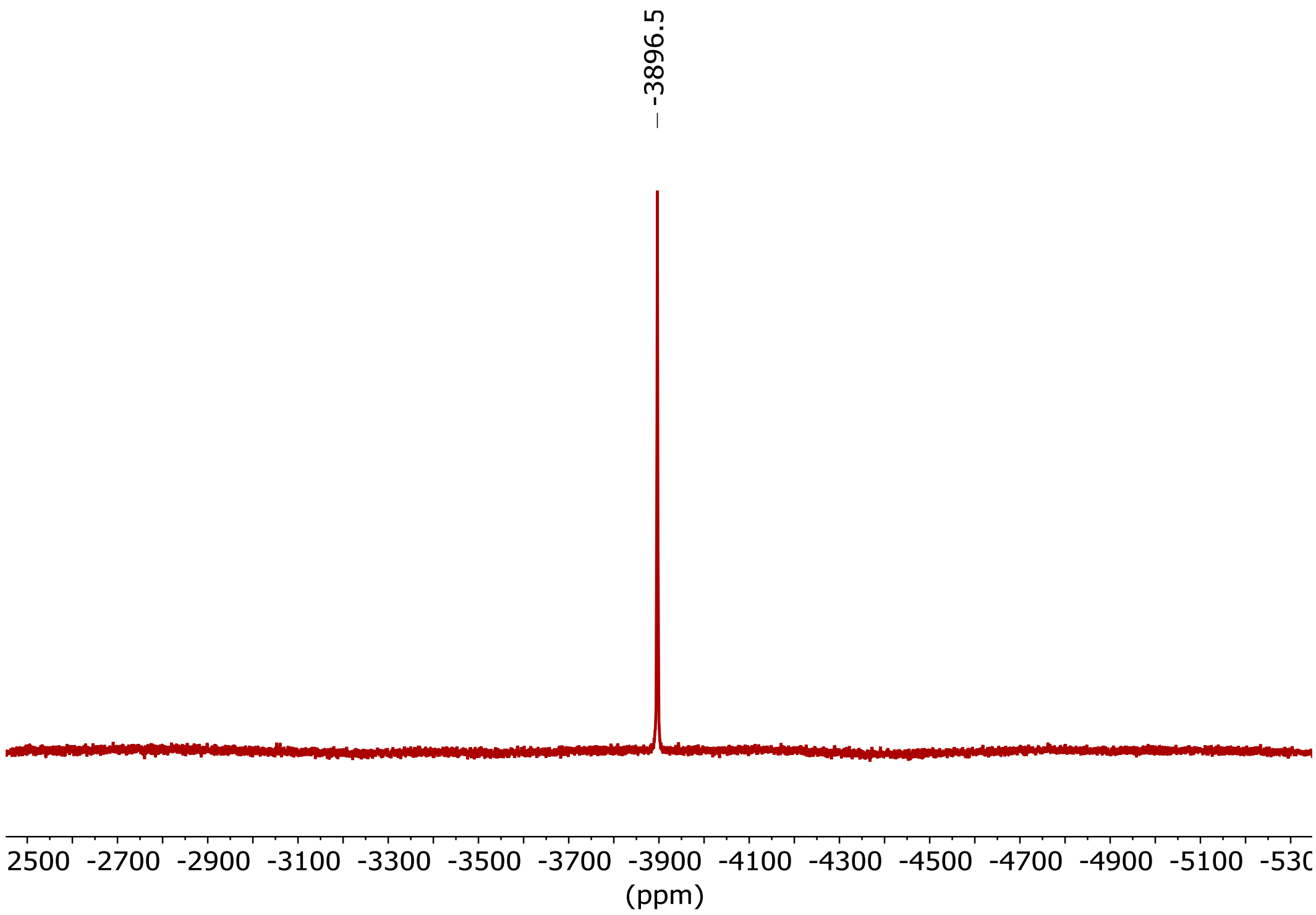

Figure S 3: ^{31}P NMR spectrum of **2** (C_6D_6, 161 MHz, 295 K).

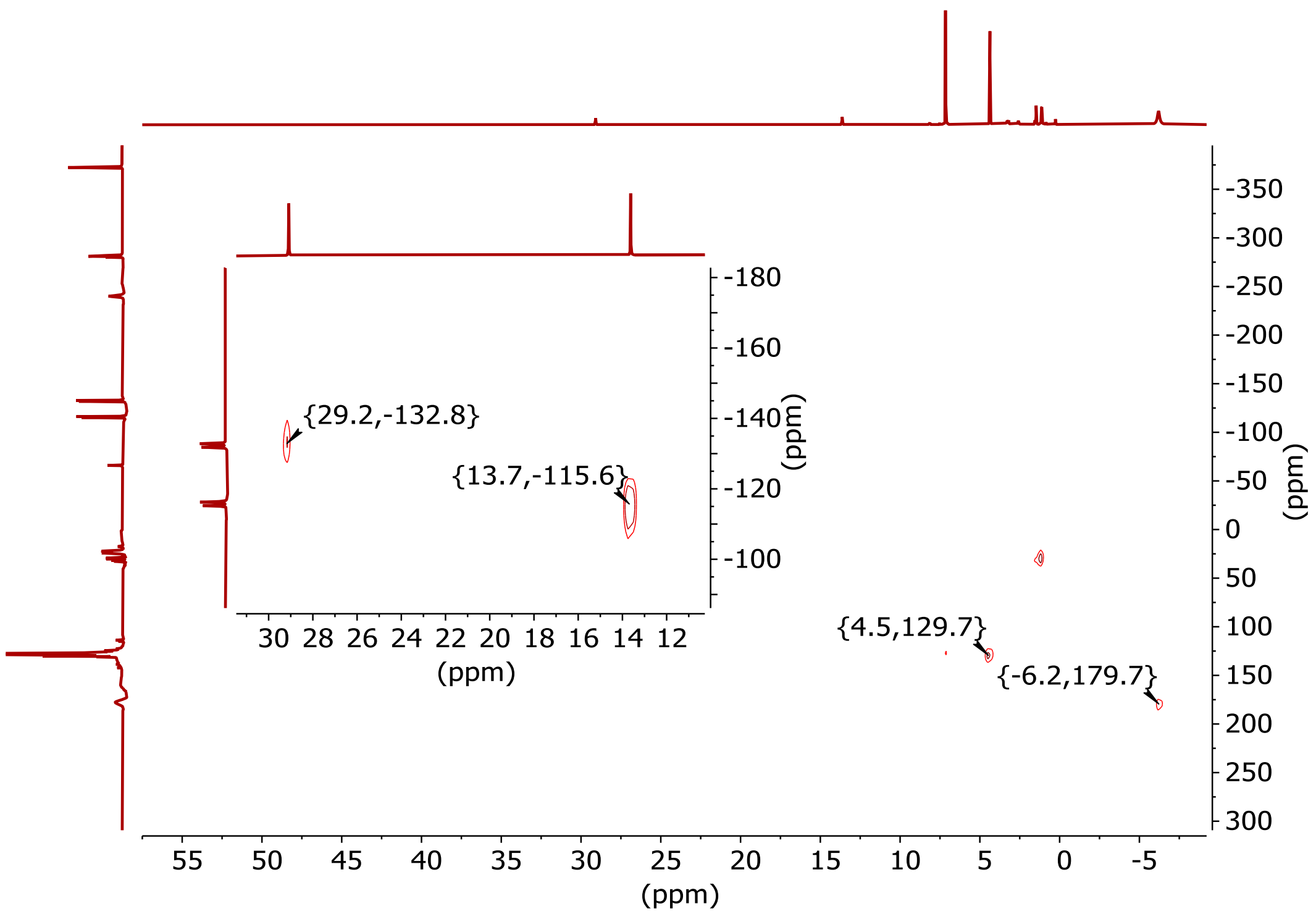

Figure S 4: ^{1}H-^{13}C HSQC spectrum of **2** (C_6D_6, 400/101 MHz, 295 K).

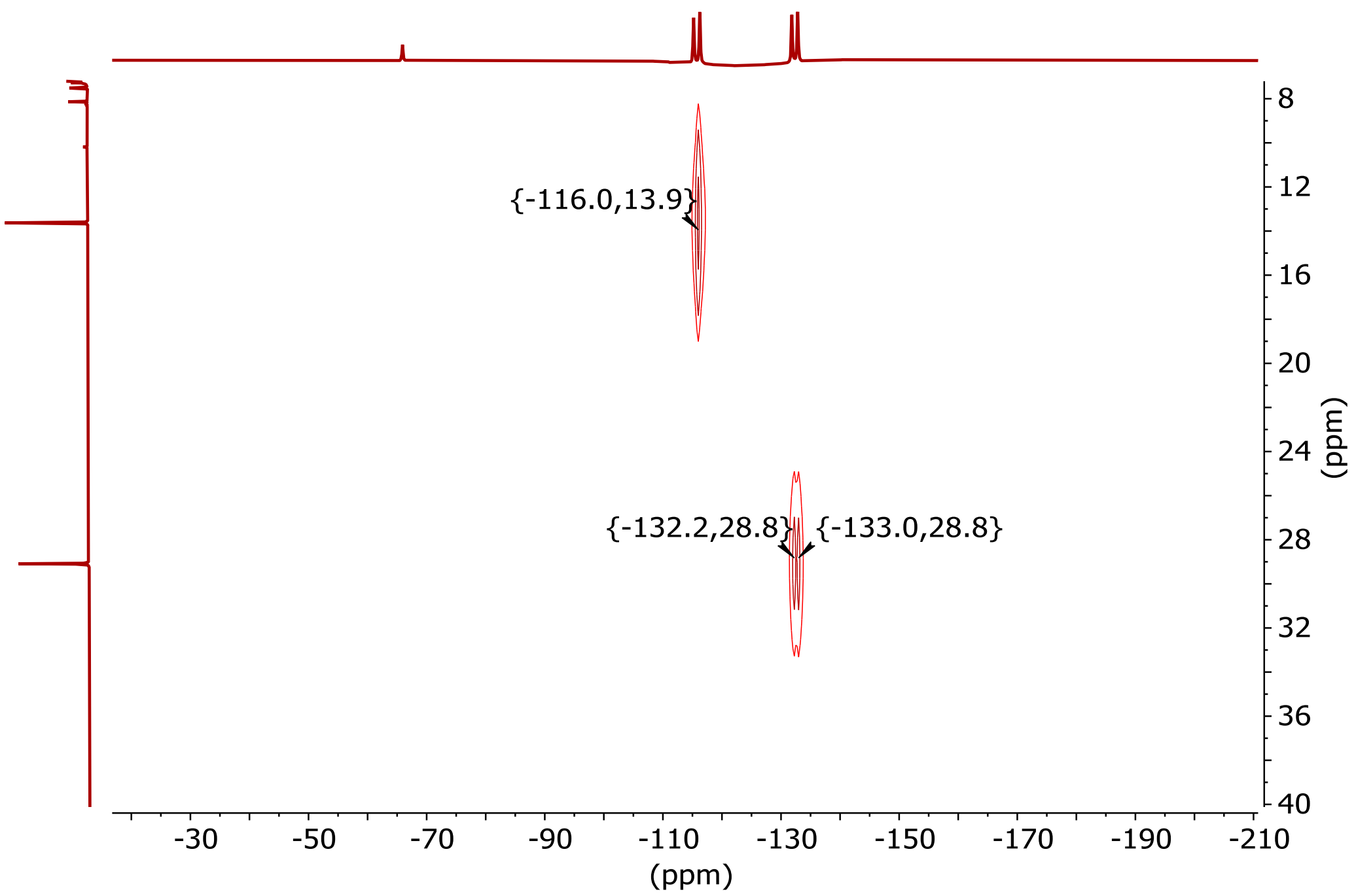

Figure S 5: ^{13}C-^{1}H HETCOR spectrum of **2** (C_6D_6, 151/600 MHz, 295 K).

Compound **4:**

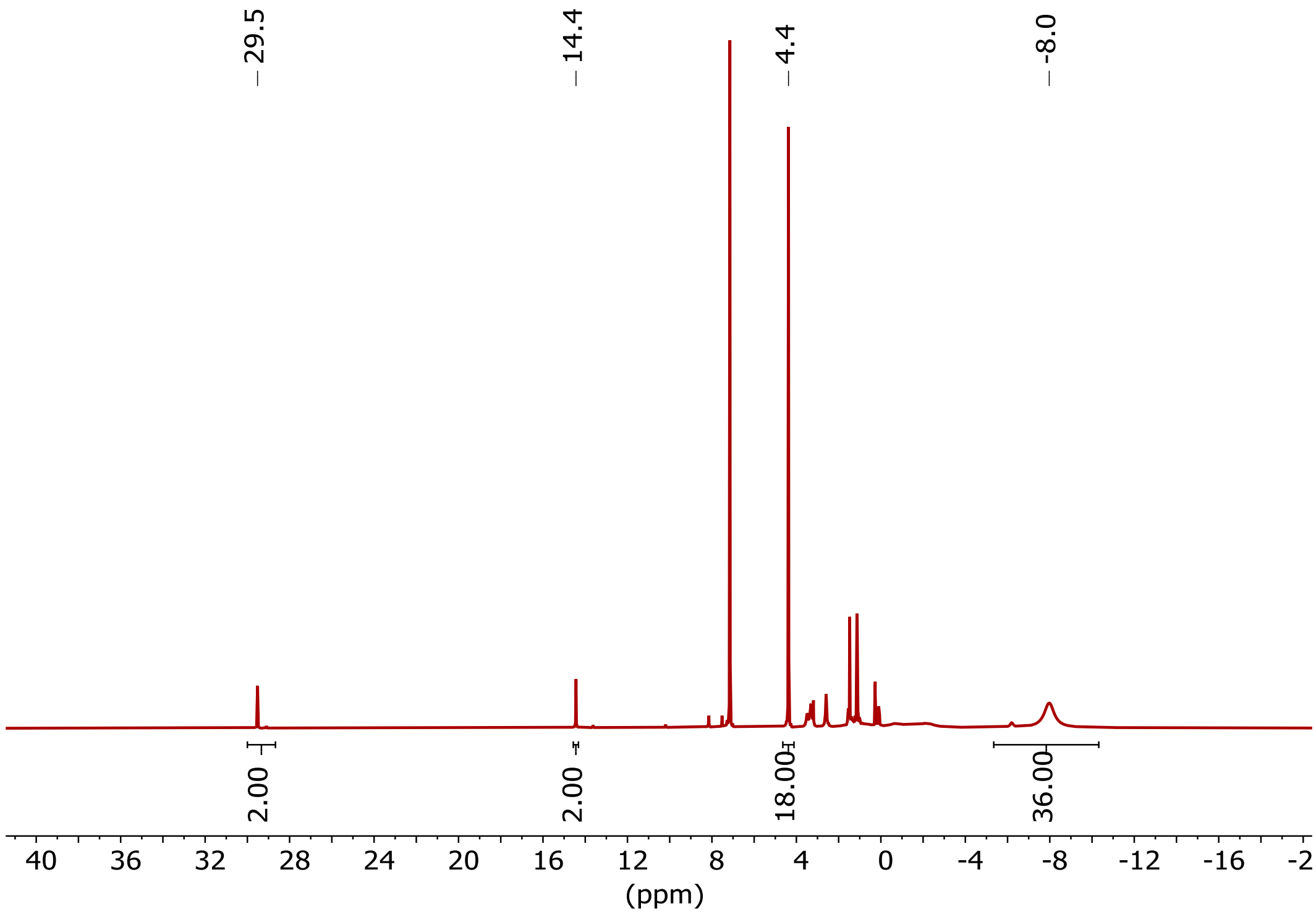

Figure S 6: ^{1}H NMR spectrum of **4** (C_6D_6, 600 MHz, 295 K).

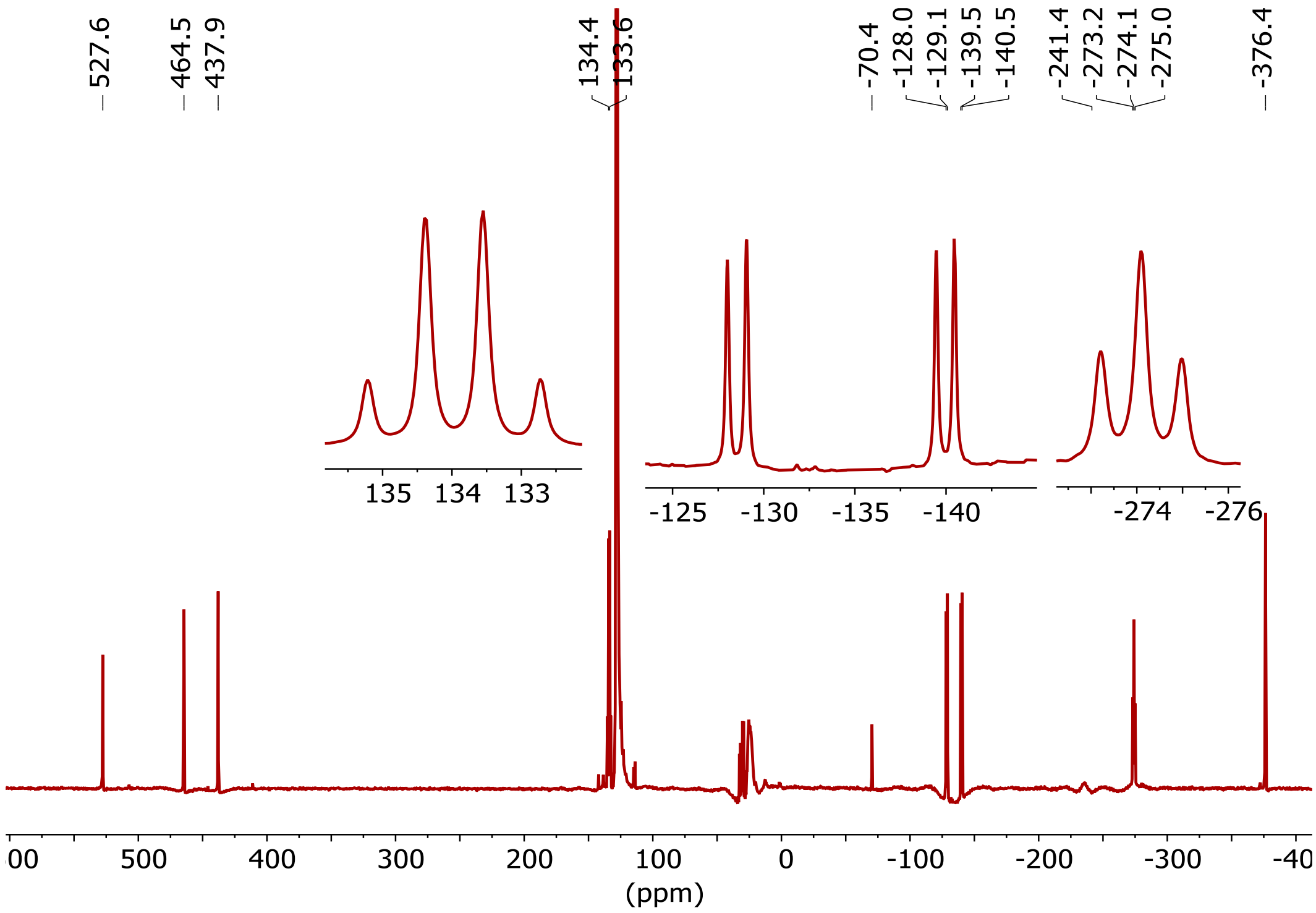

Figure S 7: ^{13}C NMR spectrum of **4** (C_6D_6, 151 MHz, 295 K).

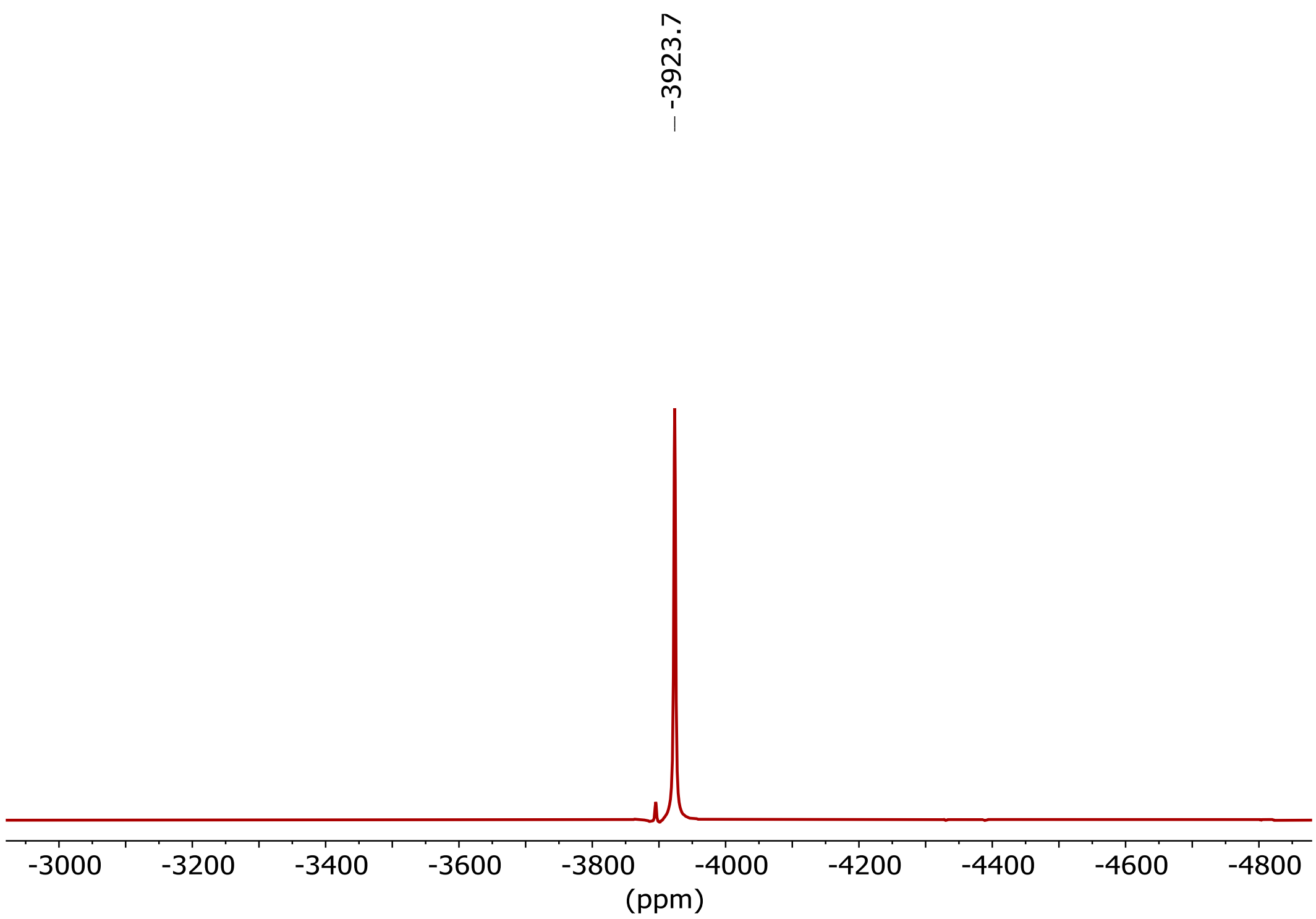

Figure S 8: ^{31}P NMR spectrum of **4** (C_6D_6, 161 MHz, 295 K).

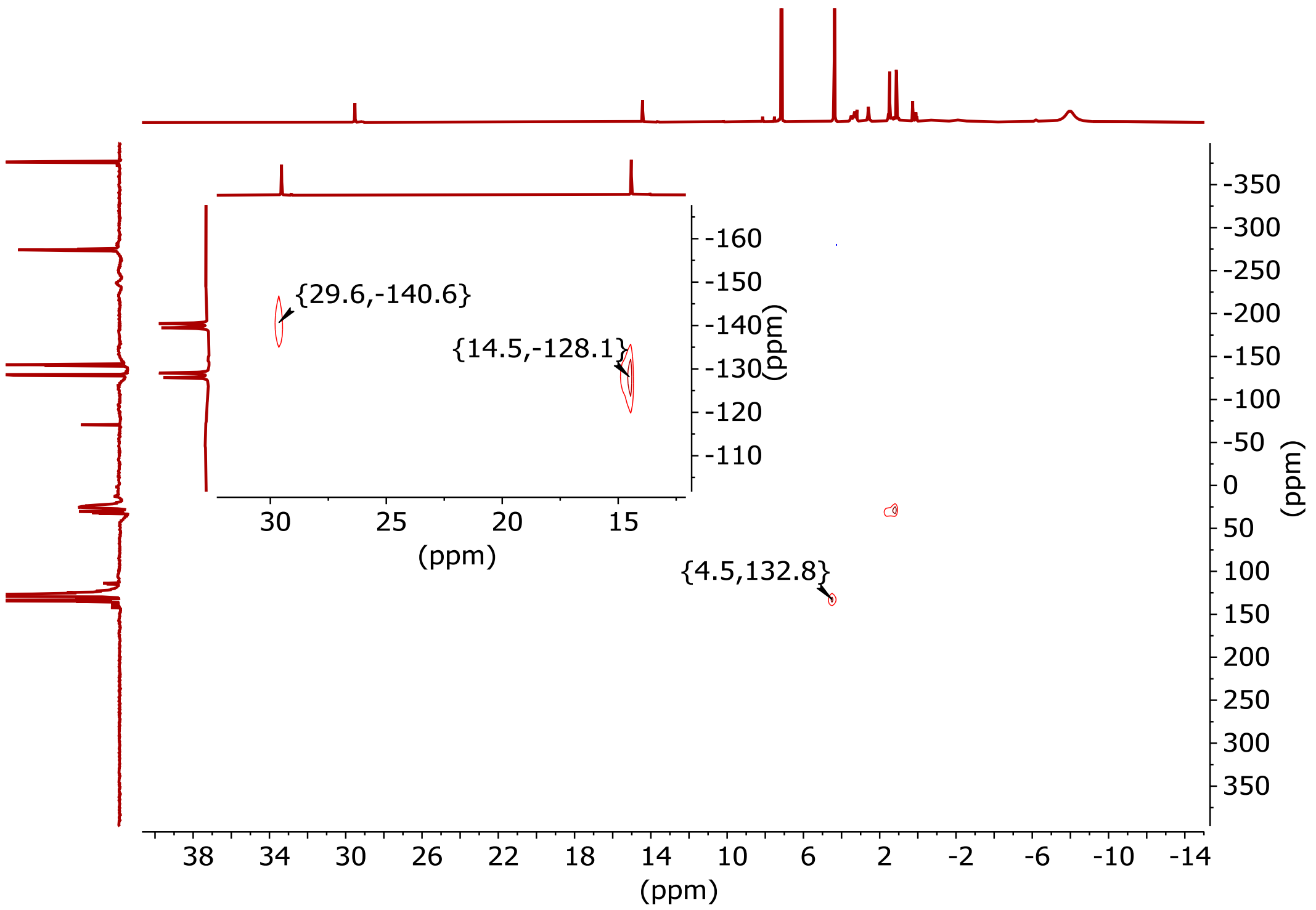

Figure S 9: ^{1}H-^{13}C HSQC spectrum of **4** (C_6D_6, 400/101 MHz, 295 K).

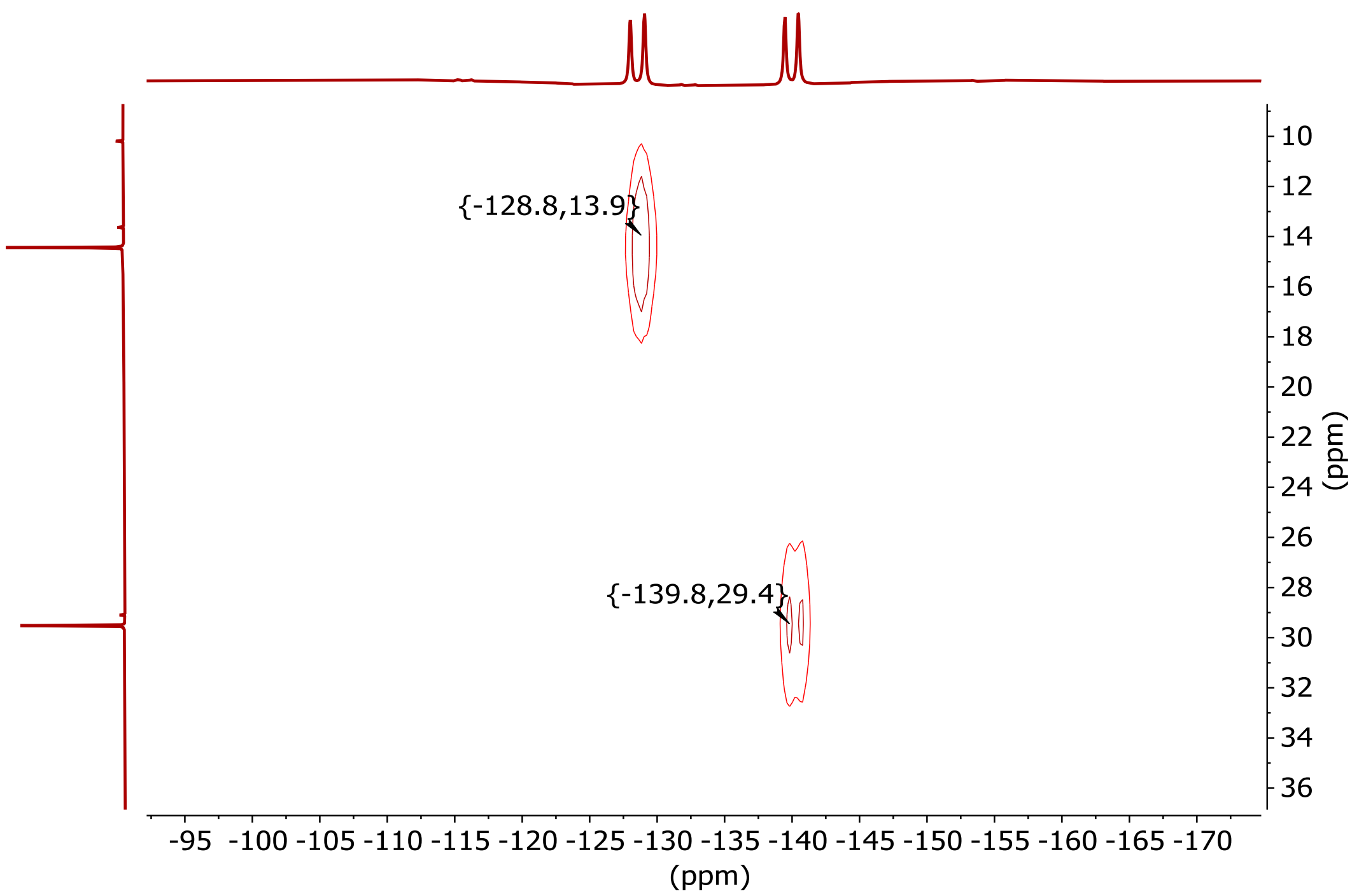

Figure S 10: ^{13}C-^{1}H HETCOR spectrum of **4** (C_6D_6, 151/600 MHz, 295 K).

Compound **5:**

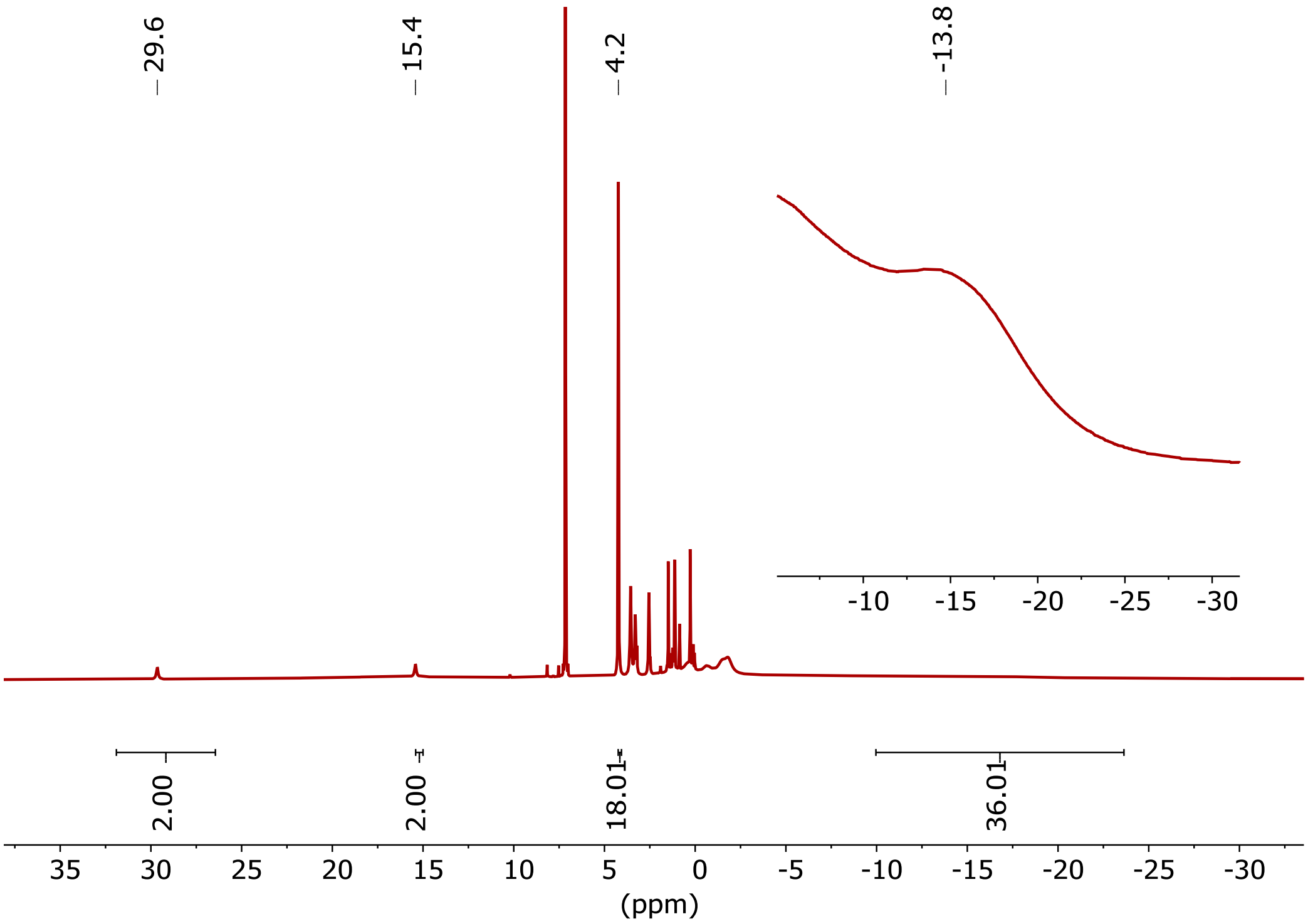

Figure S 11: ^{1}H NMR spectrum of **5** (C_6D_6, 600 MHz, 295 K).

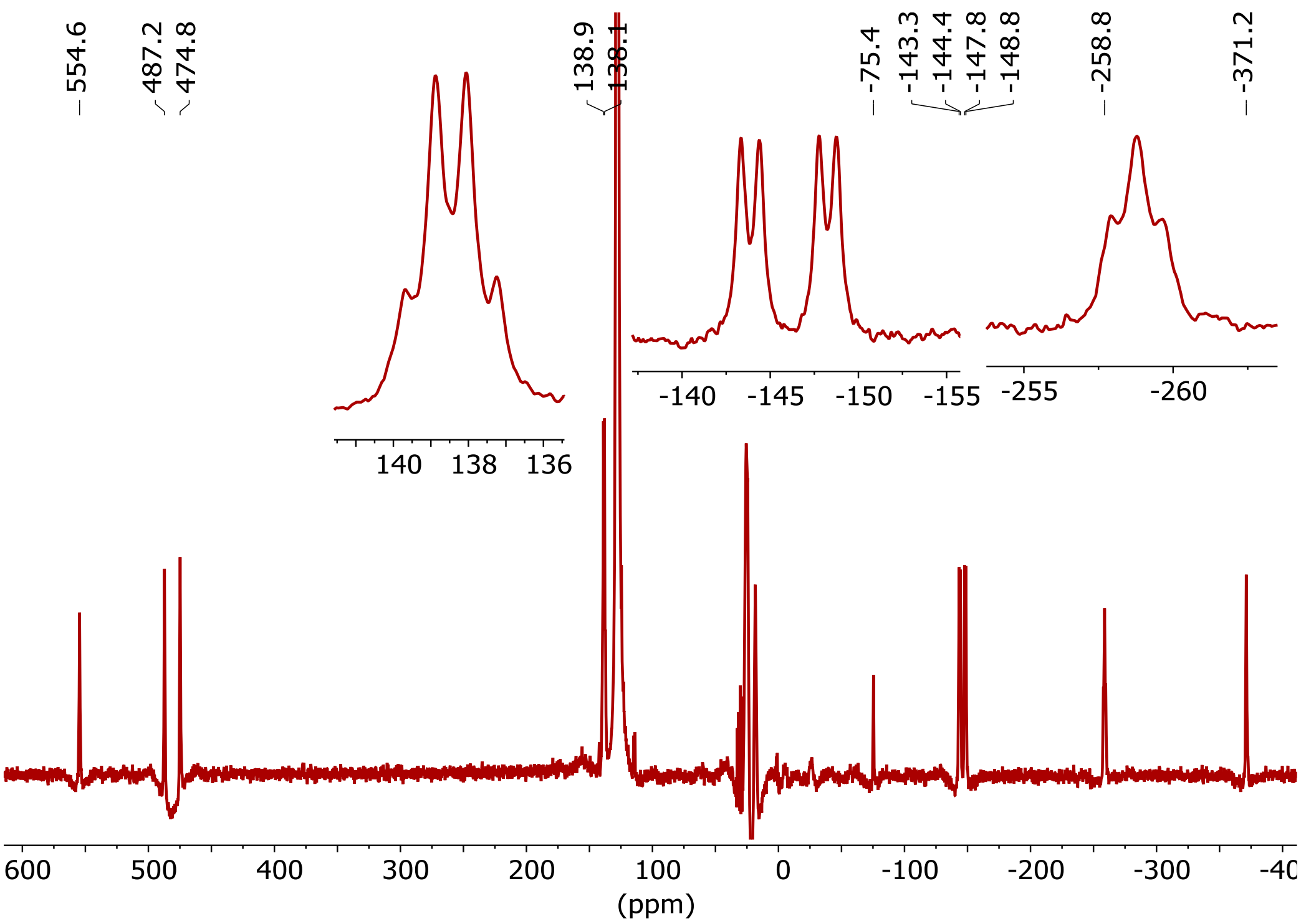

Figure S 12: ^{13}C NMR spectrum of **5** (C_6D_6, 151 MHz, 295 K).

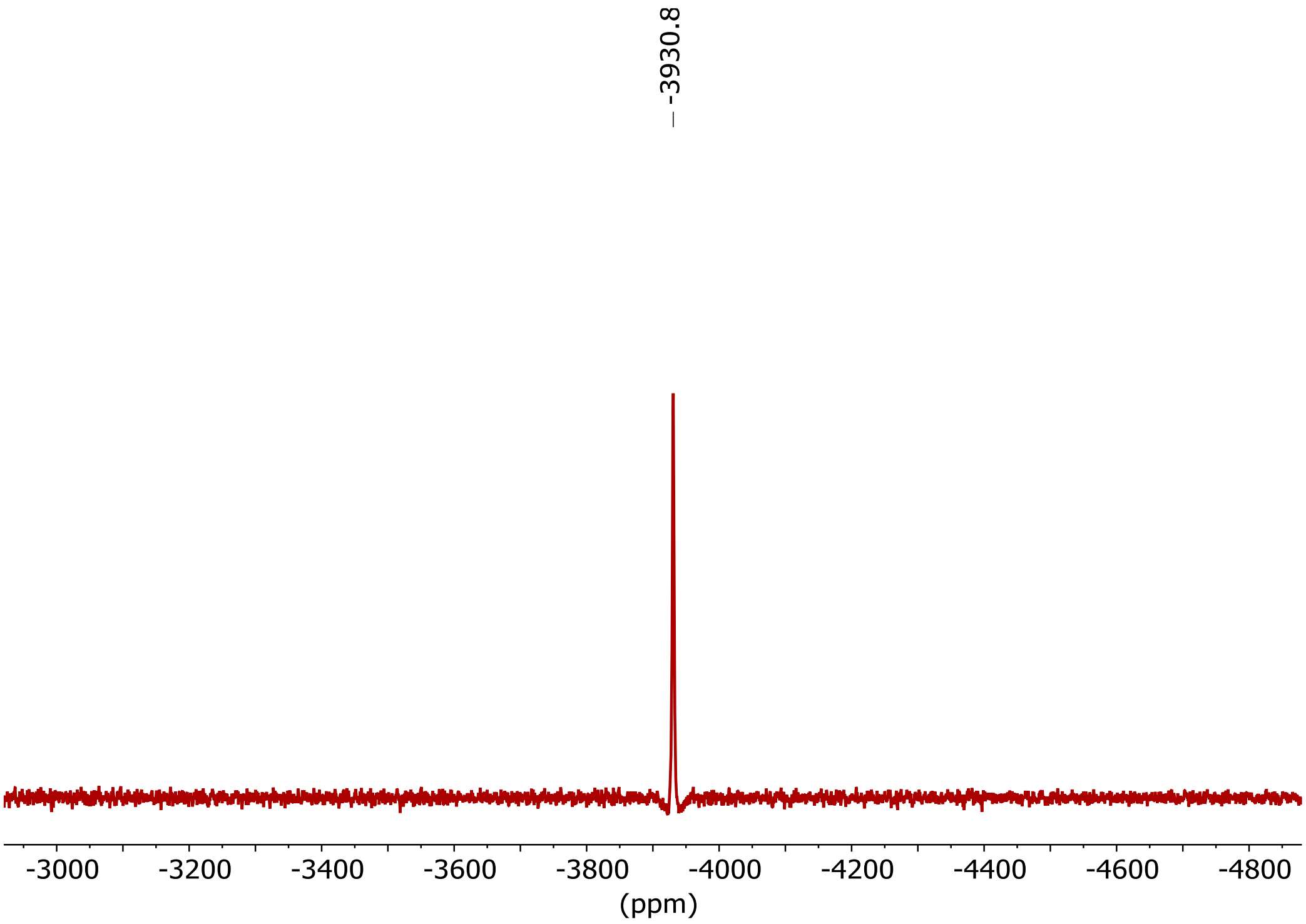

Figure S 13: ^{31}P NMR spectrum of **5** (C_6D_6, 161 MHz, 295 K).

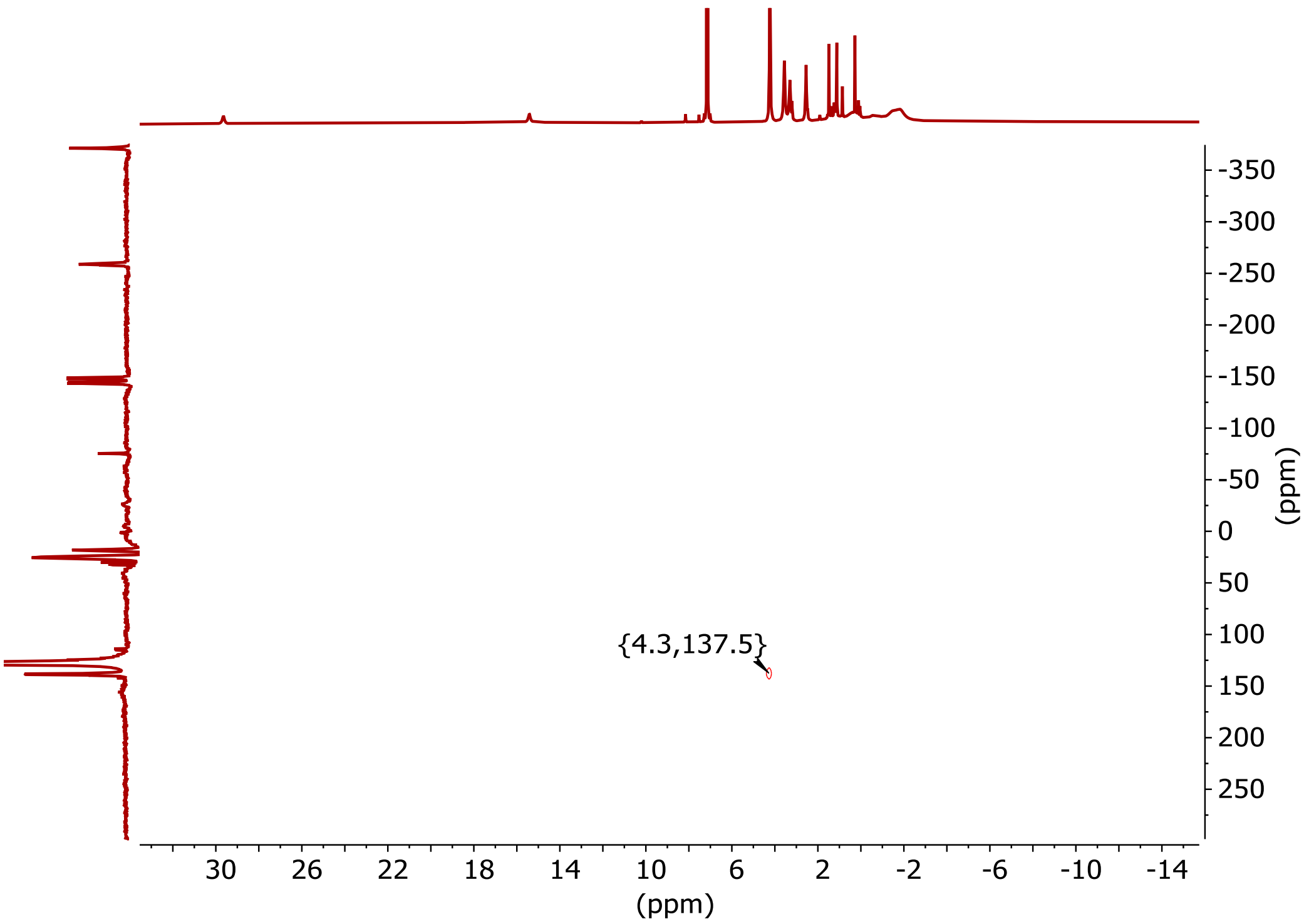

Figure S 14: ^{1}H-^{13}C HSQC spectrum of **5** (C_6D_6, 400/101 MHz, 295 K).

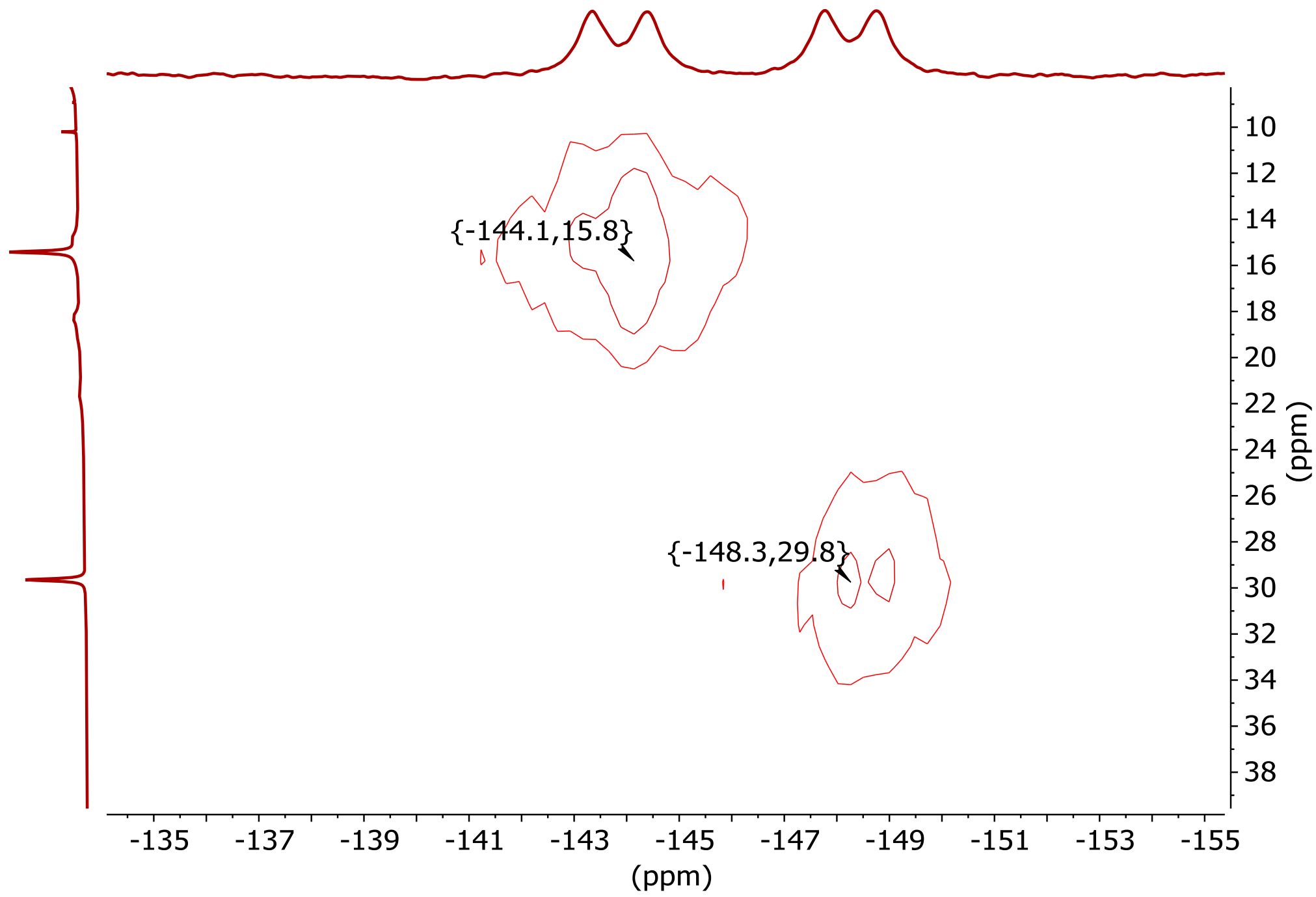

Figure S 15: ^{13}C-^{1}H HETCOR spectrum of **5** (C_6D_6, 151/600 MHz, 295 K).

Compound **3:**

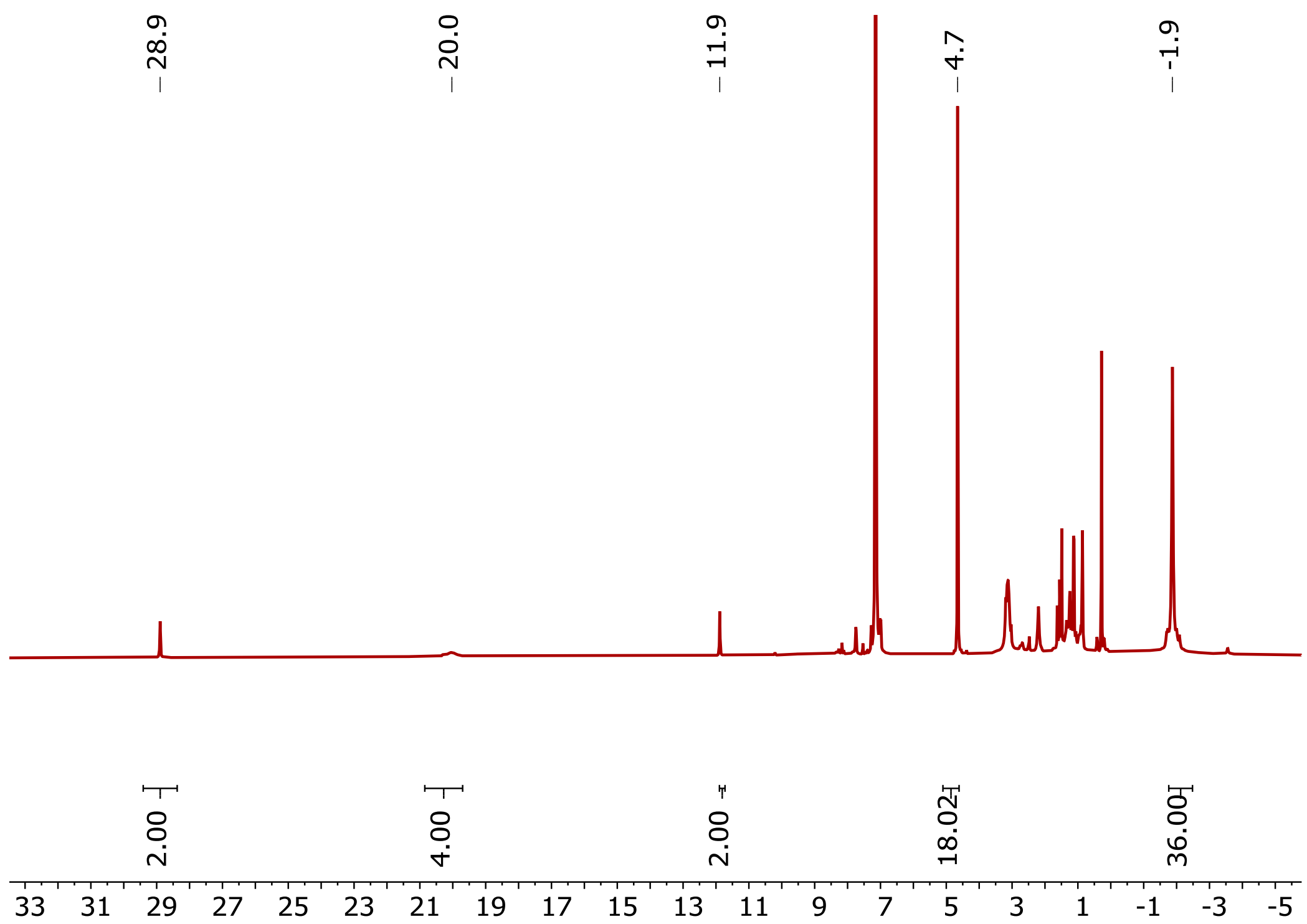

Figure S 16: ^{1}H NMR spectrum of **3** (C_6D_6, 600 MHz, 295 K).

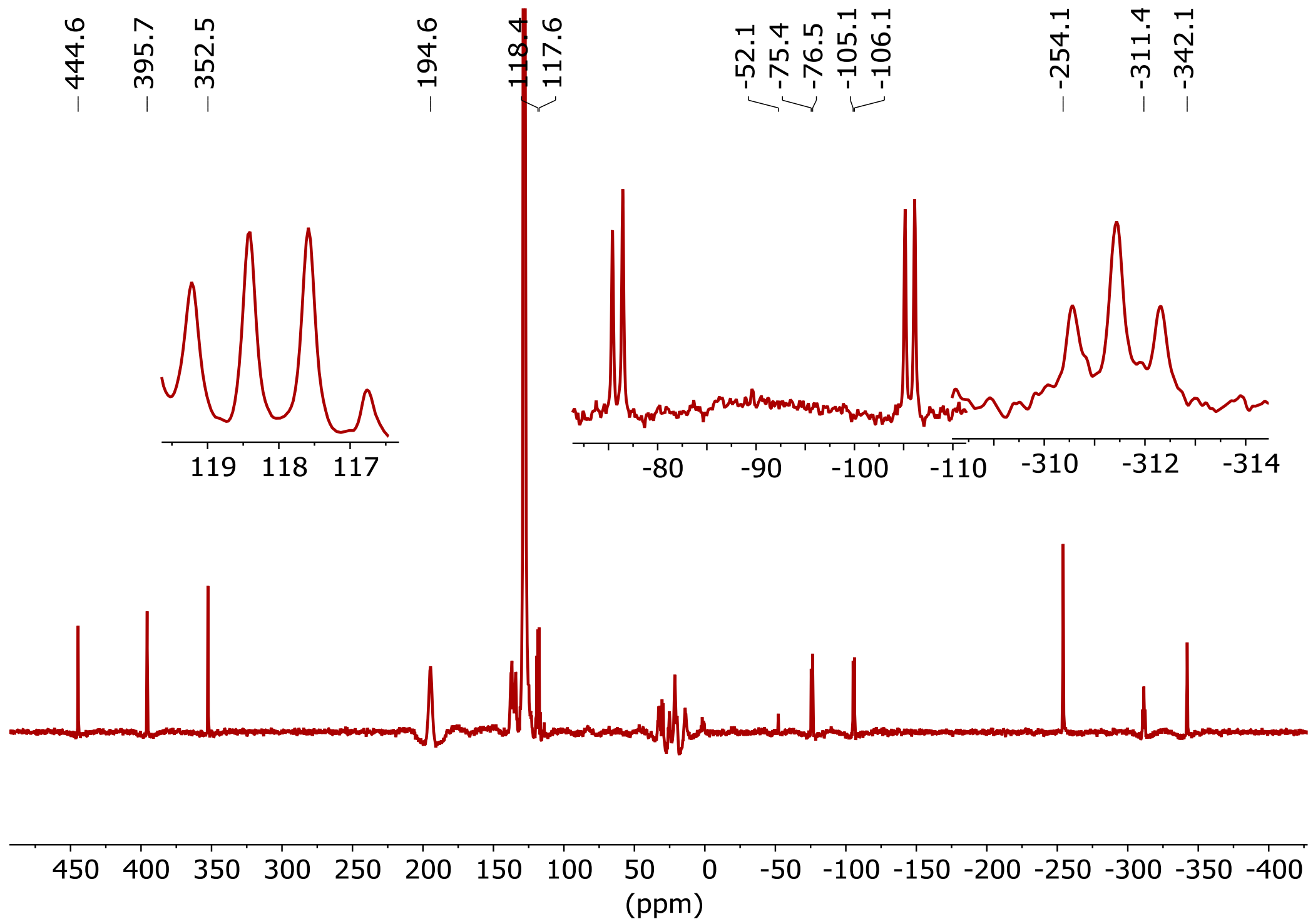

Figure S 17: ^{13}C NMR spectrum of **3** (C_6D_6, 151 MHz, 295 K).

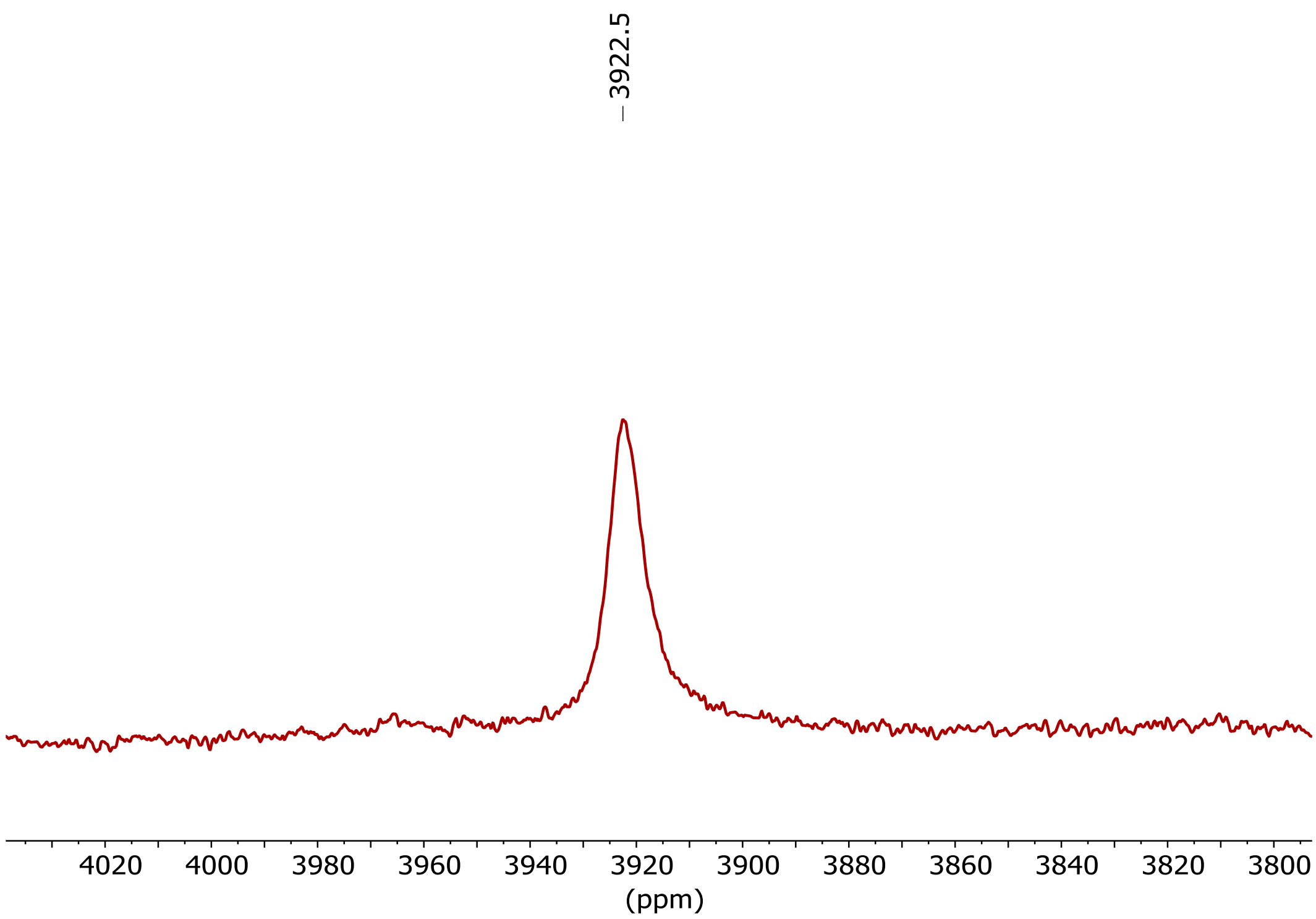

Figure S 18: ^{19}F NMR spectrum of **3** (C_6D_6, 567 MHz, 295 K).

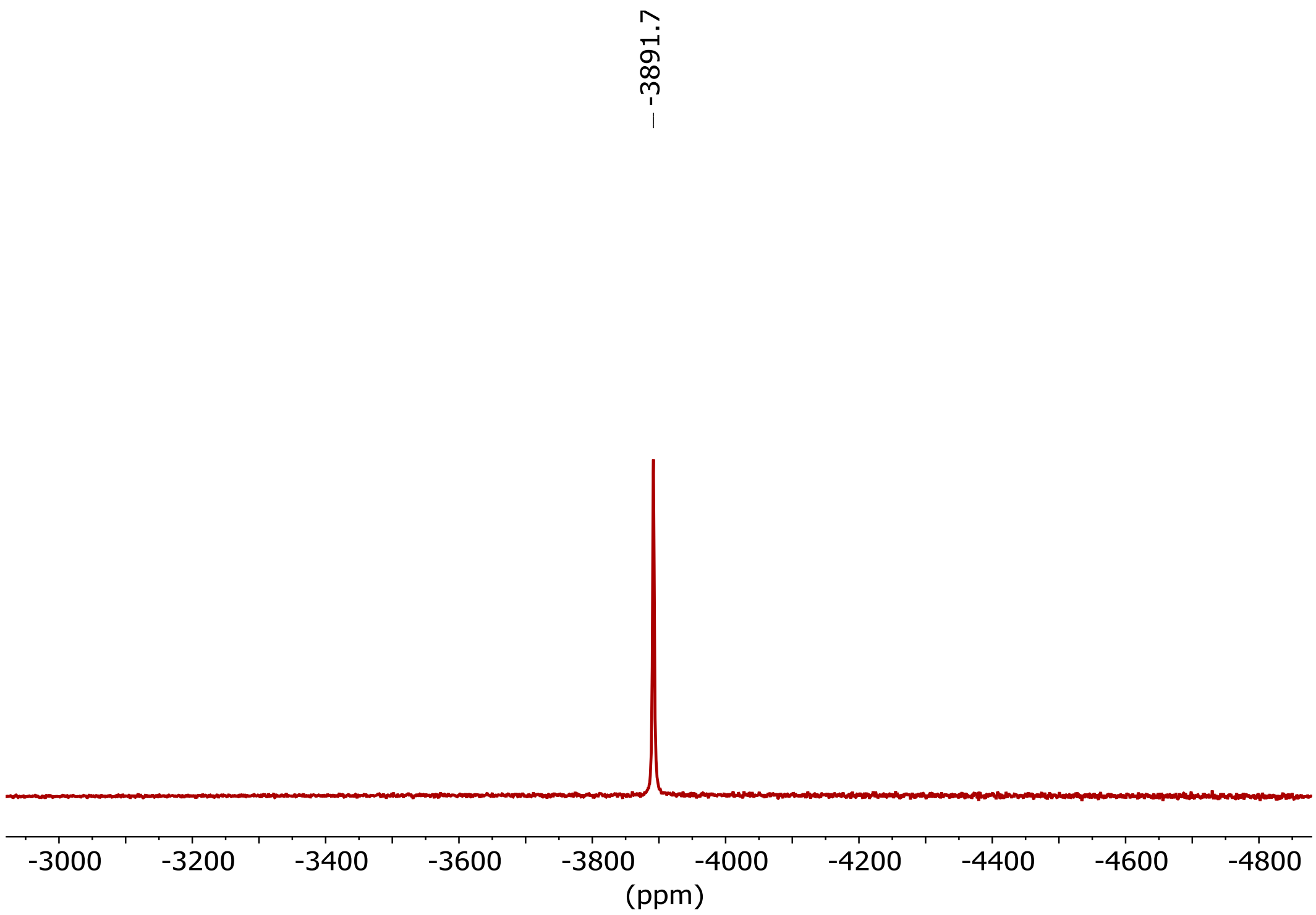

Figure S 19: ^{31}P NMR spectrum of **3** (C_6D_6, 161 MHz, 295 K).

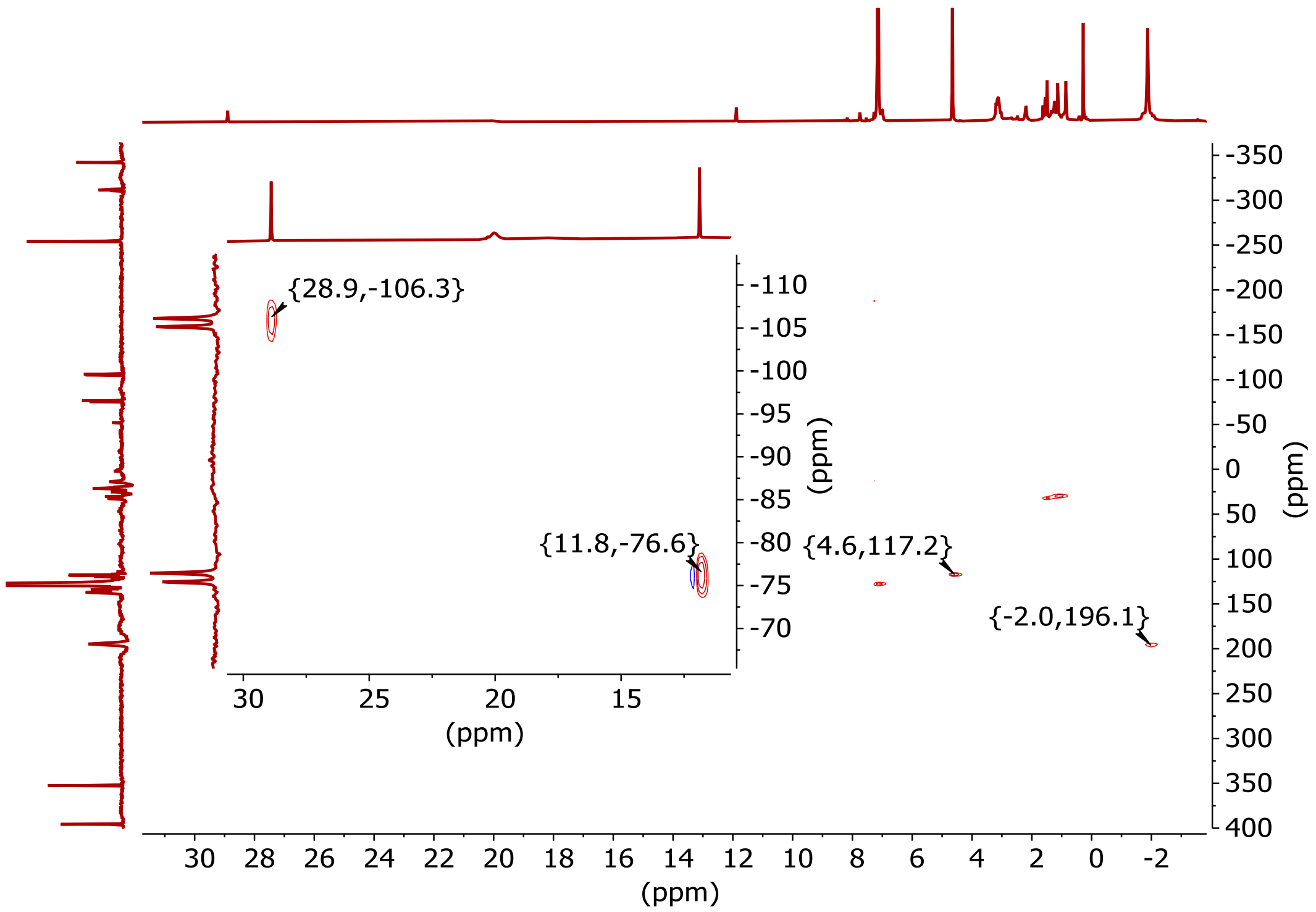

Figure S 20: ^{1}H-^{13}C HSQC spectrum of **3** (C_6D_6, 400/101 MHz, 295 K).

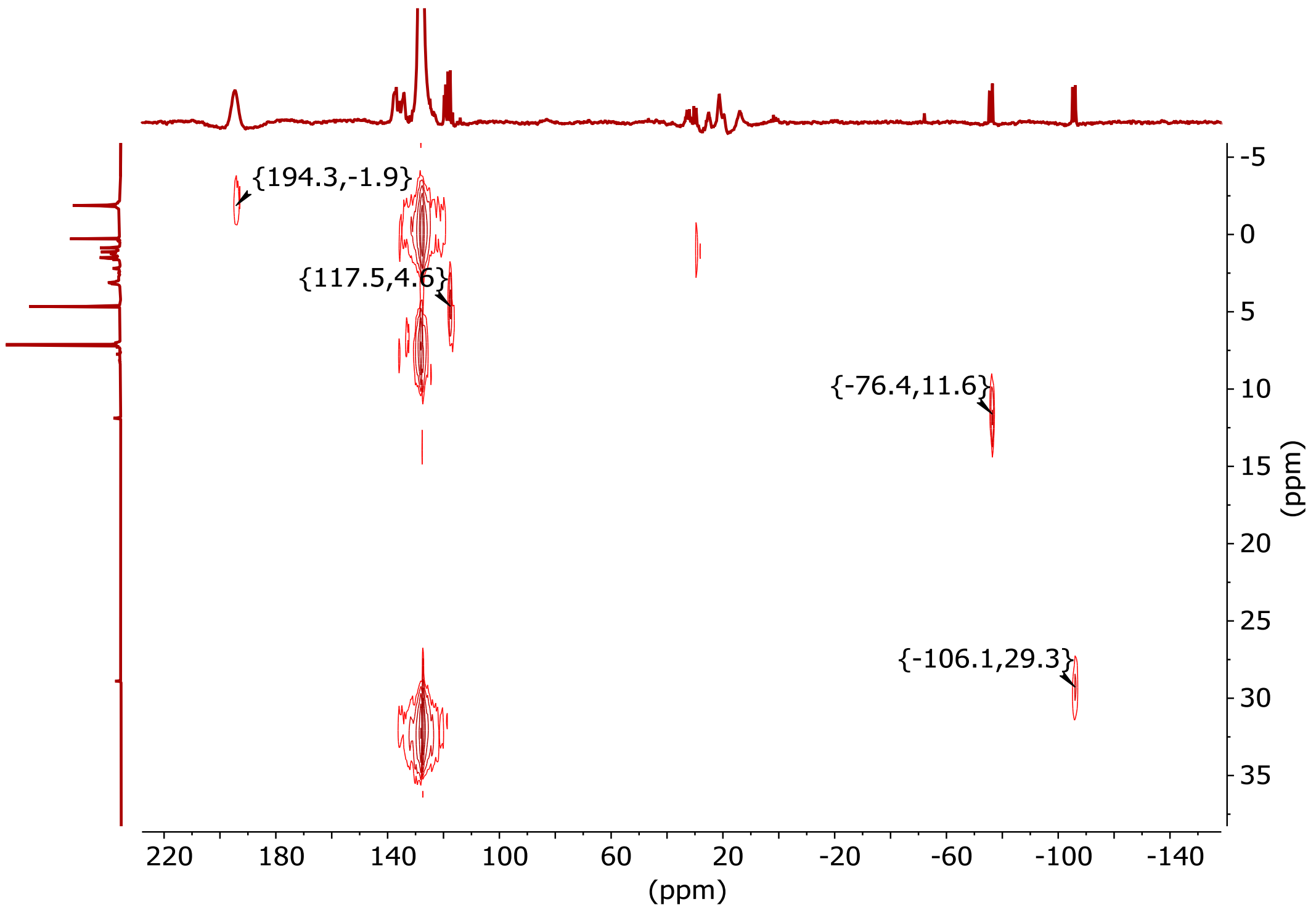

Figure S 21: ^{13}C-^{1}H HETCOR spectrum of **3** (C_6D_6, 151/600 MHz, 295 K).

2.2 General Acquisition procedure

The data acquisition parameters of the paramagnetic ^{1}H, ^{13}C, ^{19}F, ^{31}P and 2D experiments are displayed in Table S1. The NMR spectra were recorded on a Bruker Avance III 600 MHz spectrometer, equipped with a He-cooled Cryo probe, or on a Bruker Avance II 400 MHz spectrometer. The spectra were processed and analyzed with the Bruker NMR software Topspin 3.7.0 or 4.2.0. and linear back prediction was employed during data processing.

Table S 1: Standard acquisition parameters for paramagnetic ^{1}H, ^{13}C. ^{19}F, ^{31}P and HETCOR experiments at Bruker AVIII 600 and HSQC experiments at Bruker AVIII 400.

Parameter	^{1}H	^{13}C	^{19}F	^{31}P	HETCOR	HSQC
Pulse program (Bruker)	zg	zg	zg	zg	hxcoqf	hsqcph
90° pulse width *p1* [µs]	9.50	10.0	15.0	11.3	10.0 (^{13}C)	8.55
Pulse width used [°]/[µs]	9/0.95	60/6.66	30/5.00	30/3.77	90/10.0 (^{13}C)	90/8.55
Sweep width *F1* [ppm]	890	1988	245	1968	994	121
Sweep width *F2* [ppm]					238	800
Acquisition data points *td*	262k	66k	131k	66k	8k × 64	1k × 64
Acquisition time *aq* [s]	0.245	0.109	0.114	0.069	0.027/0.0004	0.011/0.0008
Relaxation delay *d1* [s]	0.05	0.05	0.1	0.01	0.05	0.2
Preacquisition delay *de* [µs]	15.0	18.0	18.0	18.0	18.0	6.50
Number of scans *ns*	2k	100k	32	10k	0.5k	32

3. UV-VIS Spectra

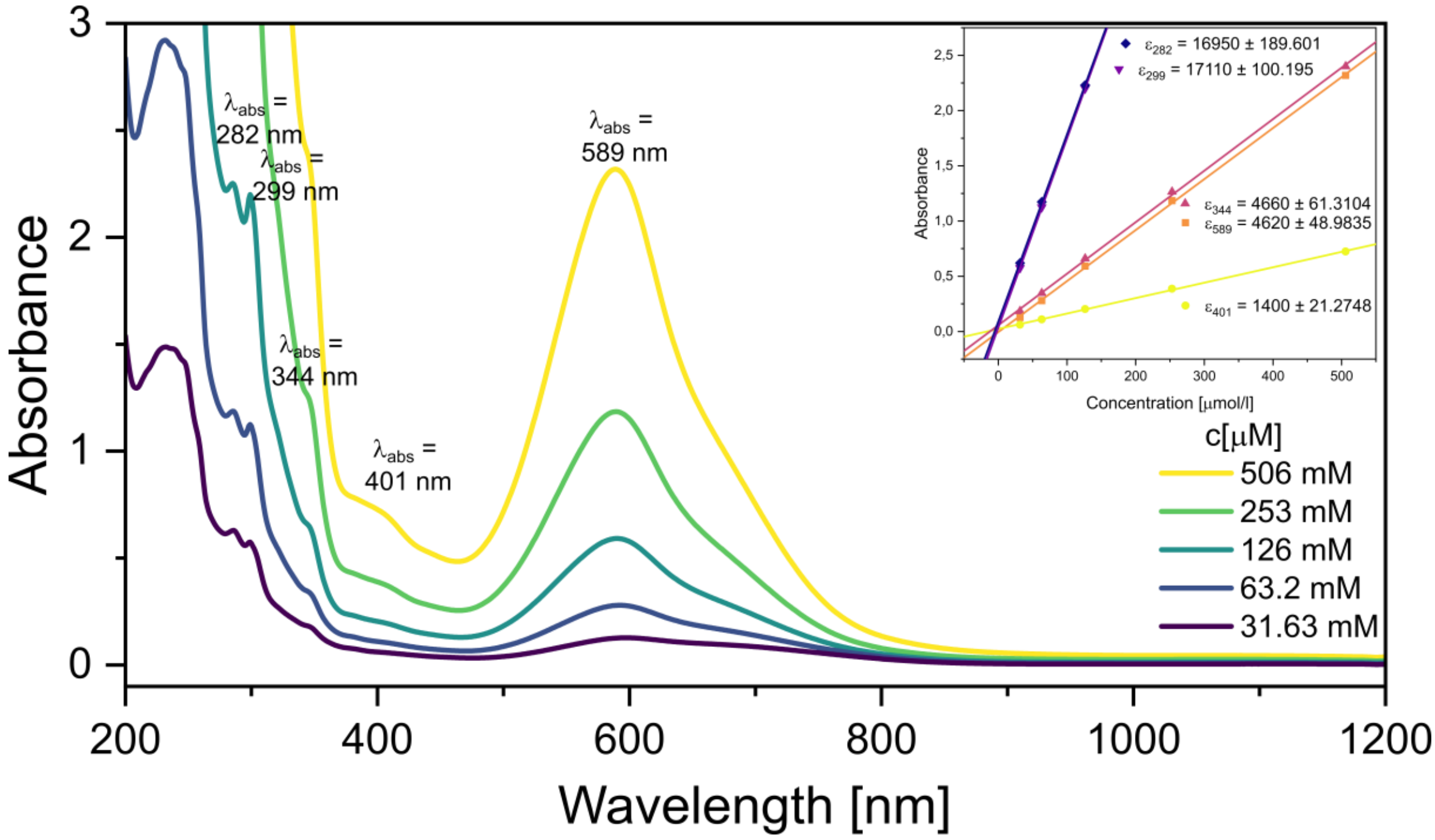

Figure S 22: Concentration dependent UV-VIS spectra of **2** (*n*pentane, 295 K).

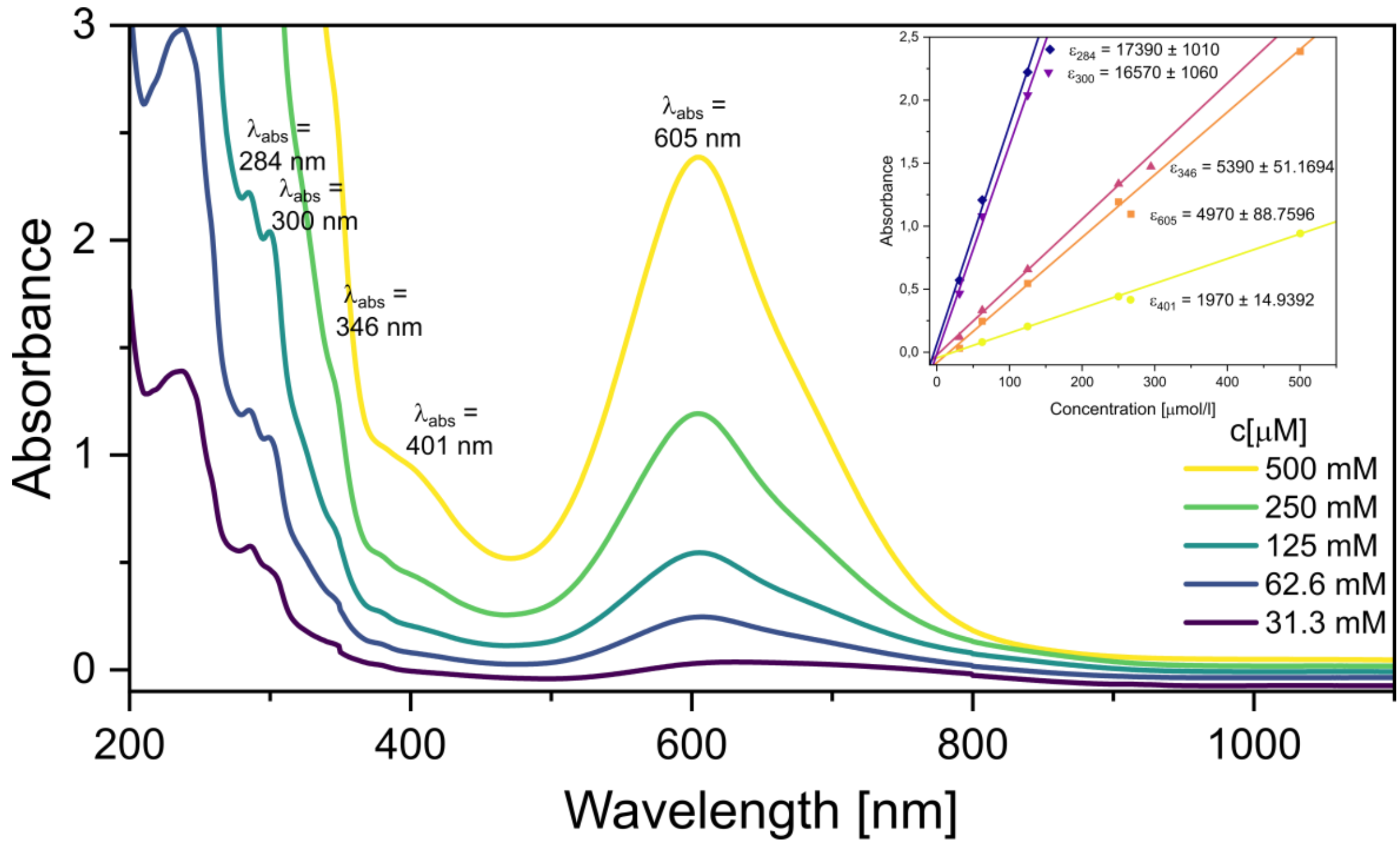

Figure S 23: Concentration dependent UV-VIS spectra of **4** (*n*pentane, 295 K).

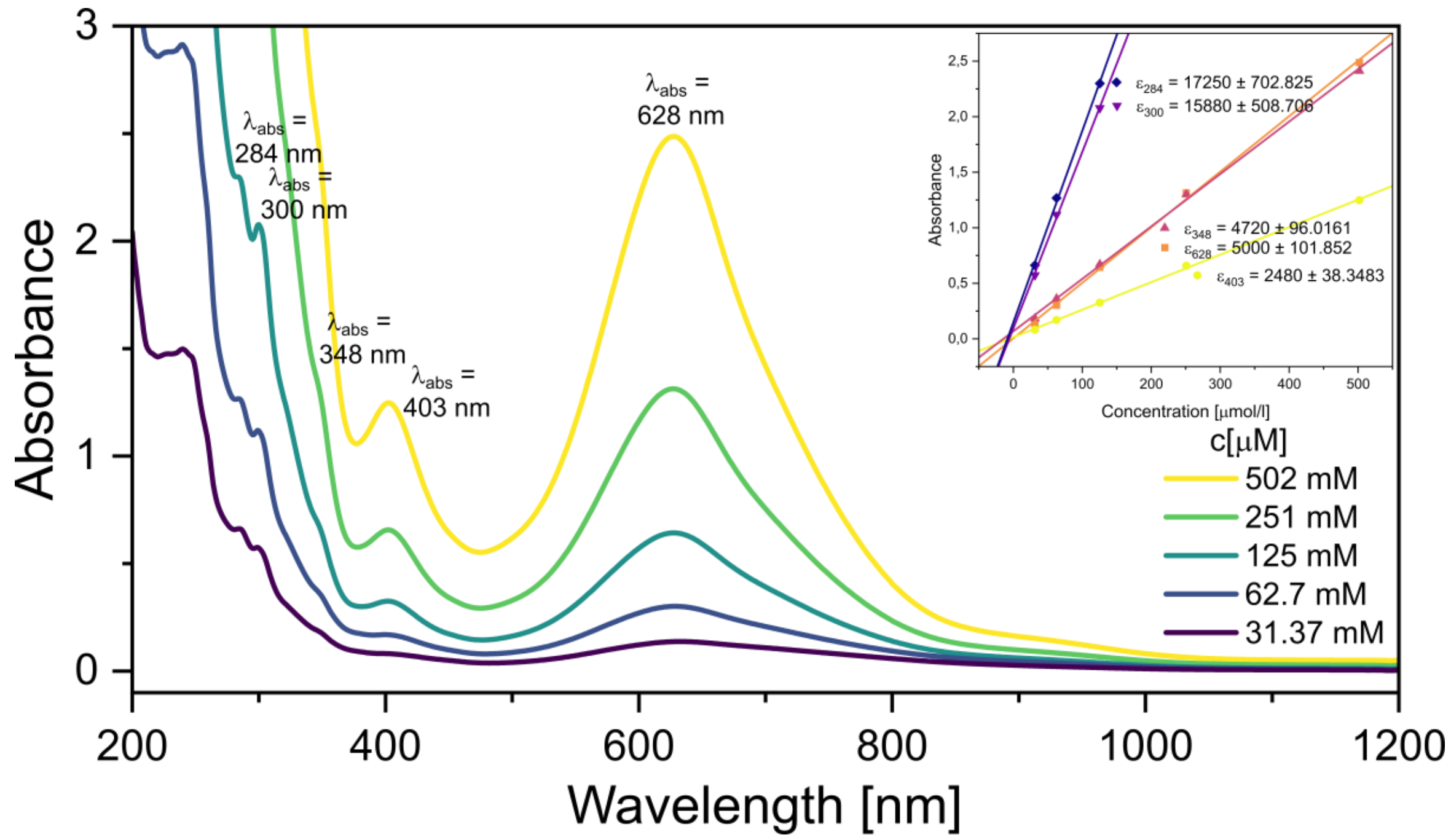

Figure S 24: Concentration dependent UV-VIS spectra of **5** (^{n}pentane, 295 K).

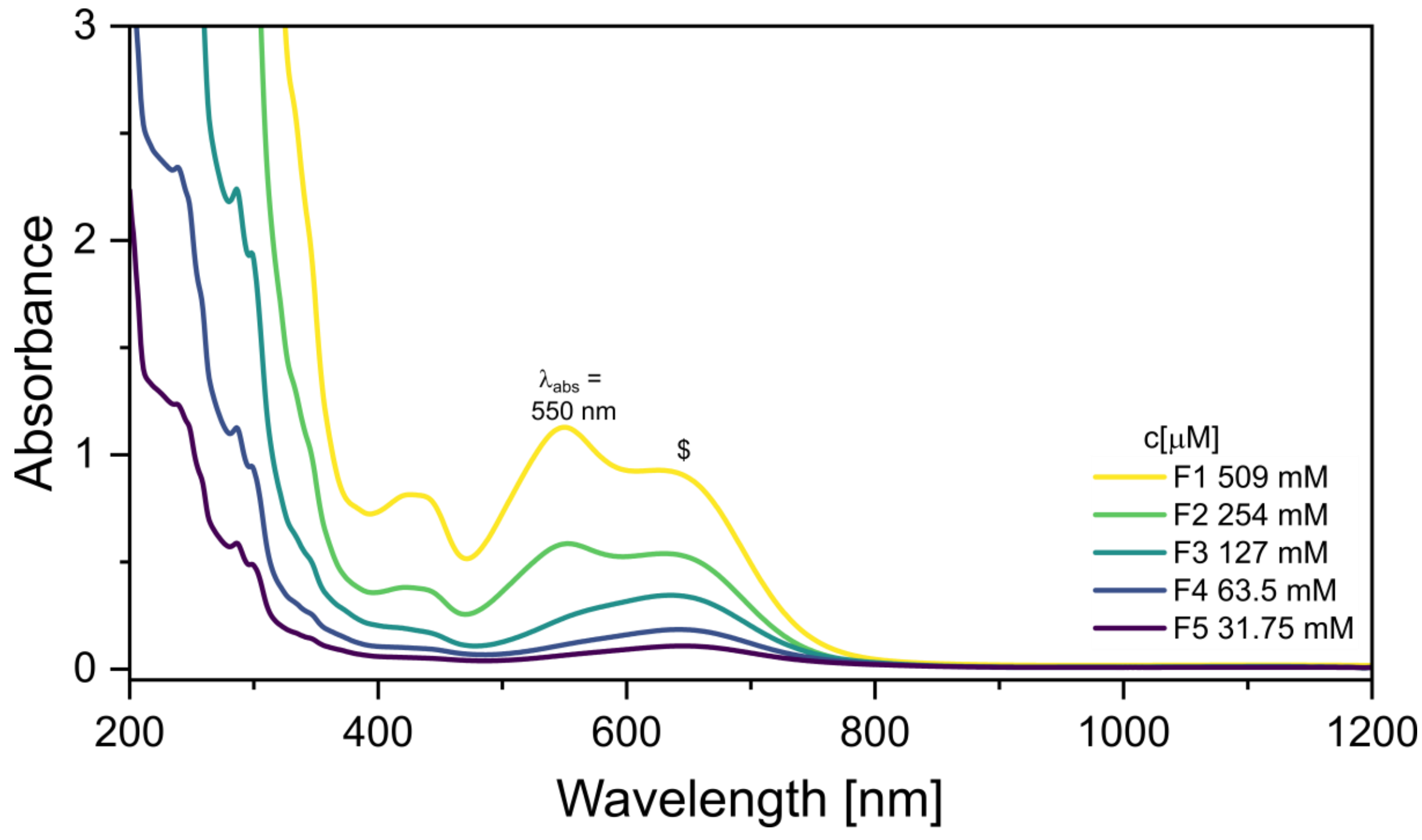

Figure S 25: Concentration dependent UV-VIS spectra of **3** (^{n}pentane, 295 K). Absorption band association with MLCT PNPRuF transition at 550 nnm. $ denotes band associated with decomposition product.

Because of the apparent decomposition of **3** in solution no molar extinction coefficients could be determined. The transition of the complex **3** associated with MLCT, which changes with the halide through the series of complexes, could still be identified at 550 nm. Another band associated with the decomposition product at 626 nm is visible as well. Compared to the band at 550 nm, which decreases at a faster rate than expected from dilution, this band decreases at a slower rate.

4. SQUID Magnetometry

The magnetic susceptibility data was fitted using the Matlab toolbox EasySpin[4] employing the following Spin Hamiltonian:

$$\hat{H} = \mu_B B \boldsymbol{g} \hat{S} + D\left(\hat{S}_Z^2 - \frac{1}{3}S(S+1)\right) + E(\hat{S}_X^2 - \hat{S}_Y^2) \quad \text{(S1)}$$

With the first term denoting the electron-spin Zeeman term involving the $\boldsymbol{g}$-tensor and the second term as axial and rhombic 2nd-order ZFS with parameters D and E, respectively. $g_Z$is assumed to be colinear with D_Z. χ_{TIP} (added as a scalar to χ_{ZZ})and a paramagnetic ($S = 1/2$ impurity; as offset baseline) were also considered during the fit.

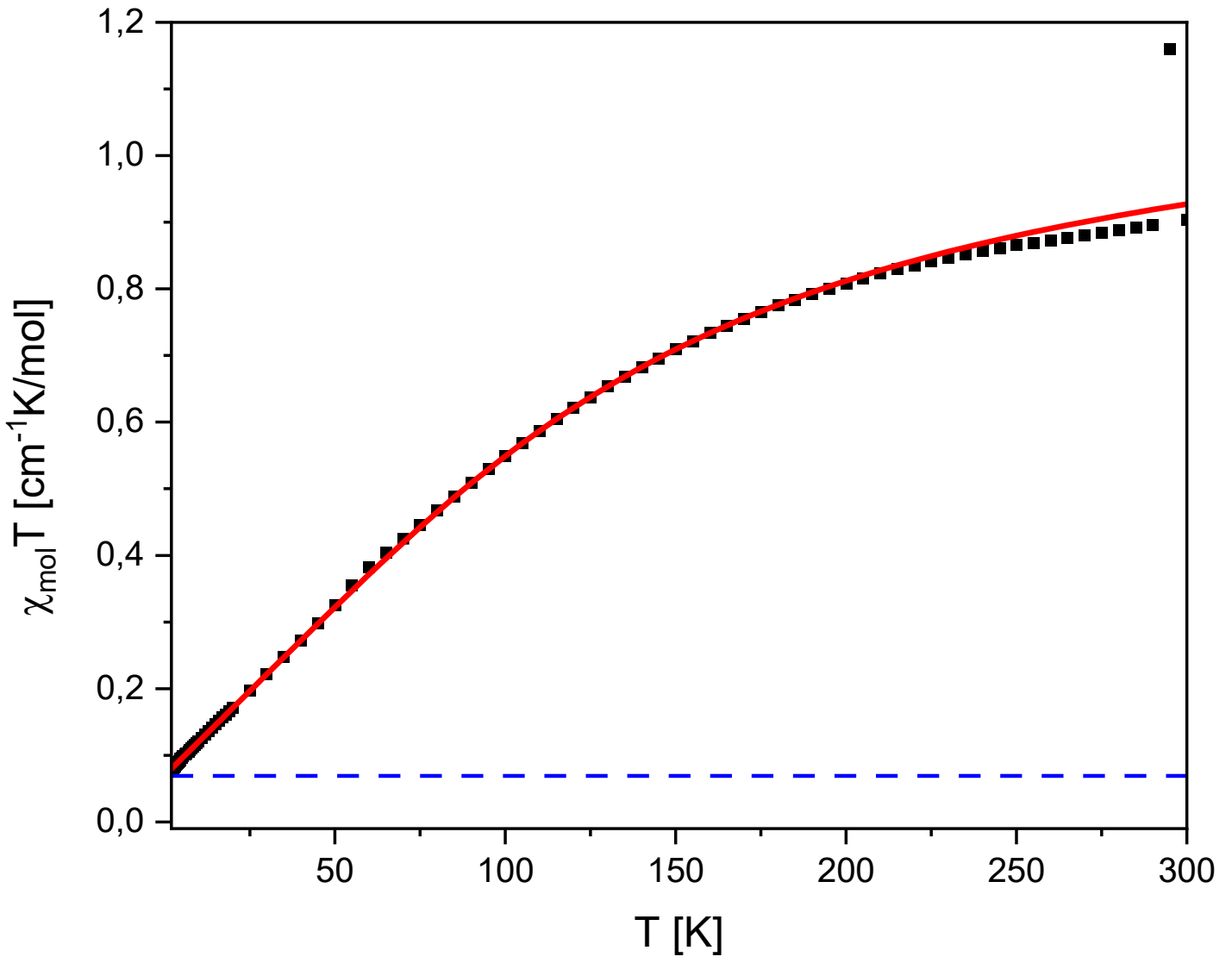

Figure S 26: Magnetic characterisation of **2** with a χT versus T plot obtained at 1 T (black squares: experimental data; red line: simulated spectrum; dashed blue line: 8% paramagnetic impurity) Fitting parameters: D = 229 cm^{-1}, $|E/D|$ = 0.31, g = [1.90, 1.60, 2.32], χ_{TIP}= 1.95×10^{-9} m^{3}mol^{-1}.

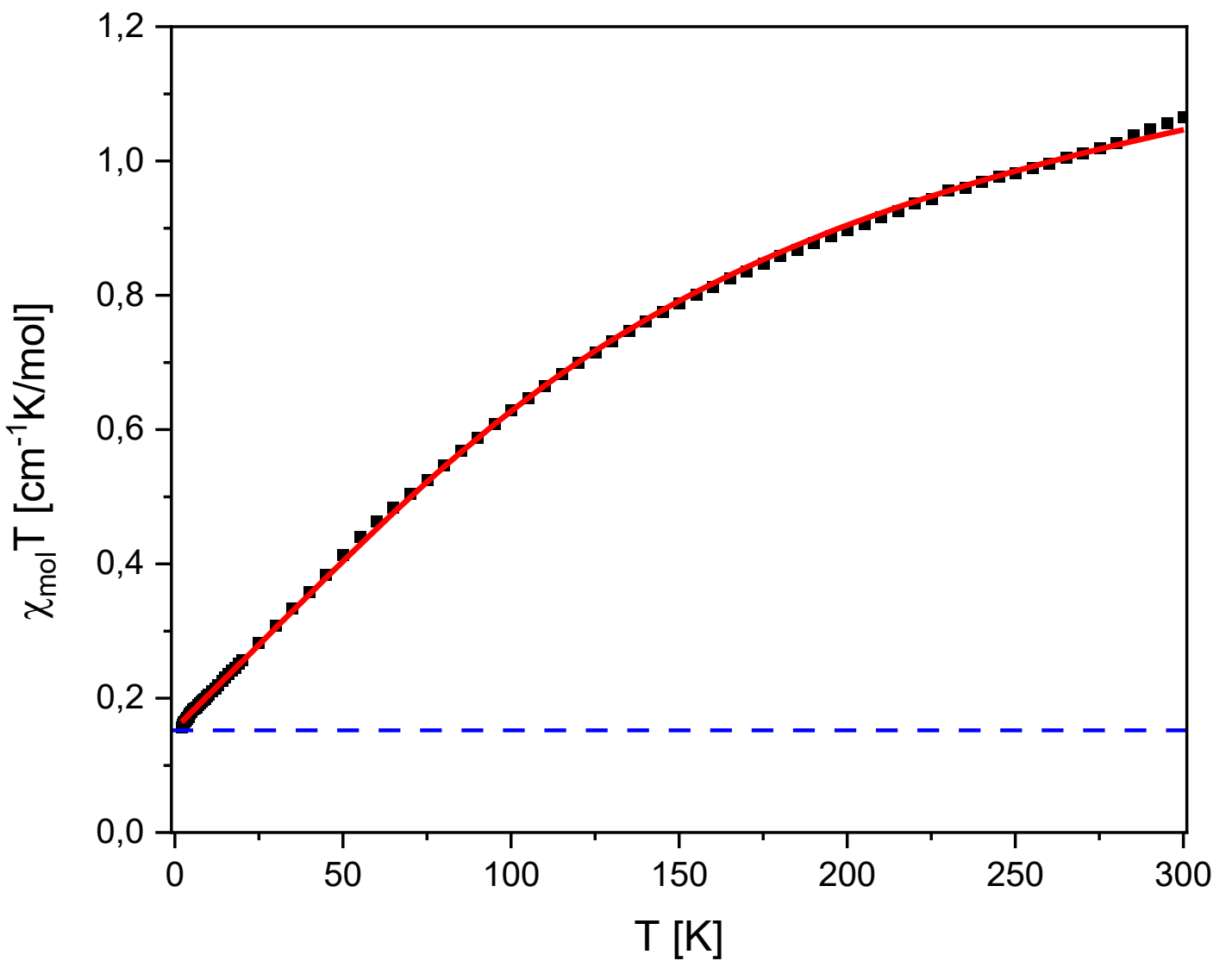

Figure S 27: Magnetic characterisation of **4** with a χT versus T plot obtained at 1 T (black squares: experimental data; red line: simulated spectrum; dashed blue line: 15% paramagnetic impurity) Fitting parameters: D = 238 cm^{-1}, $|E/D|$ = 0.33, g = [1.84, 1.56, 2.20], χ_{TIP}= 6.11×10^{-9} m^{-1}mol^{-1}.

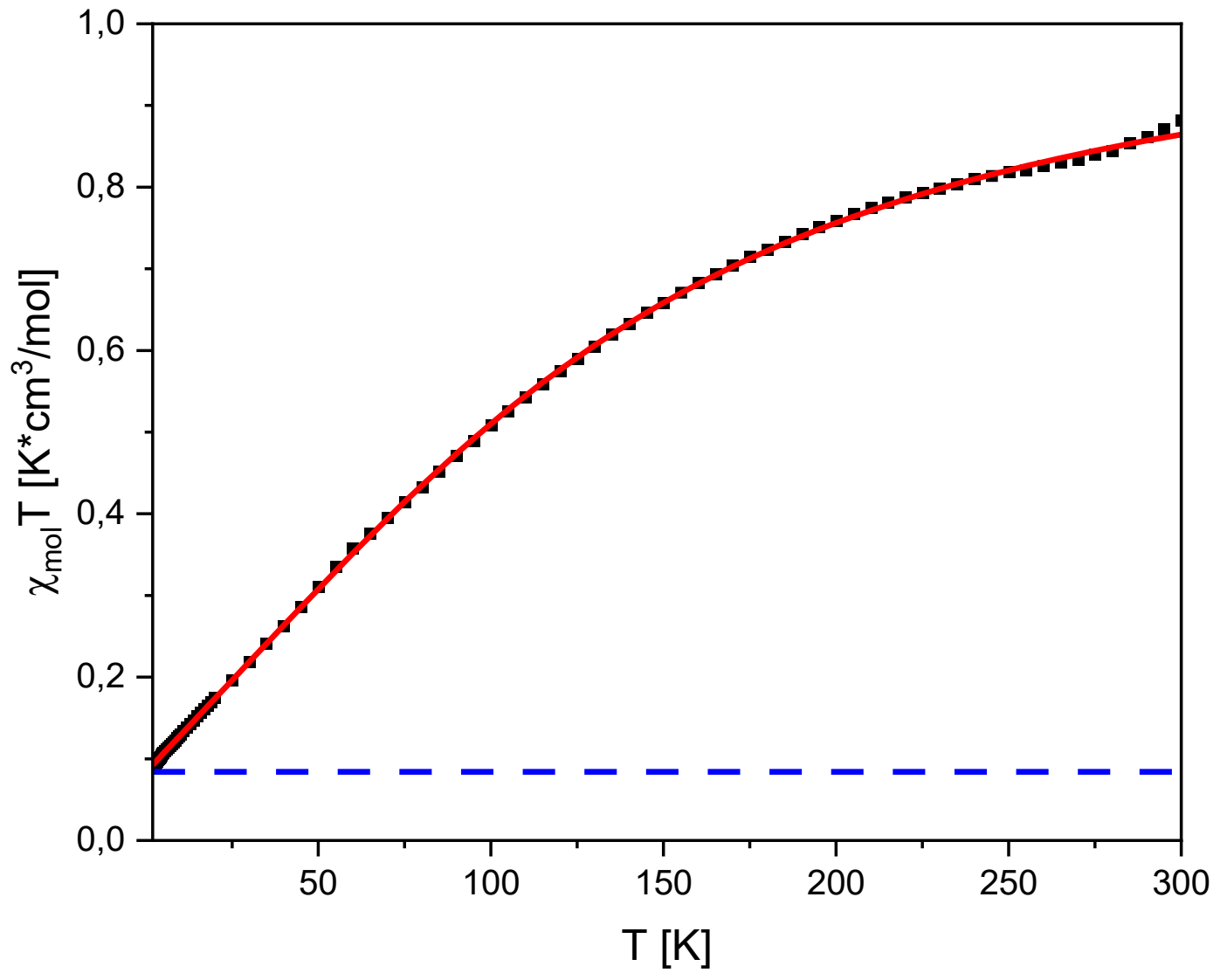

Figure S 28: Magnetic characterisation of **5** with a χT versus T plot obtained at 1 T (black squares: experimental data; red line: simulated spectrum; dashed blue line: 10% paramagnetic impurity) Fitting parameters: D = 257 cm^{-1}, $|E/D|$ = 0.33, g = [1.95, 1.58, 2.20], χ_{TIP}= 4.65×10^{-10} $m^{-1}mol^{-1}$.

5. Variable Temperature NMR experiments

5.1 VT-NMR ^{1}H, ^{13}C, ^{19}F and ^{31}P spectra of the halide complexes

Compound **2:**

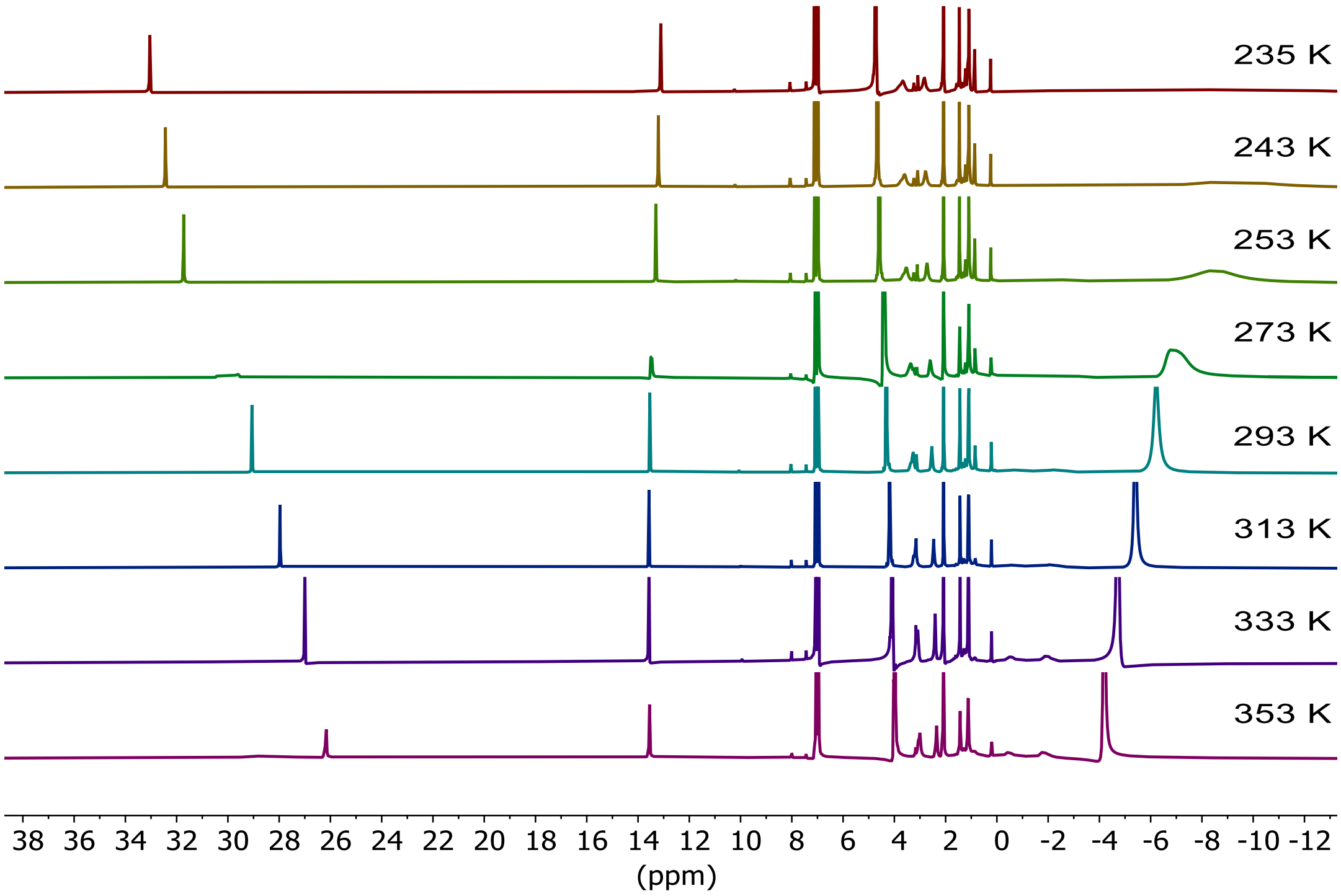

Figure S 29: ^{1}H NMR spectrum of **2** (tol-d$_6$, 600 MHz, 295 K) at different temperatures (235-353 K).

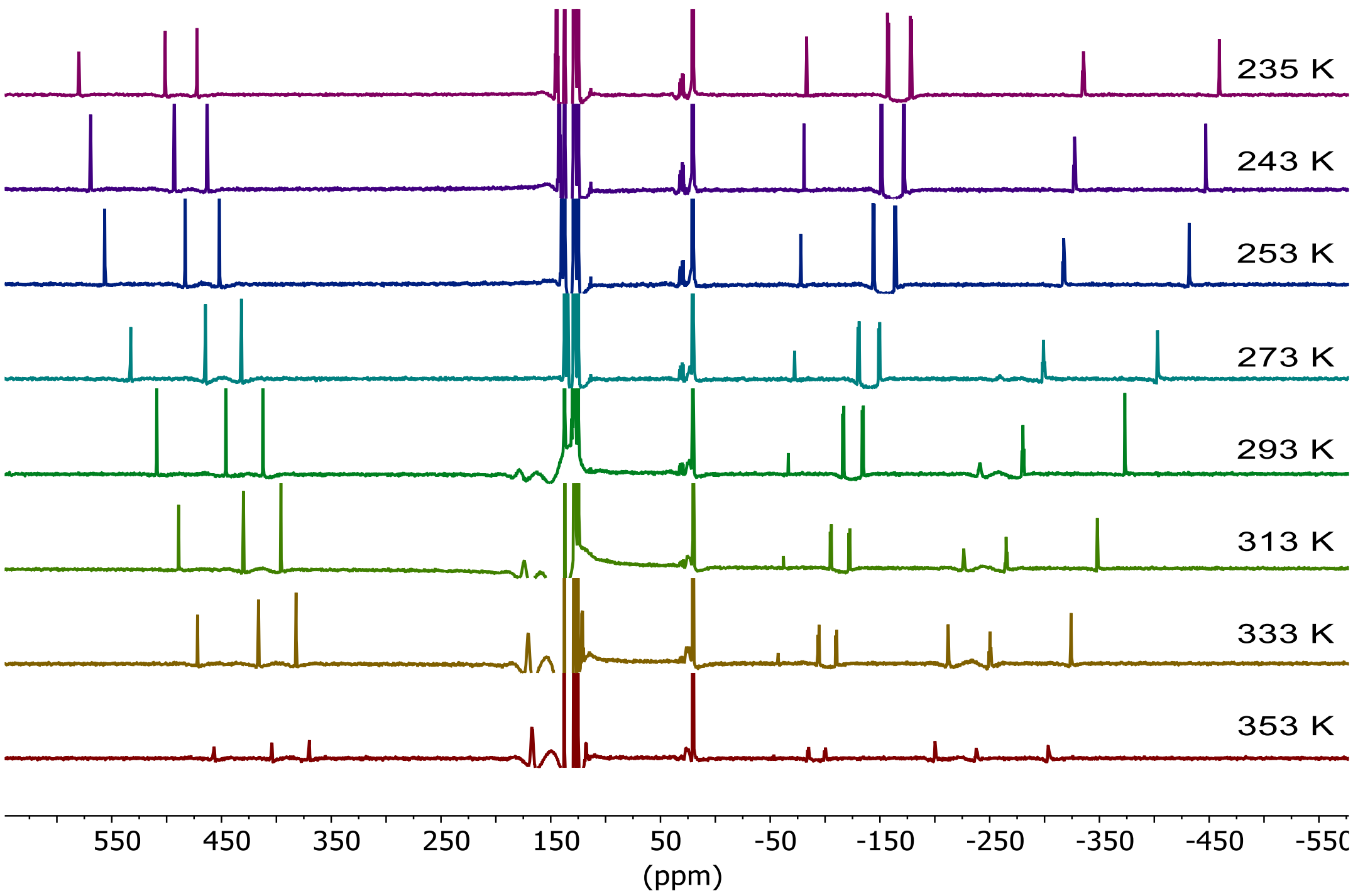

Figure S 30: ^{13}C NMR spectrum of **2** (tol-d$_6$, 151 MHz, 295 K) at different temperatures (235-353 K).

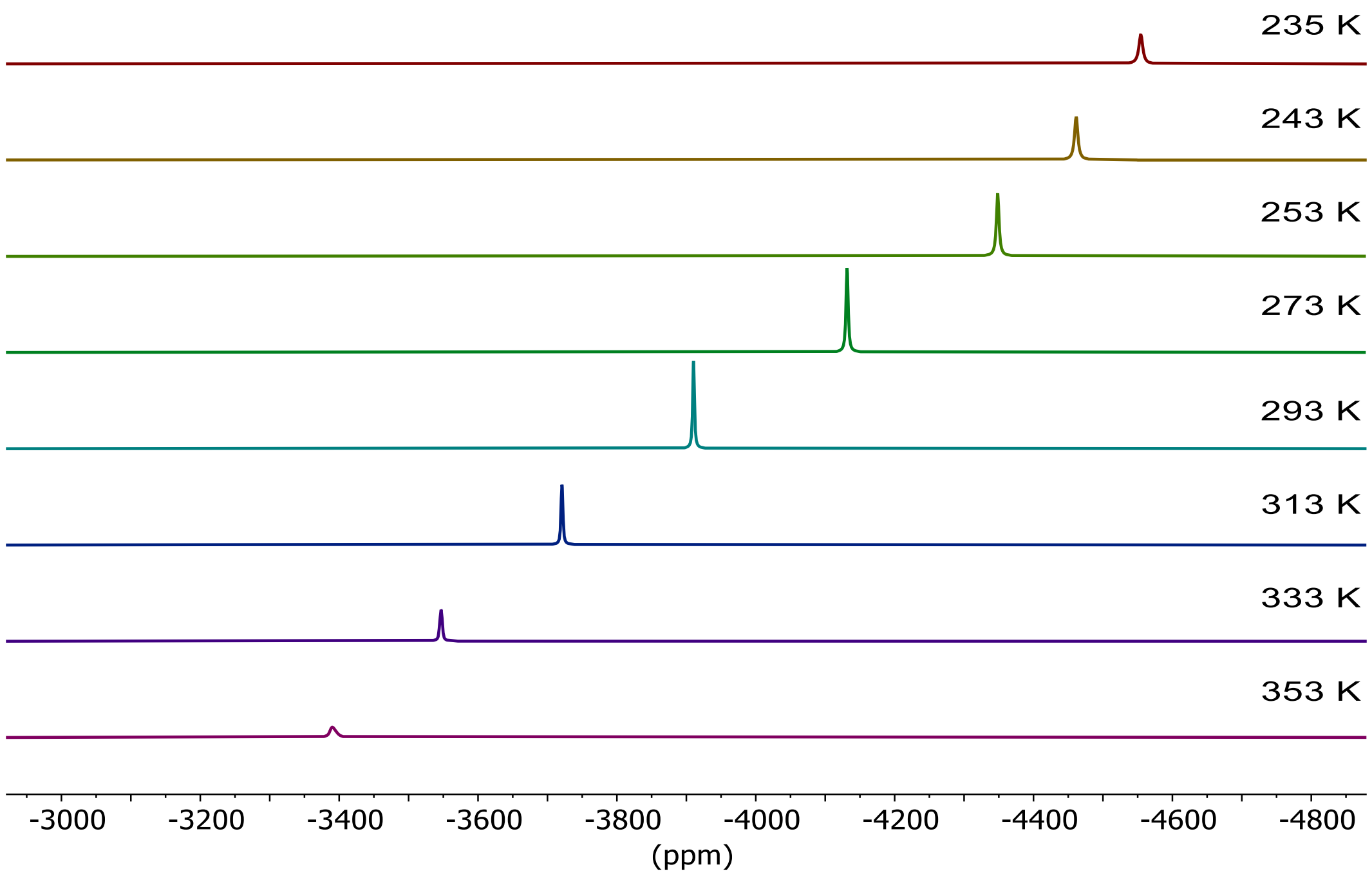

Figure S 31: ^{31}P NMR spectrum of **2** (tol-d$_6$, 242 MHz, 295 K) at different temperatures (235-353 K).

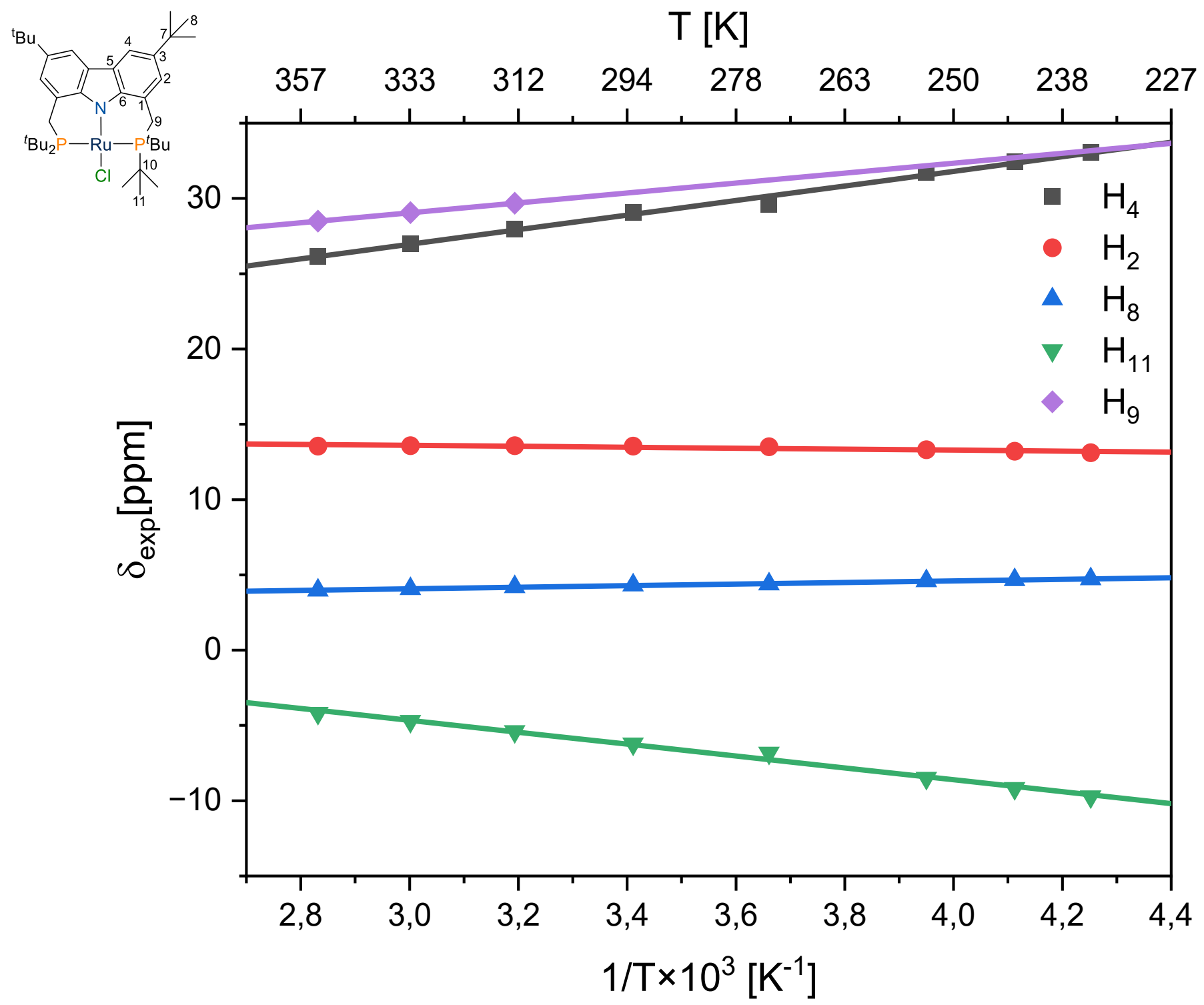

Figure S 32: Curie plot of the ^{1}H resonances of **2** (dots) and linear regression (lines).

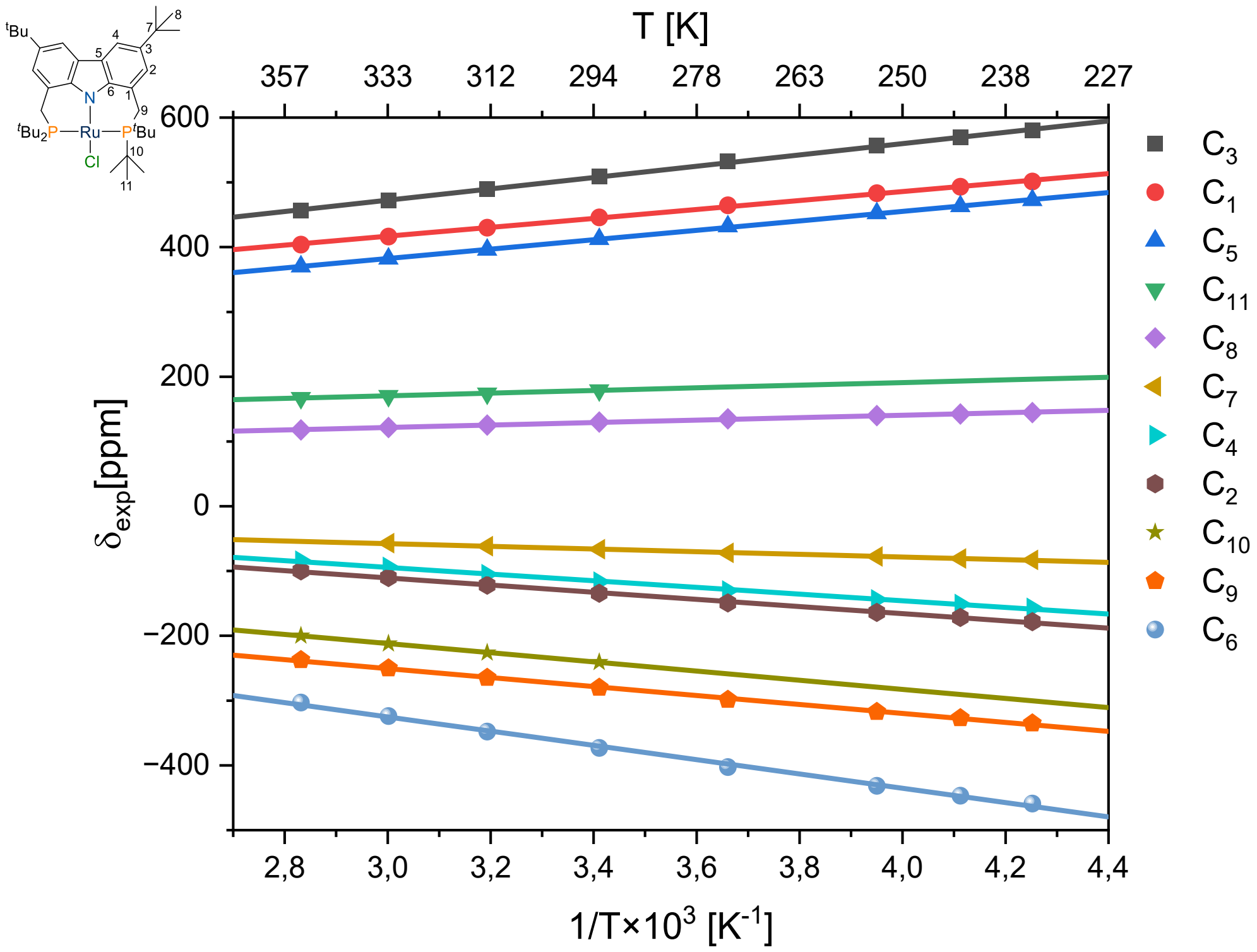

Figure S 33: Curie plot of the ^{13}C resonances of **2** (dots) and linear regression (lines).

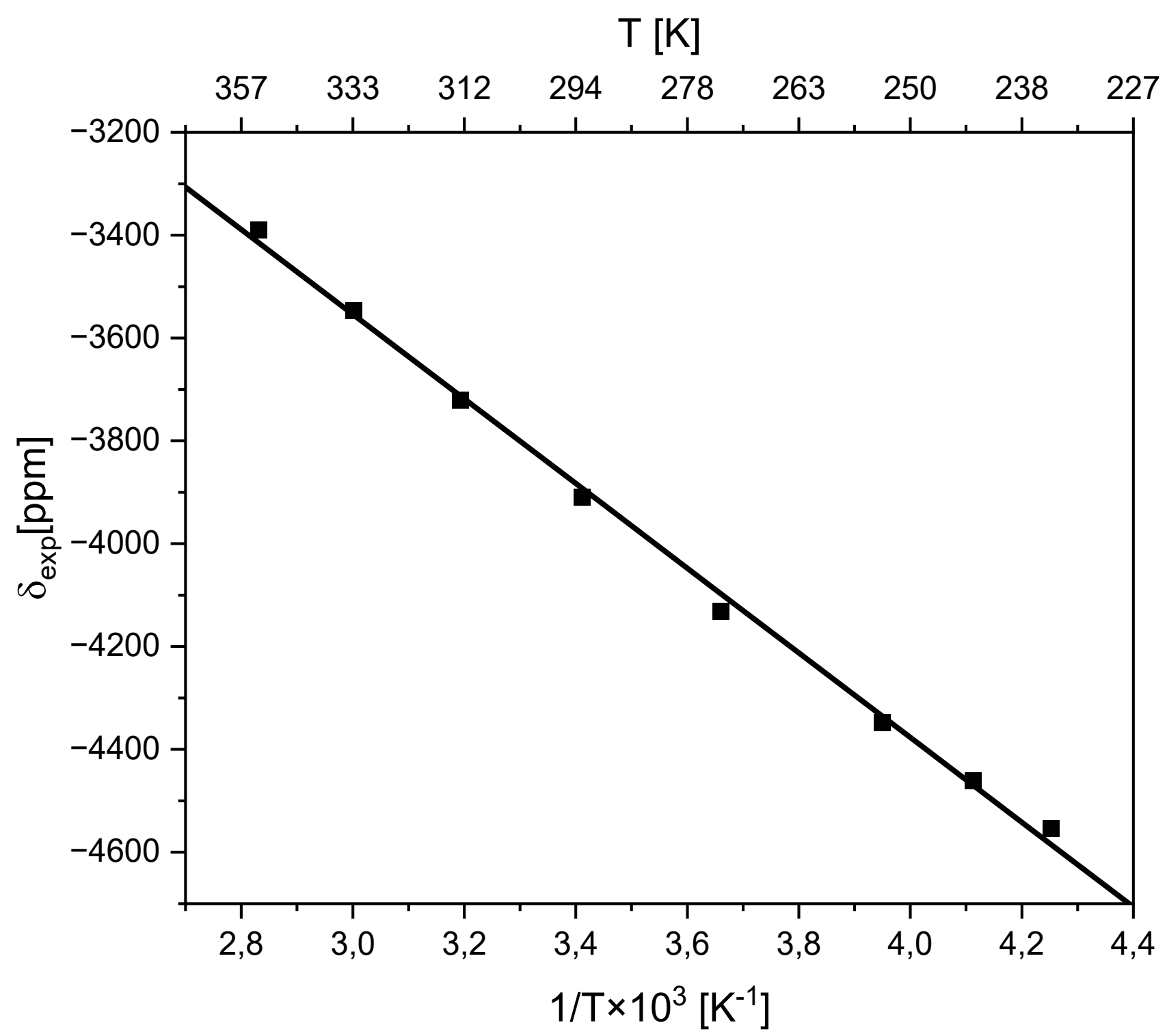

Figure S 34: Curie plot of the ^{31}P resonance of **2** (dots) and linear regression (line).

Table S 2: Parameters for the linear regression (m*x+n) of the VT ^{1}H, ^{13}C and ^{31}P resonances of **2**.

Nucleus	m	n	Adjusted R^2	Nucleus	m	n	Adjusted R^2
H^2	-0.317 ± 0.058	14,546± 0,208	0.804	C^5	72.767 ± 0.591	163.958 ± 2.118	1.000
H^4	4.839± 0.177	12,437± 0,635	0.990	C^6	-110.269 ± 2.375	5.458 ± 8.518	0.997
H^8	0.525± 0.018	2,495 ± 0,065	0.992	C^7	-20.692 ± 0.473	4.220 ± 1.745	0.997
H^9	3.290 ± 0.002	19,174 ± 0,007	1.000	C^8	18.974 ± 0.402	64.586 ± 1.443	0.997
H^{11}	-3.945 ± 0.153	7.163 ± 0.547	0.991	C^9	-69.167 ± 1.158	-43.393 ± 4.153	0.998
C^1	68.999 ± 1.010	209.730 ± 3.624	0.999	C^{10}	-70.644 ± 1.121	-0.261 ± 3.493	0.999
C^2	-55.341 ± 0.997	55.302 ± 3.575	0.998	C^{11}	20.424 ± 0.647	109.147 ± 2.015	0.997
C^3	87.481 ± 1.059	209.915 ± 3.797	0.999	P	-823.266 ± 17.010	-1083.907± 60,995	0.997
C^4	-51.527 ± 0.969	60.056 ± 3,473	0.998				

Compound **4:**

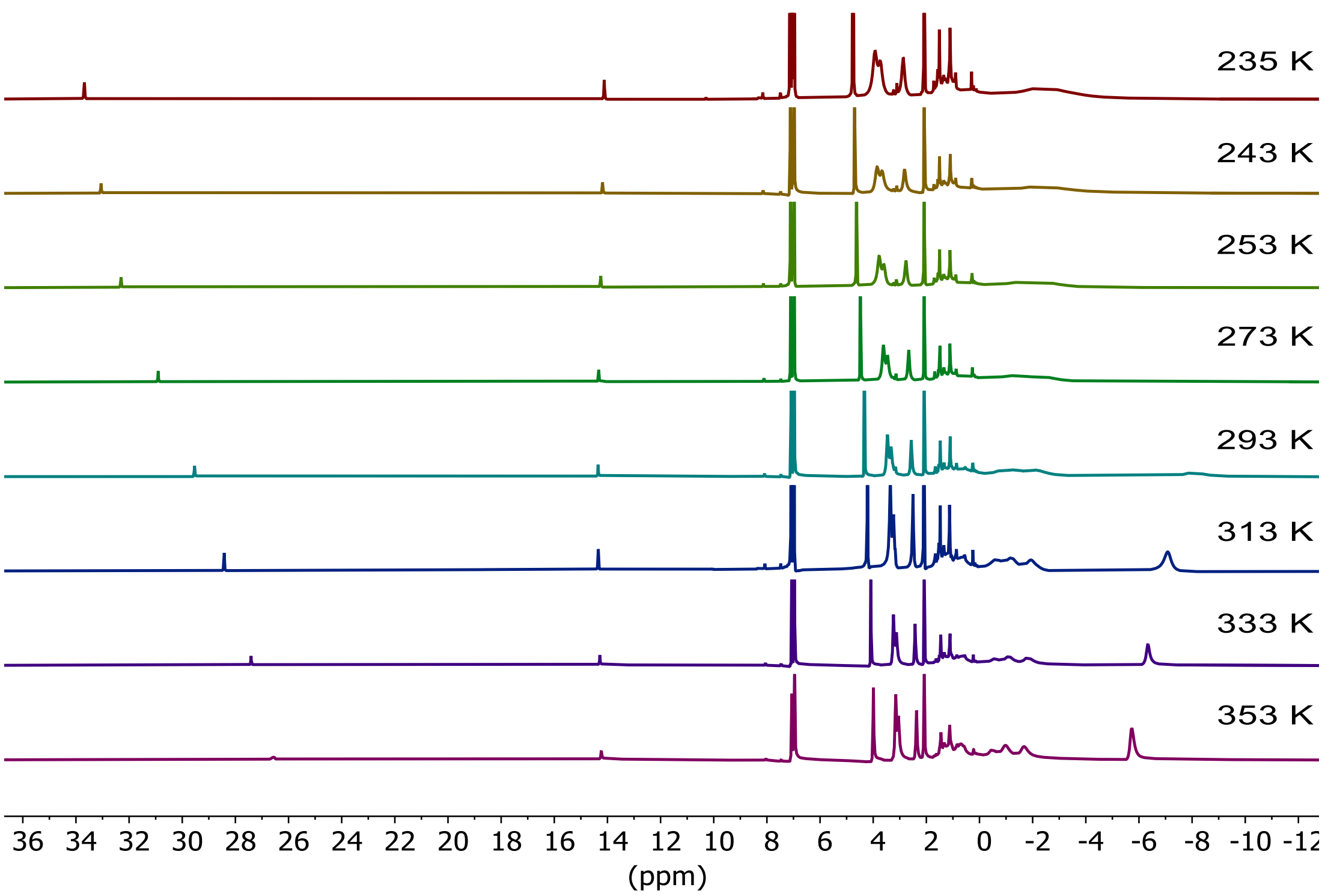

Figure S 35: ^{1}H NMR spectrum of **4** (tol-d_6, 600 MHz, 295 K) at different temperatures (235-353 K).

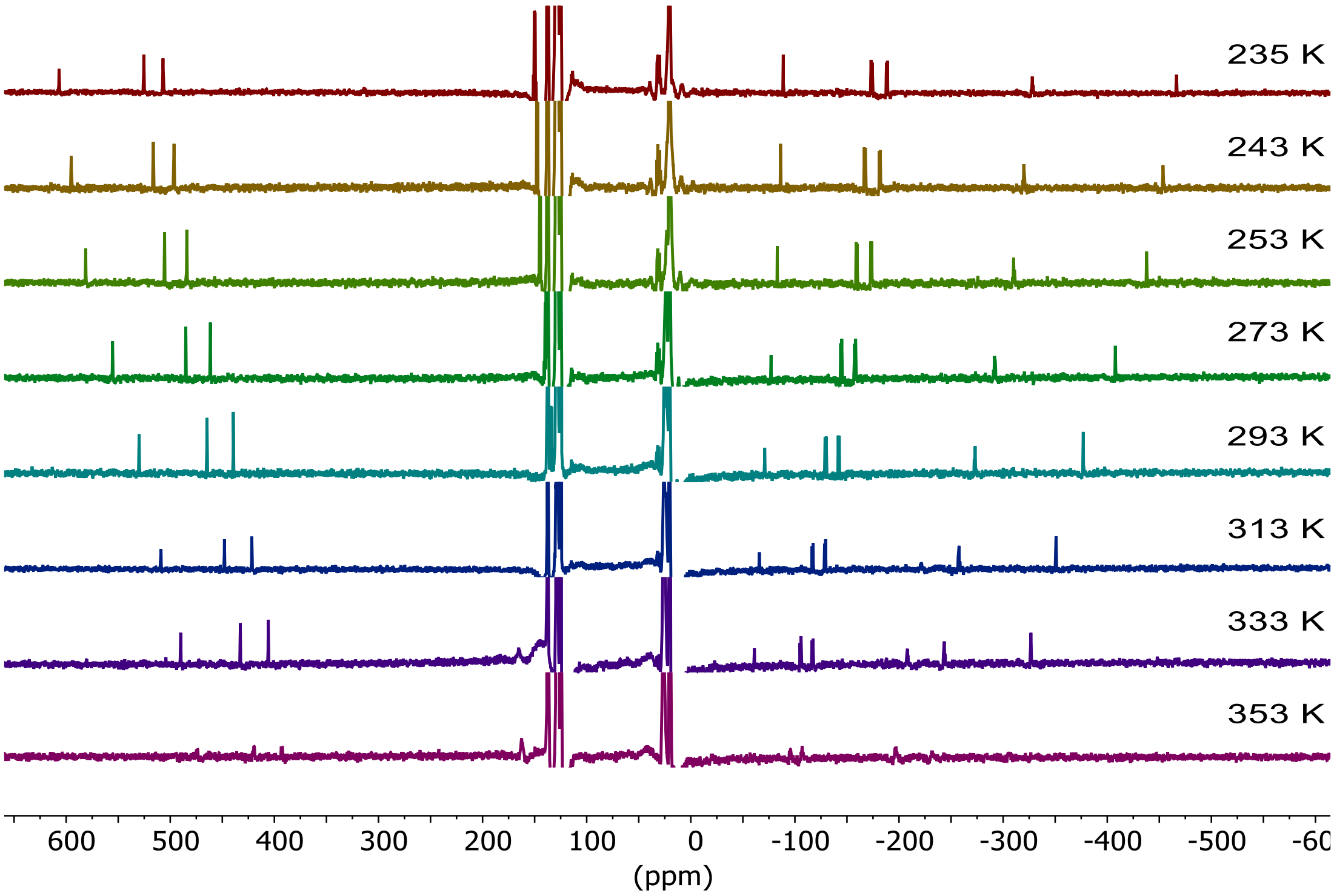

Figure S 36: ^{13}C NMR spectrum of **4** (tol-d_6, 151 MHz, 295 K) at different temperatures (235-353 K).

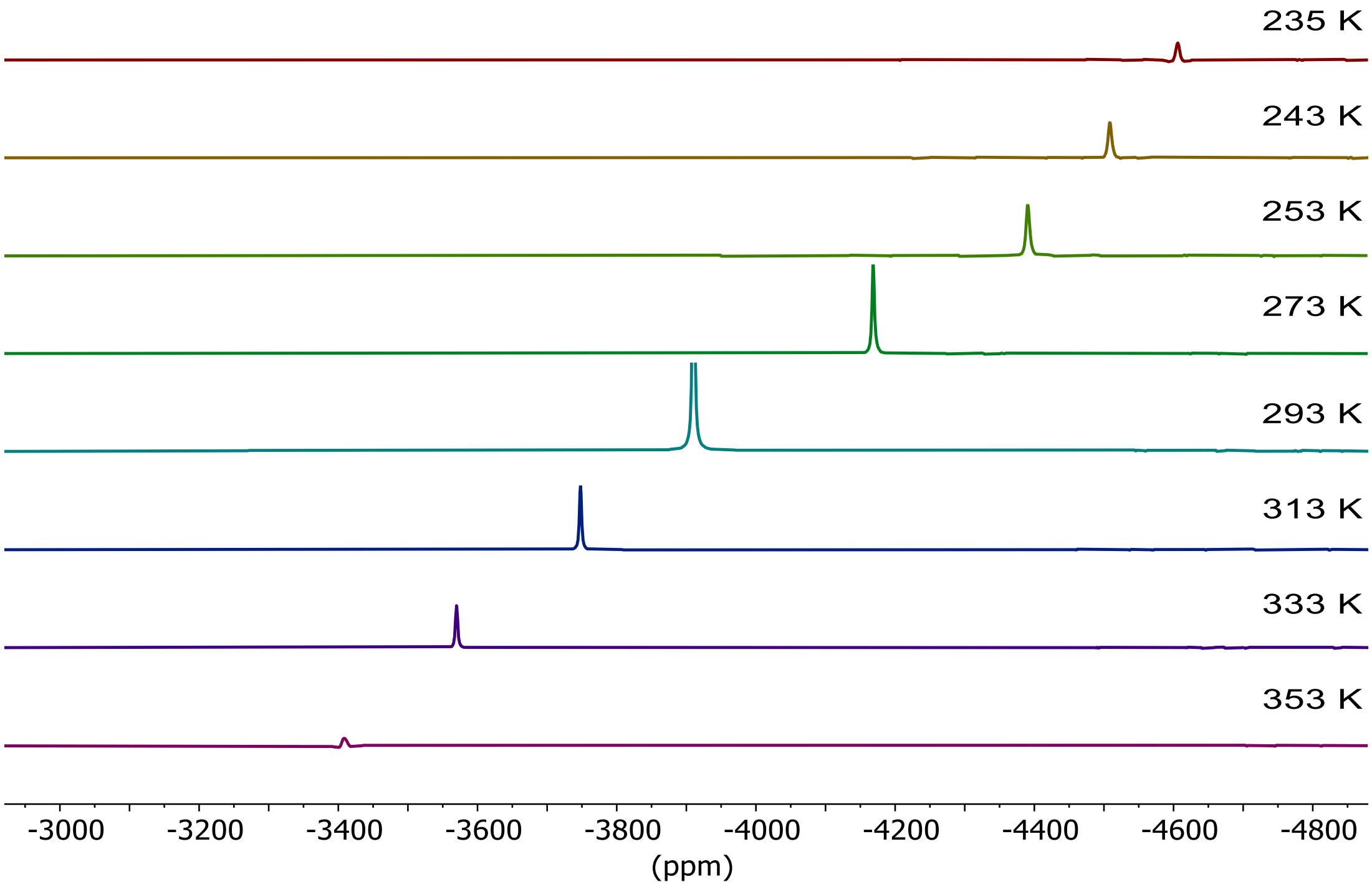

Figure S 37: ^{31}P NMR spectrum of **4** (tol-d_6, 242 MHz, 295 K) at different temperatures (235-353 K).

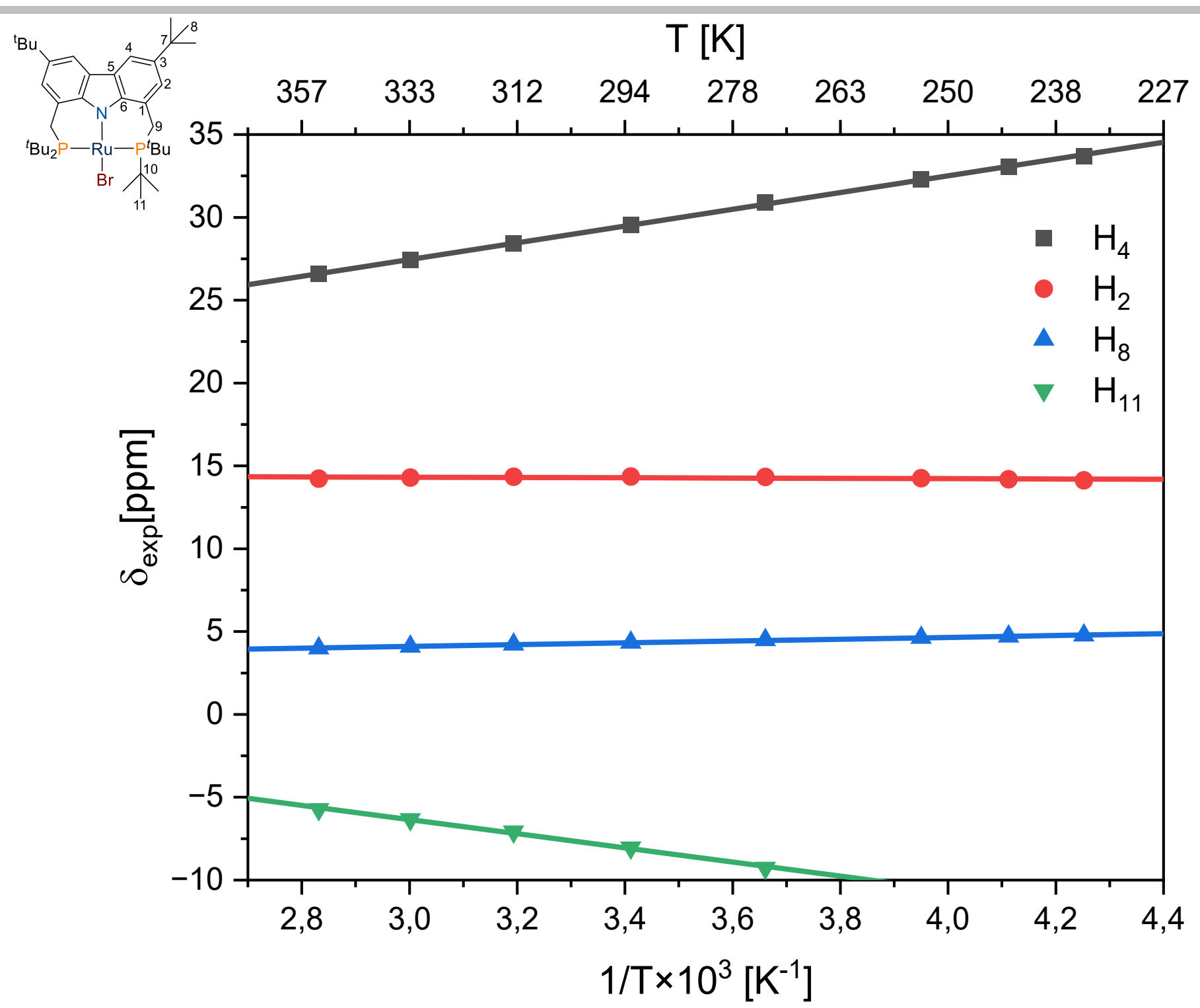

Figure S 38: Curie plot of the ^{1}H resonances of **4** (dots) and linear regression (lines).

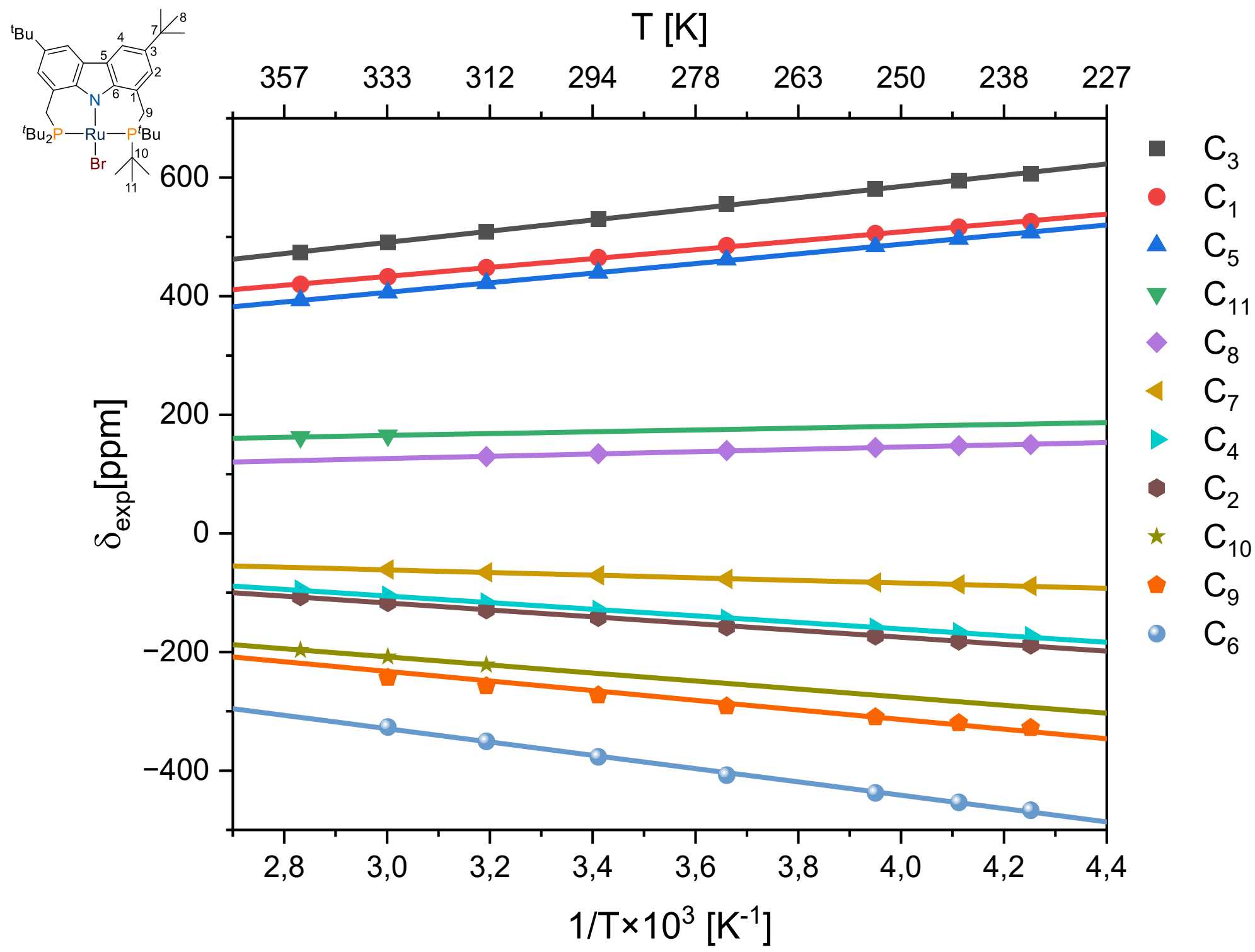

Figure S 39: Curie plot of the ^{13}C resonances of **4** (dots) and linear regression (lines).

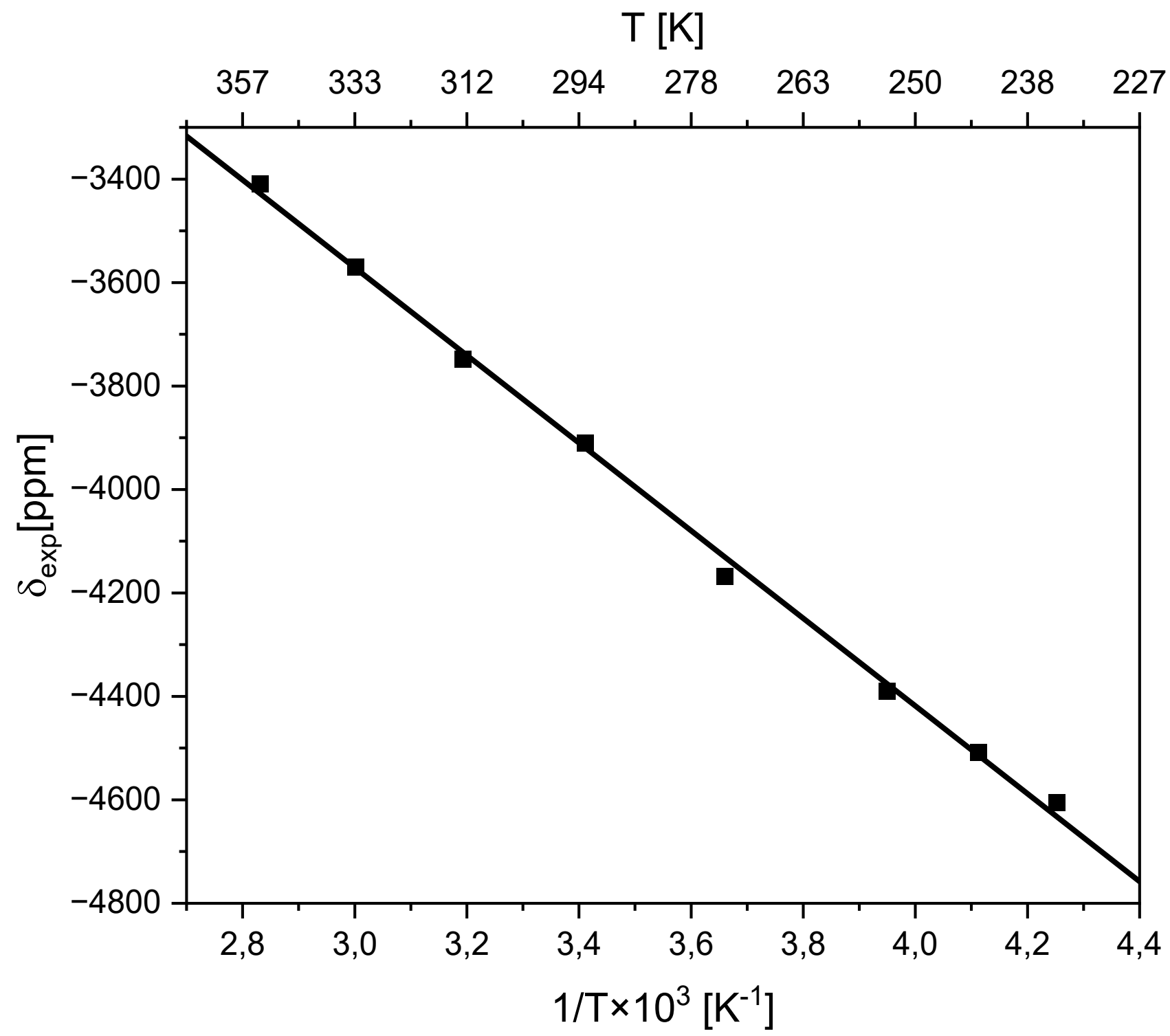

Figure S 40: Curie plot of the ^{31}P resonance of **4** (dots) and linear regression (line).

Table S 3: Parameters for the linear regression (m*x+n) of the VT ^{1}H, ^{13}C and ^{31}P resonances of **4**.

Nucleus	m	n	Adjusted R^2	Nucleus	m	n	Adjusted R^2
H^2	-0.087 ± 0.051	14.573 ± 0.185	0.212	C^5	81.136 ± 0.600	162.960 ± 2.151	1.000
H^4	5.055 ± 0.051	12.281 ± 0.182	0.999	C^6	-112.290 ± 2.462	7.401 ± 9.064	0.997
H^8	0.545 ± 0.011	2.461 ± 0.039	0.997	C^7	-22.200 ± 0.461	4.947 ± 1.695	0.997
H^9				C^8	19.405 ± 0.508	67.835 ± 1.921	0.997
H^{11}	-4.271 ± 0.157	6.456 ± 0.507	0.999	C^9	-81.197 ± 8.324	10.731 ± 29.848	0.931
C^1	75.003 ± 0.961	208.316 ± 3.445	0.999	C^{10}	-67.939 ± 1.875	-4.553 ± 5.649	0.998
C^2	-57.905 ± 0.896	56.137 ± 3.212	0.998	C^{11}	15.471	118.661	
C^3	94.361 ± 1.026	207.551 ± 3.680	0.999	P	-847.544 ± 15.917	-1028,580 ± 57,076	0.997
C^4	-55.600 ± 0.943	61.002 ± 3.382	0.998				

Compound **5:**

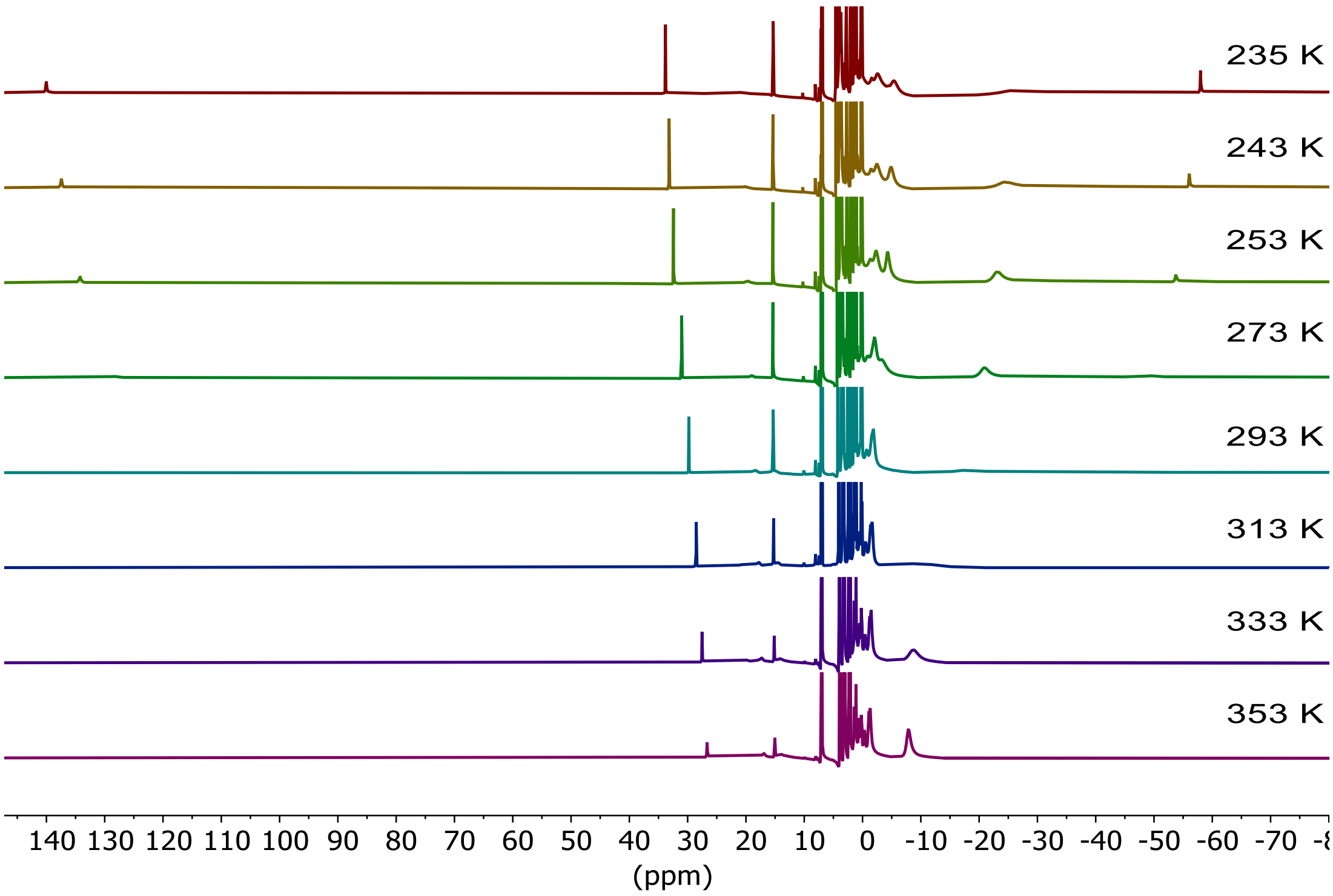

Figure S 41: ^{1}H NMR spectrum of **5** (tol-d$_6$, 600 MHz, 295 K) at different temperatures (235-353 K).

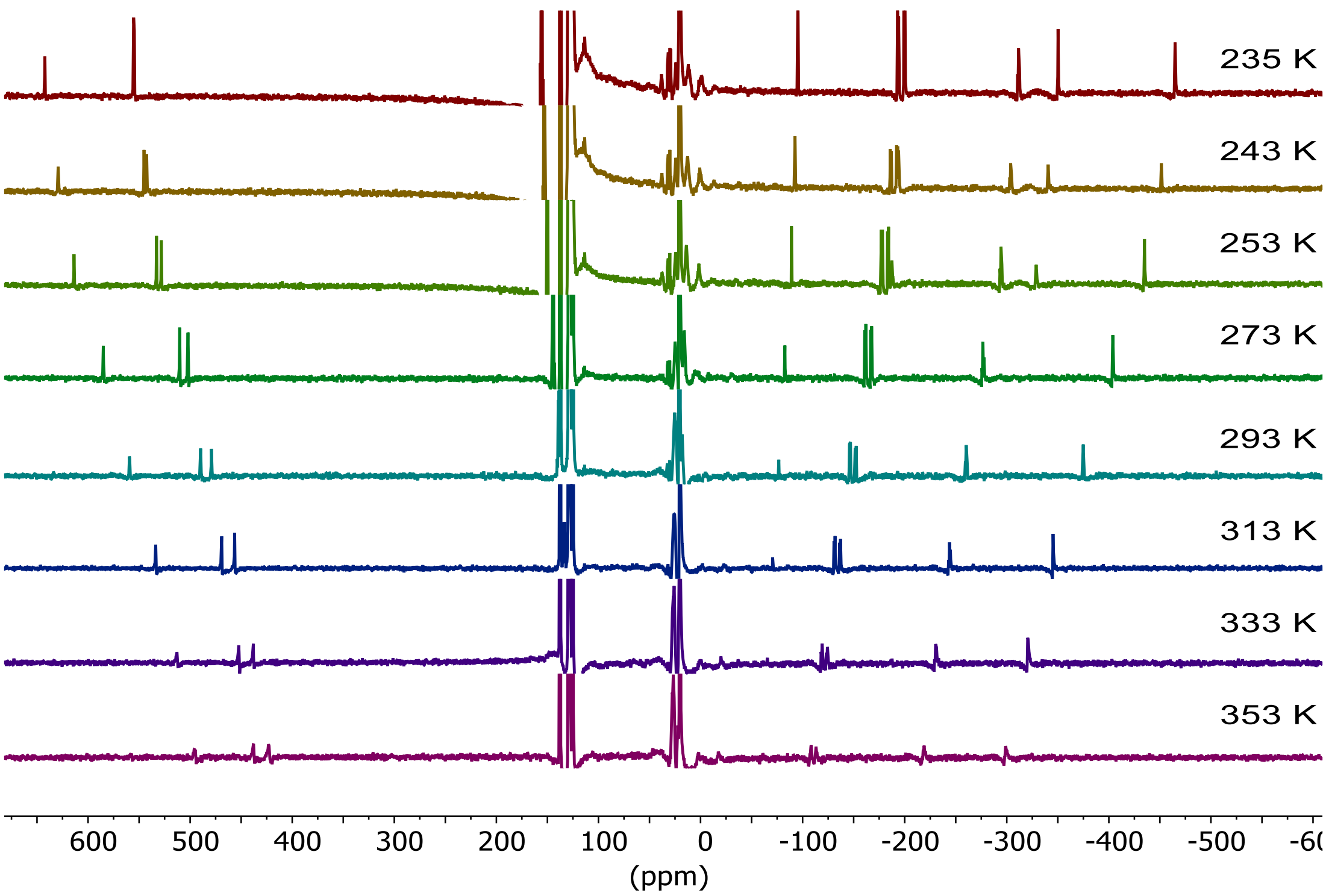

Figure S 42: ^{13}C NMR spectrum of **5** (tol-d$_6$, 151 MHz, 295 K) at different temperatures (235-353 K).

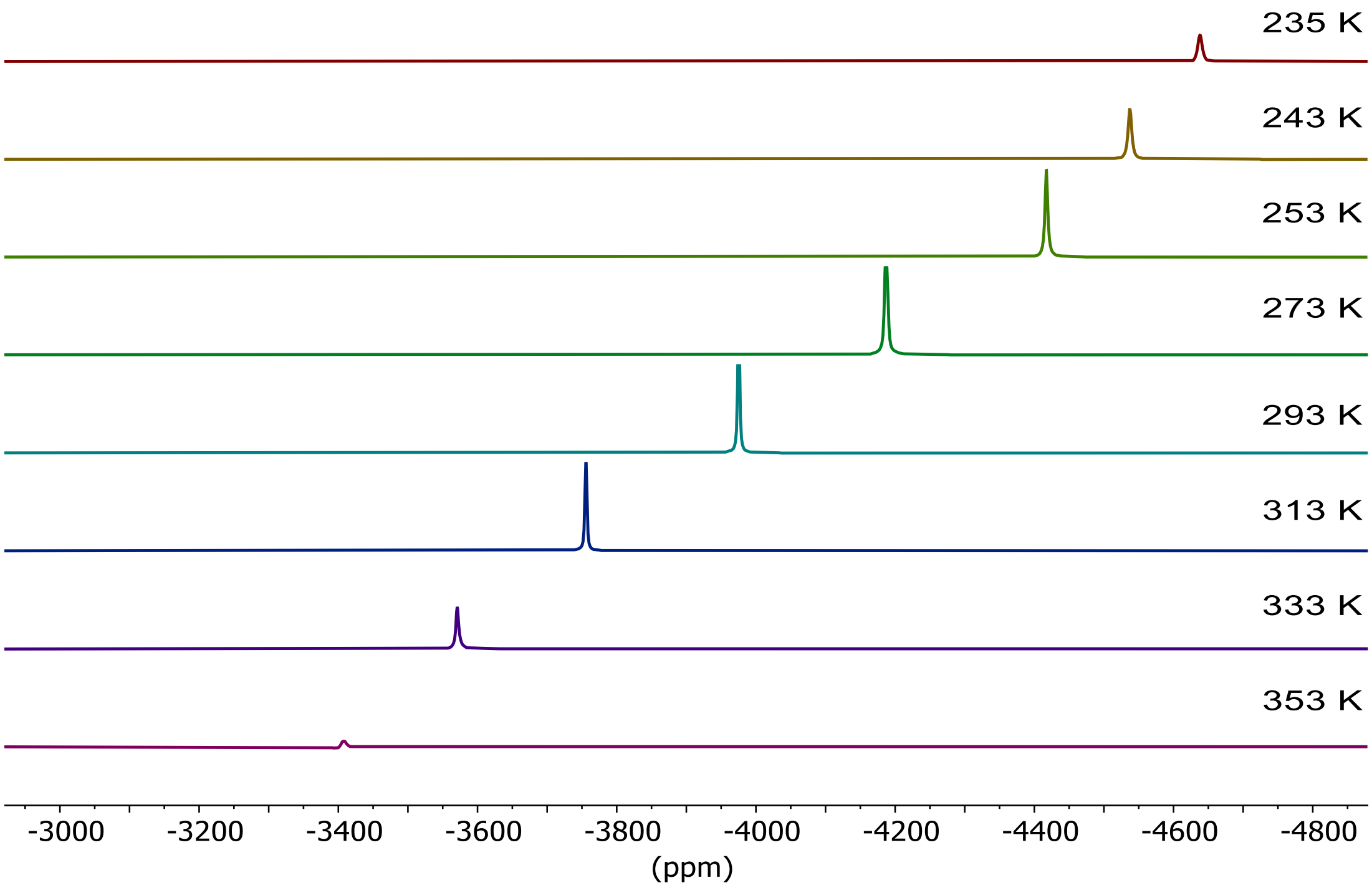

Figure S 43: ^{31}P NMR spectrum of **5** (tol-d6, 242 MHz, 295 K) at different temperatures (235-353 K).

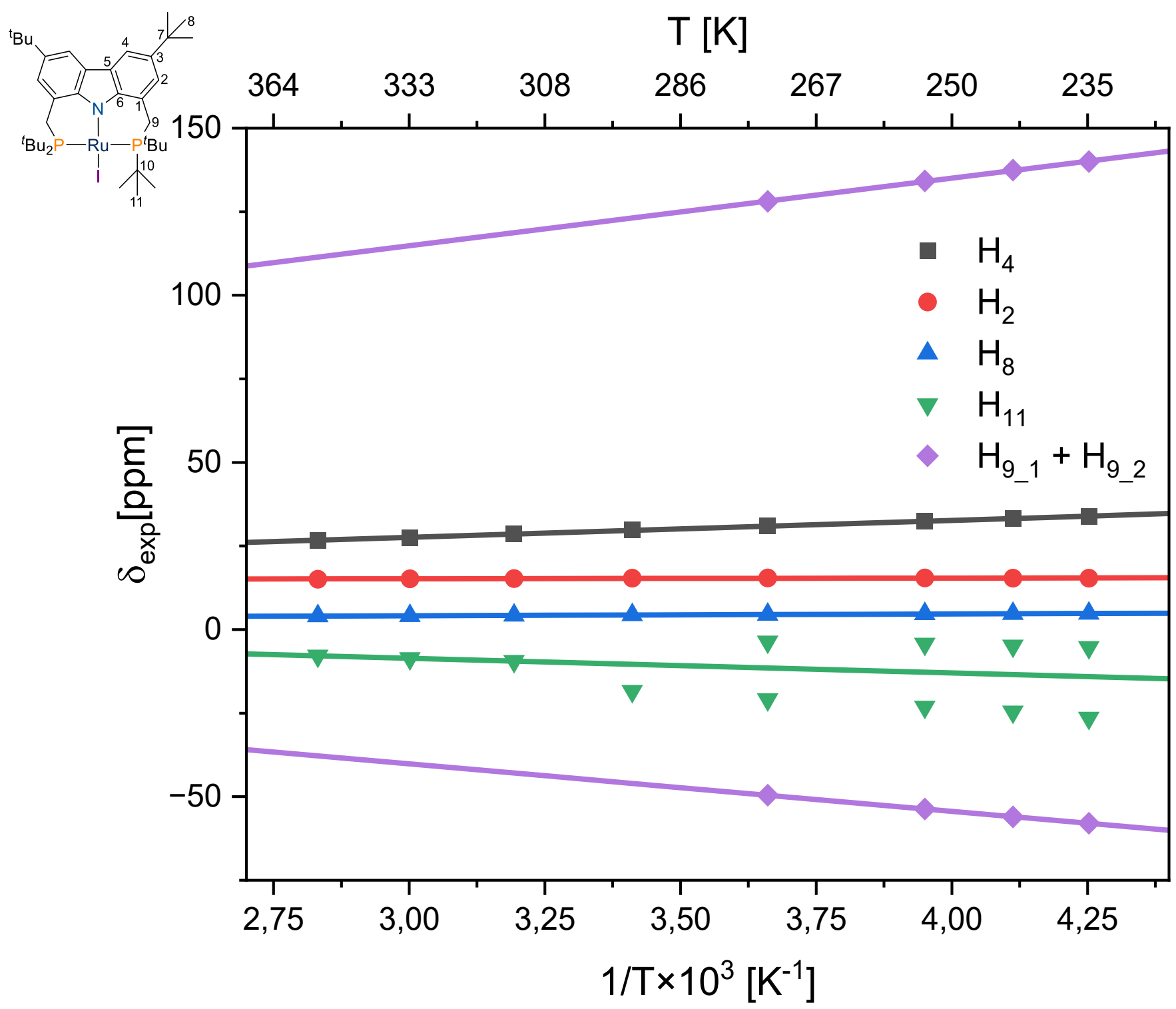

Figure S 44: Curie plot of the ^{1}H resonances of **5** (dots) and linear regression (lines). For the resonance H_{11} decoalescence into two signals is observed from 273 K and below. At 273 K and blow the decoalesced resonances of H_9 are also observed.

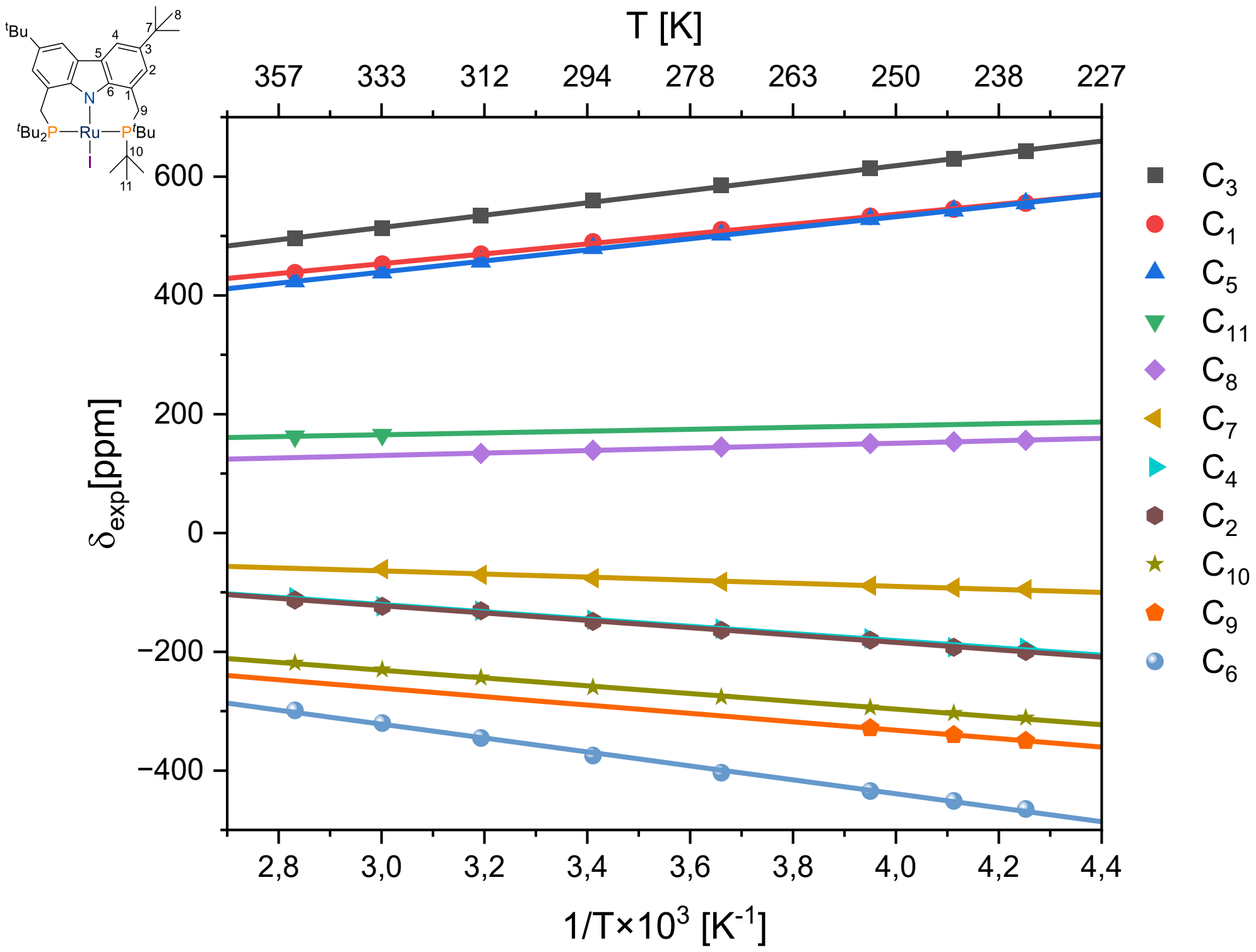

Figure S 45: Curie plot of the ^{13}C resonances of **5** (dots) and linear regression (lines).

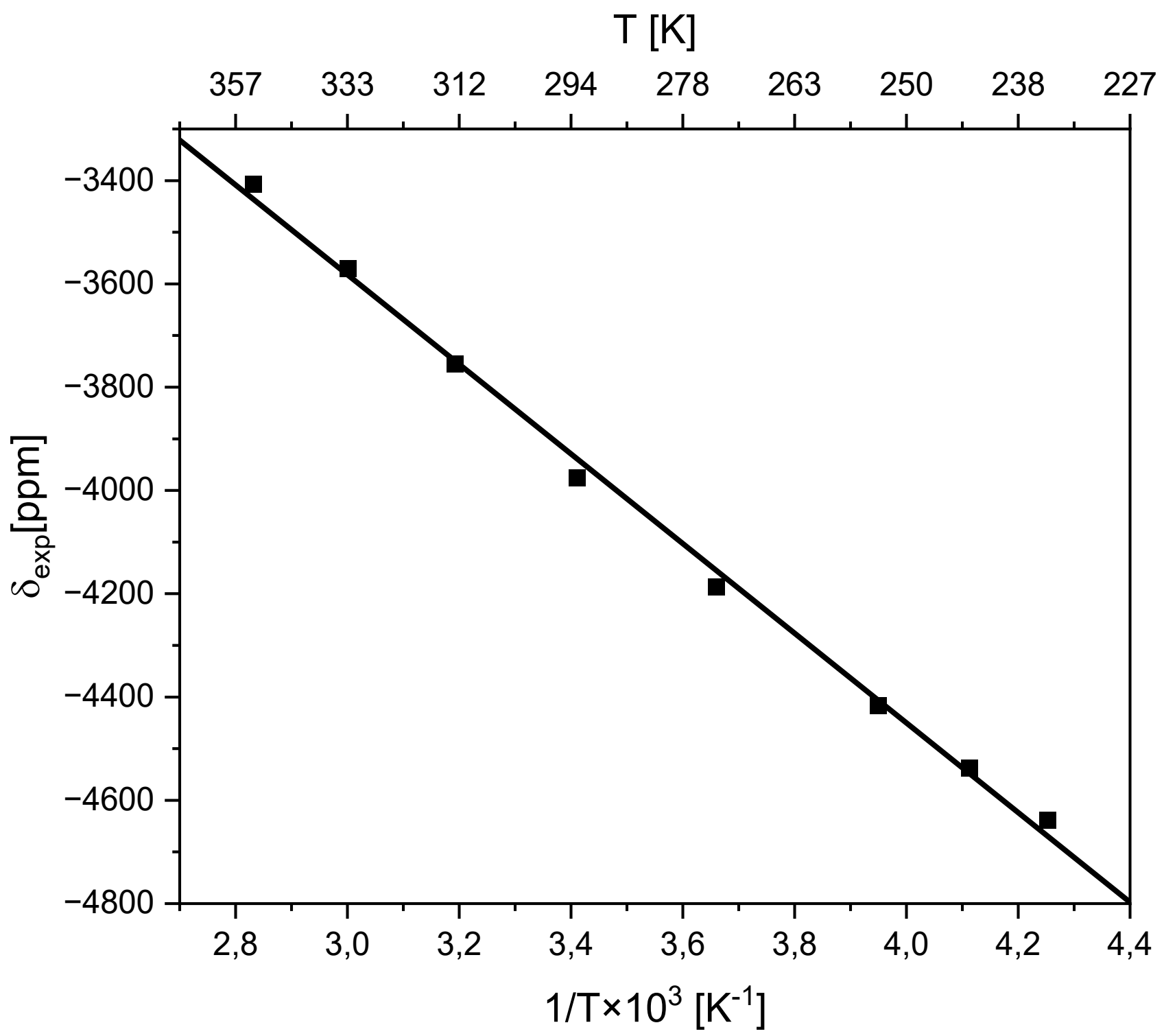

Figure S 46: Curie plot of the ^{31}P resonance of **5** (dots) and linear regression (line).

Table S 4: Parameters for the linear regression (m*x+n) of the VT ^{1}H, ^{13}C and ^{31}P resonances of **5**.

Nucleus	m	n	Adjusted R^2	Nucleus	m	n	Adjusted R^2
H^2	0.196 ± 0.053	14.578 ± 0.189	0.649	C^4	-60.786 ± 1.973	61.522 ± 7.073	0.993
H^4	5.070 ± 0.064	12.362 ± 0.231	0.999	C^5	93.310 ± 0.862	159.127 ± 3.092	0.999
H^8	0.545 ± 0.011	2.461 ± 0.039	0.997	C^6	-117.349 ± 2.526	29.964 ± 9.059	0.997
H^{9_1}	20.226 ± 0.437	54.152 ± 1.750	0.999	C^7	-25.945 ± 1.663	13.650 ± 6.121	0.976
H^{9_2}	-14.191 ± 0.091	2.331 ± 0.364	1.000	C^8	20.679 ± 0.547	68.157 ± 2.068	0.997
H^{11}	-4.362 ± 0.183	4.441 ± 0.552	0.996	C^9	-70.814 ± 0.962	-49.157 ± 3.949	1.000
C^1	83.179 ± 1.215	203.651 ± 4.356	0.999	C^{10}	-65.662 ± 1.141	-34.265 ± 4.092	0.998
C^2	-61.867 ± 1.203	62.891 ± 4.315	0.997	C^{11}	15.471	118.661	
C^3	103.990 ± 1.301	202.169 ± 4.666	0.999	P	-868.080 ± 19.561	-977.859 ± 70.144	0.996

Compound **3:**

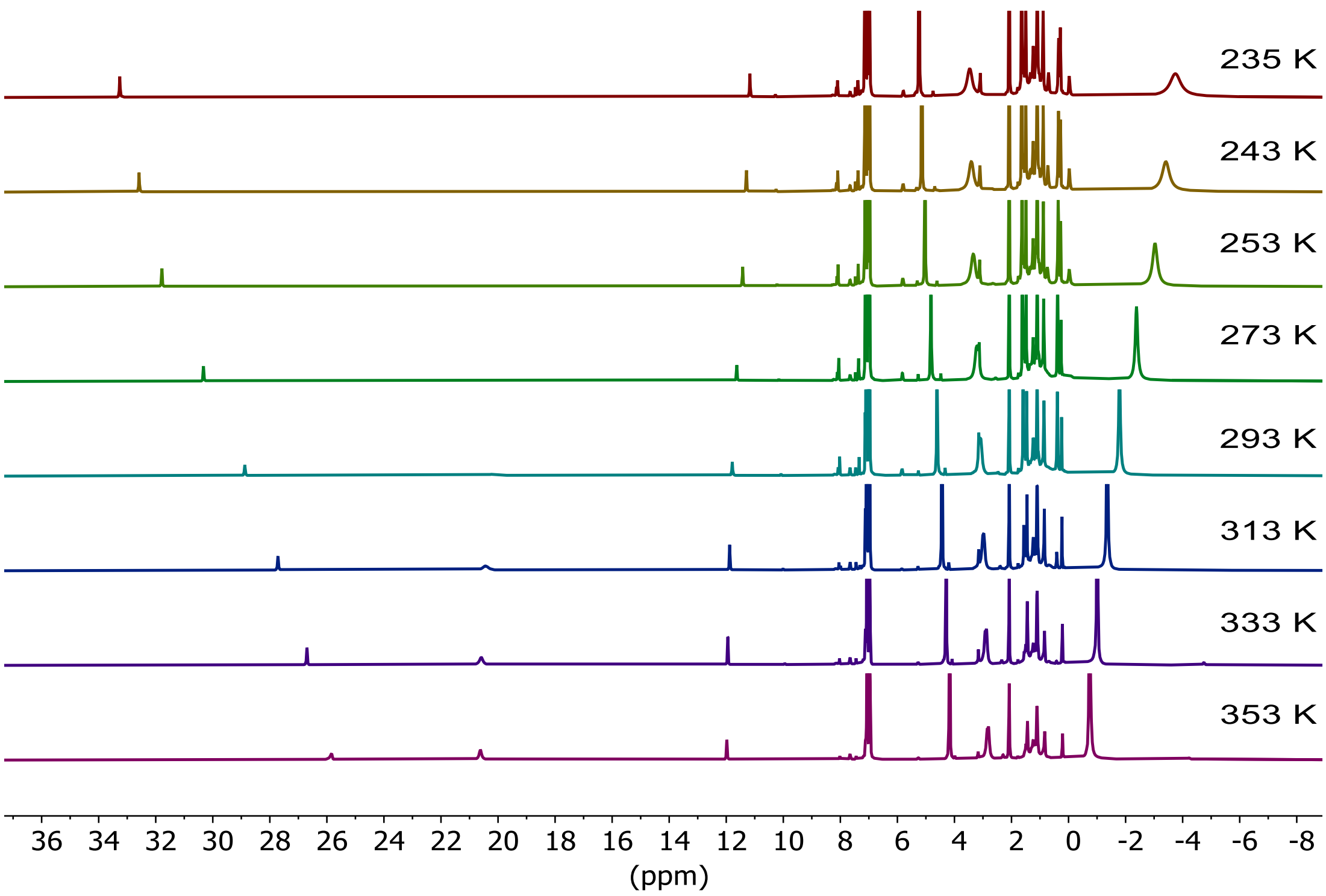

Figure S 47: ^{1}H NMR spectrum of **3** (tol-d_6, 600 MHz, 295 K) at different temperatures (235-353 K).

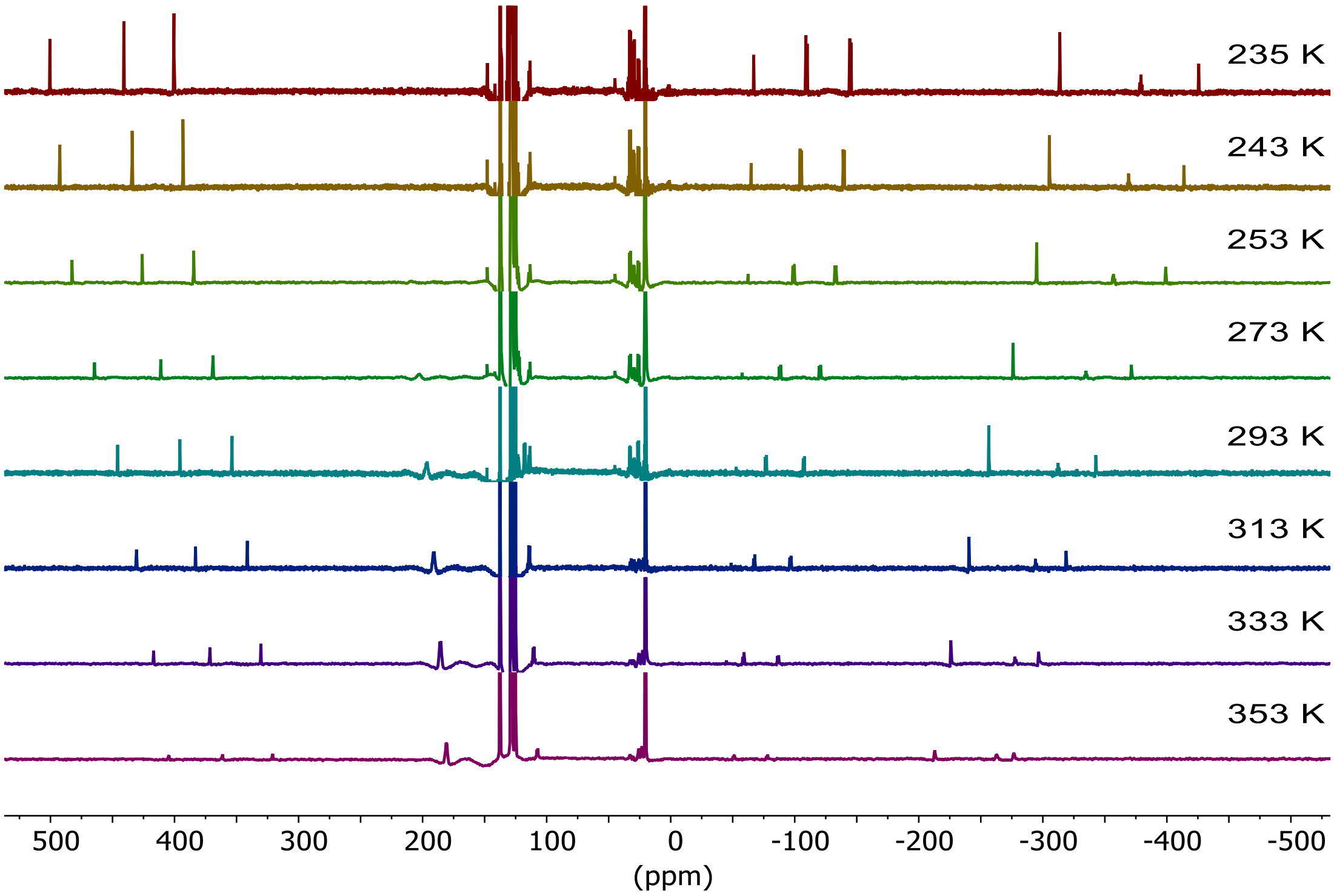

Figure S 48: ^{13}C NMR spectrum of **3** (tol-d_6, 151 MHz, 295 K) at different temperatures (235-353 K).

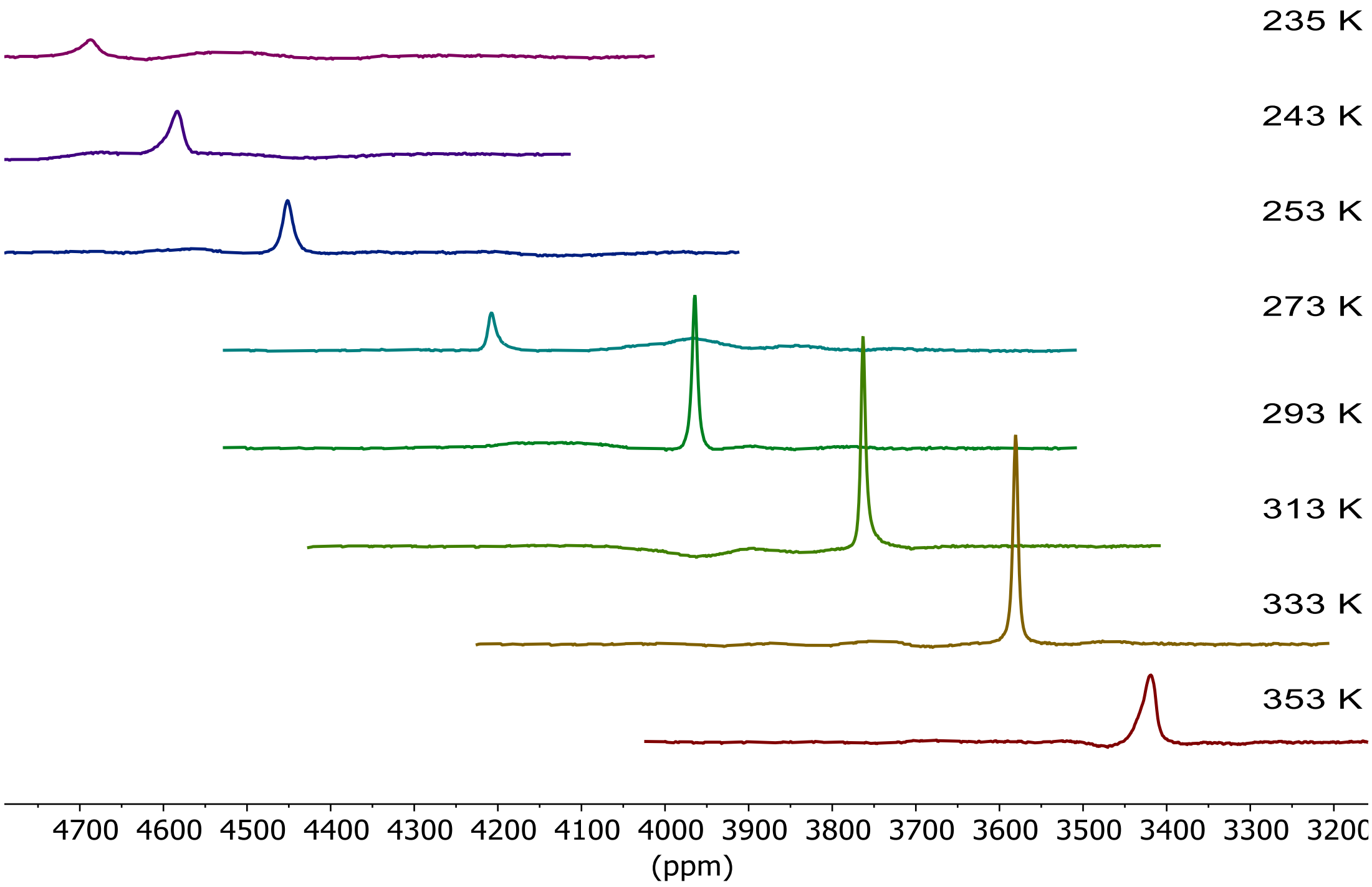

Figure S 49: ^{19}F NMR spectrum of **3** (tol-d_6, 567 MHz, 295 K) at different temperatures (235-353 K).

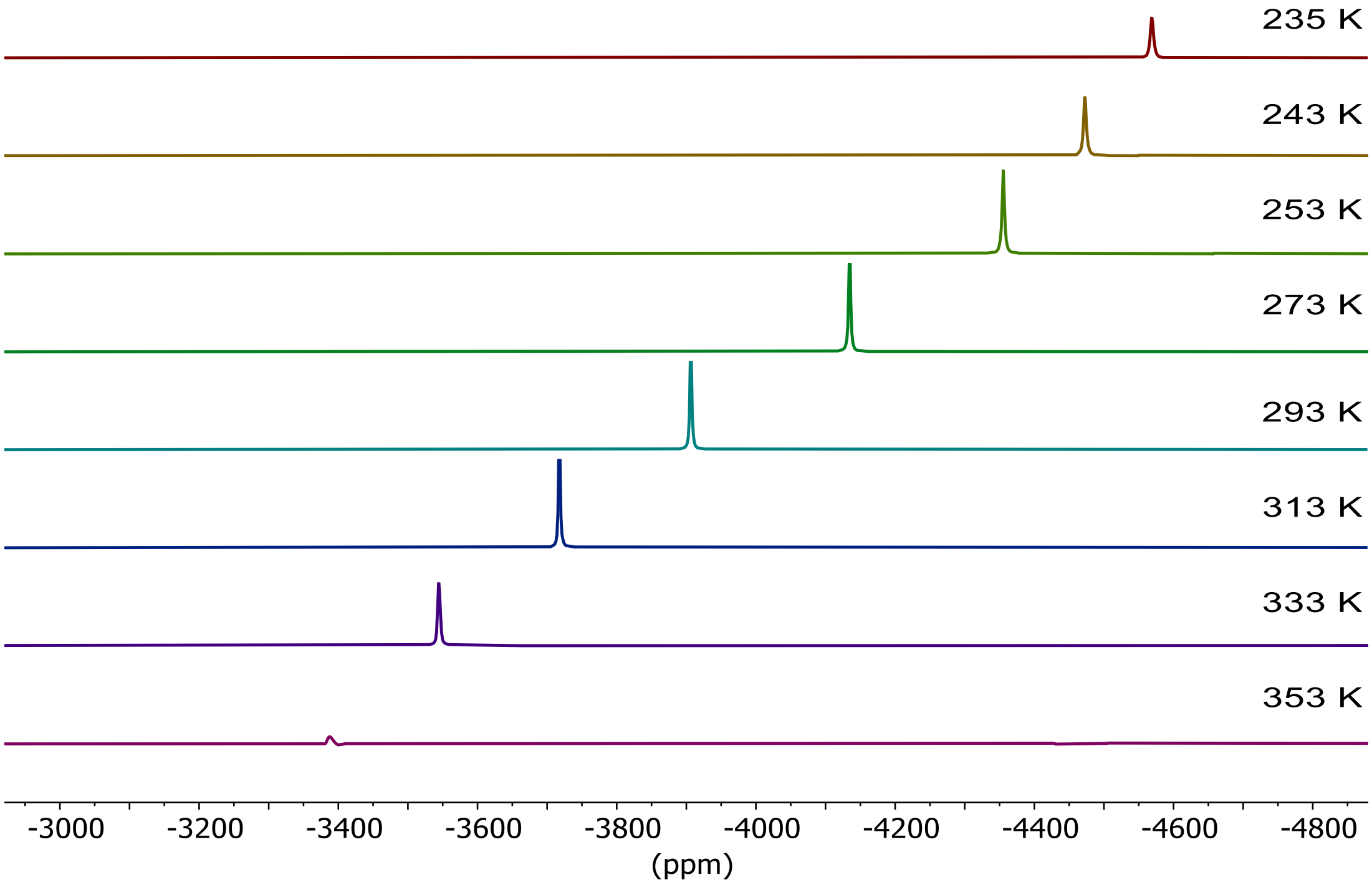

Figure S 50: ^{31}P NMR spectrum of **3** (tol-d$_6$, 242 MHz, 295 K) at different temperatures (235-353 K).

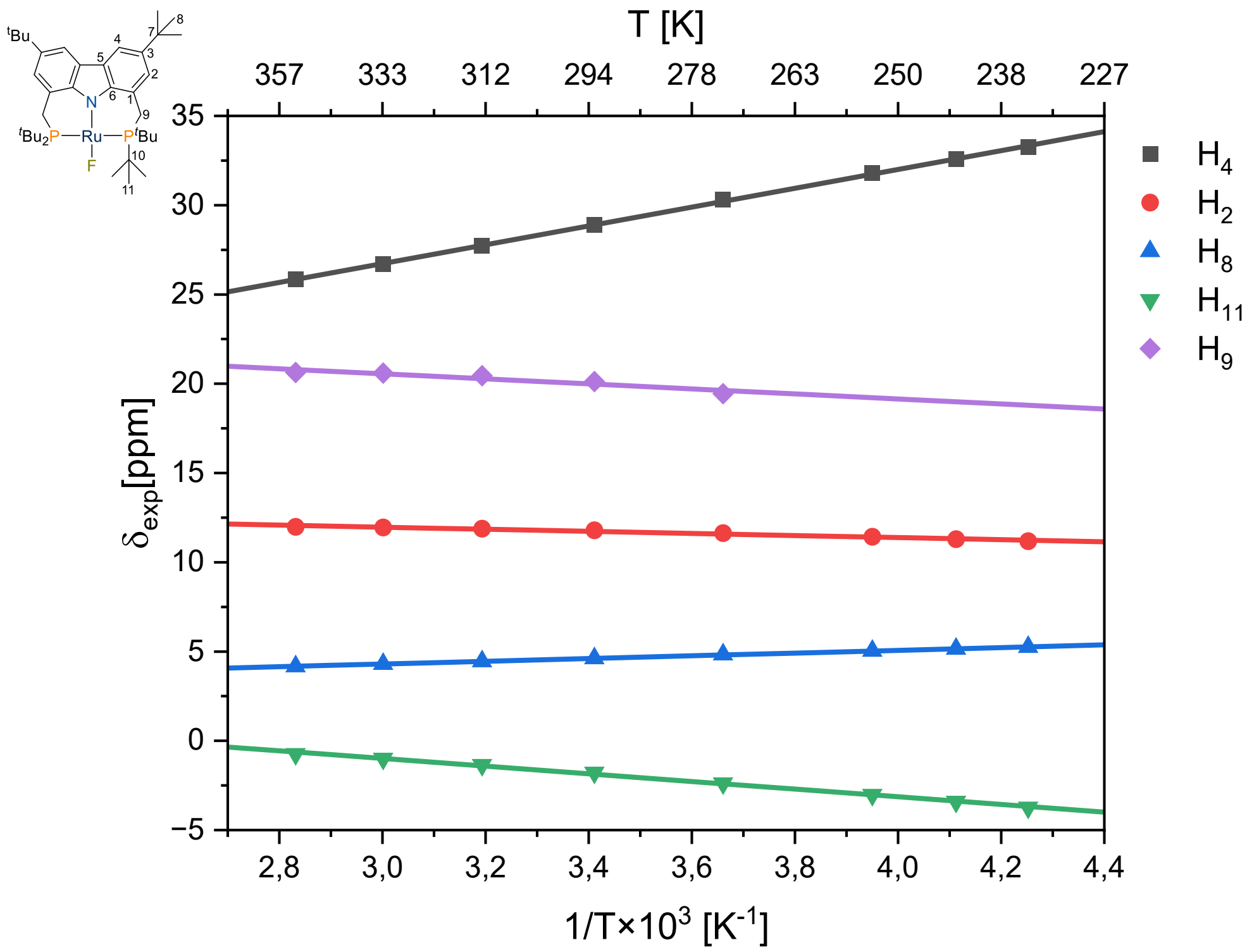

Figure S 51: Curie plot of the ^{1}H resonances of **3** (dots) and linear regression (lines).

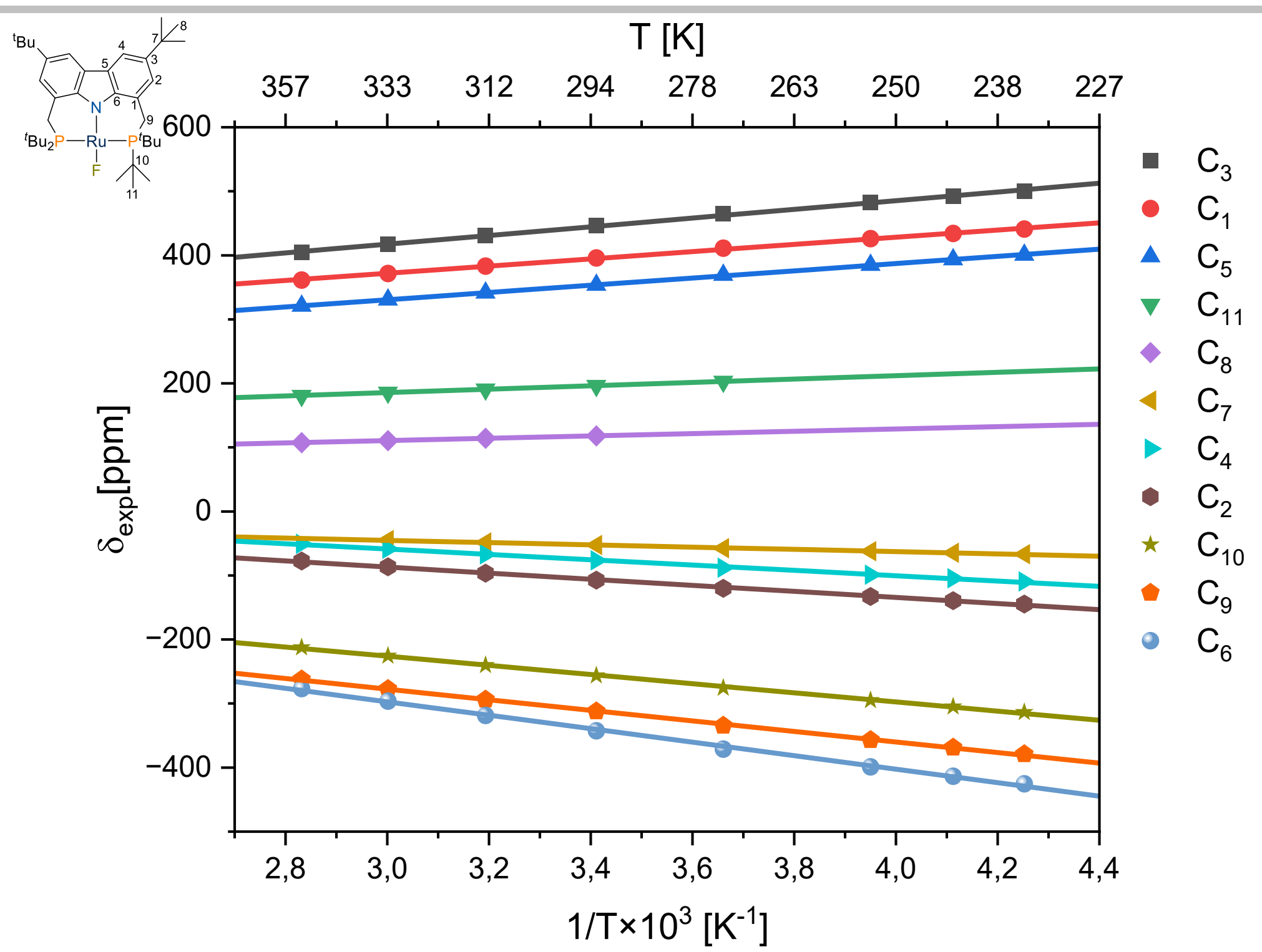

Figure S 52: Curie plot of the ^{13}C resonances of **3** (dots) and linear regression (lines).

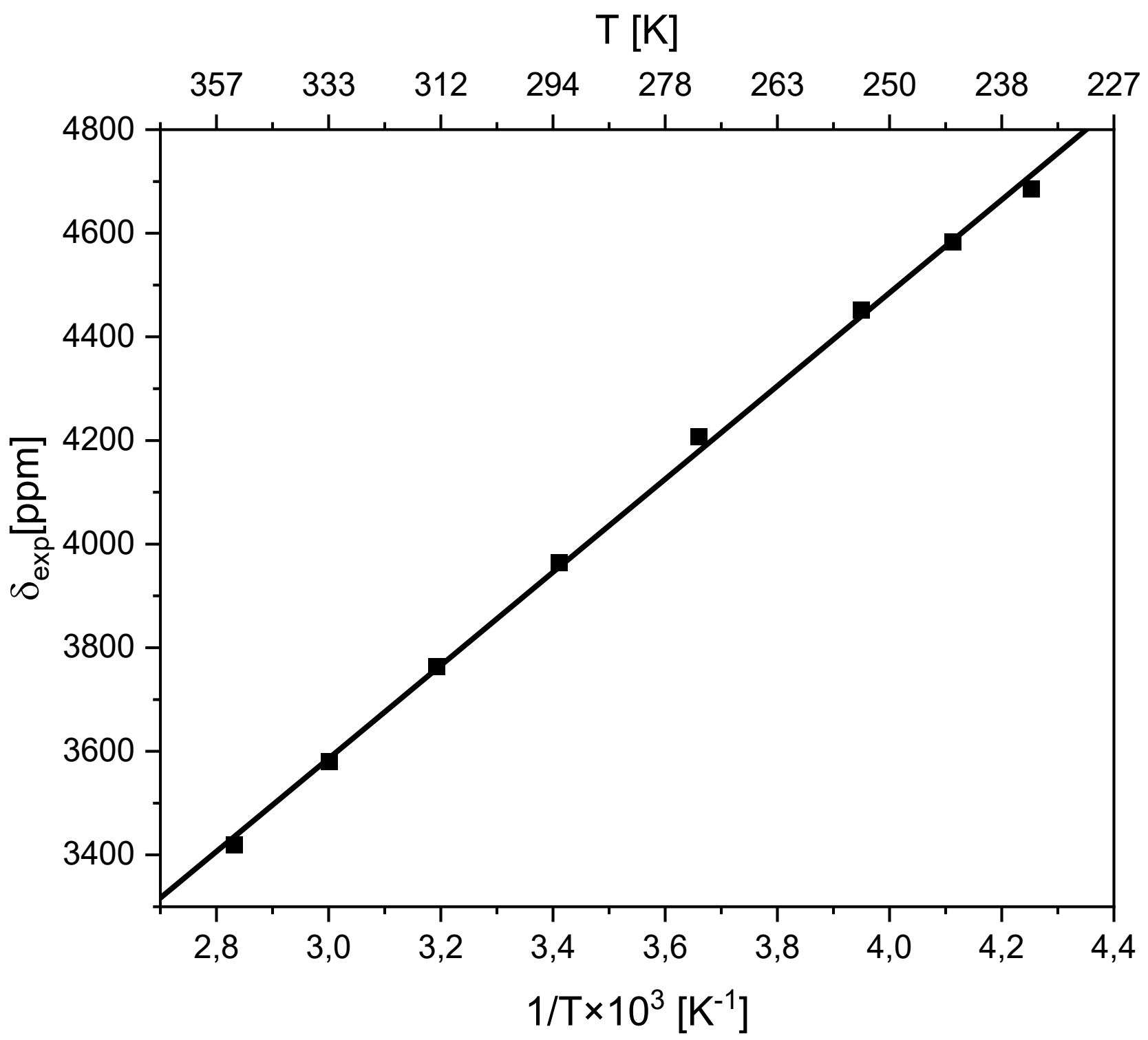

Figure S 53: Curie plot of the ^{19}F resonance of **3** (dots) and linear regression (line).

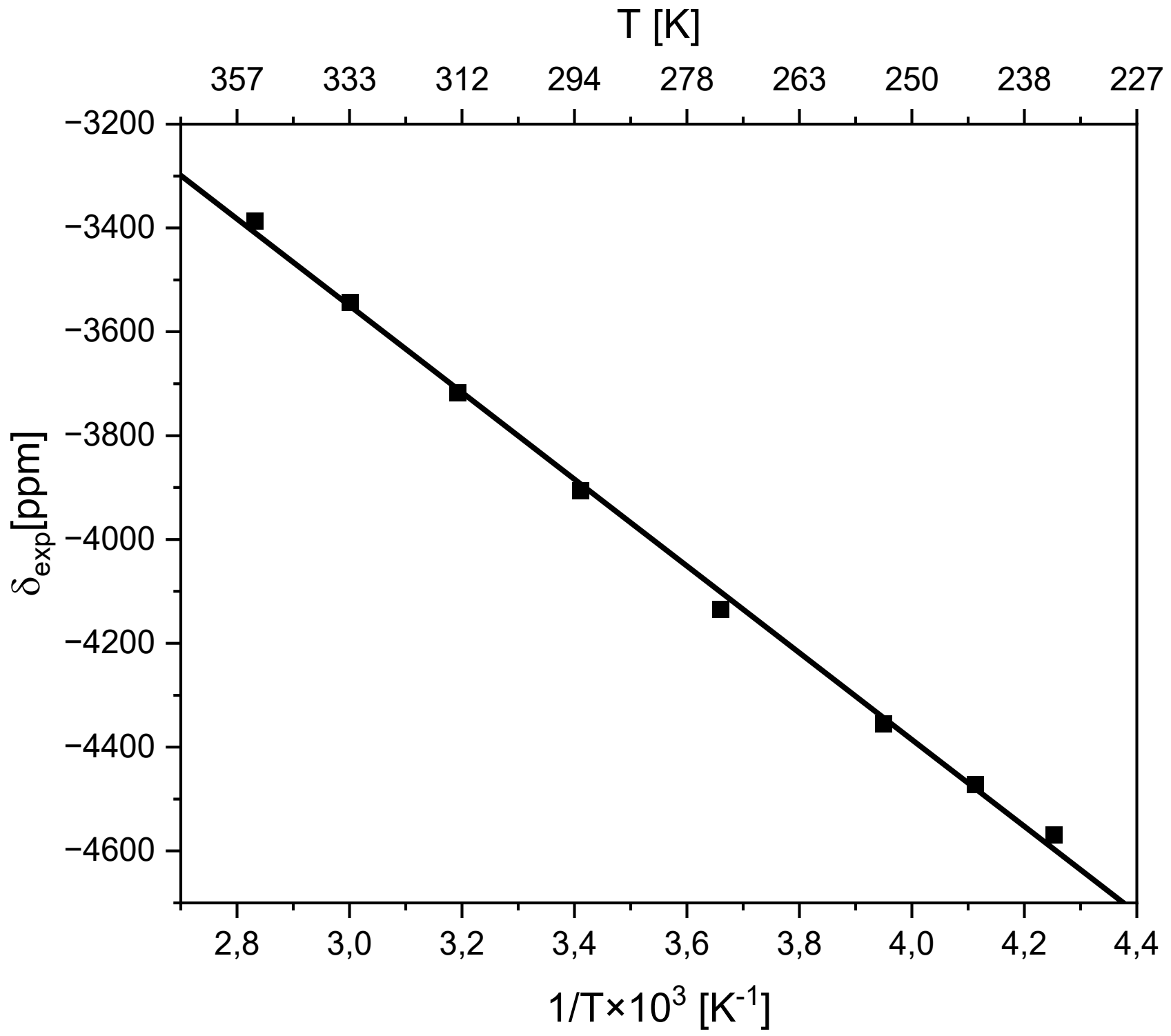

Figure S 54: Curie plot of the ^{31}P resonance of **3** (dots) and linear regression (line).

Table S 5: Parameters for the linear regression (m*x+n) of the VT ^{1}H, ^{13}C, ^{19}F and ^{31}P resonances of **3**.

Nucleus	m	n	Adjusted R^2	Nucleus	m	n	Adjusted R^2
H^2	-0.581 ± 0.041	13.703 ± 0.147	0.966	C^5	56.462 ± 0.453	161.126 ± 1.623	1.000
H^4	5.280 ± 0.045	10.881 ± 0.162	0.999	C^6	-105.375 ± 2.082	18.687 ± 7.467	0.997
H^8	0.766 ± 0.008	1.996 ± 0.028	0.999	C^7	-17.677 ± 0.400	7.733 ± 1.471	0.997
H^9	-1.406 ± 0.292	24.770 ± 0.945	0.847	C^8	18.107 ± 0.142	56.151 ± 0.443	1.000
H^{11}	-2.145 ± 0.048	5.435 ± 0.171	0.997	C^9	-82.544 ± 1.109	-30.110 ± 3.976	0.999
C^1	56.344 ± 0.884	202.823 ± 3.169	0.998	C^{10}	-71.437 ± 1.118	-11.976 ± 4.008	0.998
C^2	-47.559 ± 0.932	55.700 ± 3.342	0.997	C^{11}	26.446 ± 0.333	106.074 ± 1.078	0.999
C^3	67.947 ± 1.023	213.417 ± 3.670	0.998	F	-835.799 ± 15.308	-1042.326 ± 54.892	0.998
C^4	-41.601 ± 0.809	65.875 ± 2.901	0.997	P	898.720 ± 12.781	889.814 ± 45.830	0.999

5.2 Estimation of the T^{-2} dependence for ^1H resonances of H^2 and H^4 and calculation of *D*

The T^{-2} dependence for ^1H resonances of H^2 and H^4 form complexes **2-5** was estimated by taking into account the DFT calculated δ_{dia} values (see Section 6.4) as the 1/T = 0 limit and fitting the results with a second order polynomial to extract T^{-1} and T^{-2} dependence.

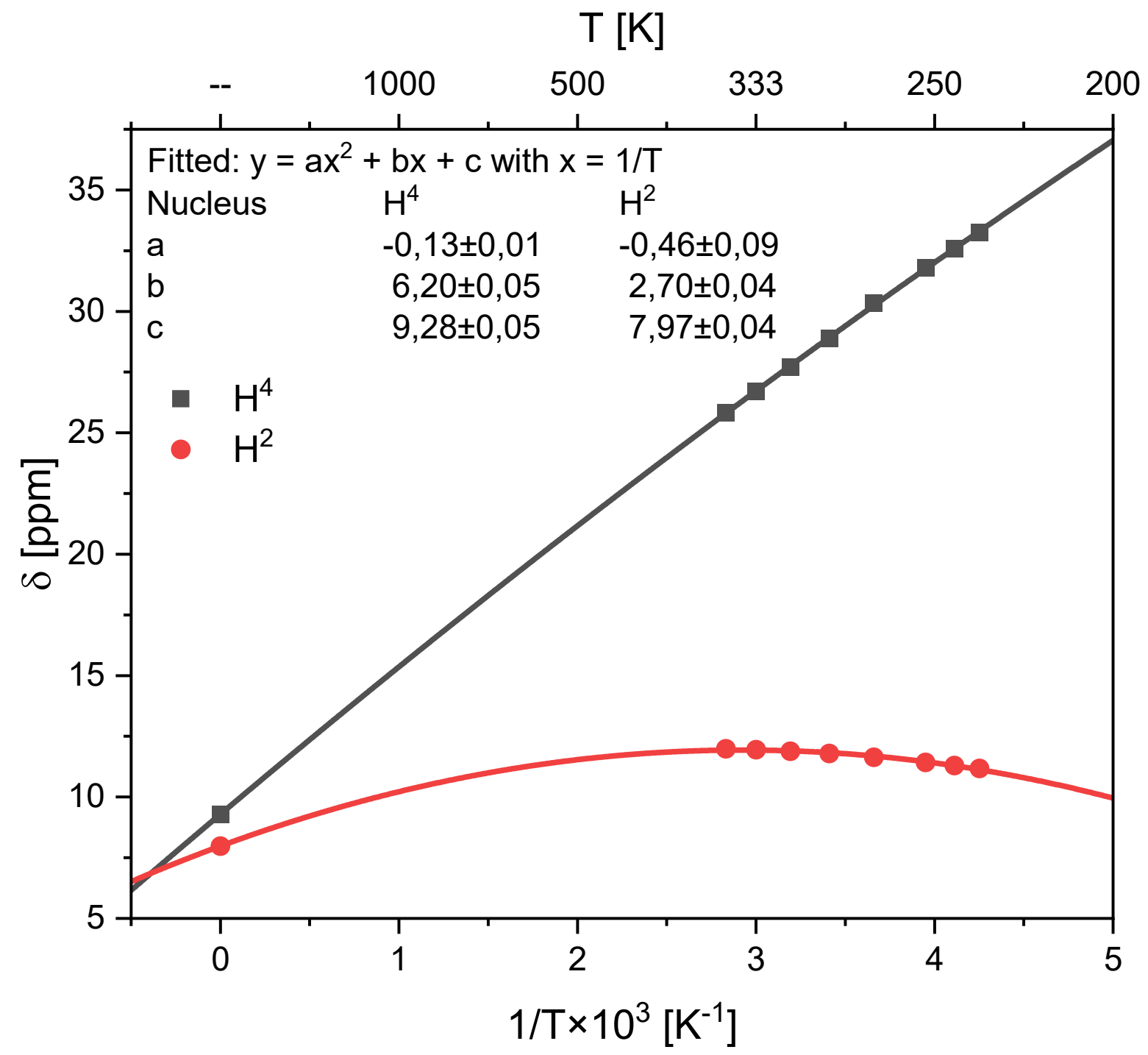

Figure S 55: Plot of the H^4 and H^2 resonances of **3** with added δ_{dia} DFT value at 1/T = 0 against inverse temperature (dots). Second order polynomial fit (lines).

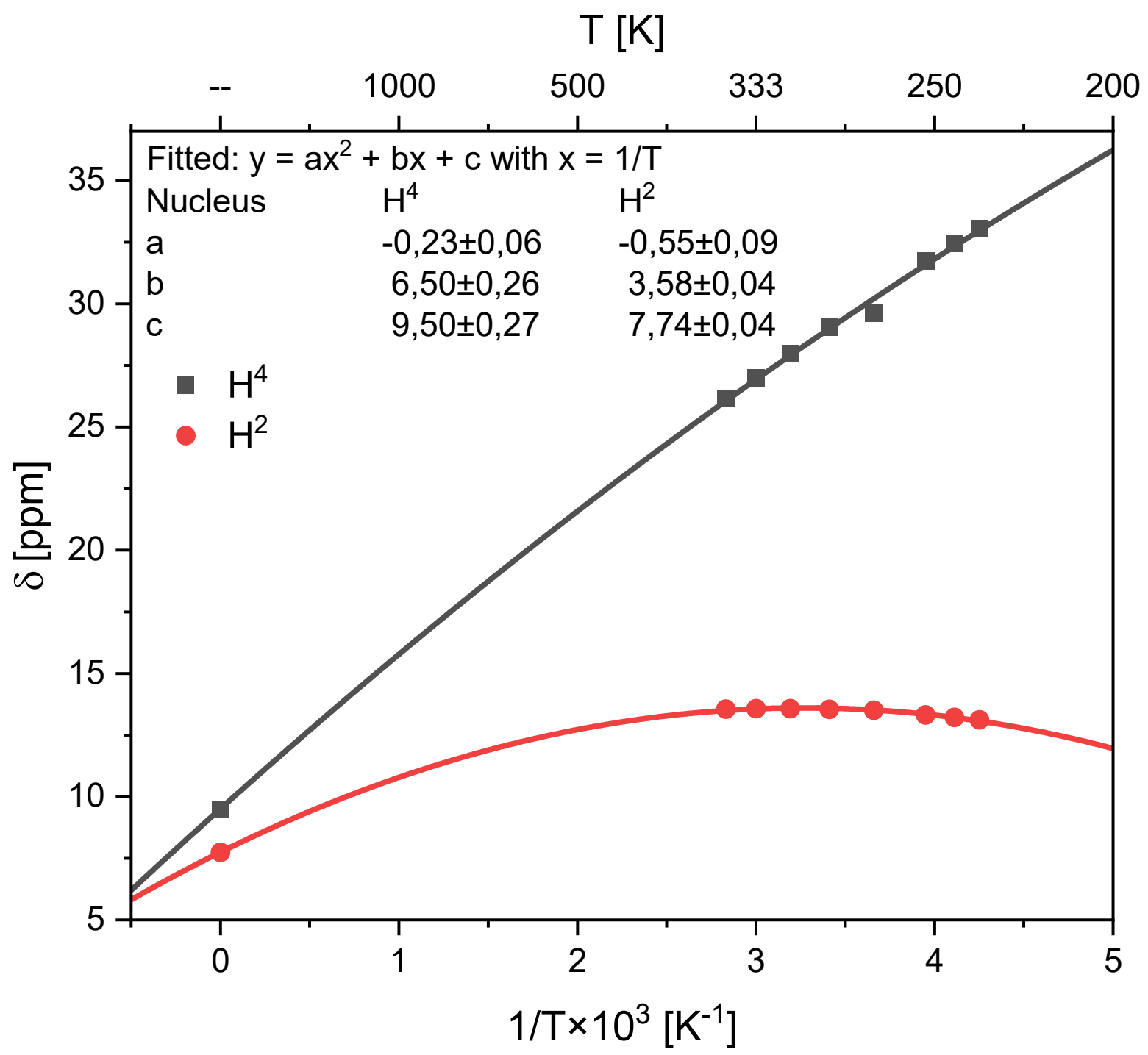

Figure S 56: Plot of the H^4 and H^2 resonances of **2** with added δ_{dia} DFT value at 1/T = 0 against inverse temperature (dots). Second order polynomial fit (lines).

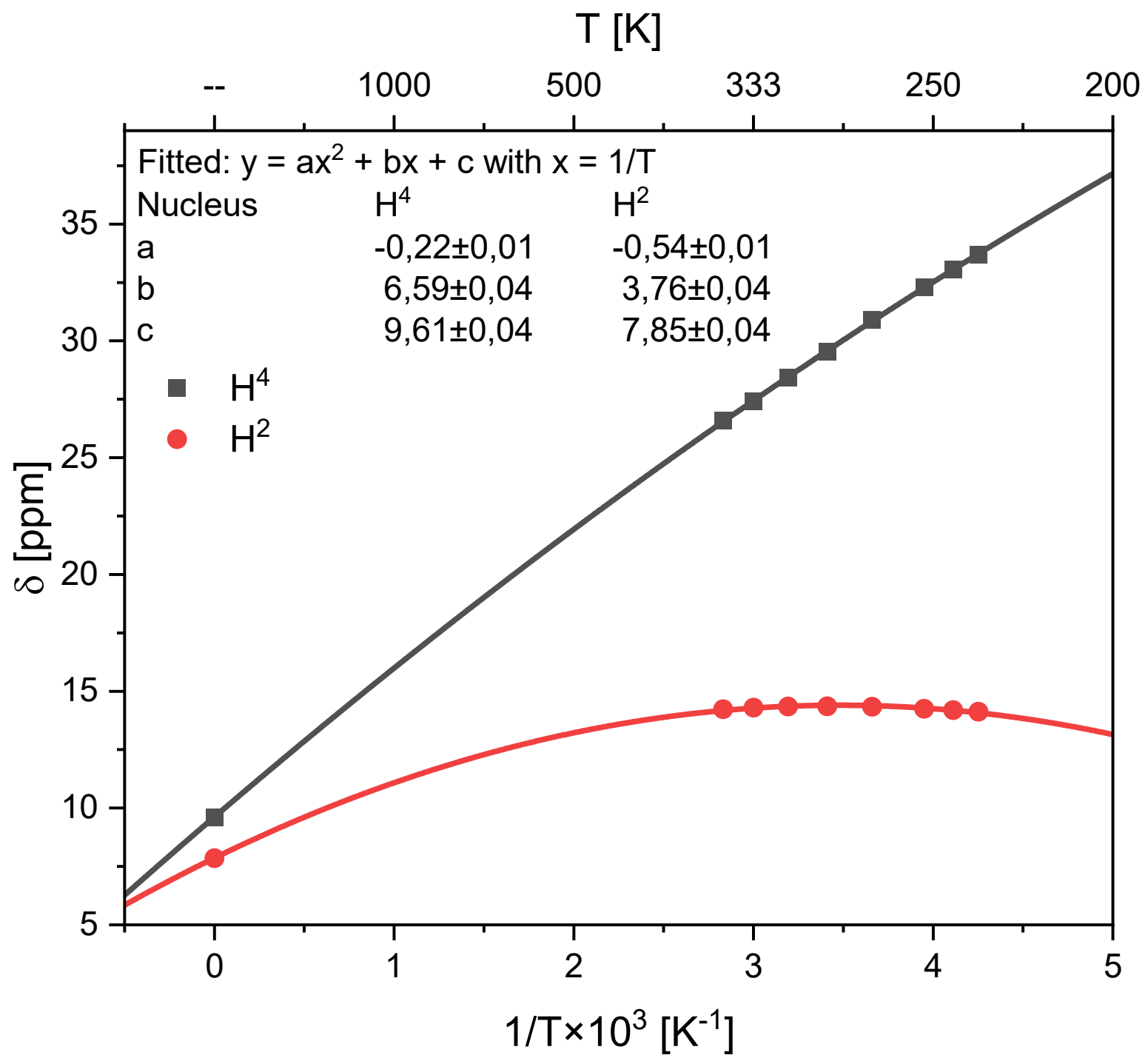

Figure S 57: Plot of the H^4 and H^2 resonances of **4** with added δ_{dia} DFT value at 1/T = 0 against inverse temperature (dots). Second order polynomial fit (lines).

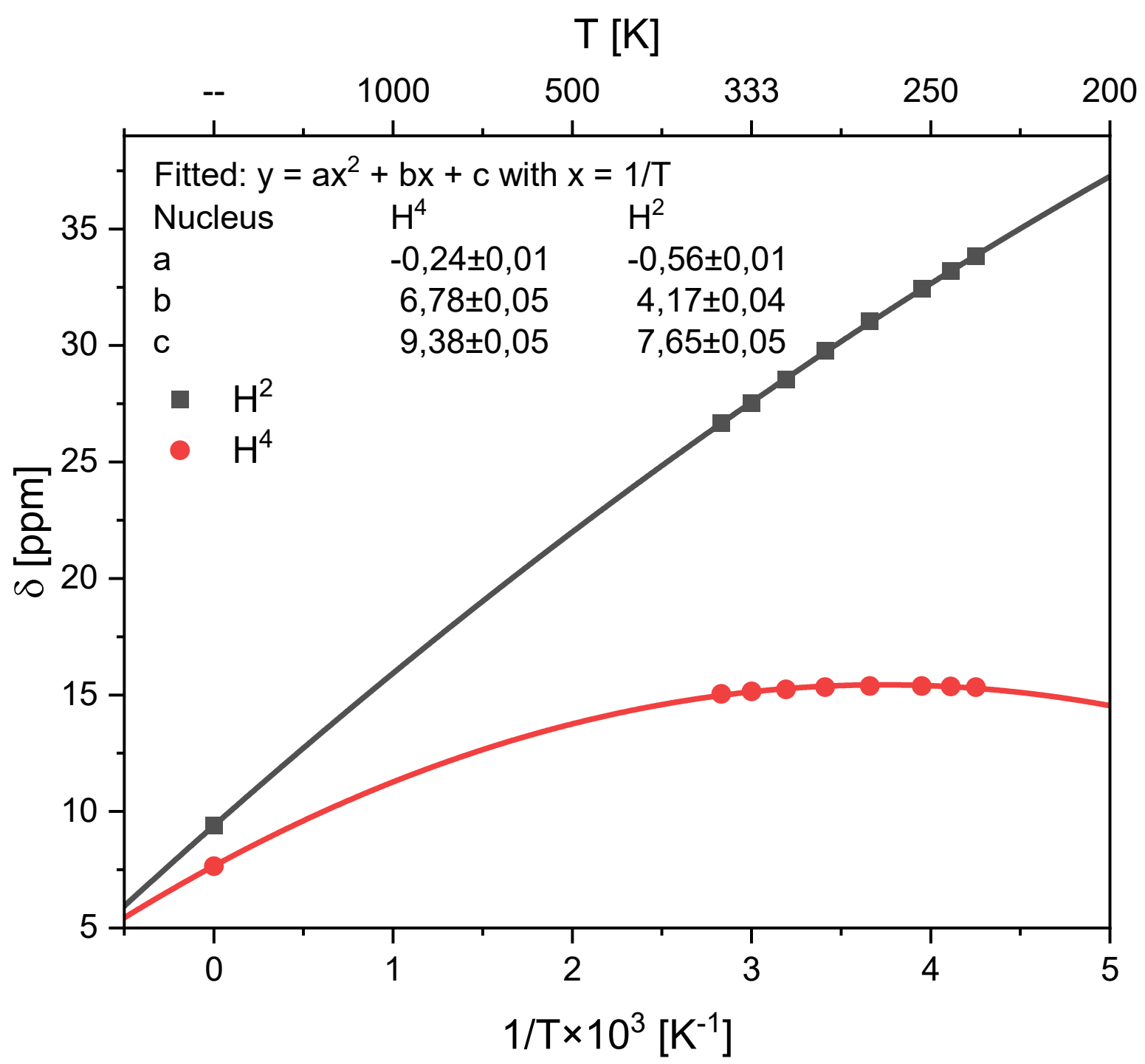

Figure S 58: Plot of the H^4 and H^2 resonances of **5** with added δ_{dia} DFT value at 1/T = 0 against inverse temperature (dots). Second order polynomial fit (lines).

In their publication Autschbach *et. al.* [5]derive the following relation for the fermi contact (δ_{FC}) and pseudo contact (δ_{PC}) term up to a T^{-2} dependence:

$$\delta_{\mathrm{FC}} = C_{\mathrm{iso}} A_{\mathrm{iso}} \left[\frac{2g_{\mathrm{iso}}}{kT} - \frac{2D\Delta g}{3k^2T^2}\right] \qquad \text{(S1)}$$

$$\delta_{\mathrm{PC}} = C_{\mathrm{iso}} \Delta A \left[\frac{4\Delta g}{kT} - \frac{2D(g_{\mathrm{iso}} + \Delta g)}{3k^2T^2}\right] \qquad \text{(S2)}$$

With $C_{\mathrm{iso}} = \frac{\mu_{\mathrm{B}}}{3g_{\mathrm{N}}\mu_{\mathrm{N}}}$, where μ_{B} is the Bohr magneton, g_{N} as the nuclear Lande factor, μ_{N} as the nuclear magneton, k as the Boltzmann constant T as the Temperature and D as the axial zero-field splitting parameter. With the main magnetic axis oriented approximately along the x-axis of the molecular structure ΔA can be calculated as $\Delta A = \frac{1}{3}\left(A_{xx} - \frac{A_{yy}+A_{zz}}{2}\right)$ as described in the original publication from DFT obtained values for the hyperfine coupling tensor $\boldsymbol{A}$ (see Section 6.3) together with A_{iso}. As the deviation by Autschbach *et al.* assume a axial $\boldsymbol{g}$ tensor and with a pseudo axial is acquired from the CASSCF calculation (see Section 6.2) Δg was calculated from $\Delta g = \frac{1}{3}\left(g_3 - \frac{g_1+g_2}{2}\right)$ from CASSCF results together with g_{iso}. Substituting equations S1 and S2 the following relation is obtained for the second order polynomial part:

$$\frac{a}{T^2} = C_{\mathrm{iso}} \left[-\frac{2D\Delta g A_{\mathrm{iso}}}{3k^2T^2} - \frac{2D(g_{\mathrm{iso}} + \Delta g)\Delta A}{3k^2T^2}\right] \qquad \text{(S3)}$$

After simplifying and rearranging the following relation for D is obtained:

$$D = \frac{2a}{C_{\mathrm{iso}}} \left[-\frac{3k^2}{\Delta g A_{\mathrm{iso}}} - \frac{3k^2}{(g_{\mathrm{iso}} + \Delta g)\Delta A}\right] \qquad \text{(S4)}$$

For the resonances H_2 and H_4 of complexes **2-5** the following D values were obtained:

Table S 6: Summary of the parameters used for the estimation of D from equation S4 and obtained D values for the H^2 and H^4 resonances of **2-5**.

Group	g_{iso}	Δg	A_{iso} [MHz]	ΔA [MHz]	a [ppmK*10^3]	D [cm^{-1}]	D_{av} [cm^{-1}]
H^2(Ru-F **3**)	2.216	0.210	0.205	-0.216	-0.461	190	134±80
H^4(Ru-F **3**)			0.252	-0.159	-0.129	77.1	
H^2(Ru-Cl **2**)	2.221	0.207	0.234	-0.218	-0.548	226	182±63
H^4(Ru-Cl **2**)			0.285	-0.162	-0.230	137	
H^2(Ru-Br **4**)	2.228	0.212	0.238	-0.220	-0.541	221	173±66
H^4(Ru-Br **4**)			0.289	-0.164	-0.216	127	
H^2(Ru-I **5**)	2.233	0.217	0.248	-0.222	-0.558	226	181±62
H^4(Ru-I **5**)			0.298	-0.169	-0.242	138	

For each complex the average of the two obtained D values D_{av} was formed and the uncertainty was calculated as the standard deviation of the individual D values from the mean.

6. Computational Section

All DFT and *ab initio* calculations were performed using Orca 6.0.1 or 6.1.0.[6-7]

Geometries were optimized using the unrestricted Kohn-Sham (UKS) r^2scan-3c[8-10] composite method including the atom-pairwise dispersion correction (D4)[11] on the def2-mSVP[12-13]level of theory using effective core potentials for Ru and I and the RI approximation (RIJCOSX)[14-15] in conjunction with the def2/J[16] auxiliary basis set to speed up calculations. The calculated ground state structures were confirmed as energy minima by analytical frequency calculations showing no negative eigenvalues in the Hessian matrix and used as the basis for electronic energies, TD-DFT, NMR, Hyperfine coupling and CASSCF computations.

Single point calculation used for electronic energies were calculated computed on the SMD[17](benzene)/ PWPB95[18]-D3(BJ)[19-22]-def2-TZVP[12] level of theory functional using effective core potentials for Ru and I and the RI approximation (RIJCOSX)[23] in conjunction with the def2/J and def2-TZVP/C[24] auxiliary basis sets to speed up calculations.

For all relativistic DFT computations, the zero-order regular approximation (ZORA)[25-27] was employed In conjunction with the ZORA-def2-TZVP[12] basis set for light atoms and SARC-ZORA-TZVP[28] for ruthenium and iodine. To speed up the calculations, the RI approximation (RIJCOSX) was used in combination with the SARC/J[16] auxiliary basis set.

Time-dependent DFT (TD-DFT) considering 40 excited states were calculated on the ZORA SMD(pentane)/TPSSh[29-30]-D3(BJ) level of theory. The depicted TD-DFT derived UV/Vis spectra in the main manuscript are shown with a peak half-width at half-height value of 0.12 eV. Natural transition orbitals (NTOs)[31] were calculated at the same level of theory.

Diamagnetic NMR shifts using the GIAO method and Hyperfine coupling constats A considering A_{iso} A_{dip} and A_{orb} contributions were calculated on the ZORA ωB97X[32]-D3(BJ) level of theory after a functional screening and comparison to experimentally obtained ^{1}H and ^{13}C shifts (see Section 6.4.1).

For CASSCF calculations, unrestricted natural orbitals (UNOs) were computed by single-point calculations on the ZORA B3LYP[33-34]-D3(BJ)/ level of theory. The CASSCF[35-36] calculations including 6 electrons and 5 orbitals in the active space (CAS(6,5)) were constructed using only the five 4d-orbitals and used for ab initio ligand field theory (AILFT)[37] and were corrected by second-order perturbation theory (SC-NEVPT2).[38-40] To speed up the calculations, the RIJCOSX approximation was used together with SARC/J and AUTOAUX[41] for generating the auxiliary correlation basis set. Spin-orbit coupling was included using the keywords SOCType 3,[42-43] SOCFlags 1,3,3,1 and SOCMaxCenter 4. PictureChange was set to true.

Chemcraft 1.8.[44] was used for the visualization of molecular geometries orbitals and spin densities.

6.1 TD-DFT calculations

6.1.1 Excitations energies and Oscillator Strength of the Halide complexes

Table S 7: Listing of the first 40 excited states of **3** (ZORA SMD(pentane)/TPSSh-D3(BJ)- def2-TZVP).

Excited State	Energy (cm^{-1})	Wavelength (nm)	Oscillator strength
1	6232.3	1604.6	0.000001
2	7663.5	1304.9	0.000070
3	9511.9	1051.3	0.000480
4	13536.5	738.7	0.000477
5	14306.8	699.0	0.000463
6	18071.0	553.4	0.121674
7	18911.8	528.8	0.000410
8	20140.0	496.5	0.000552
9	22003.0	454.5	0.005356
10	23430.1	426.8	0.005330
11	23926.0	418.0	0.000023
12	24089.8	415.1	0.000120
13	25329.5	394.8	0.010682
14	26162.4	382.2	0.003566
15	26485.1	377.6	0.000062
16	26839.2	372.6	0.000455
17	27355.7	365.6	0.000596
18	28869.7	346.4	0.034536
19	29554.2	338.4	0.006698
20	30216.1	330.9	0.002510
21	30456.5	328.3	0.000395
22	30903.4	323.6	0.030813
23	31106.0	321.5	0.023524
24	31553.3	316.9	0.006752
25	31641.2	316.0	0.000019
26	32012.0	312.4	0.057232
27	32569.7	307.0	0.010228
28	33184.1	301.3	0.002319
29	33799.9	295.9	0.012622
30	34053.2	293.7	0.000427
31	34379.2	290.9	0.071393
32	34913.8	286.4	0.005203
33	34997.8	285.7	0.013891
34	35027.6	285.5	0.002420
35	35178.0	284.3	0.006917
36	35403.2	282.5	0.003814
37	35755.6	279.7	0.013113
38	35789.5	279.4	0.006454
39	35855.3	278.9	0.001735
40	36216.8	276.1	0.074551

Table S 8: Listing of the first 40 excited states of **2** (ZORA SMD(pentane)/TPSSh-D3(BJ)- def2-TZVP).

Excited State	Energy (cm^{-1})	Wavelength (nm)	Oscillator strength
1	6837.4	1462.5	0.00000
2	7242.8	1380.7	0.00009
3	9189.4	1088.2	0.00051
4	12055.7	829.5	0.00066
5	14048.1	711.8	0.00050
6	16448.6	608.0	0.14201
7	18185.9	549.9	0.00087
8	18680.7	535.3	0.00033
9	21395.9	467.4	0.00215
10	21616.1	462.6	0.00113
11	23397.0	427.4	0.01711
12	23800.6	420.2	0.00074
13	23841.3	419.4	0.00579
14	24612.2	406.3	0.00041
15	25587.0	390.8	0.00001
16	26304.2	380.2	0.00110
17	28117.6	355.6	0.00059
18	28648.5	349.1	0.00227
19	28786.3	347.4	0.00021
20	28931.7	345.6	0.01500
21	29680.8	336.9	0.00172
22	29988.4	333.5	0.05099
23	30092.5	332.3	0.00178
24	30542.2	327.4	0.00299
25	31063.8	321.9	0.03097
26	31248.4	320.0	0.01137
27	31857.3	313.9	0.02561
28	31923.6	313.2	0.04342
29	32225.6	310.3	0.00582
30	32390.9	308.7	0.00559
31	32714.8	305.7	0.01584
32	33046.0	302.6	0.00008
33	33239.5	300.8	0.00166
34	33449.4	299.0	0.00628
35	33580.5	297.8	0.00256
36	33937.7	294.7	0.00901
37	34038.5	293.8	0.02053
38	34206.1	292.3	0.02081
39	34309.4	291.5	0.03083
40	34633.6	288.7	0.00385

Table S 9: Listing of the first 40 excited states of **4** (ZORA SMD(pentane)/TPSSh-D3(BJ)- def2-TZVP).

Excited State	Energy (cm^{-1})	Wavelength (nm)	Oscillator strength
1	6729.3	1486.0	0.00001
2	7023.2	1423.8	0.00009
3	8980.0	1113.6	0.00049

4	11682.8	856.0	0.00068
5	13895.0	719.7	0.00073
6	16028.5	623.9	0.14778
7	17729.5	564.0	0.00097
8	18490.0	540.8	0.00037
9	20666.7	483.9	0.00002
10	20981.6	476.6	0.00254
11	22987.1	435.0	0.01810
12	23546.3	424.7	0.00465
13	23663.9	422.6	0.00009
14	23992.6	416.8	0.00242
15	24580.7	406.8	0.00001
16	26289.1	380.4	0.00077
17	26931.6	371.3	0.00176
18	27435.6	364.5	0.00878
19	27482.8	363.9	0.01473
20	27674.7	361.3	0.00043
21	28016.4	356.9	0.00077
22	28797.2	347.3	0.01011
23	29622.7	337.6	0.01094
24	29783.6	335.8	0.03347
25	29939.8	334.0	0.00219
26	30131.2	331.9	0.00032
27	30653.2	326.2	0.00005
28	31029.4	322.3	0.03116
29	31216.7	320.3	0.00282
30	31252.4	320.0	0.00300
31	31326.0	319.2	0.01375
32	31402.9	318.4	0.01024
33	31747.8	315.0	0.01925
34	32047.1	312.0	0.05687
35	32705.3	305.8	0.01352
36	32746.7	305.4	0.00554
37	33021.4	302.8	0.00299
38	33787.2	296.0	0.00410
39	33889.3	295.1	0.02152
40	34155.3	292.8	0.04917

Table S 10: Listing of the first 40 excited states of **5** (ZORA SMD(pentane)/TPSSh-D3(BJ)- def2-TZVP).

Excited State	Energy (cm^{-1})	Wavelength (nm)	Oscillator strength
1	6705.2	1491.4	0.00003
2	6807.0	1469.1	0.00007
3	8763.7	1141.1	0.00056
4	11140.8	897.6	0.00077
5	13592.3	735.7	0.00279
6	15213.4	657.3	0.16495
7	16912.6	591.3	0.00115
8	18156.9	550.8	0.00033

9	19365.0	516.4	0.00006
10	20326.6	492.0	0.00173
11	21917.9	456.2	0.00376
12	22215.8	450.1	0.01989
13	23184.6	431.3	0.00000
14	23285.1	429.5	0.01239
15	23402.3	427.3	0.00047
16	23663.4	422.6	0.01070
17	24055.8	415.7	0.00371
18	25573.6	391.0	0.00207
19	26075.2	383.5	0.00037
20	26313.5	380.0	0.00084
21	27534.4	363.2	0.00039
22	27665.2	361.5	0.00047
23	27911.8	358.3	0.00039
24	28258.2	353.9	0.00593
25	28459.6	351.4	0.00069
26	28578.9	349.9	0.00572
27	29190.4	342.6	0.00899
28	29390.1	340.3	0.03645
29	29405.6	340.1	0.02118
30	29724.6	336.4	0.00996
31	30003.1	333.3	0.00193
32	30451.7	328.4	0.00113
33	30813.4	324.5	0.01542
34	31078.8	321.8	0.02208
35	31198.5	320.5	0.00764
36	31646.2	316.0	0.07459
37	32247.4	310.1	0.01612
38	32677.9	306.0	0.00775
39	32793.3	304.9	0.01021
40	33473.6	298.7	0.00489

*6.1.2 NTOs of Transition 6, Spin density and frontier MOs of **2***

Displayed in the table below are the hole and particle NTOs of the 6th transition of complexes **2-5** which is responsible for the absorption in the visible range. Next to it are the ground state spin densities together with the Mulliken reduced spin populations of the Ru d orbitals.

Table S 11: Hole and particle NTOs of transition 6 for complexes **2-5** as well as the ground state spin density and the Mullikan (reduced) spin populations of the Ru atom, as well as all binding ligand atoms and the Ru d orbitals calculated at the ZORA SMD(pentane)/TPSSh-D3(BJ) level of theory.

Compound	Hole-NTO (6)	Particle-NTO (6)	Spin Density	Mulliken Spin Populations	Mulliken Reduced Spin Populations (Ru d orbitals)
Ru-F(**3**)	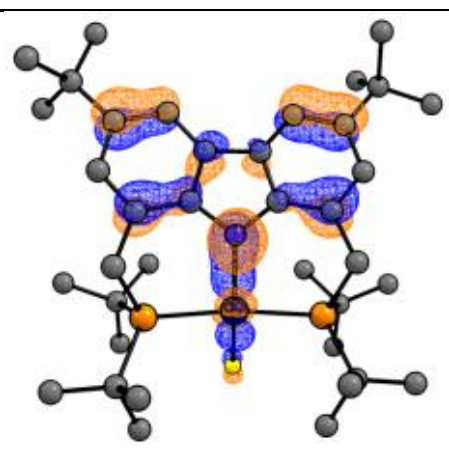	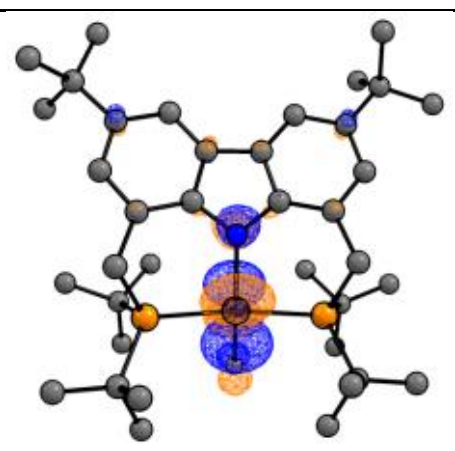	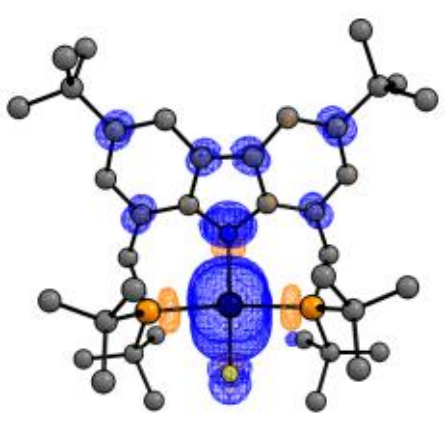	Ru (1.907)<br>P_1(-0.047)<br>P_2(-0.047)<br>N (0.030)<br>F (0.040)	dz^2 (0.813)<br>dxz (0.756)<br>dyz (0.036)<br>dx^2-y^2 (0.069)<br>dxy (0.036)

Ru-Cl(**2**)	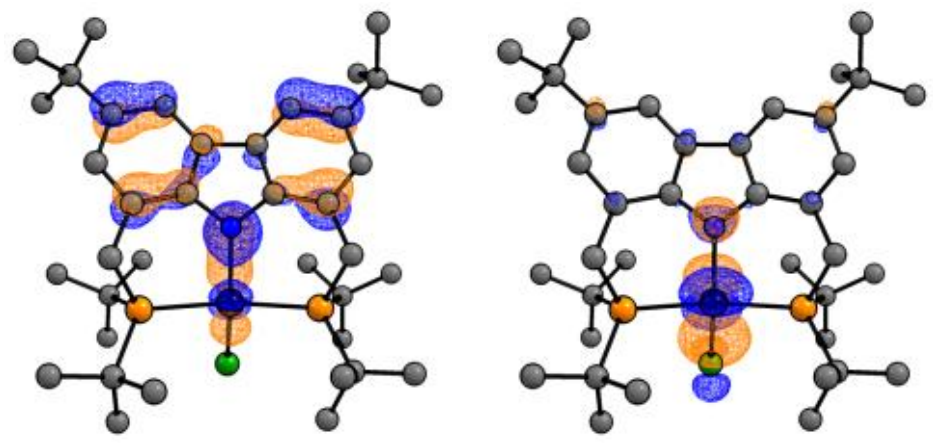	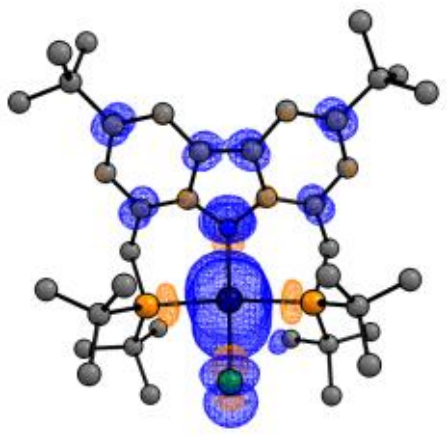	Ru (1.876)<br>P_1(-0.046)<br>P_2(-0.050)<br>N (0.059)<br>Cl (0.038)	dz^2 (0.810)<br>dxz (0.717)<br>dyz (0.049)<br>dx^2-y^2 (0.078)<br>dxy (0.024)
Ru-Br(**4**)	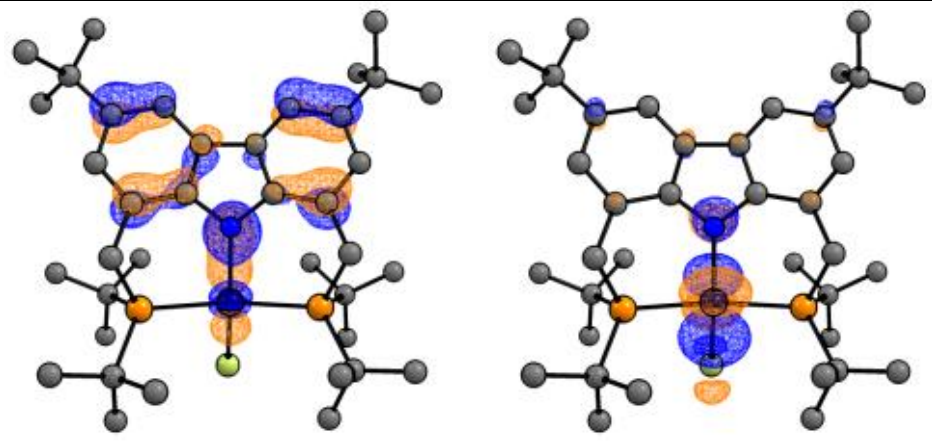	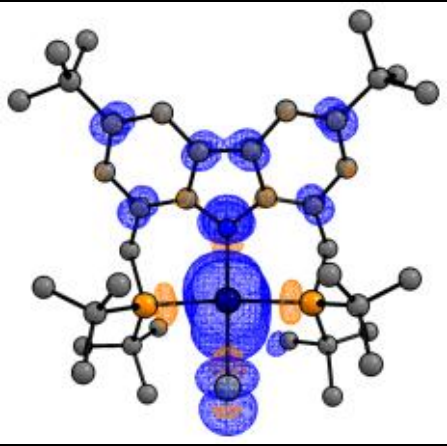	Ru (1.876)<br>P_1(-0.045)<br>P_2(-0.050)<br>N (0.062)<br>Br (0.035)	dz^2 (0.807)<br>dxz (0.710)<br>dyz (0.053)<br>dx^2-y^2 (0.083)<br>dxy (0.025)
Ru-I(**5**)	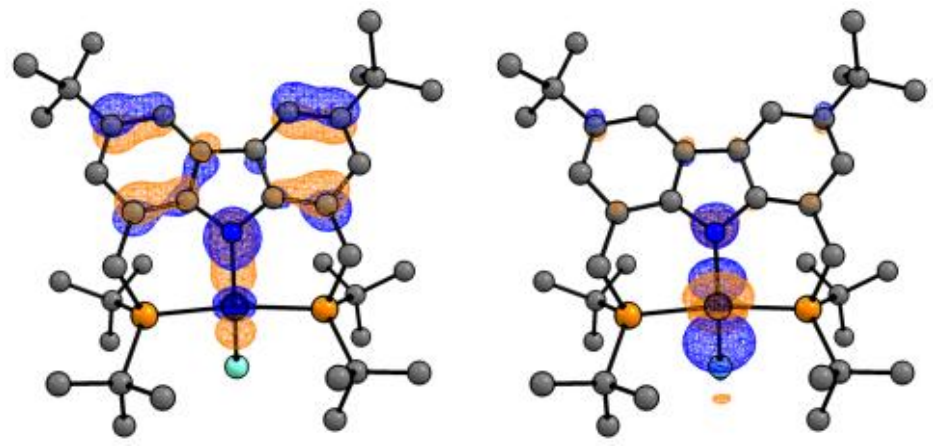	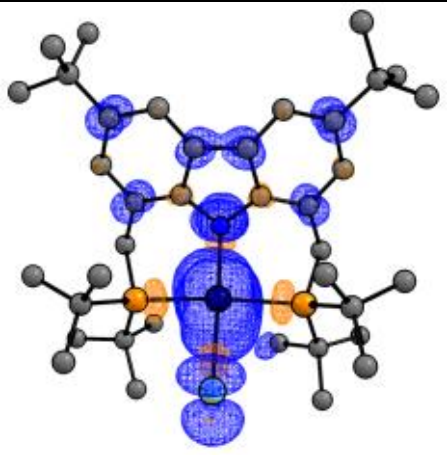	Ru (1.853)<br>P_1(-0.045)<br>P_2(-0.050)<br>N (0.076)<br>I (0.043)	dz^2 (0.786)<br>dxz (0.694)<br>dyz (0.065)<br>dx^2-y^2 (0.095)<br>dxy (0.027)

Table S 12: Selected Frontier MOs of RuCl complex **2** orbitals calculated at the ZORA SMD(pentane)/TPSSh-D3(BJ) level of theory:

MO-Number	α-MOs	β-MOs
188<br>α-Occupation<br>1.00<br>β-Occupation<br>1.00	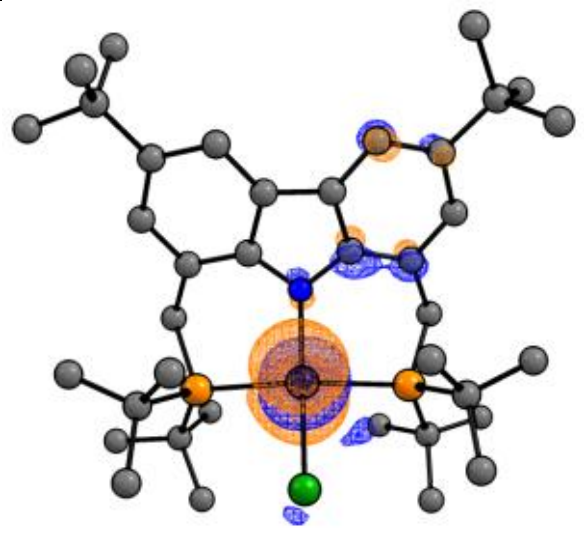	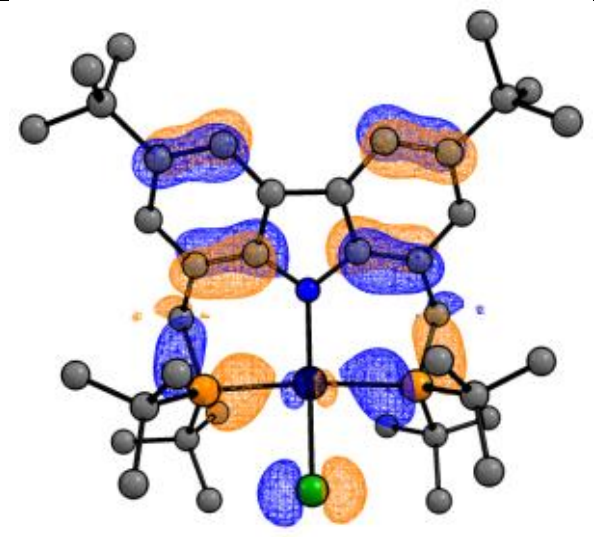
190<br>α-Occupation<br>1.00<br>β-Occupation<br>1.00	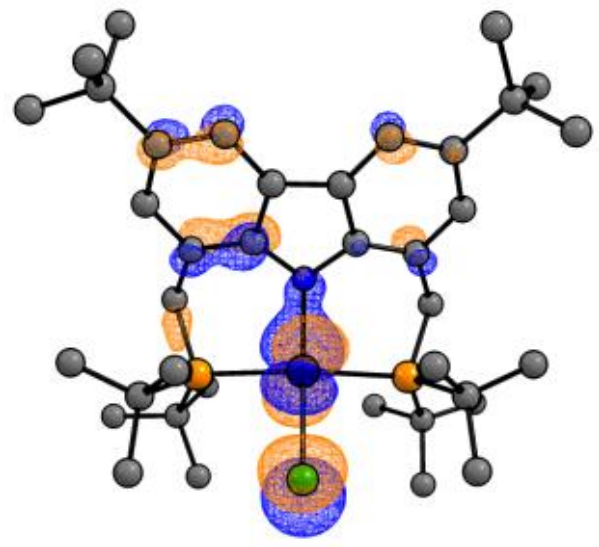	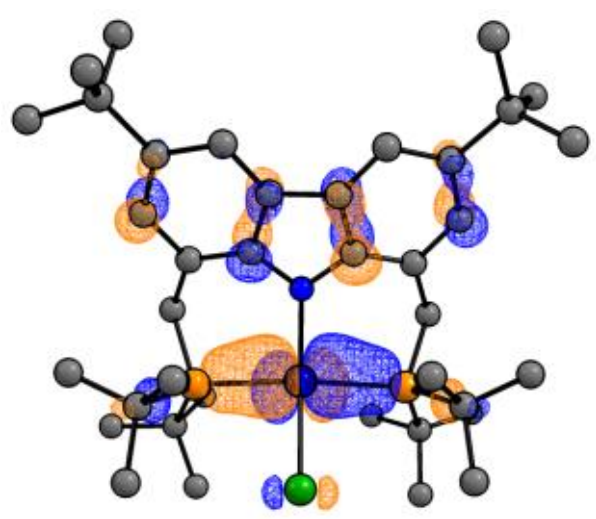
191<br>α-Occupation<br>1.00<br>β-Occupation<br>1.00	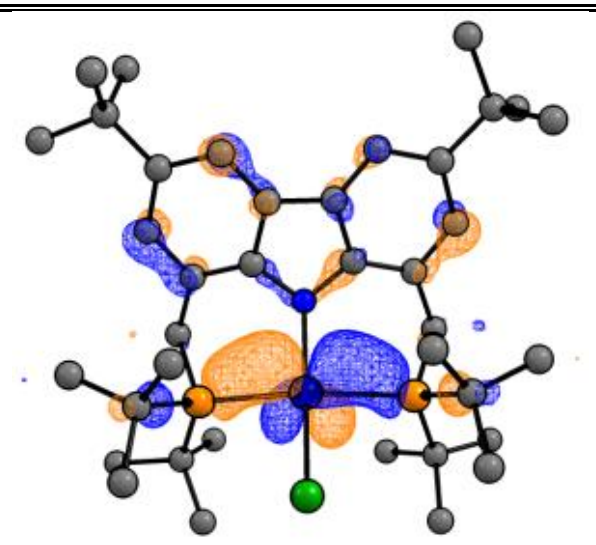	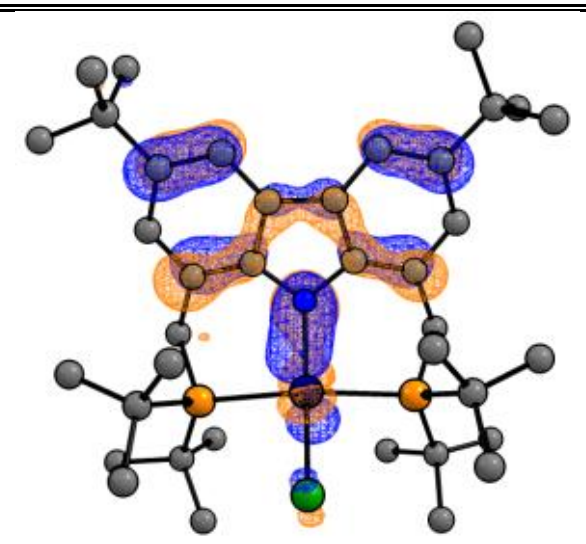

192<br>α-Occupation<br>1.00<br>β-Occupation<br>1.00	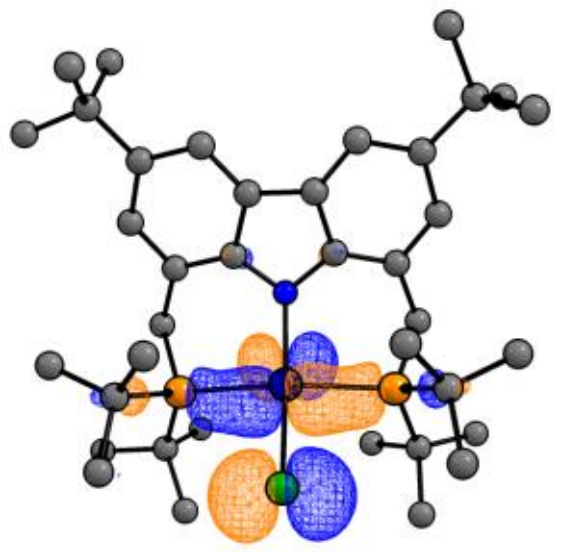	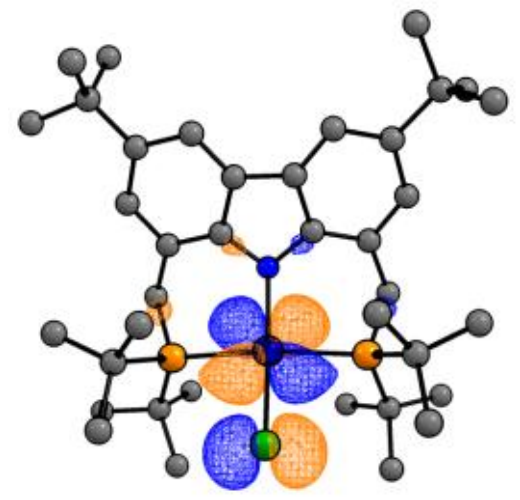
193<br>α-Occupation<br>1.00<br>β-Occupation<br>0.00	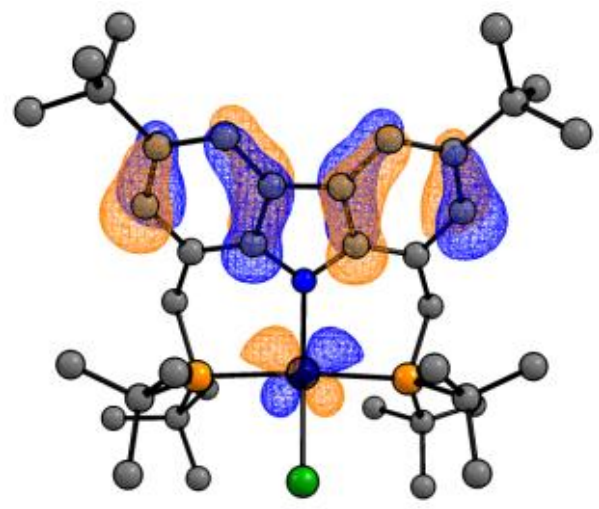	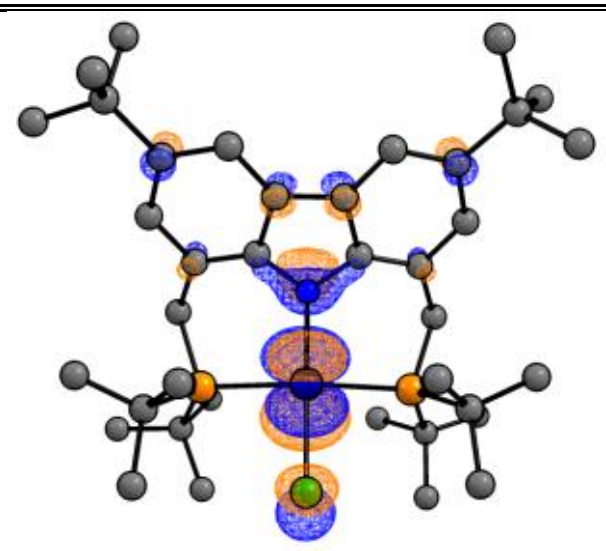
194<br>α-Occupation<br>1.00<br>β-Occupation<br>0.00	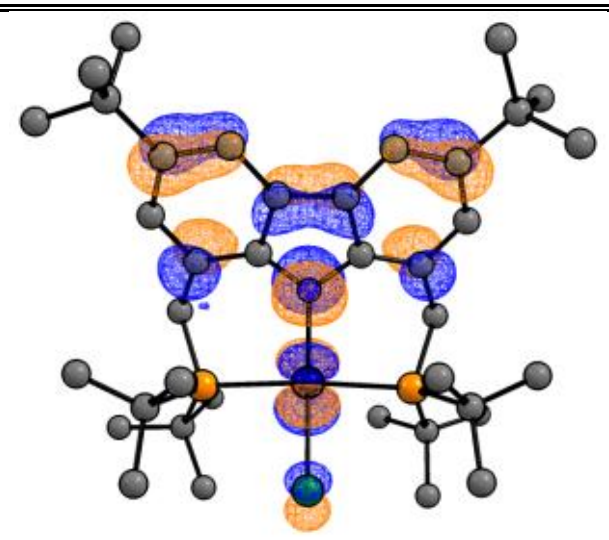	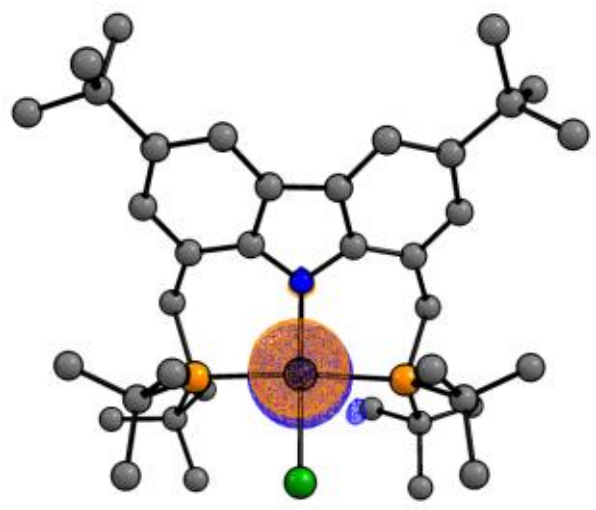
195<br>α-Occupation<br>0.00<br>β-Occupation<br>0.00	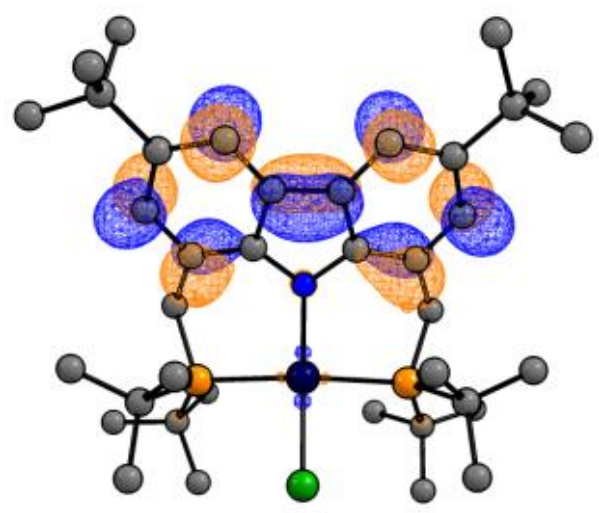	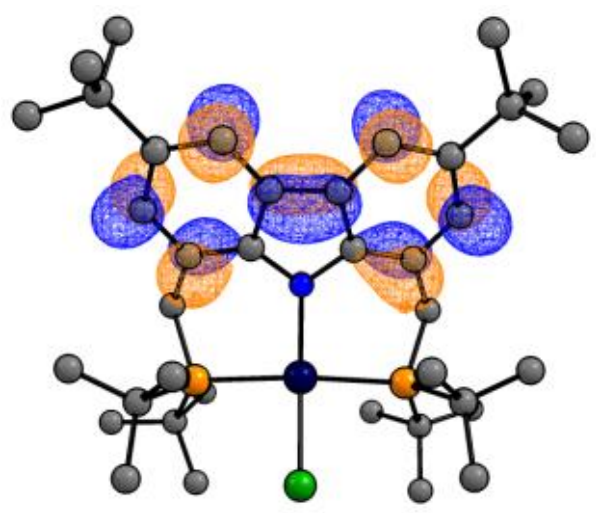
196<br>α-Occupation<br>0.00<br>β-Occupation<br>0.00	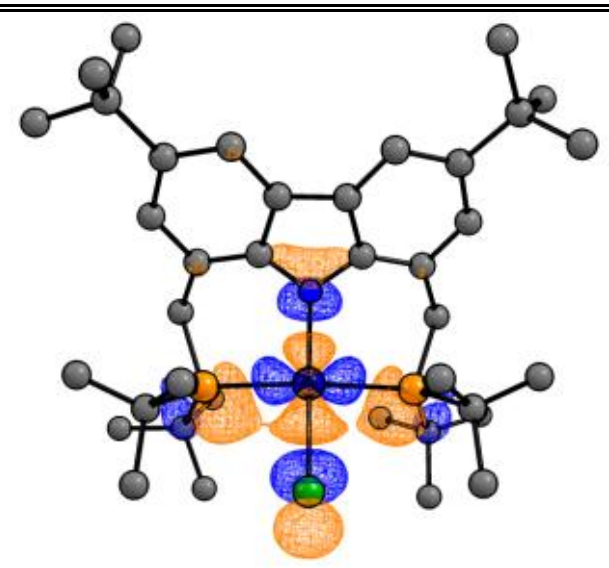	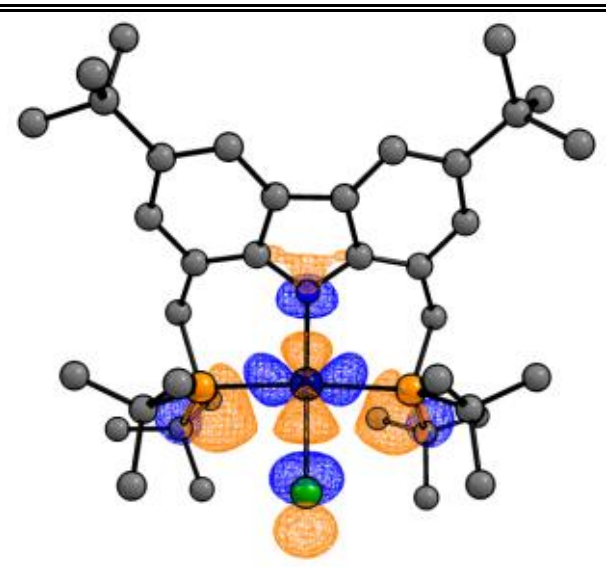

6.2 CASSCF Results

Table S 13: Configurations for the complete active space used for **3** (CAS(6,5), only the first six roots of each multiplicity are listed).

CASSCF STATES FOR BLOCK 0 MULT= 5 NROOTS= 5			
ROOT 0 E = -6891.3639090278 Eh			NEVPT2 Results
0.94865	[2]:	11211	---
0.03791	[0]:	11112	Total Energy Correction : dE = -7.65556538836063
0.00635	[4]:	21111	---
0.00416	[1]:	11121	Reference Energy : E0 = -6891.36390902782205
0.00294	[3]:	12111	---

			Total Energy (E0+dE) : E = -6899.01947441618267
ROOT 1 E =-6891.3425134403 Eh			NEVPT2 Results
0.92320	[4]:	21111	---
0.04426	[3]:	12111	Total Energy Correction : dE = -7.65353736516681
0.02200	[1]:	11121	---
0.00817	[2]:	11211	Reference Energy : E0 = -6891.34251344028326

			Total Energy (E0+dE) : E = -6898.99605080545007
ROOT 2 E =-6891.3375017365 Eh			NEVPT2 Results
0.94989	[0]:	11112	---
0.04116	[2]:	11211	Total Energy Correction : dE = -7.65354700032458
0.00852	[1]:	11121	---
			Reference Energy : E0 = -6891.33750173648787

			Total Energy (E0+dE) : E = -6898.99104873681244
ROOT 3 E =-6891.3164973591 Eh			NEVPT2 Results
0.91946	[3]:	12111	---
0.05654	[4]:	21111	Total Energy Correction : dE = -7.64346532882882
0.02145	[1]:	11121	---
			Reference Energy : E0 = -6891.31649735905012

			Total Energy (E0+dE) : E = -6898.95996268787894
ROOT 4 E =-6891.1976631433 Eh			NEVPT2 Results
0.94386	[1]:	11121	---
0.03307	[3]:	12111	Total Energy Correction : dE = -7.64228234513575
0.01376	[4]:	21111	---
0.00863	[0]:	11112	Reference Energy : E0 = -6891.19766314333447

			Total Energy (E0+dE) : E = -6898.83994548847022
CASSCF STATES FOR BLOCK 1 MULT= 3 NROOTS=45			
ROOT 0 E = -6891.432292499 Eh			NEVPT2 Results
0.90248	[12]:	11202	---
0.02989	[33]:	22101	Total Energy Correction : dE = -7.68516029053717
0.02317	[7]:	10212	---
0.01961	[13]:	11211	Reference Energy : E0 = -6891.43229249901106
0.00856	[27]:	21102	---
0.00293	[10]:	11112	Total Energy (E0+dE) : E = -6899.11745278954822
ROOT 1 E = -6891.4117238851 Eh			NEVPT2 Results
0.89123	[27]:	21102	---
0.02520	[10]:	11112	Total Energy Correction : dE = -7.68241529235092
0.02467	[22]:	20112	---
0.01932	[28]:	21111	Reference Energy : E0 = -6891.41172388511040
0.01817	[17]:	12102	---
0.01023	[12]:	11202	Total Energy (E0+dE) : E = -6899.09413917746133
ROOT 2 E =-6891.4052392806 Eh			NEVPT2 Results
0.47102	[30]:	21201	---
0.42198	[17]:	12102	Total Energy Correction : dE = -7.67989068390943

0.02471	[10]:	11112	--
0.01983	[20]:	12201	Reference Energy : E0 = -6891.40523928062703
0.01597	[24]:	20211	--
0.01083	[27]:	21102	Total Energy (E0+dE) : E = -6899.08512996453646
0.00800	[18]:	12111	--
0.00694	[3]:	02112	
0.00671	[13]:	11211	
0.00436	[31]:	21210	
ROOT 3 E =-6891.3989621102 Eh			NEVPT2 Results
0.86505	[20]:	12201	--
0.05705	[13]:	11211	Total Energy Correction : dE = -7.67593528124235
0.01415	[17]:	12102	--
0.01151	[30]:	21201	Reference Energy : E0 = -6891.39896211016821
0.01139	[10]:	11112	--
0.00890	[5]:	02211	Total Energy (E0+dE) : E = -6899.07489739141056
0.00804	[21]:	12210	--
0.00563	[33]:	22101	
0.00386	[18]:	12111	
0.00318	[24]:	20211	
ROOT 4 E =-6891.375755038 Eh			NEVPT2 Results
0.44537	[17]:	12102	--
0.44113	[30]:	21201	Total Energy Correction : dE = -7.68550411661818
0.02131	[24]:	20211	--
0.02080	[10]:	11112	Reference Energy : E0 = -6891.37575503797052
0.01381	[22]:	20112	--
0.01141	[13]:	11211	Total Energy (E0+dE) : E = -6899.06125915458870
0.01110	[27]:	21102	--
0.00942	[18]:	12111	
0.00803	[3]:	02112	
0.00408	[31]:	21210	
0.00301	[2]:	01221	
ROOT 5 E =-6891.3544684194 Eh			NEVPT2 Results
0.76352	[33]:	22101	--
0.09597	[18]:	12111	Total Energy Correction : dE = -7.67795552188909
0.07851	[28]:	21111	--
0.02751	[12]:	11202	Reference Energy : E0 = -6891.35446841937301
0.00711	[34]:	22110	--
0.00612	[20]:	12201	Total Energy (E0+dE) : E = -6899.03242394126210
0.0052	[25]:	21012	--
0.00369	[11]:	11121	
0.00308	[30]:	21201	
CASSCF STATES FOR BLOCK 2 MULT= 1 NROOTS=50			
ROOT 0 E =-6891.397231947 Eh			NEVPT2 Results
0.65068	[29]:	20202	--
0.20313	[16]:	11202	Total Energy Correction : dE = -7.69978026538229
0.08383	[7]:	02202	--
0.01543	[30]:	20211	Reference Energy : E0 = -6891.39723194702583
0.01322	[39]:	22002	--

0.00414	[35]:	21111	Total Energy (E0+dE) : E = -6899.09701221240812
0.00405	[2]:	01212	---
0.00403	[17]:	11211	
0.00360	[44]:	22200	
ROOT 1 E =-6891.3922739909 Eh			NEVPT2 Results
0.70415	[16]:	11202	---
0.11916	[29]:	20202	Total Energy Correction : dE = -7.69027161023678
0.11460	[7]:	02202	---
0.01792	[11]:	10212	Reference Energy : E0 = -6891.39227399093579
0.01509	[17]:	11211	---
0.01027	[34]:	21102	Total Energy (E0+dE) : E = -6899.08254560117257
0.00304	[22]:	12111	---
0.00271	[2]:	01212	
0.00262	[30]:	20211	
ROOT 2 E =-6891.3761189714 Eh			NEVPT2 Results
0.61036	[34]:	21102	---
0.29228	[24]:	12201	Total Energy Correction : dE = -7.68662135181876
0.02242	[17]:	11211	---
0.01842	[27]:	20112	Reference Energy : E0 = -6891.37611897142051
0.01518	[14]:	11112	---
0.01226	[35]:	21111	Total Energy (E0+dE) : E = -6899.06274032323927
0.00601	[21]:	12102	---
0.00406	[8]:	02211	
0.00358	[29]:	20202	
0.00281	[25]:	12210	
ROOT 3 E =-6891.3701870066 Eh			NEVPT2 Results
0.65305	[7]:	2202	---
0.12308	[29]:	20202	Total Energy Correction : dE = -7.68125130588760
0.07131	[39]:	22002	---
0.03000	[2]:	01212	Reference Energy : E0 = -6891.37018700658791
0.02808	[16]:	11202	---
0.02751	[44]:	22200	Total Energy (E0+dE) : E = -6899.05143831247551
0.01896	[21]:	12102	---
0.01282	[8]:	02211	
0.01033	[11]:	10212	
0.00299	[30]:	20211	
0.00265	[32]:	21012	
ROOT 4 E =-6891.3544758571 Eh			NEVPT2 Results
0.67799	[21]:	12102	---
0.14491	[37]:	21201	Total Energy Correction : dE = -7.68109626623027
0.05914	[24]:	12201	---
0.02673	[5]:	02112	Reference Energy : E0 = -6891.35447585711427
0.02392	[14]:	11112	---
0.01477	[22]:	12111	Total Energy (E0+dE) : E = -6899.03557212334454
0.01063	[7]:	2202	---
0.00814	[39]:	22002	
0.00662	[27]:	20112	
0.00486	[30]:	20211	

0.00437	[34]:	21102	
0.00302	[8]:	02211	

ROOT 5 E =-6891.3501713444 Eh			NEVPT2 Results
0.36524	[37]:	21201	---
0.21442	[24]:	12201	Total Energy Correction : dE = -7.68976019235561
0.16273	[21]:	12102	---
0.12146	[34]:	21102	Reference Energy : E0 = -6891.35017134435839
0.04556	[14]:	11112	---
0.02071	[17]:	11211	Total Energy (E0+dE) : E = -6899.03993153671399
0.01206	[30]:	20211	---
0.00695	[5]:	02112	
0.00636	[16]:	11202	
0.00566	[39]:	22002	
0.00496	[35]:	21111	
0.00486	[38]:	21210	
0.00447	[8]:	02211	
0.00426	[43]:	22110	
0.00409	[7]:	02202	
0.00299	[22]:	12111	
0.00289	[27]:	20112	

Table S 14: SC-NEVPT2 corrected transition energies of the first 10 transitions of **3**.

LOWEST ROOT (ROOT 0, MULT 3) = −6899.117452790 Eh		
ROOT	MULT	eV
0	1	0.556
1	3	0.634
2	3	0.880
1	1	0.950
3	3	1.158
2	1	1.489
4	3	1.529
3	1	1.796
5	1	2.109
6	1	2.213

Table S 15: SC-NEVPT2 corrected AILFT (one electron eigenfunctions) results for **3**, SOC constant 869.1 cm^{-1}.

Orbital	Energy (eV)	Energy(cm^{-1})	dz^2	dxz	dyz	dx^2-y^2	dxy
1 (dyz)	0.00	0.00	-0.07	0.05	0.97	-0.06	-0.20
2 (dz^2)	0.54	4341.10	-0.96	0.22	-0.09	-0.13	0.04
3 (dxy)	0.72	5815.80	0.00	-0.02	0.20	0.09	0.97
4 (dxz)	1.33	10746.40	-0.24	-0.96	0.03	0.14	-0.04
5 (dx^2-y^2)	4.95	39896.80	0.10	-0.17	-0.03	-0.97	0.09

Table S 16: Electronic g-values and zero-field splitting parameters for **3** from effective Hamiltonian.

g_1	g_2	g_3	g_{iso}
1.958	2.056	2.636	2.216

D(cm^{-1}) =	222.7	E/D	0.267

Table S 17: Magnetic susceptibility tensor (cm^3 K mol^{-1}) for **3** at 298 K (SC-NEVPT2 corrected).

2.1978	-0.0725	0.1298
-0.0725	0.5905	0.0189
0.1298	0.0189	1.0573

Table S 18: Configurations for the complete active space used for **2** (CAS(6,5), only the first six roots of each multiplicity are listed).

CASSCF STATES FOR BLOCK 0 MULT= 5 NROOTS= 5			
ROOT 0 E =-7253.5318205159 Eh			NEVPT2 Results
0.93846	[2]:	11211	---
0.02918	[0]:	11112	Total Energy Correction : dE = -7.59630186005870
0.01893	[4]:	21111	---
0.00863	[1]:	11121	Reference Energy : E0 = -7253.53182051586555
0.00480	[3]:	12111	---
			Total Energy (E0+dE) : E = -7261.12812237592425
ROOT 1 E =-7253.5132222716 Eh			NEVPT2 Results
0.95018	[0]:	11112	---
0.03641	[2]:	11211	Total Energy Correction : dE = -7.59538166531456
0.00965	[1]:	11121	---
			Reference Energy : E0 = -7253.51322227162200

			Total Energy (E0+dE) : E = -7261.10860393693656
ROOT 2 E =-7253.5121881340 Eh			NEVPT2 Results
0.91239	[4]:	21111	---
0.05145	[3]:	12111	Total Energy Correction : dE = -7.59385344360453
0.02065	[2]:	11211	---
0.00847	[0]:	11112	Reference Energy : E0 = -7253.51218813397190
0.00704	[1]:	11121	---
			Total Energy (E0+dE) : E = -7261.10604157757643
ROOT 3 E =-7253.4936049400 Eh			NEVPT2 Results
0.91029	[3]:	12111	---
0.06272	[4]:	21111	Total Energy Correction : dE = -7.58425835817434
0.02312	[1]:	11121	---
			Reference Energy : E0 = -7253.49360494000666

			Total Energy (E0+dE) : E = -7261.07786329818100
ROOT 4 E =-7253.3834820783 Eh			NEVPT2 Results
0.95155	[1]:	11121	---
0.03195	[3]:	12111	Total Energy Correction : dE = -7.58199066360248
0.00979	[0]:	11112	---
0.00372	[4]:	21111	Reference Energy : E0 = -7253.38348207830131
0.00299	[2]:	11211	---
			Total Energy (E0+dE) : E = -7260.96547274190380
CASSCF STATES FOR BLOCK 1 MULT= 3 NROOTS=45			

ROOT 0 E =-7253.5923768868 Eh			NEVPT2 Results
0.87259	[12]:	11202	---
0.03094	[33]:	22101	Total Energy Correction : dE = -7.63014394830770
0.02475	[27]:	21102	---
0.02283	[7]:	10212	Reference Energy : E0 = -7253.59237688678786
0.0225	[13]:	11211	---
0.00865	[10]:	11112	Total Energy (E0+dE) : E = -7261.22252083509557
0.00505	[20]:	12201	---
0.00467	[17]:	12102	

ROOT 1 E =-7253.5733352425 Eh			NEVPT2 Results
0.83438	[27]:	21102	---
0.05213	[17]:	12102	Total Energy Correction : dE = -7.62663200962288
0.02835	[12]:	11202	---
0.02562	[22]:	20112	Reference Energy : E0 = -7253.57333524248952
0.01905	[28]:	21111	---
0.01343	[30]:	21201	Total Energy (E0+dE) : E = -7261.19996725211240
0.00776	[10]:	11112	---
0.00536	[24]:	20211	
0.00312	[33]:	22101	
0.00281	[25]:	21012	

ROOT 2 E =-7253.5681000767 Eh			NEVPT2 Results
0.52871	[17]:	12102	---
0.27770	[30]:	21201	Total Energy Correction : dE = -7.61994925744420
0.06812	[27]:	21102	---
0.04648	[20]:	12201	Reference Energy : E0 = -7253.56810007667991
0.03456	[10]:	11112	---
0.00987	[18]:	12111	Total Energy (E0+dE) : E = -7261.18804933412412
0.00697	[24]:	20211	---
0.00647	[28]:	21111	
0.00541	[13]:	11211	
0.00337	[3]:	02112	
0.00295	[31]:	21210	

ROOT 3 E =-7253.5609722887 Eh			NEVPT2 Results
0.80988	[20]:	12201	---
0.05791	[13]:	11211	Total Energy Correction : dE = -7.61879995226627
0.04172	[30]:	21201	---
0.01759	[17]:	12102	Reference Energy : E0 = -7253.56097228872568
0.01668	[33]:	22101	---
0.01337	[10]:	11112	Total Energy (E0+dE) : E = -7261.17977224099195
0.01054	[18]:	12111	---
0.00828	[21]:	12210	
0.00577	[12]:	11202	
0.00552	[24]:	20211	
0.00274	[28]:	21111	

ROOT 4 E =-7253.5392372721 Eh			NEVPT2 Results
0.56664	[30]:	21201	---
0.30315	[17]:	12102	Total Energy Correction : dE = -7.63195174104476
0.02567	[24]:	20211	---

0.02079	[20]:	12201	Reference Energy : E0 = -7253.53923727209167
0.01807	[13]:	11211	---
0.01478	[22]:	20112	Total Energy (E0+dE) : E = -7261.17118901313643
0.01061	[10]:	11112	---
0.00964	[18]:	12111	
0.00832	[28]:	21111	
0.00572	[31]:	21210	
0.00364	[2]:	01221	
0.00285	[3]:	02112	
ROOT 5 E =-7253.5158019112 Eh			NEVPT2 Results
0.77468	[33]:	22101	---
0.08018	[28]:	21111	Total Energy Correction : dE = -7.62145101834267
0.05884	[18]:	12111	---
0.03224	[12]:	11202	Reference Energy : E0 = -7253.51580191124413
0.016	[20]:	12201	---
0.00797	[34]:	22110	Total Energy (E0+dE) : E = -7261.13725292958679
0.00546	[25]:	21012	---
0.00476	[30]:	21201	
0.00446	[27]:	21102	
0.00295	[32]:	22011	
CASSCF STATES FOR BLOCK 2 MULT= 1 NROOTS=50			
ROOT 0 E =-7253.5561450348 Eh			NEVPT2 Results
0.51289	[29]:	20202	---
0.30479	[16]:	11202	Total Energy Correction : dE = -7.64228793277744
0.10354	[7]:	2202	---
0.01449	[30]:	20211	Reference Energy : E0 = -7253.55614503482047
0.01087	[39]:	22002	---
0.01038	[2]:	01212	Total Energy (E0+dE) : E = -7261.19843296759791
0.00757	[17]:	11211	---
0.00656	[21]:	12102	
0.00512	[27]:	20112	
0.00431	[14]:	11112	
0.00419	[35]:	21111	
0.00352	[37]:	21201	
ROOT 1 E =-7253.5542002986 Eh			NEVPT2 Results
0.58773	[16]:	11202	---
0.16398	[29]:	20202	Total Energy Correction : dE = -7.63489763871348
0.15482	[7]:	02202	---
0.02466	[34]:	21102	Reference Energy : E0 = -7253.55420029856941
0.02434	[11]:	10212	---
0.01420	[17]:	11211	Total Energy (E0+dE) : E = -7261.18909793728290
0.00497	[14]:	11112	---
0.00462	[30]:	20211	
0.00421	[27]:	20112	
0.00357	[8]:	02211	
0.00295	[22]:	12111	
ROOT 2 E =-7253.5378691209 Eh			NEVPT2 Results
0.61709	[34]:	21102	---

0.27374	[24]:	12201	Total Energy Correction : dE = -7.63098810164865
0.02244	[17]:	11211	---
0.01893	[27]:	20112	Reference Energy : E0 = -7253.53786912088617
0.01588	[21]:	12102	---
0.01396	[35]:	21111	Total Energy (E0+dE) : E = -7261.16885722253483
0.00977	[29]:	20202	---
0.00601	[16]:	11202	
0.00487	[14]:	11112	
0.00306	[32]:	21012	
0.00291	[25]:	12210	

ROOT 3 E =-7253.5366350105 Eh			NEVPT2 Results
0.56221	[7]:	02202	---
0.17367	[29]:	20202	Total Energy Correction : dE = -7.62655167148569
0.07718	[39]:	22002	---
0.04438	[21]:	12102	Reference Energy : E0 = -7253.53663501045958
0.02644	[2]:	01212	---
0.02489	[44]:	22200	Total Energy (E0+dE) : E = -7261.16318668194526
0.01808	[11]:	10212	---
0.01478	[16]:	11202	
0.01219	[8]:	02211	
0.00898	[34]:	21102	
0.00556	[5]:	02112	
0.00555	[30]:	20211	
0.00494	[27]:	20112	
0.00377	[37]:	21201	
0.00334	[14]:	11112	
0.00266	[32]:	21012	

ROOT 4 E =-7253.5213695735 Eh			NEVPT2 Results
0.74136	[21]:	12102	---
0.04189	[14]:	11112	Total Energy Correction : dE = -7.61962789411882
0.03945	[37]:	21201	---
0.03481	[7]:	02202	Reference Energy : E0 = -7253.52136957349012
0.03416	[39]:	22002	---
0.02298	[34]:	21102	Total Energy (E0+dE) : E = -7261.14099746760894
0.01664	[22]:	12111	---
0.01487	[5]:	02112	
0.01002	[16]:	11202	
0.00939	[17]:	11211	
0.00748	[27]:	20112	
0.00561	[19]:	12012	
0.00337	[32]:	21012	

ROOT 5 E =-7253.5123184876 Eh			NEVPT2 Results
0.53583	[24]:	12201	---
0.1911	[34]:	21102	Total Energy Correction : dE = -7.62814503642767
0.05027	[21]:	12102	---
0.04905	[37]:	21201	Reference Energy : E0 = -7253.51231848759198
0.03952	[17]:	11211	---
0.01776	[14]:	11112	Total Energy (E0+dE) : E = -7261.14046352401965

0.01773	[42]:	22101	---
0.01558	[16]:	11202	
0.01406	[27]:	20112	
0.01007	[35]:	21111	
0.00972	[30]:	20211	
0.00785	[22]:	12111	
0.00730	[29]:	20202	
0.00554	[25]:	12210	
0.00524	[5]:	02112	
0.00478	[40]:	22011	
0.00289	[8]:	02211	
0.00275	[32]:	21012	

Table S 19: SC-NEVPT2 corrected transition energies of the first 10 transitions of **2**.

LOWEST ROOT (ROOT 0, MULT 3) = −7261.222520835 Eh		
ROOT	MULT	eV
1	3	0.614
0	1	0.655
1	1	0.909
2	3	0.938
3	3	1.163
4	3	1.397
2	1	1.460
3	1	1.615
6	1	2.029
4	1	2.218

Table S 20: SC-NEVPT2 corrected AILFT (one electron eigenfunctions) results for **2**, SOC constant 854.99 cm^{-1}.

Orbital	Energy (eV)	Energy(cm^{-1})	dz^2	dxz	dyz	dx^2-y^2	dxy
1 (dyz)	0.00	0.00	-0.13	0.07	0.97	-0.09	-0.18
2 (dz^2)	0.47	3800.30	-0.95	0.25	-0.15	-0.08	0.01
3 (dxy)	0.51	4103.10	-0.03	-0.02	0.19	0.09	0.98
4 (dxz)	1.12	9005.80	0.27	0.95	-0.03	-0.15	0.05
5 (dx^2-y^2)	4.52	36447.50	-0.05	0.17	0.06	0.98	-0.10

Table S 21: Electronic g-values and zero-field splitting parameters for **2** from effective Hamiltonian.

g_1	g_2	g_3	g_{iso}
1.963	2.067	2.635	2.221
D(cm^{-1}) =	216.6	E/D	0.255

Table S 22: Magnetic susceptibility tensor (cm^3 K mol^{-1}) for **2** at 298 K (SC-NEVPT2 corrected).

2.1729	-0.0785	0.1577
-0.0785	0.6067	0.0362
0.1577	0.0362	0.0362

Table S 23: Configurations for the complete active space used for **4** (CAS(6,5), only the first six roots of each multiplicity are listed).

CASSCF STATES FOR BLOCK 0 MULT= 5 NROOTS= 5

ROOT 0: E= -9414.3823142750 Eh			NEVPT2 Results
0.93685	[2]:	11211	---
0.02631	[0]:	11112	Total Energy Correction : dE = -7.70829643551770
0.02327	[4]:	21111	---
0.00949	[1]:	11121	Reference Energy : E0 = -9414.38231427504252
0.00408	[3]:	12111	---
			Total Energy (E0+dE) : E = -9422.09061071056021
ROOT 0: E= -9414.3653513073 Eh			NEVPT2 Results
0.95843	[0]:	11112	---
0.03229	[2]:	11211	Total Energy Correction : dE = -7.70747085125004
0.00659	[1]:	11121	---
			Reference Energy : E0 = -9414.36535130726952

			Total Energy (E0+dE) : E = -9422.07282215851956
ROOT 2: E= -9414.3633519575 Eh			NEVPT2 Results
0.90828	[4]:	21111	---
0.05494	[3]:	12111	Total Energy Correction : dE = -7.70588293328365
0.02579	[2]:	11211	---
0.00556	[0]:	11112	Reference Energy : E0 = -9414.36335195746324
0.00543	[1]:	11121	---
			Total Energy (E0+dE) : E = -9422.06923489074688
ROOT 3: E= -9414.3465281085 Eh			NEVPT2 Results
0.90850	[3]:	12111	---
0.06507	[4]:	21111	Total Energy Correction : dE = -7.69613023553029
0.02290	[1]:	11121	---
0.00267	[0]:	11112	Reference Energy : E0 = -9414.34652810846455

			Total Energy (E0+dE) : E = -9422.04265834399484
ROOT 4: E= -9414.2401307694 Eh			NEVPT2 Results
0.95559	[1]:	11121	---
0.03067	[3]:	12111	Total Energy Correction : dE = -7.69374551060173
0.00703	[0]:	11112	---
0.00421	[2]:	11211	Reference Energy : E0 = -9414.24013076941810
0.00250	[4]:	21111	---
			Total Energy (E0+dE) : E = -9421.93387628001983

CASSCF STATES FOR BLOCK 1 MULT= 3 NROOTS=45

ROOT 0: E= -9414.4386614975 Eh			NEVPT2 Results
0.86862	[12]:	11202	---
0.03154	[33]:	22101	Total Energy Correction : dE = -7.74318327414585
0.03041	[27]:	21102	---
0.02211	[7]:	10212	Reference Energy : E0 = -9414.43866149753921
0.01684	[13]:	11211	---
0.01099	[10]:	11112	Total Energy (E0+dE) : E = -9422.18184477168506
0.00666	[20]:	12201	---
0.00401	[17]:	12102	
ROOT 1: E= -9414.4203272453 Eh			NEVPT2 Results

0.81414	[27]:	21102	---
0.06621	[17]:	12102	Total Energy Correction : dE = -7.73962989002575
0.03329	[12]:	11202	---
0.02473	[22]:	20112	Reference Energy : E0 = -9414.42032724533419
0.02044	[30]:	21201	---
0.01311	[28]:	21111	Total Energy (E0+dE) : E = -9422.15995713535995
0.00632	[24]:	20211	---
0.00543	[10]:	11112	
0.00386	[25]:	21012	
0.00384	[33]:	22101	
ROOT 2: E= -9414.4157566645 Eh			NEVPT2 Results
0.54767	[17]:	12102	---
0.23329	[30]:	21201	Total Energy Correction : dE = -7.73195701535042
0.08749	[27]:	21102	---
0.05594	[20]:	12201	Reference Energy : E0 = -9414.41575666445715
0.03649	[10]:	11112	---
0.00679	[18]:	12111	Total Energy (E0+dE) : E = -9422.14771367980757
0.00659	[28]:	21111	---
0.00654	[13]:	11211	
0.00492	[24]:	20211	
0.00273	[3]:	02112	
ROOT 3: E= -9414.4082468086 Eh			NEVPT2 Results
0.79068	[20]:	12201	---
0.05649	[13]:	11211	Total Energy Correction : dE = -7.73137730988492
0.04993	[30]:	21201	---
0.02122	[17]:	12102	Reference Energy : E0 = -9414.40824680861624
0.02049	[33]:	22101	---
0.01367	[10]:	11112	Total Energy (E0+dE) : E = -9422.13962411850116
0.01319	[18]:	12111	---
0.00738	[12]:	11202	
0.00590	[24]:	20211	
0.00576	[21]:	12210	
0.00373	[28]:	21111	
0.00281	[27]:	21102	
ROOT 4: E= -9414.3865612481 Eh			NEVPT2 Results
0.58697	[30]:	21201	---
0.27389	[17]:	12102	Total Energy Correction : dE = -7.74497601935946
0.02721	[20]:	12201	---
0.02575	[24]:	20211	Reference Energy : E0 = -9414.38656124810768
0.02373	[13]:	11211	---
0.01550	[22]:	20112	Total Energy (E0+dE) : E = -9422.13153726746714
0.01211	[28]:	21111	---
0.00807	[10]:	11112	
0.00747	[18]:	12111	
0.00419	[31]:	21210	
0.00379	[2]:	01221	
0.0026	[8]:	10221	
ROOT 5: E= -9414.3632742993 Eh			NEVPT2 Results

0.77487	[33]:	22101	---
0.07903	[28]:	21111	Total Energy Correction : dE = -7.73411392297021
0.05452	[18]:	12111	---
0.0344	[12]:	11202	Reference Energy : E0 = -9414.36327429930861
0.01971	[20]:	12201	---
0.00572	[25]:	21012	Total Energy (E0+dE) : E = -9422.09738822227882
0.00571	[34]:	22110	---
0.00473	[27]:	21102	
0.00426	[32]:	22011	
0.00418	[30]:	21201	
0.0027	[4]:	02121	

CASSCF STATES FOR BLOCK 2 MULT= 1 NROOTS=50

ROOT 0: E= -9414.4021823346 Eh			NEVPT2 Results
0.45301	[29]:	20202	---
0.36415	[16]:	11202	Total Energy Correction : dE = -7.75454002377046
0.10351	[7]:	02202	---
0.01251	[2]:	01212	Reference Energy : E0 = -9414.40218233464839
0.00995	[39]:	22002	---
0.00971	[30]:	20211	Total Energy (E0+dE) : E = -9422.15672235841885
0.00760	[21]:	12102	---
0.00678	[17]:	11211	
0.00651	[14]:	11112	
0.00552	[27]:	20112	
0.00452	[35]:	21111	
0.00386	[37]:	21201	

ROOT 1: E= -9414.4008721978 Eh			NEVPT2 Results
0.52815	[16]:	11202	---
0.19335	[29]:	20202	Total Energy Correction : dE = -7.74785437399623
0.18220	[7]:	02202	---
0.02650	[34]:	21102	Reference Energy : E0 = -9414.40087219775342
0.02516	[11]:	10212	---
0.0093	[17]:	11211	Total Energy (E0+dE) : E = -9422.14872657174965
0.00585	[27]:	20112	---
0.00560	[14]:	11112	
0.00418	[30]:	20211	
0.00307	[8]:	02211	
0.00304	[22]:	12111	

ROOT 2: E= -9414.3848265925 Eh			NEVPT2 Results
0.61527	[34]:	21102	---
0.27178	[24]:	12201	Total Energy Correction : dE = -7.74396046577385
0.02199	[17]:	11211	---
0.02139	[21]:	12102	Reference Energy : E0 = -9414.38482659245710
0.01760	[27]:	20112	---
0.01236	[29]:	20202	Total Energy (E0+dE) : E = -9422.12878705823096
0.01016	[35]:	21111	---
0.00828	[16]:	11202	
0.00385	[32]:	21012	

0.00370	[14]:	11112	
ROOT 3: E= -9414.3845000896 Eh			NEVPT2 Results
0.53034	[7]:	02202	---
0.19405	[29]:	20202	Total Energy Correction : dE = -7.73974756968346
0.08021	[39]:	22002	---
0.05026	[21]:	12102	Reference Energy : E0 = -9414.38450008957443
0.02444	[44]:	22200	---
0.02386	[2]:	01212	Total Energy (E0+dE) : E = -9422.12424765925789
0.02099	[11]:	10212	---
0.01468	[34]:	21102	
0.01190	[16]:	11202	
0.00810	[8]:	2211	
0.00754	[27]:	20112	
0.00660	[5]:	02112	
0.00503	[30]:	20211	
0.00499	[37]:	21201	
0.00358	[14]:	11112	
ROOT 4: E= -9414.3699095822 Eh			NEVPT2 Results
0.72654	[21]:	12102	---
0.04219	[7]:	02202	Total Energy Correction : dE = -7.73215239961246
0.04132	[39]:	22002	---
0.04095	[14]:	11112	Reference Energy : E0 = -9414.36990958220122
0.03573	[37]:	21201	---
0.02941	[34]:	21102	Total Energy (E0+dE) : E = -9422.10206198181368
0.01228	[5]:	02112	---
0.01153	[22]:	12111	
0.01078	[17]:	11211	
0.01040	[16]:	11202	
0.00777	[27]:	20112	
0.00691	[19]:	12012	
0.00421	[32]:	21012	
0.00252	[35]:	21111	
ROOT 5: E= -9414.3595824362 Eh			NEVPT2 Results
0.52726	[24]:	12201	---
0.17808	[34]:	21102	Total Energy Correction : dE = -7.74067938102235
0.05941	[21]:	12102	---
0.05495	[37]:	21201	Reference Energy : E0 = -9414.35958243619280
0.0387	[17]:	11211	---
0.02114	[42]:	22101	Total Energy (E0+dE) : E = -9422.10026181721514
0.01807	[16]:	11202	---
0.01678	[14]:	11112	
0.01382	[27]:	20112	
0.01109	[30]:	20211	
0.00962	[22]:	12111	
0.0087	[35]:	21111	
0.00707	[29]:	20202	
0.00557	[5]:	02112	
0.00543	[40]:	22011	

0.00388	[25]:	12210
0.00317	[32]:	21012

Table S 24: SC-NEVPT2 corrected transition energies of the first 10 transitions of **4**.

LOWEST ROOT (ROOT 0, MULT 3) = −9422.181844772 Eh		
ROOT	MULT	eV
1	3	0.596
0	1	0.684
1	1	0.901
2	3	0.929
3	3	1.149
4	3	1.369
2	1	1.444
3	1	1.567
6	1	2.039
4	1	2.171

Table S 25: SC-NEVPT2 corrected AILFT (one electron eigenfunctions) results for **4**, SOC constant 837.64 cm^{-1}.

Orbital	Energy (eV)	Energy(cm^{-1})	dz^2	dxz	dyz	dx^2-y^2	dxy
1 (dyz)	0.00	0.00	-0.14	0.06	0.97	-0.10	-0.18
2 (dz^2)	0.45	3618.10	-0.95	0.27	-0.17	-0.07	-0.03
3 (dxy)	0.46	3732.50	-0.07	-0.02	0.18	0.07	0.98
4 (dxz)	1.06	8552.80	-0.28	-0.95	0.02	0.15	-0.06
5 (dx^2-y^2)	4.36	35158.90	0.04	-0.17	-0.07	-0.98	0.08

Table S 26: Electronic g-values and zero-field splitting parameters for **4** from effective Hamiltonian.

g_1	g_2	g_3	g_{iso}
1.964	2.068	2.653	2.229
D(cm^{-1}) =	215.0	E/D	0.267

Table S 27: Magnetic susceptibility tensor (cm^3 K mol^{-1}) for **4** at 298 K (SC-NEVPT2 corrected).

2.2064	-0.0666	0.1791
-0.0666	0.6093	0.0404
0.1791	0.0404	1.0865

Table S 28: Configurations for the complete active space used for **5** (CAS(6,5), only the first six roots of each multiplicity are listed).

CASSCF STATES FOR BLOCK 0 MULT= 5 NROOTS= 5			
ROOT 0: E= -14013.2097526587 Eh			NEVPT2 Results
0.91345	[2]:	11211	---
0.04275	[4]:	21111	Total Energy Correction : dE = -7.68297647294457
0.03066	[0]:	11112	---
0.01219	[1]:	11121	Reference Energy : E0 = -14013.20975265868219

			Total Energy (E0+dE) : E = -14020.89272913162677

ROOT 1: E= -14013.1953467898 Eh			NEVPT2 Results
0.96091	[0]:	11112	---
0.03240	[2]:	11211	Total Energy Correction : dE = -7.68240610867724
0.00638	[3]:	12111	---
			Reference Energy : E0 = -14013.19534678982382

			Total Energy (E0+dE) : E = -14020.87775289850106
ROOT 2: E= -14013.1915586997 Eh			NEVPT2 Results
0.86252	[4]:	21111	---
0.08722	[3]:	12111	Total Energy Correction : dE = -7.68074469550811
0.04270	[2]:	11211	---
0.00392	[1]:	11121	Reference Energy : E0 = -14013.19155869966198
0.00364	[0]:	11112	---
			Total Energy (E0+dE) : E = -14020.87230339517009
ROOT 3: E= -14013.1768282362 Eh			NEVPT2 Results
0.86757	[3]:	12111	---
0.09406	[4]:	21111	Total Energy Correction : dE = -7.67050052335253
0.03301	[1]:	11121	---
0.00459	[0]:	11112	Reference Energy : E0 = -14013.17682823616087

			Total Energy (E0+dE) : E = -14020.84732875951340
ROOT 4: E= -14013.0762495034 Eh			NEVPT2 Results
0.95056	[1]:	11121	---
0.03787	[3]:	12111	Total Energy Correction : dE = -7.66829050409069
0.01069	[2]:	11211	---
			Reference Energy : E0 = -14013.07624950337777

			Total Energy (E0+dE) : E = -14020.74454000746846
CASSCF STATES FOR BLOCK 1 MULT= 3 NROOTS=45			
ROOT 0: E= -14013.2606358101 Eh			NEVPT2 Results
0.83892	[12]:	11202	---
0.05343	[27]:	21102	Total Energy Correction : dE = -7.71971648017825
0.02782	[33]:	22101	---
0.02685	[7]:	10212	Reference Energy : E0 = -14013.26063581014569
0.02234	[10]:	11112	---
0.01503	[20]:	12201	Total Energy (E0+dE) : E = -14020.98035229032394
0.00386	[18]:	12111	---
ROOT 1: E= -14013.2432757850 Eh			NEVPT2 Results
0.71501	[27]:	21102	---
0.12360	[17]:	12102	Total Energy Correction : dE = -7.71619851584910
0.04847	[12]:	11202	---
0.04141	[30]:	21201	Reference Energy : E0 = -14013.24327578500197
0.02843	[22]:	20112	---
0.00924	[33]:	22101	Total Energy (E0+dE) : E = -14020.95947430085107
0.00903	[25]:	21012	---
0.00901	[24]:	20211	
0.00369	[10]:	11112	

ROOT 2: E= -14013.2394039614 Eh			NEVPT2 Results
0.50197	[17]:	12102	---
0.15620	[27]:	21102	Total Energy Correction : dE = -7.70702034774877
0.15609	[30]:	21201	---
0.09619	[20]:	12201	Reference Energy : E0 = -14013.23940396141916
0.04895	[10]:	11112	---
0.01248	[13]:	11211	Total Energy (E0+dE) : E = -14020.94642430916792
0.00534	[22]:	20112	---
0.00485	[15]:	12012	
0.00406	[28]:	21111	
0.00375	[18]:	12111	
0.00291	[24]:	20211	
ROOT 3: E= -14013.2311642818 Eh			NEVPT2 Results
0.68511	[20]:	12201	---
0.08870	[30]:	21201	Total Energy Correction : dE = -7.70686229531384
0.07118	[13]:	11211	---
0.03819	[17]:	12102	Reference Energy : E0 = -14013.23116428183857
0.03337	[33]:	22101	---
0.02159	[18]:	12111	Total Energy (E0+dE) : E = -14020.93802657715241
0.01385	[12]:	11202	---
0.01080	[27]:	21102	
0.00939	[10]:	11112	
0.00918	[24]:	20211	
0.00880	[28]:	21111	
0.00283	[32]:	22011	
ROOT 4: E= -14013.2098819242 Eh			NEVPT2 Results
0.56458	[30]:	21201	---
0.25330	[17]:	12102	Total Energy Correction : dE = -7.72114176836112
0.05104	[20]:	12201	---
0.03148	[24]:	20211	Reference Energy : E0 = -14013.20988192415280
0.03109	[13]:	11211	---
0.02650	[28]:	21111	Total Energy (E0+dE) : E = -14020.93102369251392
0.01489	[22]:	20112	---
0.00720	[10]:	11112	
0.00400	[8]:	10221	
0.00350	[2]:	01221	
ROOT 5: E= -14013.1864273849 Eh			NEVPT2 Results
0.74402	[33]:	22101	---
0.10449	[28]:	21111	Total Energy Correction : dE = -7.70985936542638
0.03839	[18]:	12111	---
0.03834	[12]:	11202	Reference Energy : E0 = -14013.18642738494054
0.03490	[20]:	12201	---
0.01102	[32]:	22011	Total Energy (E0+dE) : E = -14020.89628675036693
0.00560	[25]:	21012	---
0.00488	[13]:	11211	
0.00453	[27]:	21102	
0.00384	[4]:	02121	

CASSCF STATES FOR BLOCK 2 MULT= 1 NROOTS=50

ROOT 0: E= -14013.2242461860 Eh

0.52094	[16]:	11202
0.31682	[29]:	20202
0.06313	[7]:	02202
0.01942	[34]:	21102
0.01780	[14]:	11112
0.01630	[2]:	01212
0.00793	[39]:	22002
0.00618	[27]:	20112
0.00584	[37]:	21201
0.00491	[24]:	12201
0.00400	[35]:	21111
0.00398	[21]:	12102

NEVPT2 Results

Total Energy Correction : dE = -7.73059176221068

Reference Energy : E0 = -14013.22424618602417

Total Energy (E0+dE) : E = -14020.95483794823485

ROOT 1: E= -14013.2236298662 Eh

0.34359	[16]:	11202
0.28423	[29]:	20202
0.25032	[7]:	02202
0.03032	[11]:	10212
0.0222	[34]:	21102
0.01925	[21]:	12102
0.01530	[27]:	20112
0.00708	[14]:	11112
0.00652	[5]:	02112
0.00612	[37]:	21201

NEVPT2 Results

Total Energy Correction : dE = -7.72431521978251

Reference Energy : E0 = -14013.22362986624285

Total Energy (E0+dE) : E = -14020.94794508602536

ROOT 2: E= -14013.2084749336 Eh

0.45347	[7]:	02202
0.22853	[29]:	20202
0.09070	[21]:	12102
0.07511	[39]:	22002
0.02898	[11]:	10212
0.02492	[2]:	01212
0.02246	[44]:	22200
0.01405	[16]:	11202
0.01307	[27]:	20112
0.01229	[5]:	02112
0.01024	[37]:	21201
0.00718	[14]:	11112
0.00353	[32]:	21012
0.00281	[35]:	21111

NEVPT2 Results

Total Energy Correction : dE = -7.71616929330048

Reference Energy : E0 = -14013.20847493357905

Total Energy (E0+dE) : E = -14020.92464422687954

ROOT 3: E= -14013.2078391724 Eh

0.61642	[34]:	21102
0.25793	[24]:	12201
0.02685	[21]:	12102
0.02496	[27]:	20112
0.02378	[17]:	11211
0.01460	[16]:	11202
0.00782	[22]:	12111
0.00690	[7]:	02202

NEVPT2 Results

Total Energy Correction : dE = -7.72046023903204

Reference Energy : E0 = -14013.20783917237713

Total Energy (E0+dE) : E = -14020.92829941140917

0.00497	[32]:	21012	
ROOT 4: E= -14013.1946707000 Eh			NEVPT2 Results
0.62578	[21]:	12102	---
0.07009	[7]:	02202	Total Energy Correction : dE = -7.70773827873927
0.06388	[39]:	22002	---
0.05908	[34]:	21102	Reference Energy : E0 = -14013.19467070003157
0.04683	[14]:	11112	---
0.03833	[37]:	21201	Total Energy (E0+dE) : E = -14020.90240897877084
0.01461	[17]:	11211	---
0.01218	[19]:	12012	
0.01113	[27]:	20112	
0.00961	[16]:	11202	
0.00960	[32]:	21012	
0.00560	[2]:	01212	
0.00541	[35]:	21111	
0.00504	[24]:	12201	
0.00487	[5]:	02112	
0.00463	[30]:	20211	
ROOT 5: E= -14013.1827645697 Eh			NEVPT2 Results
0.45721	[24]:	12201	---
0.11989	[34]:	21102	Total Energy Correction : dE = -7.71610470873748
0.0956	[21]:	12102	---
0.09492	[37]:	21201	Reference Energy : E0 = -14013.18276456969215
0.04955	[17]:	11211	---
0.03068	[42]:	22101	Total Energy (E0+dE) : E = -14020.89886927842963
0.02626	[16]:	11202	---
0.02082	[30]:	20211	
0.01557	[27]:	20112	
0.01328	[22]:	12111	
0.01066	[7]:	02202	
0.01054	[35]:	21111	
0.00768	[39]:	22002	
0.00695	[5]:	02112	
0.00693	[14]:	11112	
0.00638	[40]:	22011	
0.0054	[29]:	20202	
0.00297	[32]:	21012	
0.00291	[19]:	12012	
0.00277	[11]:	10212	
0.0027	[10]:	10122	

Table S 29: SC-NEVPT2 corrected transition energies of the first 10 transitions of **5**.

LOWEST ROOT (ROOT 0, MULT 3) = −14020.980352290 Eh		
ROOT	MULT	eV
1	3	0.568
0	1	0.694
1	1	0.882
2	3	0.923

3	3	1.152
4	3	1.342
3	1	1.416
2	1	1.516
6	1	2.047
4	1	2.121

Table S 30: SC-NEVPT2 corrected AILFT (one electron eigenfunctions) results for **5**, SOC constant 808.69 cm^{-1}.

Orbital	Energy (eV)	Energy(cm^{-1})	dz^2	dxz	dyz	dx^2-y^2	dxy
1 (dyz)	0.00	0.00	-0.19	0.03	0.95	-0.11	-0.21
2 (dxy)	0.39	3142.00	0.01	0.08	-0.21	0.02	-0.97
3 (dz^2)	0.42	3385.30	0.92	-0.34	0.19	0.06	-0.06
4 (dxz)	1.00	8053.50	0.34	0.92	0.04	-0.18	0.07
5 (dx^2-y^2)	4.13	33328.60	-0.01	0.19	0.10	0.98	0.01

Table S 31: Electronic g-values and zero-field splitting parameters for **5** from effective Hamiltonian.

g_1	g_2	g_3	g_{iso}
1.965	2.067	2.668	2.233
D(cm^{-1}) =	214.0	E/D	0.280

Table S 32: Magnetic susceptibility tensor (cm^3 K mol^{-1}) for **5** at 298 K (SC-NEVPT2 corrected).

2.2064	-0.0666	0.1791
-0.0666	0.6093	0.0404
0.1791	0.0404	1.0865

6.3 Computation of HCF Tensors

Table S 33: Components of ^{1}H, ^{13}C, ^{19}F and ^{31}P hyperfine coupling tensors for **3** computed with ZORA ωB97X-D3(BJ)- def2-TZVP in units of ppm Å^{-3}. Atom number refers to the position of the atom in the cartesian coordinates of the molecule.

Atom No.	A_{xx}	A_{xy}	A_{xz}	A_{yx}	A_{yy}	A_{yz}	A_{zx}	A_{zy}	A_{zz}
6	-746.5	1048.2	92.4	806.3	1105.4	318.2	58.1	358.4	-929.2
7	994.5	2509.8	1255.6	1975.4	2801.6	1674.1	1030.5	1800.2	810.6
10	386.1	824.0	-191.7	661.7	479.8	-125.2	-164.5	-112.6	-214.5
13	768.1	287.7	-406.1	245.9	84.1	-101.4	-322.9	-101.4	-57.5
19	677.8	-350.8	-394.2	-306.0	127.6	141.4	-312.2	133.9	-75.7
22	184.5	-797.9	-162.1	-636.2	644.7	137.9	-140.4	128.0	-242.7
25	657.5	-2195.9	1111.7	-1672.1	2938.4	-1746.8	860.5	-1841.8	905.6
26	-1031.6	-830.2	88.4	-596.2	1092.4	-402.2	40.4	-426.4	-1012.5
29	-3.0	275.0	-220.5	219.7	-12.6	-217.2	-174.6	-219.1	-16.1
30	131.9	203.5	-124.7	164.9	136.4	-112.6	-102.0	-112.3	19.6
31	-46.5	299.1	-150.8	232.8	134.0	-162.4	-121.0	-161.3	-193.7
33	75.7	323.2	-26.8	257.8	66.6	14.2	-17.5	20.3	-212.5
34	197.3	215.8	-51.1	177.1	102.0	-29.7	-39.1	-26.8	-31.1
35	235.1	280.3	23.6	229.2	-60.0	0.7	33.0	5.6	-170.4
37	286.4	142.0	-155.0	109.4	-135.7	-33.8	-124.9	-32.7	-143.5
38	128.0	148.7	-129.3	119.4	-73.1	-53.2	-105.2	-52.7	-88.6

39	190.6	155.0	-258.2	120.7	-139.8	-66.0	-212.5	-67.1	-58.8
42	-4.4	-201.0	-71.2	-161.6	80.3	70.8	-56.0	68.6	-120.3
43	-77.1	-276.6	-109.0	-214.6	208.0	141.5	-86.1	139.5	-170.1
44	7.1	-301.3	-39.9	-238.8	169.4	23.7	-29.6	19.2	-188.2
46	215.3	-293.1	4.2	-244.4	-43.3	2.8	14.3	-1.8	-162.7
47	290.5	-183.2	-122.4	-147.1	-99.0	41.8	-96.3	38.2	-175.5
48	286.2	-192.3	-73.5	-160.4	142.9	47.5	-57.6	44.8	44.1
50	183.5	-181.5	-151.6	-150.2	129.4	107.1	-123.9	105.3	93.7
51	127.9	-193.5	-284.7	-156.6	-108.5	98.0	-234.2	95.0	-35.1
52	-13.8	-257.6	-228.9	-208.3	-23.1	223.8	-180.9	222.1	14.5
55	439.1	-1640.1	-44.8	-1294.8	581.3	29.7	-2.5	-6.7	-839.4
56	379.6	-1190.0	-696.1	-970.0	111.8	527.1	-547.2	512.4	-474.3
57	3020.3	-3680.7	-1471.5	-3014.8	-366.1	914.9	-1042.9	829.5	-1895.0
59	-652.3	-58.8	-35.5	-44.3	917.4	384.9	-35.3	399.9	-417.3
60	-369.1	-432.6	-181.7	-346.1	554.4	379.5	-147.2	383.5	-270.0
61	-412.9	-488.5	-44.2	-374.7	886.3	90.1	-35.4	84.4	-440.1
63	-2638.5	-1559.3	-2520.7	-1374.0	784.7	4210.9	-2198.2	4288.2	3659.3
64	-171.7	-630.7	-858.9	-529.1	589.0	1114.9	-745.6	1131.3	847.9
65	-1418.9	248.2	173.3	207.3	530.3	1778.1	129.5	1841.8	248.1
68	-11.4	-2137.6	1609.6	-1633.7	389.3	-1513.4	1282.4	-1585.4	-292.3
69	-514.0	-584.2	476.6	-434.7	254.3	-790.3	364.4	-825.6	52.4
70	-358.2	-928.8	333.5	-702.9	716.9	-522.3	268.3	-557.7	-455.9
72	-559.5	-16.8	-9.5	0.5	860.8	-283.5	-14.0	-292.3	-401.2
73	-759.5	23.5	-30.9	31.0	255.0	-485.4	-37.6	-503.1	-402.1
74	-579.2	508.9	-193.3	402.1	809.6	-490.7	-161.7	-496.2	-403.2
76	-813.4	1564.3	-1562.3	1279.3	64.3	-1856.0	-1273.1	-1902.8	315.7
77	-221.2	145.7	-146.3	147.7	810.0	-1156.0	-155.7	-1195.6	1546.9
78	-3152.4	244.1	-76.6	320.1	-90.8	-3828.5	-253.6	-3967.0	4675.5
81	-1149.4	-151.1	86.5	-93.2	484.7	-1500.8	24.1	-1554.3	367.3
82	-2880.0	953.8	-1452.2	897.8	-86.9	-3608.6	-1367.1	-3740.5	3895.6
84	3515.5	2922.1	-2932.0	2367.2	-1373.6	-1141.9	-2278.1	-1132.8	-1242.8
85	1072.4	1873.7	-336.7	1483.9	217.1	-222.2	-216.3	-185.4	-903.8
87	-303.1	652.1	-110.4	525.3	739.0	-183.4	-87.3	-172.6	-449.3
88	-611.4	217.0	-81.5	197.3	834.3	-429.4	-79.2	-436.9	-373.3
91	-101.2	1123.8	287.1	878.8	602.5	353.9	250.9	398.4	-571.7
92	-312.6	770.0	545.6	597.1	225.2	682.5	443.1	728.3	-123.5
93	681.9	2382.9	1580.6	1841.3	139.8	1196.7	1314.3	1294.9	-653.7
95	-3007.5	609.7	1236.4	415.4	528.6	4239.0	904.5	4382.7	4561.7
96	-78.9	86.2	129.1	54.6	1168.5	1248.1	92.8	1289.5	1659.8
97	-1336.5	-1432.6	-1240.0	-1171.3	659.2	2247.3	-988.7	2280.0	299.1
99	-685.9	-319.5	-107.5	-224.3	965.1	457.6	-85.5	468.7	-454.6
100	-730.7	138.3	41.3	115.5	346.8	448.1	30.6	470.9	-432.9
101	-530.5	192.2	11.7	165.4	895.8	193.4	8.4	209.1	-425.4
102	467.3	1034.3	-902.4	838.3	-151.9	-537.8	-732.4	-541.3	-278.5
103	-481.7	517.6	-826.3	443.1	65.6	-995.3	-731.6	-1026.7	762.5
104	-303.8	476.5	-248.3	394.0	399.3	-419.0	-207.5	-424.2	-212.7
5	-4106.7	2840.1	67.2	2029.8	-651.9	379.8	-107.7	511.4	-4333.7
8	3918.3	2265.5	2780.1	1607.2	1435.9	-908.4	2928.2	-958.1	4404.9
9	-2316.7	1237.0	-1915.3	1065.7	-1834.1	88.4	-1833.7	104.8	-4889.1

11	4024.7	264.1	3849.0	220.1	740.4	-770.4	3887.0	-719.5	6657.6
12	-2564.4	763.2	-2343.9	641.9	-2417.5	78.8	-2230.3	106.5	-5551.8
14	4123.2	752.4	3471.2	6.8	-2514.8	-704.3	3903.0	-603.5	4264.4
15	-2399.9	4117.5	-1633.2	2791.3	-8053.4	54.4	-1510.3	353.0	-11515.5
16	-2604.8	-4719.8	-1296.6	-3350.8	-7064.1	-3.6	-1235.0	-301.3	-11193.3
17	3758.6	-1457.9	3055.4	-752.0	-2346.3	251.7	3473.6	119.3	3303.2
18	-2504.8	-714.7	-2262.3	-590.7	-2109.8	190.9	-2152.2	154.9	-5232.1
20	3871.4	-651.1	3793.4	-601.5	789.6	299.3	3821.8	252.9	6277.0
21	-2587.2	-1176.2	-1769.2	-998.5	-1640.4	118.1	-1694.4	100.0	-4598.2
23	3871.0	-2435.6	3203.5	-1756.4	1910.9	630.7	3340.0	691.5	4345.7
24	-5036.4	-2282.3	141.3	-1505.1	-796.0	-605.1	-49.6	-684.3	-4725.4
27	-902.4	356.6	-156.9	288.1	-1159.3	-114.5	-119.7	-111.3	-1281.4
28	1651.2	210.5	-118.9	150.1	1868.9	-287.3	-85.2	-286.5	1550.7
32	1683.6	383.4	24.0	328.6	1679.6	104.8	38.4	108.9	1400.7
36	282.9	192.9	-201.3	152.1	-68.4	-67.2	-164.9	-67.6	-54.8
40	-919.6	-375.8	-134.9	-308.1	-1032.1	130.1	-99.2	124.5	-1214.6
41	28.0	-293.7	-99.6	-234.9	198.9	91.2	-79.6	87.6	-147.3
45	2530.0	-292.5	-33.2	-250.1	1970.1	40.4	-20.0	35.2	1867.3
49	1935.3	-250.5	-310.5	-207.7	1782.0	166.1	-267.7	164.5	2035.0
53	-3415.2	-1534.7	-408.2	-1220.2	407.4	1080.5	-242.9	1117.7	-3379.6
54	485.4	-2309.9	-666.7	-1846.9	558.7	612.3	-518.6	581.9	-1249.0
58	-1194.2	-487.8	-145.7	-381.8	320.2	387.0	-126.9	393.0	-1065.7
62	6498.7	-1074.1	-882.0	-936.0	12566.7	2955.8	-746.8	2995.8	8790.6
66	-6257.8	-221.7	-40.8	-70.0	-2706.5	-1073.7	-105.2	-1154.5	-6180.1
67	-499.8	-1221.0	663.1	-915.6	999.3	-1131.7	518.6	-1193.4	-92.8
71	-1514.7	43.6	-32.8	36.8	-20.0	-507.0	-34.0	-527.0	-1149.3
75	4671.4	1138.5	-779.6	1091.7	8367.6	-2827.7	-695.9	-2929.8	7330.3
79	-2973.8	1717.2	-652.7	1428.7	140.1	-1378.7	-464.2	-1433.5	-2936.2
80	3540.9	1265.0	-894.0	1211.4	7666.0	-2584.8	-815.6	-2645.7	5812.8
83	1790.4	2389.1	-1174.2	1952.1	1134.1	-828.4	-958.5	-821.6	-208.1
86	-1153.3	612.3	-223.7	505.4	81.6	-468.2	-197.7	-471.0	-1066.9
89	-6063.7	773.6	34.0	555.1	-2441.2	879.2	-16.4	977.7	-6170.9
90	-117.7	1538.7	710.7	1197.4	906.4	922.0	586.3	999.3	-312.9
94	5634.7	-477.4	-172.1	-495.8	10531.8	3081.9	-160.5	3173.9	8441.2
98	-1539.1	174.5	31.2	160.5	22.0	439.0	32.0	464.6	-1250.6
1	77051.0	-2974.8	-60742.5	-7259.7	-12804.0	5758.4	-80541.8	6960.1	98218.5
2	-51436.5	-998.8	603.4	-770.8	-55563.8	-2511.4	660.8	-3074.5	-51671.0
3	-52147.2	365.7	880.1	289.1	-57181.4	2165.4	1023.9	2684.3	-53161.4

Table S 34: Components of ^{1}H, ^{13}C, and ^{31}P hyperfine coupling tensors for **2** computed with ZORA *ω*B97X-D3(BJ)- def2-TZVP in units of ppm Å^{-3}. Atom number refers to the position of the atom in the cartesian coordinates of the molecule.

Atom No.	A_{xx}	A_{xy}	A_{xz}	A_{yx}	A_{yy}	A_{yz}	A_{zx}	A_{zy}	A_{zz}
6	-794.1	1036.1	71.6	807.3	1038.4	302.9	45.3	352.7	-1000.7
7	1089.1	2490.3	1210.6	1963.1	2807.1	1647.9	1005.5	1792.9	809.0
10	422.9	818.6	-219.6	662.3	490.4	-141.3	-185.7	-124.6	-180.8
13	784.3	289.6	-427.6	248.3	117.9	-107.9	-338.9	-108.1	-25.5
19	711.7	-367.9	-393.0	-325.8	185.8	132.7	-308.6	122.0	-50.5
22	246.0	-814.6	-137.2	-654.6	660.4	96.8	-120.3	82.8	-225.5
25	896.5	-2242.1	1329.1	-1704.0	2852.1	-1896.0	1030.5	-1999.2	1255.7

26	-963.7	-851.7	139.9	-610.3	1067.8	-534.2	75.4	-564.9	-930.1
29	-14.3	269.1	-222.0	216.8	-19.3	-225.4	-175.8	-226.4	-6.1
30	141.1	201.2	-129.9	164.5	148.6	-119.0	-106.2	-118.0	37.0
31	-55.9	290.8	-156.4	228.0	132.2	-170.4	-125.2	-167.7	-194.6
33	70.1	321.5	-35.5	258.1	67.3	10.5	-22.7	17.9	-215.4
34	204.2	216.4	-58.3	179.0	110.1	-34.2	-44.2	-30.9	-22.2
35	234.9	282.7	16.6	232.4	-63.2	-3.2	30.0	2.3	-174.4
37	283.7	139.1	-166.4	107.0	-140.3	-35.2	-133.4	-34.3	-142.8
38	123.3	146.8	-136.6	118.5	-77.3	-56.0	-111.0	-55.3	-86.8
39	181.4	150.8	-267.9	117.6	-145.7	-66.8	-220.5	-67.9	-52.2
42	-2.3	-205.3	-62.5	-166.6	84.3	60.5	-48.6	57.2	-130.0
43	-73.4	-281.4	-96.4	-219.7	220.7	120.0	-75.5	115.6	-187.3
44	13.7	-304.5	-25.8	-243.2	167.2	3.5	-17.2	-2.5	-191.5
46	226.6	-291.4	18.8	-245.4	-54.6	-1.0	29.0	-6.5	-165.6
47	296.3	-181.3	-121.6	-147.5	-102.1	38.2	-94.7	33.8	-184.7
48	307.7	-195.7	-67.2	-165.2	159.1	44.5	-51.9	41.0	52.6
50	198.2	-188.7	-145.6	-158.0	151.1	107.0	-118.9	104.0	96.9
51	122.9	-203.4	-281.8	-166.9	-104.3	101.5	-232.1	96.6	-39.9
52	-17.4	-264.6	-215.0	-215.8	-4.6	230.5	-169.4	226.5	-6.5
55	-50.7	-1129.2	-91.1	-894.0	666.3	89.9	-55.1	63.0	-664.8
56	151.8	-1042.3	-558.2	-858.2	209.8	501.3	-438.7	483.9	-429.1
57	1387.1	-2590.7	-530.6	-2115.0	232.4	465.2	-328.4	387.1	-1490.3
59	-710.6	143.2	31.0	114.9	898.5	666.9	13.0	698.7	-285.6
60	-385.0	-326.9	-184.5	-266.5	628.9	485.9	-154.2	494.0	-128.9
61	-448.9	-239.3	-55.0	-181.7	993.4	234.7	-48.7	240.8	-341.9
63	375.9	-2260.8	-4929.4	-1928.6	691.4	4181.5	-4223.0	4183.0	4252.6
64	1457.7	-627.4	-1039.7	-533.1	2145.2	1261.9	-914.0	1269.9	2960.8
65	-1817.3	312.7	381.8	254.8	332.7	3096.8	276.7	3200.0	2172.6
68	-85.6	-1778.3	1144.7	-1352.1	556.4	-1181.4	923.3	-1244.1	-393.3
69	-514.1	-523.8	380.6	-381.8	291.7	-703.0	285.4	-733.8	-53.5
70	-363.5	-778.0	240.8	-586.5	737.4	-434.4	193.1	-465.8	-422.5
72	-553.0	20.7	-20.3	31.1	843.4	-250.1	-24.3	-253.8	-420.3
73	-739.1	32.3	-36.5	42.6	255.0	-450.9	-43.9	-464.9	-427.0
74	-558.7	518.8	-197.1	415.9	816.1	-481.9	-165.4	-480.9	-407.1
76	-946.9	1462.7	-1499.3	1213.6	210.6	-1963.6	-1244.8	-2012.7	519.6
77	-355.6	94.7	-100.5	113.8	673.6	-1098.6	-129.2	-1135.4	1193.5
78	-2655.7	-217.0	585.6	-42.9	-48.7	-3420.8	268.8	-3549.5	3741.3
81	-1123.0	-185.0	140.1	-110.3	296.7	-1450.8	50.6	-1503.4	513.6
82	-2857.1	719.9	-1243.5	695.1	-570.8	-3291.5	-1239.0	-3432.9	4057.9
84	2579.2	2386.4	-2668.5	1941.8	-1123.8	-1103.4	-2115.4	-1097.3	-835.1
85	854.2	1797.6	-421.7	1438.0	263.4	-303.2	-282.5	-255.0	-907.1
87	-346.9	564.0	-132.5	464.8	713.7	-250.9	-108.6	-239.0	-423.0
88	-610.7	142.3	-66.3	146.3	786.4	-487.7	-73.5	-495.3	-321.1
91	-187.1	978.4	159.4	774.5	684.2	239.7	149.6	285.2	-561.5
92	-374.5	685.5	376.3	533.3	350.9	580.5	309.8	624.8	-248.3
93	359.3	2015.8	1049.3	1565.6	438.3	927.2	901.0	1022.7	-680.0
95	-2455.5	847.0	1319.2	599.5	891.0	3909.5	975.2	4051.9	3358.2
96	-162.1	91.5	91.5	58.1	1174.3	1188.7	59.1	1227.3	1250.2
97	-1291.2	-1403.2	-1140.0	-1153.1	977.2	2232.1	-914.8	2252.7	216.7

99	-656.3	-385.5	-107.1	-273.0	1010.9	377.7	-85.5	382.8	-505.1
100	-723.5	87.4	9.7	78.7	386.0	379.6	5.3	399.8	-491.9
101	-535.4	113.3	-9.5	111.5	896.1	101.9	-10.4	114.9	-450.1
102	329.8	924.6	-803.6	756.2	-53.2	-535.9	-658.8	-536.3	-184.2
103	-512.4	432.3	-751.7	375.5	-13.6	-953.3	-678.4	-986.5	837.7
104	-399.5	402.9	-235.3	341.8	309.0	-451.5	-202.2	-456.3	-223.1
5	-4382.5	2700.5	-52.0	2009.4	-1051.3	373.4	-170.9	557.1	-4754.3
8	4518.5	2181.8	3163.5	1515.5	1708.8	-1043.7	3344.8	-1083.3	5103.2
9	-2758.5	1269.0	-2207.0	1099.3	-2087.0	118.5	-2116.6	133.3	-5395.8
11	4513.7	224.1	4275.6	189.7	803.6	-806.8	4309.2	-759.6	7090.2
12	-2774.9	778.8	-2536.8	659.1	-2480.8	54.0	-2413.9	91.6	-5782.3
14	5046.0	837.6	3729.4	65.6	-2026.2	-617.9	4228.8	-462.6	5038.4
15	-2746.5	4202.4	-2379.9	2876.6	-7850.5	-31.6	-2176.6	273.3	-12352.6
16	-2033.4	-4859.1	-1888.5	-3578.1	-5954.8	-38.4	-1822.5	-386.6	-11032.4
17	4695.8	-1293.8	3324.8	-511.8	-1777.6	587.5	3755.4	405.8	4270.8
18	-2671.7	-900.8	-2405.9	-781.2	-2147.8	-32.4	-2297.9	-83.2	-5457.0
20	4381.3	-265.3	4268.8	-240.9	937.2	821.6	4290.8	766.8	6726.0
21	-2935.4	-1343.4	-1968.5	-1172.6	-1881.7	-140.9	-1894.5	-160.9	-5095.2
23	4538.1	-2100.2	3747.8	-1402.7	2269.5	992.9	3875.4	1052.1	5015.2
24	-4394.9	-2389.9	182.9	-1652.0	-393.4	-799.6	-36.8	-918.0	-4202.8
27	-1001.3	353.8	-164.1	288.1	-1258.2	-123.3	-122.9	-118.9	-1368.6
28	1804.1	198.5	-116.9	140.9	2043.8	-311.7	-82.0	-309.3	1726.0
32	1815.3	397.0	22.0	343.8	1823.3	106.5	40.0	110.9	1524.3
36	282.5	190.1	-213.3	150.5	-68.8	-70.0	-174.5	-70.0	-45.2
40	-1015.7	-380.2	-114.5	-315.7	-1128.9	125.7	-79.6	116.9	-1328.6
41	36.4	-300.9	-87.2	-242.9	212.5	72.0	-68.4	66.4	-153.7
45	2760.5	-287.7	-20.4	-247.7	2146.6	38.4	-8.4	31.6	2042.6
49	2088.2	-264.5	-308.5	-222.9	1948.9	187.7	-266.9	184.5	2200.6
53	-3454.0	-1427.9	-496.2	-1154.5	278.9	1269.0	-386.2	1320.2	-3302.4
54	364.6	-1748.0	-479.0	-1405.5	1012.1	519.4	-362.6	491.0	-728.7
58	-1498.7	-334.6	-152.1	-266.1	101.6	539.5	-140.5	561.5	-1233.4
62	16106.0	-910.4	-1323.0	-738.3	22341.8	4609.8	-1038.9	4625.0	19261.5
66	-5649.1	-218.5	0.4	-74.0	-2474.8	-864.4	-68.4	-919.2	-5667.9
67	-407.4	-1020.9	515.0	-760.0	1130.5	-962.1	392.6	-1010.1	-84.0
71	-1495.9	46.8	-40.0	40.8	-5.6	-474.6	-36.8	-489.0	-1148.9
75	1939.3	621.1	-459.0	617.9	5064.8	-2540.0	-471.4	-2628.4	4521.3
79	-3327.6	1433.9	-564.3	1208.6	-329.0	-1447.1	-414.2	-1519.9	-3148.7
80	2088.6	908.0	-781.6	891.6	5367.3	-2515.2	-757.2	-2574.8	4591.8
83	1468.3	2088.6	-1122.5	1727.6	1017.7	-885.2	-934.4	-868.4	-58.8
86	-1196.6	513.0	-218.9	434.6	0.0	-506.6	-204.1	-508.2	-1027.3
89	-5639.1	629.9	-20.8	458.6	-2263.5	524.2	-32.4	608.7	-5873.2
90	-175.7	1317.0	471.8	1034.1	1120.1	729.5	397.4	804.8	-352.2
94	3331.6	-195.3	-73.2	-222.5	7704.8	2684.1	-92.8	2768.1	5642.7
98	-1483.1	115.7	-8.0	122.9	116.1	350.2	8.8	370.6	-1244.2
2	-53524.1	-1113.9	781.0	-890.0	-59983.9	-3321.2	723.9	-4055.2	-54521.9
3	-52203.5	1209.7	781.5	1064.2	-58520.0	2677.1	889.8	3306.1	-53894.2

Table S 35: Components of ^{1}H, ^{13}C, and ^{31}P hyperfine coupling tensors for **4** computed with ZORA ωB97X-D3(BJ)- def2-TZVP in units of ppm Å^{-3}. Atom number refers to the position of the atom in the cartesian coordinates of the molecule.

Atom No.	A_{xx}	A_{xy}	A_{xz}	A_{yx}	A_{yy}	A_{yz}	A_{zx}	A_{zy}	A_{zz}
6	-813.5	1046.9	80.6	812.1	1032.9	327.3	47.7	383.8	-1010.5
7	1105.7	2540.9	1264.3	1995.1	2830.6	1704.1	1038.2	1861.6	859.5
10	430.1	825.4	-214.5	666.8	497.2	-135.3	-178.8	-112.6	-179.5
13	791.3	296.1	-429.1	254.4	123.8	-106.9	-332.9	-105.1	-26.2
19	724.2	-369.4	-396.3	-325.8	181.9	126.1	-304.6	113.7	-52.4
22	266.1	-826.4	-127.8	-663.4	647.9	82.6	-110.9	63.2	-226.6
25	964.9	-2292.4	1431.8	-1740.4	2727.7	-1926.1	1104.0	-2042.0	1327.1
26	-957.6	-888.3	165.7	-640.8	1023.6	-577.6	90.6	-613.8	-917.9
29	-15.2	272.6	-220.3	219.3	-13.9	-226.6	-172.0	-224.1	-13.0
30	143.0	203.9	-128.6	166.7	153.1	-118.3	-103.7	-115.2	34.7
31	-56.3	293.7	-154.6	229.8	136.8	-167.4	-122.1	-161.1	-199.7
33	71.0	324.2	-32.7	259.9	67.4	13.7	-18.8	23.5	-215.4
34	206.7	218.7	-56.5	180.7	111.9	-32.9	-41.2	-28.1	-22.3
35	237.3	284.8	19.6	234.0	-63.7	-2.1	34.6	5.1	-175.0
37	286.1	142.1	-166.5	109.7	-140.2	-35.7	-130.5	-33.5	-144.9
38	124.4	149.5	-136.3	120.7	-76.3	-56.4	-108.8	-54.4	-89.1
39	182.5	154.7	-268.6	121.1	-144.9	-68.7	-217.9	-68.0	-55.5
42	2.0	-208.8	-60.9	-169.3	82.3	57.0	-46.4	52.0	-132.4
43	-67.7	-288.6	-94.0	-225.6	220.5	111.9	-72.8	105.0	-191.8
44	21.7	-309.2	-21.6	-246.9	160.4	-2.4	-12.7	-10.5	-191.5
46	236.8	-288.7	20.3	-242.2	-62.0	-0.3	32.3	-7.3	-166.7
47	300.2	-179.8	-124.8	-145.5	-105.4	36.6	-94.8	31.0	-185.2
48	310.6	-195.9	-67.4	-164.9	154.4	43.4	-50.5	38.7	49.5
50	203.9	-191.9	-145.7	-160.4	154.4	106.6	-117.6	102.2	96.8
51	123.8	-208.1	-284.3	-170.5	-105.1	102.5	-231.1	95.9	-40.9
52	-15.4	-269.6	-214.5	-219.4	0.1	232.2	-167.0	225.6	-16.2
55	-83.8	-1086.6	-89.5	-860.7	683.7	87.9	-53.6	53.6	-645.5
56	140.0	-1037.8	-542.5	-854.9	223.4	494.0	-422.3	467.6	-436.7
57	1267.9	-2495.5	-437.2	-2040.0	291.2	407.6	-244.2	310.9	-1468.4
59	-718.4	156.3	38.2	123.2	895.2	699.8	13.0	730.1	-264.2
60	-394.0	-321.3	-185.3	-263.1	627.6	495.7	-157.6	499.7	-127.9
61	-455.5	-216.3	-54.1	-164.9	1001.1	249.4	-51.5	253.6	-335.6
63	780.9	-2379.6	-5078.1	-2026.5	662.4	4130.1	-4320.6	4103.2	4061.3
64	1587.5	-648.7	-1074.5	-551.8	2244.3	1266.0	-945.4	1266.1	3080.1
65	-1808.7	238.1	323.0	184.5	364.5	3219.5	205.3	3311.8	2452.2
68	-145.8	-1713.3	1086.3	-1293.6	576.5	-1158.5	868.1	-1222.0	-360.2
69	-527.1	-501.3	353.9	-361.0	305.2	-692.1	259.0	-720.5	-61.2
70	-373.1	-755.2	227.7	-567.0	744.9	-428.5	179.2	-461.3	-411.3
72	-554.7	31.0	-23.0	38.2	843.9	-238.3	-29.0	-239.1	-425.5
73	-738.7	42.5	-42.5	50.3	255.6	-439.4	-50.4	-449.2	-440.6
74	-551.6	529.2	-196.2	422.1	822.7	-465.2	-165.5	-456.1	-414.8
76	-926.0	1459.0	-1465.2	1205.2	246.9	-1910.4	-1206.7	-1934.6	437.6
77	-415.5	108.8	-121.2	125.5	616.7	-1077.5	-148.0	-1104.9	1050.3
78	-2612.8	-183.1	470.1	-16.8	-35.2	-3313.0	162.6	-3416.0	3443.1
81	-1140.7	-218.6	186.1	-138.9	218.0	-1467.0	79.3	-1517.0	589.8
82	-2932.1	675.1	-1245.2	649.6	-715.0	-3263.7	-1256.5	-3395.5	4263.1
84	2433.8	2356.3	-2621.8	1930.9	-1054.0	-1133.4	-2061.2	-1099.4	-800.8
85	760.6	1763.4	-448.0	1416.0	316.8	-336.0	-299.4	-272.3	-884.8

87	-369.6	530.2	-139.3	436.8	721.3	-280.9	-116.5	-265.1	-407.3
88	-618.7	105.8	-56.6	115.8	773.4	-517.4	-69.9	-523.1	-295.5
91	-222.5	942.5	123.6	744.1	724.4	205.8	117.3	253.9	-558.3
92	-399.6	667.2	334.1	515.1	398.2	556.2	270.7	598.7	-279.1
93	267.5	1976.0	924.5	1531.5	543.9	877.0	794.1	973.3	-693.1
95	-2404.5	863.4	1230.6	601.5	991.5	3806.7	876.9	3920.0	2913.8
96	-241.1	79.9	68.2	44.6	1139.4	1161.6	33.6	1191.8	1058.7
97	-1268.3	-1408.3	-1109.3	-1157.3	1053.8	2173.6	-891.1	2174.8	131.3
99	-643.6	-418.0	-106.5	-300.1	1018.4	342.8	-88.3	341.1	-517.2
100	-727.3	66.6	-1.0	60.0	398.4	352.8	-6.6	369.4	-514.4
101	-543.6	80.4	-15.3	83.6	900.7	67.0	-18.9	78.1	-457.1
102	281.1	905.0	-798.8	742.8	-37.0	-554.7	-650.8	-544.2	-164.3
103	-529.1	406.4	-743.7	352.8	-35.5	-956.4	-674.1	-983.0	894.3
104	-423.4	378.8	-232.4	321.8	300.0	-471.7	-201.8	-472.1	-205.9
5	-4368.1	2748.1	-49.6	2031.8	-997.3	404.6	-190.1	605.9	-4735.8
8	4556.6	2159.8	3203.1	1486.3	1792.4	-1134.9	3389.6	-1135.3	5197.7
9	-2785.3	1305.8	-2228.3	1136.5	-2114.6	171.3	-2129.4	191.7	-5465.4
11	4539.7	154.5	4310.8	118.1	830.0	-902.0	4342.9	-854.8	7125.8
12	-2753.7	820.4	-2536.8	691.9	-2466.0	90.0	-2405.5	136.9	-5785.9
14	5294.9	879.2	3729.4	73.6	-1761.6	-663.5	4283.6	-447.0	5214.5
15	-2772.1	4297.2	-2510.8	2886.2	-7633.6	-16.4	-2298.3	335.4	-12395.1
16	-1639.2	-4935.9	-1933.7	-3569.7	-5621.5	-117.7	-1875.3	-515.0	-10809.1
17	5004.8	-1251.4	3291.2	-425.0	-1509.5	670.3	3768.2	433.8	4384.5
18	-2614.0	-958.1	-2389.9	-828.0	-2130.2	-105.7	-2275.5	-164.1	-5384.1
20	4443.7	-141.3	4284.4	-114.5	953.7	972.5	4304.4	918.8	6634.3
21	-2929.8	-1405.5	-1970.1	-1234.2	-1926.9	-228.9	-1889.3	-253.3	-5092.0
23	4661.4	-2005.3	3787.0	-1299.8	2338.3	1141.3	3910.3	1165.4	4977.6
24	-4252.8	-2482.0	230.1	-1729.6	-375.4	-875.2	-16.0	-1008.5	-4096.3
27	-1008.1	357.8	-161.3	291.3	-1263.8	-123.3	-117.3	-116.1	-1380.3
28	1820.1	201.7	-115.7	142.9	2070.6	-310.5	-78.8	-305.3	1735.2
32	1827.3	400.2	26.8	346.6	1831.3	110.9	46.4	116.9	1536.7
36	284.9	194.1	-213.7	154.1	-67.2	-70.8	-171.7	-69.2	-47.6
40	-1007.7	-382.6	-112.1	-317.4	-1132.9	123.7	-75.2	112.5	-1333.4
41	41.2	-306.5	-84.4	-247.3	207.3	65.6	-64.8	58.0	-158.1
45	2746.9	-280.5	-25.6	-240.1	2119.4	38.4	-11.6	30.8	2018.6
49	2112.2	-270.5	-305.7	-226.9	1975.3	194.9	-261.7	189.3	2225.1
53	-3442.0	-1415.5	-497.4	-1149.3	280.5	1267.0	-387.8	1305.8	-3290.4
54	389.0	-1692.8	-458.6	-1367.8	1107.3	500.6	-338.6	461.0	-658.3
58	-1519.9	-325.4	-154.9	-260.9	94.8	550.7	-145.7	569.1	-1244.2
62	16550.6	-1030.9	-1405.1	-845.6	22589.9	4667.8	-1123.7	4682.6	19649.7
66	-5546.2	-194.1	3.6	-58.0	-2440.4	-809.2	-70.0	-850.0	-5568.6
67	-397.4	-972.9	482.2	-717.5	1173.8	-945.6	356.6	-988.1	-52.0
71	-1496.3	56.0	-49.6	46.4	-6.4	-463.4	-45.2	-470.2	-1155.3
75	1292.2	588.3	-468.6	581.9	4261.2	-2430.0	-477.4	-2492.0	3744.2
79	-3338.8	1349.4	-560.3	1146.5	-311.7	-1504.3	-415.8	-1578.3	-3069.5
80	2063.4	820.8	-752.4	810.8	5335.7	-2592.0	-743.6	-2639.6	4754.7
83	1337.0	2029.4	-1129.7	1696.0	993.3	-942.4	-919.6	-896.8	-64.0
86	-1233.4	477.0	-216.9	407.8	-23.6	-529.1	-207.7	-527.0	-1016.9
89	-5538.2	562.3	-46.8	397.0	-2196.2	413.8	-60.4	487.4	-5764.7

90	-233.3	1268.2	402.6	989.3	1172.6	679.1	335.8	752.0	-384.6
94	2441.6	-165.3	-88.8	-198.1	6647.9	2543.2	-117.3	2602.4	4578.6
98	-1479.9	86.8	-25.2	98.8	137.7	318.6	-6.0	331.0	-1252.2
2	-54382.1	-1247.5	691.6	-951.9	-61454.2	-3613.1	717.9	-4341.2	-55710.8
3	-52584.1	1428.6	585.0	1241.3	-59158.7	3059.9	828.2	3663.3	-54712.2

Table S 36: Components of ^{1}H, ^{13}C, and ^{31}P hyperfine coupling tensors for **5** computed with ZORA ωB97X-D3(BJ)- def2-TZVP in units of ppm $Å^{-3}$. Atom number refers to the position of the atom in the cartesian coordinates of the molecule.

Atom No.	A_{xx}	A_{xy}	A_{xz}	A_{yx}	A_{yy}	A_{yz}	A_{zx}	A_{zy}	A_{zz}
6	-870.6	990.9	63.5	754.0	1120.3	349.0	24.0	419.7	-1014.0
7	983.8	2565.5	1257.5	1992.3	3039.2	1823.7	1007.1	2008.0	892.6
10	395.2	823.4	-222.2	668.7	557.0	-147.0	-179.7	-109.5	-169.4
13	784.5	318.6	-437.9	281.1	153.5	-127.7	-326.8	-117.5	-21.0
19	772.1	-366.4	-392.4	-319.5	171.1	97.3	-287.9	84.3	-59.1
22	353.9	-851.8	-90.7	-690.1	583.6	30.1	-74.4	-2.1	-224.9
25	1210.6	-2333.9	1728.2	-1780.9	2289.0	-1966.9	1353.9	-2133.9	1625.2
26	-873.6	-979.7	251.5	-733.8	870.4	-700.0	158.2	-755.5	-835.2
29	-39.1	271.6	-213.3	221.1	7.9	-242.6	-162.5	-231.5	-20.0
30	125.9	203.3	-127.2	167.9	165.1	-125.5	-100.0	-117.4	30.2
31	-76.6	286.4	-150.9	225.2	159.3	-172.4	-116.4	-157.9	-204.7
33	54.5	326.5	-35.7	264.0	84.8	13.9	-17.5	29.4	-216.6
34	205.5	222.8	-59.1	186.3	135.3	-37.1	-40.3	-28.4	-12.6
35	228.7	293.1	15.1	244.6	-50.3	-5.4	36.3	6.2	-177.8
37	275.1	155.0	-174.8	125.0	-132.8	-43.4	-131.9	-36.8	-143.8
38	111.8	155.9	-140.5	128.8	-68.7	-64.1	-109.0	-58.1	-89.2
39	166.4	166.6	-276.2	135.2	-139.2	-82.1	-219.3	-75.6	-54.4
42	20.2	-216.5	-53.2	-177.2	69.4	43.1	-38.0	35.0	-140.0
43	-42.9	-308.2	-83.7	-245.6	208.5	84.0	-63.0	71.8	-205.9
44	51.0	-318.0	-5.0	-256.1	130.9	-22.6	4.6	-35.6	-191.3
46	266.9	-270.1	31.0	-224.4	-91.0	-1.2	47.2	-11.2	-169.0
47	314.9	-166.2	-126.3	-131.5	-119.7	27.7	-90.5	20.4	-189.4
48	327.5	-191.5	-63.7	-160.6	139.3	37.0	-44.0	30.5	43.5
50	227.9	-199.1	-143.6	-167.1	159.8	100.6	-113.7	94.0	94.8
51	135.5	-215.6	-284.9	-176.2	-114.7	96.7	-227.5	88.4	-50.8
52	3.2	-284.6	-212.7	-233.4	5.0	229.1	-164.2	218.5	-46.1
55	-31.6	-1090.3	-80.1	-880.5	620.3	68.6	-41.4	17.7	-650.5
56	218.4	-1048.4	-538.6	-875.3	166.9	464.8	-418.1	426.0	-466.5
57	1427.7	-2407.3	-345.6	-1992.7	121.7	323.3	-142.8	200.1	-1464.6
59	-727.9	106.6	10.5	64.5	923.3	719.6	-26.9	746.4	-260.5
60	-352.5	-359.6	-205.9	-308.7	642.5	494.9	-185.3	492.0	-117.7
61	-426.7	-256.6	-64.3	-213.4	1016.3	247.4	-68.1	245.9	-324.3
63	1089.0	-2574.3	-5535.8	-2216.4	555.3	4232.8	-4752.8	4193.3	4009.9
64	1795.8	-702.9	-1198.4	-611.2	2317.3	1254.0	-1078.2	1246.9	3197.3
65	-1838.0	126.7	183.8	54.5	368.0	3320.3	13.4	3390.7	2501.7
68	-122.3	-1593.3	990.0	-1204.5	492.3	-1066.2	806.5	-1151.0	-299.3
69	-508.4	-486.8	328.1	-352.6	284.8	-654.1	238.5	-687.0	-71.9
70	-339.5	-735.9	215.4	-563.4	707.1	-411.7	171.0	-453.2	-385.9
72	-551.5	3.8	-23.8	5.1	841.5	-229.8	-33.0	-226.7	-430.1
73	-730.4	21.9	-38.7	28.5	252.5	-425.7	-48.6	-429.7	-451.4

74	-565.8	490.1	-186.9	384.1	856.5	-458.5	-159.0	-433.5	-416.0
76	-980.0	1322.4	-1306.3	1097.7	363.9	-1851.0	-1073.4	-1821.7	371.8
77	-507.3	74.1	-99.8	97.1	508.2	-1030.4	-134.8	-1044.8	827.9
78	-2468.5	-260.6	461.5	-85.7	-116.9	-3042.3	133.6	-3115.3	2955.6
81	-1113.2	-276.3	281.8	-192.0	25.0	-1439.1	145.6	-1491.4	792.6
82	-3060.1	480.1	-1071.1	471.3	-1108.9	-3060.5	-1161.2	-3178.6	4758.1
84	2336.6	2428.6	-2719.7	2053.2	-927.5	-1271.9	-2120.0	-1167.8	-744.0
85	573.5	1741.3	-512.0	1425.7	427.8	-432.0	-342.2	-331.4	-878.9
87	-414.9	457.4	-155.8	374.1	732.1	-359.0	-133.5	-334.1	-372.6
88	-623.3	27.0	-33.8	43.4	724.4	-587.1	-56.9	-591.2	-227.5
91	-317.0	854.1	44.7	669.9	841.8	121.2	48.4	179.1	-566.0
92	-472.1	591.3	226.5	444.7	527.9	495.4	175.2	537.6	-350.1
93	-1.8	1856.2	674.0	1438.7	847.5	812.4	589.6	918.9	-729.2
95	-2425.2	631.4	744.4	351.6	1336.7	3619.2	403.2	3677.5	1965.8
96	-390.4	-0.5	-16.8	-43.3	1087.5	1070.7	-55.6	1087.0	628.3
97	-1082.5	-1464.2	-1009.6	-1231.3	1139.8	1860.3	-833.0	1829.8	-202.3
99	-584.2	-504.8	-92.8	-388.2	1005.4	216.7	-85.4	201.3	-545.5
100	-725.1	-4.6	-27.8	-9.9	424.5	273.8	-36.3	284.5	-567.5
101	-542.2	-7.3	-23.2	0.3	897.4	-31.1	-32.2	-24.1	-460.0
102	237.8	876.3	-827.4	734.1	11.9	-611.0	-672.5	-575.4	-77.7
103	-578.8	337.9	-724.6	295.9	-130.1	-949.7	-669.8	-964.1	1017.5
104	-447.9	322.9	-229.3	274.7	289.2	-521.4	-203.6	-513.5	-134.9
5	-4193.6	2686.1	-125.3	1970.9	-491.8	419.8	-277.7	657.1	-4499.7
8	4551.0	2263.9	3328.8	1602.8	2004.9	-1082.9	3548.1	-988.9	5398.6
9	-2987.8	1306.2	-2342.7	1145.3	-2095.0	125.7	-2226.3	161.7	-5623.5
11	4772.3	272.9	4504.5	237.3	866.0	-747.6	4533.3	-700.7	7216.2
12	-2867.0	808.0	-2616.4	677.9	-2413.5	-33.2	-2466.8	37.6	-5850.0
14	5701.9	1261.4	3808.6	458.6	-1353.0	-407.0	4468.9	-46.4	5521.0
15	-2953.0	4494.5	-2758.1	3033.8	-7008.5	-198.9	-2470.4	273.3	-12433.9
16	-871.2	-4927.9	-1864.1	-3508.1	-5483.4	-481.4	-1788.0	-986.5	-10524.6
17	5510.6	-824.0	3350.8	44.8	-1129.3	1093.3	3894.6	764.0	4629.8
18	-2557.6	-1135.7	-2378.7	-994.9	-2247.9	-399.8	-2251.5	-471.8	-5384.1
20	4661.4	322.2	4372.5	349.4	1119.7	1528.7	4390.9	1477.9	6548.7
21	-2887.4	-1596.8	-1948.9	-1427.1	-2175.0	-542.3	-1858.1	-573.9	-5165.6
23	4965.2	-1646.8	3924.3	-943.6	2482.0	1565.5	4033.1	1507.1	5014.0
24	-3671.7	-2703.3	394.6	-1972.9	-402.2	-1102.9	111.7	-1288.2	-3701.4
27	-1060.5	366.6	-162.9	303.7	-1273.0	-133.7	-111.7	-118.1	-1411.1
28	1798.9	192.5	-108.9	136.1	2087.4	-316.1	-68.8	-302.5	1720.4
32	1914.5	411.4	28.0	360.2	1956.5	114.5	52.8	125.7	1637.6
36	270.1	205.3	-219.7	167.7	-58.4	-82.4	-171.3	-74.8	-50.8
40	-1000.1	-382.6	-99.6	-317.4	-1182.2	112.1	-58.4	96.4	-1375.9
41	66.4	-319.8	-72.0	-261.3	184.9	42.8	-51.2	30.4	-168.5
45	2782.9	-248.1	-29.6	-208.5	2100.2	34.0	-11.6	24.8	2015.4
49	2220.7	-283.3	-300.9	-237.7	2067.0	205.7	-254.9	196.5	2311.9
53	-3165.5	-1561.1	-560.7	-1317.8	405.0	1234.2	-458.6	1256.2	-3164.7
54	323.0	-1701.6	-444.6	-1405.5	798.4	426.6	-321.8	364.6	-874.0
58	-1552.7	-380.2	-181.7	-328.2	35.6	535.9	-179.7	546.7	-1313.8
62	18151.0	-1231.4	-1578.7	-1050.1	24531.2	4940.3	-1351.8	4986.4	21197.6
66	-5324.5	-270.9	20.0	-136.1	-2417.1	-728.3	-60.8	-754.8	-5344.1

67	-398.6	-935.6	450.6	-697.5	1062.1	-890.4	325.0	-934.8	-74.8
71	-1508.3	17.2	-55.6	4.4	-33.6	-459.0	-50.0	-453.0	-1173.8
75	159.7	359.4	-370.2	372.2	2807.3	-2249.5	-402.6	-2269.9	2469.6
79	-3236.3	1174.6	-575.1	1009.7	-108.1	-1718.0	-435.0	-1789.6	-2668.5
80	1880.1	605.5	-652.3	618.7	4939.5	-2706.5	-689.9	-2734.9	5031.2
83	1459.5	2039.0	-1210.2	1769.6	1435.9	-1118.9	-945.6	-1003.3	270.1
86	-1346.6	396.2	-216.1	343.0	-122.1	-599.1	-216.5	-590.3	-1021.3
89	-5379.7	344.2	-110.1	183.7	-2123.8	127.7	-124.9	185.3	-5571.8
90	-414.2	1119.7	244.5	854.8	1301.4	566.7	199.7	639.9	-489.0
94	1008.9	-288.5	-216.5	-337.8	4883.9	2175.8	-258.9	2197.0	2637.6
98	-1499.5	-10.8	-60.0	8.8	125.7	219.3	-43.2	218.1	-1313.0
2	-54812.3	-1041.1	778.5	-643.1	-62940.7	-4291.7	1001.8	-5000.2	-56970.2
3	-53451.0	1816.6	172.1	1611.4	-59815.7	4036.9	664.7	4586.1	-56820.9

6.4 Prediction and Simulation of the NMR spectra

6.4.1 Calculation of chemical shift with two approaches

The paramagnetic ^{1}H, ^{13}C, ^{19}F and ^{31}P NMR spectra of complexes **2-5** were calculated with CUVNMR[45] using two different approaches for the calculation of δ_{HF} in the relation:[46]

$$\delta_{\mathrm{calc}} = \delta_{\mathrm{orb}} + \delta_{\mathrm{HF}} \qquad \text{(S5)}$$

δ_{dia} was computed by DFT this time. For this, nuclear magnetic shielding tensors were calculated at the ZORA ωB97X-D3(BJ)- def2-TZVP level of theory. Diamagnetic shifts were then obtained by referencing against computed shielding tensors of tetramethylsilane (TMS) at the same level of theory.
For the first approach δ_{HF} was calculated from the following relations:

$$\delta_{\mathrm{HF}} = \delta_{\mathrm{FC}} + \delta_{\mathrm{PC}} \qquad \text{(S6)}$$

$$\delta_{\mathrm{FC}} = \frac{g_e^2 \mu_0 \mu_B^2 S(S+1) A_{\mathrm{iso}}}{3kT} \qquad \text{(S7)}$$

$$\delta_{\mathrm{PC}} = \frac{10^6}{12\pi r^5}\left[3\vec{r}^{T}\boldsymbol{\chi}\vec{r} - r^2 \mathrm{Tr}(\boldsymbol{\chi})\right] \qquad \text{(S8)}$$

In the equations χ is given in units of m^3 and A_{iso} is given in the units ppm/m^3 obtained from the values in MHz by multiplying with the factor $\frac{10^{12} h}{\gamma_I \, \mu_0 \mu_{\mathrm{B}} \, g_e \hbar}$.

With g_e as the electron Lande factor, μ_0 as the vacuum magnetic permeability, μ_{B} as the Bohr magneton, γ_I as the gyromagnetic ratio, h and $\hbar$ as the Planck and the reduced Planck constant, S as the total electron spin of the molecule, k as the Boltzmann constant, T as the temperature, A_{iso} as the isotropic hyperfine coupling constant, $\boldsymbol{\chi}$ as the magnetic susceptibility and r as the metal nucleus vector. For this purpose, A_{iso} was obtained from DFT calculation of the hyperfine tensor (*vide supra*) and $\boldsymbol{\chi}$ was obtained from the CASSCF calculations (*vide supra*). To find the most suitable DFT functional for the prediction and simulation of the NMR spectra a functional screening was performed for **2**, in which δ_{FC} values calculated with different functionals were used to obtain δ_{obs} values with the calculation of δ_{orb} and δ_{PC} as described above. The deviation from simulated and experimental spectra was quantified as the sum of the absolute value difference between observed and calculated values for ^{1}H and ^{13}C resonances $\Sigma(|\delta_{\mathrm{exp}} - \delta_{\mathrm{calc}}|)$ and the following results were obtained:

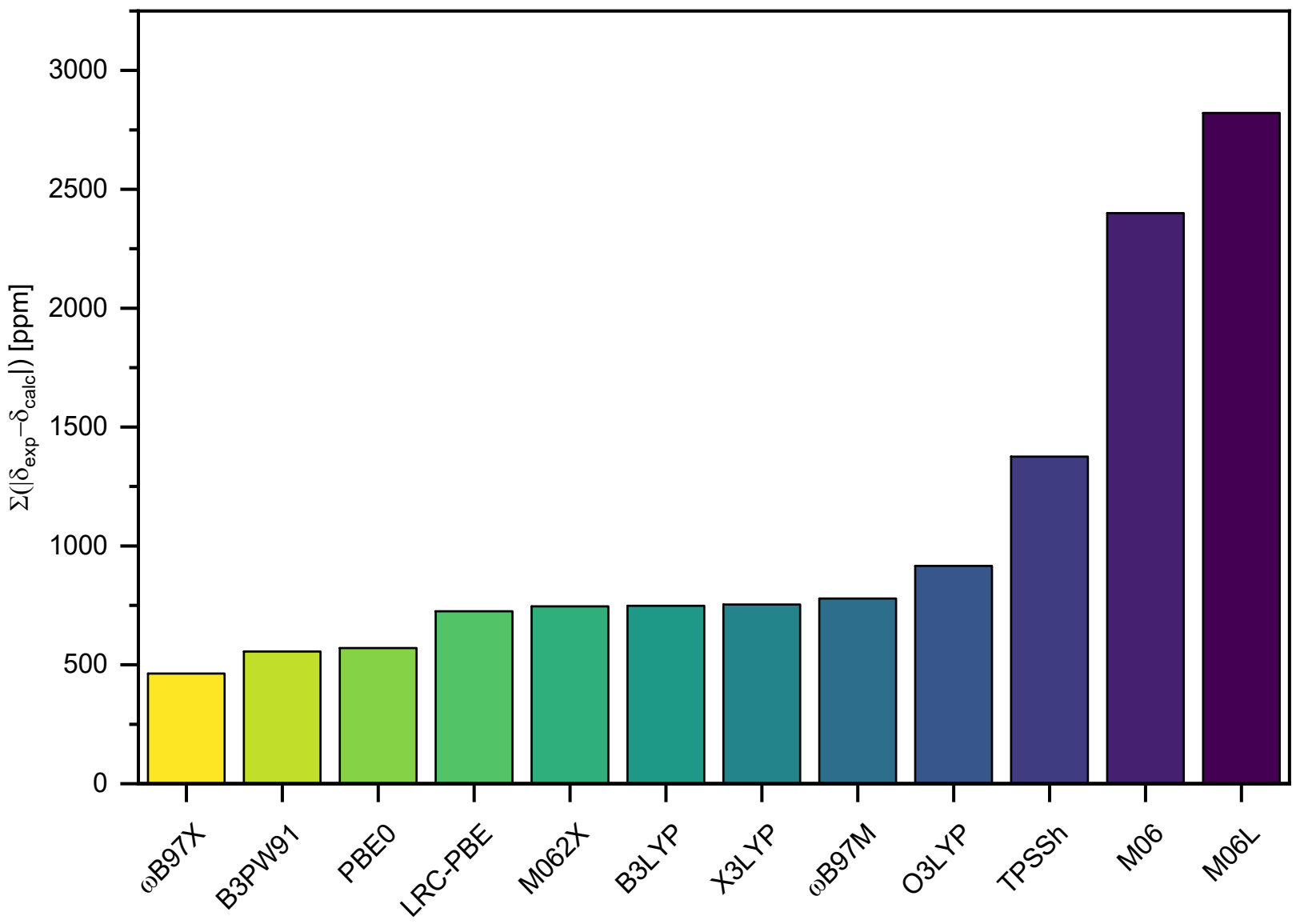

Figure S 59: Deviation between experimentally obtained δ_{exp} and calculated δ_{obs} (equation S2 and S3) and chemical shift values with different DFT functionals.

Based on the results of the functional screening ωB97X was chosen as the best functional and used for the calculation of hyperfine tensors for complexes **2-5**.

In the second approach δ_{obs} was calculated from the relationship between the susceptibility tensor χ and hyperfine coupling tensor $\boldsymbol{A}$ expressed in units of ppm Å^{-3} each nucleus as given in the main manuscript of this publication:[47-48]

$$\delta_{\mathrm{HF}} = \frac{1}{3}\mathrm{Tr}(\boldsymbol{A\chi}) \qquad \text{(S9)}$$

The susceptibility tensor was iteratively refined to minimize the root-mean-square deviation (RMSD) between δ_{obs} and the experimental shifts. Line broadening effects were simulated by estimating electron relaxation times (T_{1e}, T_{2e}), reorientation correlation times (τ_R) and ignoring exchange correlation (since $\tau_M \gg T_{1e}, T_{2e}, \tau_R$ was assumed) giving transverse relaxation rates from which the width at half-maximum of the NMR signals was derived.

The molecular orientations of the complexes **2-5** was chosen such that the approximate mirror plane (neglecting ^{t}Bu rotations) of the molecules coincided with the xz-plane in the reference coordinate system(see Figure S60). This led to a simplified susceptibility tensor, with the off-diagonal elements $\chi_{xy} = \chi_{yx} = \chi_{yz} = \chi_{zy} = 0$.

Furthermore, the paramagnetic shift δ_{HF} can be decomposed into Fermi contact (δ_{FC}) and pseudocontact (δ_{PC}) contributions:

$$\delta_{\mathrm{FC}} = \chi_{\mathrm{iso}} A_{\mathrm{iso}} \qquad \text{(S10)}$$

$$\delta_{\mathrm{PC}} = \frac{1}{3}\mathrm{Tr}(\Delta\boldsymbol{\chi}\boldsymbol{A}_{\mathrm{dip}}) \qquad \text{(S11)}$$

With $\Delta\boldsymbol{\chi} = \boldsymbol{\chi} - \chi_{\mathrm{iso}} I$ and $\boldsymbol{A}_{\mathrm{dip}} = \boldsymbol{A} - \boldsymbol{A}_{\mathrm{iso}} I$.

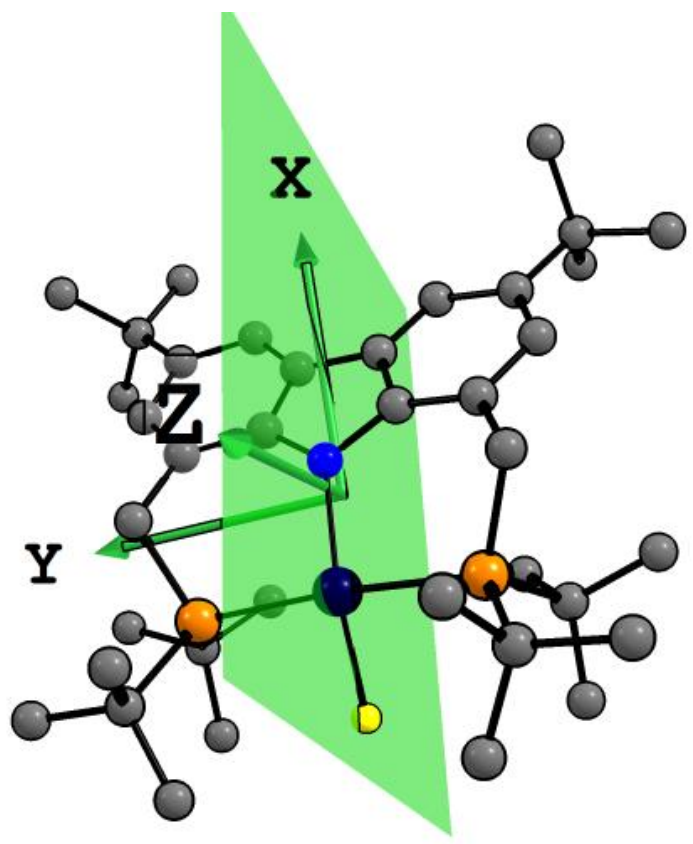

Figure S 60: Molecular orientation of **3** used for all NMR related computations as an example, complexes **2**,**4** and **5** were oriented analogously. Mirror plane along the xz-axis shown in green.

Table S 37: Fitted mean values (for chemically equivalent atoms of the diamagnetic shift (δ_{dia}) fermi contact (δ_{FC}) and pseudo contact (δ_{PC}) components with both methods of the ^{1}H, ^{13}C. ^{19}F and ^{31}P chemical shifts, with the total calculated shift (δ_{obs}) and linewidth (FWHM) for **3**.

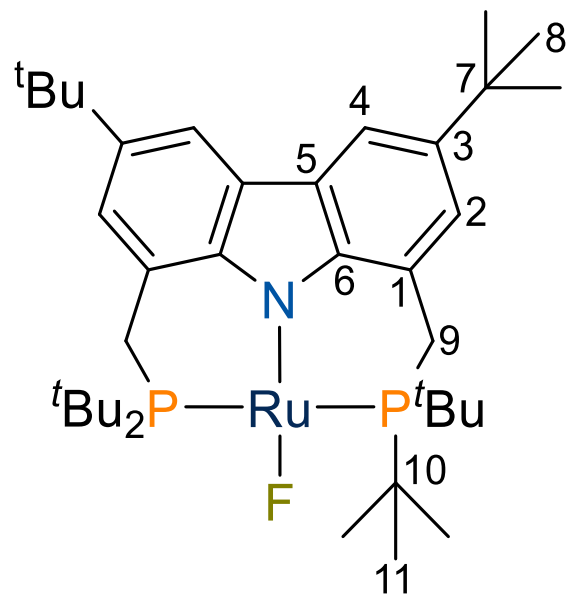

Group [ppm]	δ_{orb}	Calculation equation S7, S8 δ_{FC}	δ_{PC}	δ_{calc}	Calculation equation S10, S11 δ_{FC}	δ_{PC}	δ_{calc}	FWHM
H_2	7.74	14.49	-3.71	18.52	15.45	-1.29	21.90	0.02
H_4	9.05	17.84	13.80	40.69	19.03	3.66	31.74	0.02
H_8	1.58	1.42	1.87	4.87	1.51	0.45	3.54	0.00
H_9	2.40	44.39	-26.28	20.51	47.35	-7.68	42.07	0.25
H_{11}	0.40	6.65	-14.36	-7.31	7.09	-10.22	-2.73	0.19
C_1	131.64	232.72	17.26	381.62	248.25	38.84	418.74	0.32
C_2	137.00	-209.08	5.19	-66.89	-223.02	-11.77	-97.80	0.22
C_3	138.29	261.66	10.06	410.01	279.14	44.16	461.59	0.42
C_4	127.47	-238.50	20.45	-90.58	-254.42	-10.76	-137.71	0.28
C_5	133.60	123.90	53.53	311.02	132.18	70.91	336.68	0.24
C_6	162.79	-501.22	125.03	-213.41	-534.68	51.97	-319.92	1.21
C_7	39.01	-76.18	3.78	-33.39	-81.26	1.29	-40.96	0.02
C_8	34.05	86.57	2.32	122.95	92.35	0.65	127.06	0.04
C_9	25.92	-229.95	-47.14	-251.17	-245.30	-24.27	-243.66	0.27
C_{10}	39.58	-245.62	-61.07	-267.11	-262.01	-24.31	-246.75	0.32
C_{11}	30.35	163.28	-26.11	167.52	174.18	-18.60	185.93	0.42
F	40.14	3802.43	632.74	4475.31	4056.25	-125.68	3970.71	421.88
P	74.96	-3758.31	-297.63	-3980.97	-4009.19	38.02	-3896.20	88.11

Table S 38: Fitted susceptibility tensor (χ) for **3** in cm^{3} K mol^{-1}.

1.4965	0.0000	0.1862
0.0000	0.7999	0.0000
0.1862	0.0000	0.9126

Table S 39: Relaxation parameters used for the simulation of ^{1}H. ^{13}C, ^{19}F and ^{31}P signal linewidths for **3**

T_{1e}	2.18*10^{-9} s
T_{2e}	4.81*10^{-13} s
τ_R	2.26*10^{-7} s
r	7.164 Å
η (benzene)	0.630 mPa*s

Table S 40: Experimental chemical shifts and their corresponding weights used for the fitting procedure of the calculated **3** signals.

Group	δ_{exp}	Weight
H_2	11.9	1

H_4	28.9	1
H_8	4.7	1
H_9	20.0	1
H_{11}	-1.9	1
C_1	395.7	1
C_2	-76.0	1
C_3	444.6	1
C_4	-105.6	1
C_5	352.5	1
C_6	-342.1	1
C_7	-52.1	1
C_8	118.0	1
C_9	-311.4	1
C_{10}	-254.1	1
C_{11}	194.6	1
F	3922.5	0.2
P	-3891.7	0.2

Table S 41: Fitted mean values (for chemically equivalent atoms of the diamagnetic shift (δ_{dia}) fermi contact (δ_{FC}) and pseudo contact (δ_{PC}) components with both methods of the ^{1}H, ^{13}C. and ^{31}P chemical shifts, with the total calculated shift (δ_{obs}) and linewidth (FWHM) for **2**.

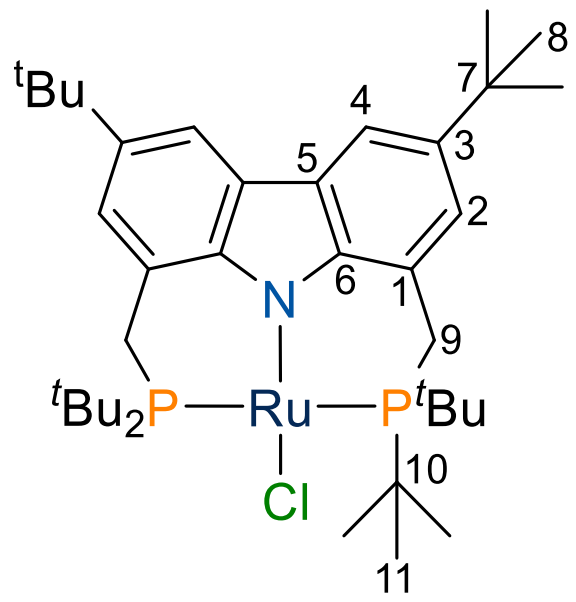

Group [ppm]	δ_{orb}	Calculation equation S7, S8 δ_{FC}	δ_{PC}	δ_{calc}	Calculation equation S10, S11 δ_{FC}	δ_{PC}	δ_{calc}	FWHM
H_2	7.50	16.54	-3.52	20.52	17.17	-8.21	16.46	0.02
H_4	9.25	20.17	12.82	42.24	20.94	2.78	32.98	0.02
H_8	1.45	1.50	1.66	4.61	1.56	-0.22	2.79	0.00
H_9	2.00	47.55	-22.61	26.95	49.37	-31.28	20.09	0.25
H_{11}	0.43	11.32	-16.71	-4.97	11.75	-4.69	7.48	0.17
C_1	130.98	270.96	16.73	418.66	281.27	27.25	439.50	0.39
C_2	132.11	-235.84	4.82	-98.91	-244.83	-27.49	-140.22	0.25
C_3	138.36	286.14	9.33	433.83	297.05	58.60	494.00	0.47
C_4	123.30	-249.43	19.09	-107.04	-258.93	-23.98	-159.62	0.29
C_5	141.75	178.43	50.42	370.60	185.23	82.24	409.22	0.30
C_6	165.00	-491.15	118.97	-207.17	-509.87	-15.57	-360.44	1.14
C_7	38.40	-83.11	3.45	-41.26	-86.27	-0.06	-47.93	0.03
C_8	31.58	94.35	2.08	128.01	97.95	-0.29	129.23	0.05
C_9	23.60	-224.44	-43.44	-244.28	-232.99	-55.77	-265.16	0.24
C_{10}	33.47	-239.01	-60.14	-265.68	-248.13	-48.45	-263.11	0.27
C_{11}	27.78	179.73	-27.93	179.59	186.58	-23.53	190.84	0.69
P	91.18	-3892.73	-286.84	-4088.38	-4041.10	78.75	-3871.16	88.33

Table S 42: Fitted susceptibility tensor (χ) for **2** in cm³ K mol⁻¹.

1.2565	0.0000	-0.0976
0.0000	0.3987	0.0000
-0.0976	0.0000	1.4676

Table S 43: Relaxation parameters used for the simulation of ^{1}H. ^{13}C, and ^{31}P signal linewidths for **2.**

T_{1e}	2.12*10^{-9} s
T_{2e}	4.47*10^{-13} s
τ_R	2.21*10^{-7} s
r	7.106 Å
η (benzene)	0.630 mPa*s

Table S 44: Experimental chemical shifts and their corresponding weights used for the fitting procedure of the calculated **2** signals.

Group	δ_{exp}	Weight
H_2	13.6	1.00
H_4	29.1	1.00
H_8	4.4	1.00
H_{11}	-6.2	1.00
C_1	446.0	1.00
C_2	-115.7	1.00
C_3	507.2	1.00
C_4	-132.2	1.00
C_5	411.1	1.00
C_6	-372.4	1.00
C_7	-65.9	1.00
C_8	129.8	1.00
C_9	-281.0	1.00
C_{10}	-240.0	1.00
C_{11}	178.0	1.00
P	-3896.0	0.20

Table S 45: Fitted mean values (for chemically equivalent atoms of the diamagnetic shift (δ_{dia}) fermi contact (δ_{FC}) and pseudo contact (δ_{PC}) components with both methods of the ^{1}H, ^{13}C. and ^{31}P chemical shifts, with the total calculated shift (δ_{obs}) and linewidth (FWHM) for **4**.

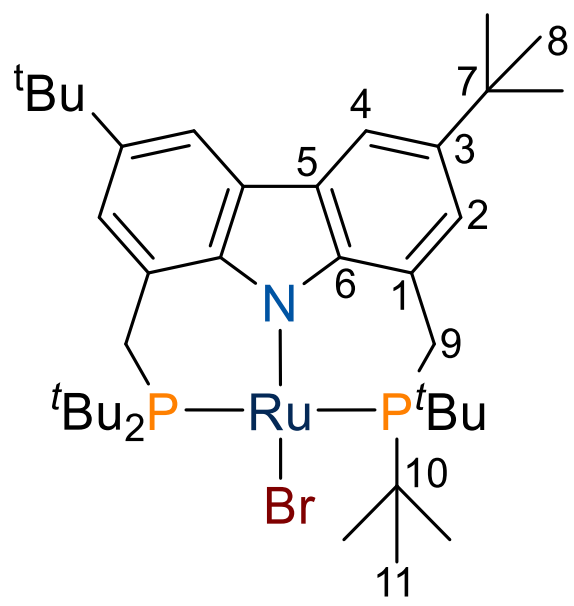

Group [ppm]	δ_{orb}	Calculation equation S7, S8 δ_{FC}	δ_{PC}	δ_{calc}	Calculation equation S10, S11 δ_{FC}	δ_{PC}	δ_{calc}	FWHM
H_2	7.62	16.79	-3.19	21.22	17.31	-7.45	17.48	0.02
H_4	9.38	20.39	12.83	42.60	21.02	8.00	38.40	0.01
H_8	1.51	1.52	1.69	4.72	1.56	1.01	4.08	0.00

H_9	2.20	47.82	-20.76	29.25	49.29	-42.08	9.40	0.21
H_{11}	0.43	11.04	-17.76	-6.30	11.38	-5.61	6.20	0.14
C_1	132.30	275.29	18.24	425.83	283.75	4.06	420.11	0.33
C_2	132.58	-237.72	5.25	-99.88	-245.03	-12.06	-124.52	0.21
C_3	140.30	287.02	9.50	436.82	295.85	32.43	468.58	0.39
C_4	124.27	-247.32	19.20	-103.84	-254.92	-3.98	-134.63	0.23
C_5	142.24	194.57	50.77	387.58	200.56	70.73	413.53	0.26
C_6	165.96	-478.28	120.80	-191.51	-492.98	16.48	-310.54	0.90
C_7	39.00	-83.39	3.51	-40.88	-85.96	1.57	-45.38	0.02
C_8	32.86	94.71	2.12	129.69	97.62	1.00	131.48	0.04
C_9	18.06	-220.30	-41.47	-243.71	-227.08	-67.10	-276.11	0.20
C_{10}	32.42	-235.36	-61.28	-264.23	-242.61	-56.44	-266.63	0.21
C_{11}	27.33	171.82	-29.03	170.12	177.11	-27.64	176.79	0.56
P	89.89	-3955.40	-287.62	-4153.13	-4076.99	98.20	-3888.91	74.62

Table S 46: Fitted susceptibility tensor (χ) for **4** in cm^3 K mol^{-1}.

1.3932	0.0000	-0.2823
0.0000	0.2516	0.0000
-0.2823	0.0000	1.4558

Table S 47: Relaxation parameters used for the simulation of ^{1}H. ^{13}C, and ^{31}P signal linewidths for **4.**

T_{1e}	$2.12*10^{-9}$ s
T_{2e}	$3.58*10^{-13}$ s
τ_R	$2.20*10^{-7}$ s
r	7.096 Å
η (benzene)	0.630 mPa*s

Table S 48: Experimental chemical shifts and their corresponding weights used for the fitting procedure of the calculated **4** signals.

Group	δ_{exp}	Weight
H_2	14.4	1.0
H_4	29.5	1.0
H_8	4.4	1.0
H_{11}	-8.0	1.0
C_1	464.5	1.0
C_2	-128.6	1.0
C_3	527.6	1.0
C_4	140.0	1.0
C_5	437.9	1.0
C_6	-376.4	1.0
C_7	-70.4	1.0
C_8	134.5	1.0
C_9	-274.1	1.0
C_{10}	-241.4	1.0
P	-3923.7	0.2

Table S 49: Fitted mean values (for chemically equivalent atoms of the diamagnetic shift (δ_{dia}) fermi contact (δ_{FC}) and pseudo contact (δ_{PC}) components with both methods of the ^{1}H, ^{13}C. and ^{31}P chemical shifts, with the total calculated shift (δ_{obs}) and linewidth (FWHM) for **5**.

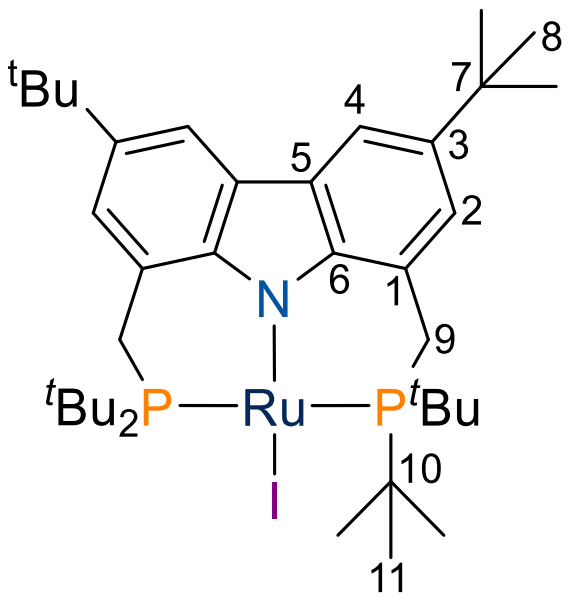

Group [ppm]	δ_{orb}	Calculation equation S7, S8 δ_{FC}	δ_{PC}	δ_{calc}	Calculation equation S10, S11 δ_{FC}	δ_{PC}	δ_{calc}	FWHM
H_2	7.41	17.50	-2.89	22.03	18.21	-9.97	15.66	0.05
H_4	9.15	21.08	12.01	42.24	21.94	2.55	33.64	0.04
H_8	1.50	1.51	1.46	4.47	1.57	-0.62	2.45	0.01
H_9	2.65	49.37	-15.91	36.11	51.38	-31.66	22.38	0.56
H_{11}	0.34	10.18	-18.98	-8.47	10.59	-9.56	1.38	0.34
C_1	131.41	285.72	19.38	436.50	297.37	51.82	480.61	0.89
C_2	131.06	-244.98	5.25	-108.68	-254.97	-42.00	-165.92	0.58
C_3	145.14	294.71	8.98	448.83	306.74	93.40	545.28	1.05
C_4	126.01	-249.48	18.21	-105.26	-259.67	-39.02	-172.68	0.61
C_5	134.74	220.95	48.98	404.67	229.96	122.28	486.98	0.77
C_6	169.81	-459.60	119.49	-170.30	-478.35	-24.09	-332.63	2.21
C_7	42.91	-85.45	3.20	-39.34	-88.94	-0.30	-46.34	0.06
C_8	39.61	96.96	1.87	138.45	100.92	-0.67	139.85	0.11
C_9	21.34	-198.48	-36.94	-214.08	-206.57	-67.23	-252.46	0.44
C_{10}	35.81	-222.93	-62.34	-249.46	-232.02	-59.30	-255.51	0.50
C_{11}	28.13	162.61	-30.17	160.57	169.24	-31.48	165.89	1.60
P	95.83	-4035.06	-285.92	-4225.15	-4199.70	105.38	-3998.50	199.43

Table S 50: Fitted susceptibility tensor (χ) for **5** in cm^3 K mol^{-1}.

1.3746	0.0000	-0.0016
0.0000	0.2401	0.0000
-0.0016	0.0000	1.5163

Table S 51: Relaxation parameters used for the simulation of ^{1}H. ^{13}C, and ^{31}P signal linewidths for **5.**

T_{1e}	$2.10*10^{-9}$ s
T_{2e}	$1.06*10^{-12}$ s
τ_R	$2.19*10^{-7}$ s
r	7.081 Å
η (benzene)	0.630 mPa*s

Table S 52: Experimental chemical shifts and their corresponding weights used for the fitting procedure of the calculated **5** signals.

Group	δ_{exp}	Weight
H_2	15.4	1
H_4	29.6	1
H_8	4.2	1
H_{11}	-13.8	1
C_1	487.2	1
C_2	-143.9	1

C_3	554.6	1
C_4	-148	1
C_5	474.8	1
C_6	-371.2	1
C_7	-75.4	1
C_8	138	1
C_9	-258.8	1
P	-3930.8	0.2

*6.4.2 Investigation of the influence of the N-Ru-X angle on A, for the Ru-Cl complex **2***

To investigate the influence of the N-Ru-X bond angle on the A_{iso}, and therefore as δ_{FC}, constrained optimizations of the Ru-Cl complex **2** at different angles have been performed and the A_{iso} values for the six ring carbons of the carbazole backbone as well as the P Atoms are listed below. As in these resonances δ_{calc} is dominated by δ_{FC} and they are conformationally rigid, these serve as a probe to investigate the degree in which the N-Ru-X influences the magnitude of chemical shift in these complexes.

Table S 53: Comparison if the A_{iso} values of carbazole nuclei C^1-C^6 and P of complex **2** at different angles.

Nuceleus	A_{iso} [MHz]			
	N-Ru-X [°]: 158 (Normal)	N-Ru-X [°]: 180 (Straight)	N-Ru-X [°]: 136 (bent down)	N-Ru-X [°]: 202 (bent up)
C^1	0.964	0.807	1.049	0.837
C^2	-0.839	-0.610	-0.951	-0.574
C^3	1.018	0.718	1.182	0.658
C^4	-0.888	-0.567	-1.017	-0.466
C^5	0.635	0.830	0.753	1.233
C^6	-1.748	-0.729	-1.973	-0.462
P	-22.319	-22.708	-22.390	-23.409

6.4.3 Assignment of the H^2/C^2 and H^4/C^4 resonances based on residual dipolar couplings (RDCs)

All resonances observed experimentally could be assigned unambiguously based on the prediction of the chemical shift mentioned above with the exception of H^2/C^2 and H^4/C^4. Even though a tentative assignment based on the predicted results is possible, the signals are in close proximity in the ^{13}C NMR, and para ^1H shifts are generally less pronounced, and are not reproduced as well theoretically compared to ^{13}C resonances. An approach not based on computations was thought out to validate the predicted results.
The RDC of a paramagnetic complex in solution in axial $\Delta\chi_{\mathrm{ax}}$ approximation is given by:[46]

$$\Delta\nu^{\mathrm{rdc}} = -\frac{B_0^2\gamma_A\gamma_B\hbar S_{LS}}{120\pi^2 kTr_{AB}^3}[\Delta\chi_{\mathrm{ax}}(3\cos^2\alpha - 1)] \qquad \text{(S12)}$$

Where B_0 is the magnetic field, γ_A and γ_B the gyromagnetic ratios of the two nuclei, S_{LS} as the generalized Lipari-Szabo order parameter, k as the Boltzmann constant, T as the Temperature, $\Delta\chi_{\mathrm{ax}}$ as the axial component of the magnetic susceptibility, r_{AB} as the bond vector between the two coupling nuclei A and B, and α as the angle between the AB vector and the main magnetic axis χ_{x}. The magnetic axes of complex **2** relative to the coordinate axes are shown in **Figure S61:**

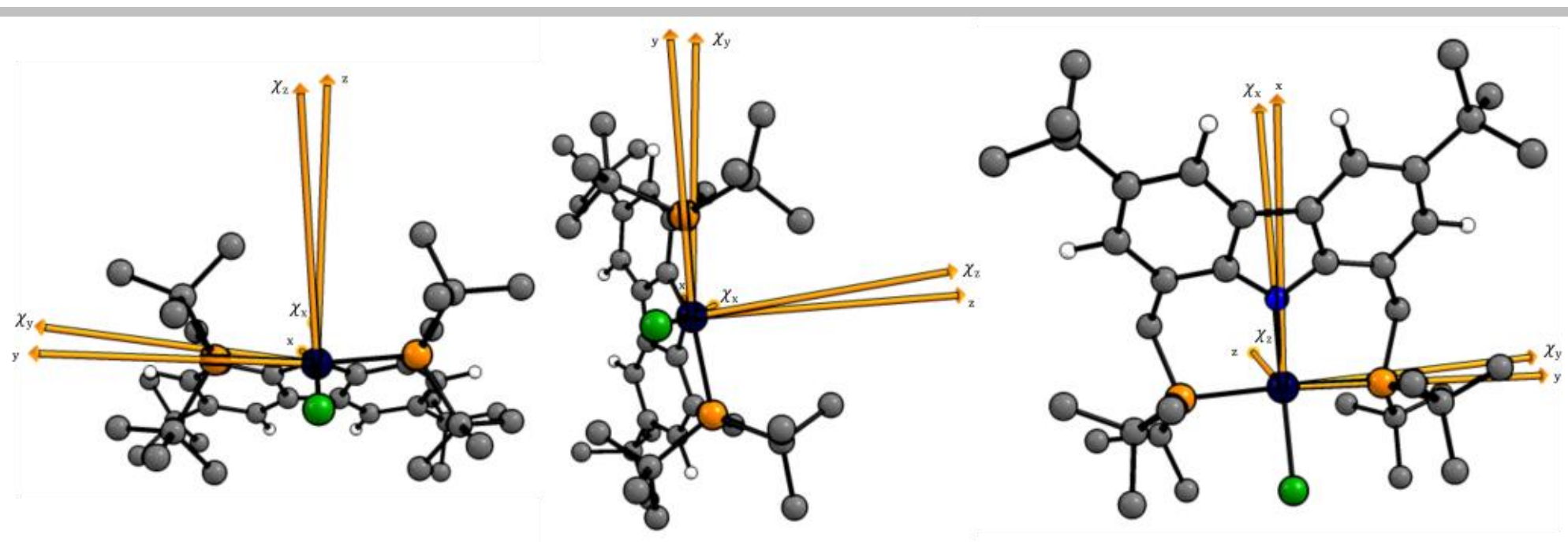

Figure S 61: The orientation of the *ab initio* CASSCF calculated magnetic axes χ_x, χ_y and χ_z relative to the coordinate axis *x,y* and *z*.

As shown, the magnetic axes (with the main magnetic axis χ_x) the magnetic axes align approximately with the reference coordinate system. For the absolute sign of the RDC changes above and below the magic angle of approximately 54.74°. The following angle is recorded for the angle between χ_x and the C^2-H^2/C^4-H^4 bond vector:

Table S 54: Angle between the bond vector and the main magnetic axis χ_x.

Vector	Angle
C^2-H^2	85.3°
C^4-H^4	49.3°

This results in an expected positive RDC for the coupling of C^2-H^2 and a negative RDC for the coupling of C^4-H^4. They add to the scalar spin-spin *J*-coupling and can be evaluated by the observed total signal splittings. Based on the couplings observed in ^{13}C-NMR the resonances of C^2 andC^4 could be assigned unambiguously confirming the assignment based on predicted shifts, and via 2D-NMR experiments (*vide supra*) the corresponding ^{1}H resonance could be assigned as well:

Table S 55: Observed *J*-couplings and chemical shifts for the resonances assigned to C^2-H^2/C^4-H^4 measured in C_6D_6, 600 MHz, 295 K.

Group	^{13}C shift [ppm]	^{1}H shift [ppm]	*J* coupling [Hz]
C^2-H^2	-115.7	13.6	158.5
C^4-H^4	-132.3	29.1	149.9

From the results obtained for **2** assignments for complexes **3**,**4** and **5** could be inferred. These results can be confirmed further when examining the temperature dependence of the observed *J*-coupling because of partial self-alignment in the magnetic field due to anisotropic magnetic susceptibility of the molecule. As expected, the observed coupling of C^2-H^2 decreases with temperature while the C^4-H^4 increases:

Table S 56: Temperature dependence of the observed *J*-coupling in **2** for C_2-H_2/C_4-H_4 measured in tol-d_8, 600 MHz.

T [K]	*J*-C^2-H^2 [Hz]	*J*-C^4-H^4 [Hz]
235	162.9	146.0
243	162.0	147.6
253	161.3	148.2
273	160.5	148.8
293	158.8	150.1
313	157.0	150.4
333	156.8	150.2

6.5 Cartesian coordinates of the Complexes

Cartesian coordinates (reported in Å) of complexes **2-5** S = 1.

2 r^{2}scan-3c/def2-mSVP/D(4)

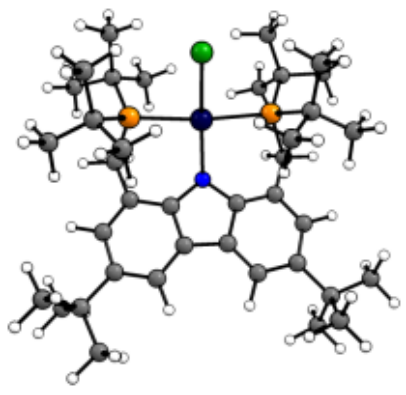

Ru	0.000000000	0.000000000	0.000000000
Cl	-2.235786883	0.079251128	-0.839219961
P	0.000000000	2.369458121	0.000000000
P	-0.217654683	-2.340041293	0.296692915
N	2.052203036	-0.101266260	-0.049611064
C	1.671543381	2.915458115	0.603027878
H	1.764463438	4.007007596	0.649324068
H	1.756320559	2.528528073	1.630296818
C	2.764830380	2.338529410	-0.242233384
C	3.735434686	3.176767398	-0.795891544
H	3.646619420	4.242965406	-0.589874183
C	4.818019043	2.724229599	-1.579391314
C	4.940894526	1.347213207	-1.771203673
H	5.770964088	0.929977491	-2.335651353
C	3.995436031	0.481686776	-1.213302755
C	2.877615643	0.956290940	-0.481818746
C	2.764703050	-1.265336985	-0.405666396
C	3.921460999	-0.954555942	-1.168663470
C	4.765434193	-1.948554245	-1.661428868
H	5.636144963	-1.664520893	-2.250957197
C	4.505872535	-3.294173926	-1.375912751
C	3.399425903	-3.581104952	-0.555262829
H	3.207477659	-4.609845622	-0.260161518
C	2.526561963	-2.604584024	-0.057078084
C	1.423753183	-2.983611613	0.882208822
H	1.600506782	-2.484123375	1.845119781
H	1.416926691	-4.066666084	1.059456178
C	5.795312003	3.744668405	-2.169641522
C	5.026560153	4.689539271	-3.116439606
H	4.556969802	4.120059579	-3.928650802
H	5.710370994	5.427758698	-3.558004343
H	4.236434610	5.235097698	-2.585686865
C	6.441708616	4.567718237	-1.037346393
H	5.692580077	5.123847182	-0.461085935
H	7.151823443	5.294439321	-1.455486875
H	6.983829208	3.911766456	-0.344403093
C	6.918099340	3.071067453	-2.971562635
H	7.515241382	2.400136755	-2.340594400
H	7.590254838	3.838586260	-3.376117657
H	6.519523892	2.494741821	-3.816491106
C	5.433765159	-4.384844521	-1.922083739
C	4.967360286	-5.797787800	-1.541404297
H	5.650398901	-6.537044520	-1.979223525
H	3.958547742	-6.005829488	-1.922109226
H	4.969499273	-5.946607869	-0.453810500
C	6.854056311	-4.178674042	-1.357545637
H	6.844303964	-4.237726523	-0.261733178
H	7.260006949	-3.199714005	-1.639406438
H	7.533045917	-4.952318193	-1.742290633
C	5.476467201	-4.294122532	-3.460674018
H	6.142204579	-5.067326650	-3.868535195
H	5.845926886	-3.317834408	-3.796009207
H	4.474317072	-4.439806888	-3.883959297
C	-1.123103214	3.051917474	1.400142792
C	-2.617279514	3.092491361	1.040657096
H	-2.859534545	3.932493174	0.384163410
H	-3.185833391	3.232117638	1.972419739
H	-2.954422461	2.164871003	0.568294683
C	-0.693673262	4.446944961	1.878920677
H	0.319459998	4.456211406	2.296014624
H	-1.380514100	4.773115902	2.673948056
H	-0.748971049	5.189726063	1.073726019
C	-0.959759753	2.040423840	2.553701333
H	-1.391519034	1.067992170	2.276380630
H	-1.506005959	2.408637564	3.434985275
H	0.085395822	1.886411011	2.851999773
C	-0.207922340	3.272393414	-1.675359447
C	-1.685029135	3.485985170	-2.042376677
H	-2.281463934	2.579212169	-1.899232123
H	-1.734345608	3.765205434	-3.105267957
H	-2.129254200	4.306535531	-1.469371537
C	0.514109644	4.627123907	-1.718526365
H	0.156310841	5.313293193	-0.940800064
H	0.311833547	5.097829893	-2.692011561
H	1.599507139	4.515547096	-1.626830442
C	0.415101432	2.328437805	-2.724203185
H	1.468500248	2.107373447	-2.517475322
H	0.355834693	2.812680945	-3.710637741
H	-0.140656745	1.383321995	-2.773584596
C	-1.352031098	-2.843636199	1.754914717
C	-0.731294696	-2.240479306	3.032375029
H	0.168534147	-2.776212848	3.355091419
H	-0.477124706	-1.178881767	2.901256218
C	-2.758622166	-2.242562494	1.601527541
H	-2.727000723	-1.148788196	1.585176016
H	-3.264314760	-2.569253292	0.689149641
C	-1.462018636	-4.364863501	1.936664394
H	-2.007231726	-4.839719701	1.113962482
H	-0.480666849	-4.846136838	2.030917503
C	-0.593922611	-3.365489525	-1.275298850
C	-2.103960745	-3.467052038	-1.544073854
H	-2.613635419	-4.108508381	-0.815813954
H	-2.243736291	-3.924159870	-2.535102432
H	-2.581439002	-2.481973182	-1.553399303
C	0.050079852	-2.589886557	-2.442986633
H	-0.412561996	-1.601766529	-2.558193397
H	-0.113698958	-3.154314235	-3.373462453
H	1.130788344	-2.462298310	-2.309646804
C	0.007485992	-4.778836919	-1.227309668
H	1.102002127	-4.752349878	-1.200659978
H	-0.286278748	-5.310785787	-2.144304467
H	-0.355331644	-5.364943527	-0.375357036
H	-3.364875782	-2.561699639	2.462510196
H	-1.466128151	-2.310351837	3.847012281
H	-2.019809436	-4.570134825	2.862633206

3 r^{2}scan-3c/def2-mSVP/D(4)

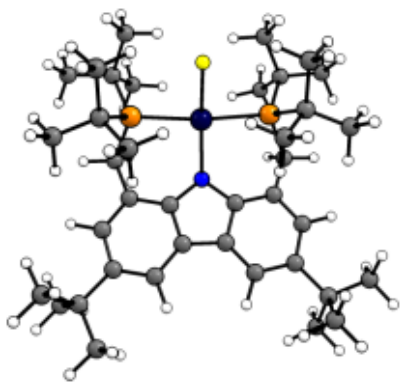

Ru	0.000000000	0.000000000	0.000000000
F	-1.904063372	0.052627489	-0.575571390
P	0.000000000	2.351173803	0.000000000
P	-0.242005052	-2.325050927	0.181574327
N	2.052654356	-0.104795441	-0.050984555
C	1.649513405	2.917091226	0.633410525
H	1.737481490	4.010091883	0.690734573
H	1.723573943	2.517214919	1.654831798
C	2.761756318	2.344656714	-0.195220840
C	3.735437868	3.191914723	-0.728365926
H	3.632132747	4.256971097	-0.522327342
C	4.836733623	2.751385449	-1.493499092
C	4.968854222	1.377522847	-1.696146649
H	5.807975534	0.969652422	-2.254162656
C	4.016740326	0.501708926	-1.164697726
C	2.887120690	0.961400662	-0.440770741
C	2.758840270	-1.262910657	-0.437749627
C	3.931956640	-0.933872597	-1.169665315
C	4.766140701	-1.914115269	-1.706523154
H	5.648468553	-1.612402282	-2.269634904
C	4.476039500	-3.268287413	-1.508327048
C	3.351004354	-3.580406999	-0.722393827
H	3.129294816	-4.621288952	-0.498829020
C	2.492584214	-2.619204031	-0.172346745
C	1.372256061	-3.053477543	0.726355448
H	1.550600688	-2.642506922	1.730094623
H	1.342362705	-4.147847548	0.810778526
C	5.821649138	3.781459021	-2.053756612
C	5.069642855	4.736801611	-3.003360121
H	4.617289311	4.176477105	-3.831614196
H	5.760242220	5.481905610	-3.422328094
H	4.268223984	5.274156722	-2.481306885
C	6.444742438	4.591447144	-0.899109492
H	5.682889498	5.136875274	-0.329268133
H	7.160226663	5.326009905	-1.293982310
H	6.975828877	3.927648650	-0.205061350
C	6.961101991	3.120347509	-2.842495600
H	7.547404488	2.442554640	-2.208727988
H	7.639288804	3.894193149	-3.224455674
H	6.579913966	2.553791225	-3.701970179
C	5.386935230	-4.343812524	-2.110550135
C	4.884487192	-5.766678938	-1.824161087
H	5.557537930	-6.493011129	-2.297707467
H	3.877141237	-5.926950242	-2.230962055
H	4.866329533	-5.982287675	-0.747953413
C	6.803959298	-4.204117603	-1.518549239
H	6.779609764	-4.330716035	-0.428679575
H	7.231904938	-3.217784558	-1.734426701
H	7.472238037	-4.966111353	-1.943400257
C	5.451708354	-4.160540286	-3.640171108
H	6.102593243	-4.924746679	-4.087276085
H	5.850468521	-3.175830863	-3.911261313
H	4.451589980	-4.253663618	-4.082730539
C	-1.220288600	3.080207159	1.276938523
C	-2.663929124	2.628010698	0.988396196
H	-3.099719192	3.158647804	0.137815017
H	-3.281629486	2.855691131	1.869805814
H	-2.718996947	1.555445509	0.777923618
C	-1.170816386	4.610706182	1.376621639
H	-0.160295217	4.986757032	1.580796607
H	-1.819257594	4.935830942	2.204125161
H	-1.543543340	5.088732550	0.463284868
C	-0.816195755	2.473590870	2.636905058
H	-0.801541236	1.374497744	2.596158765
H	-1.560423130	2.769494584	3.389742973
H	0.162068403	2.822766097	2.985873082
C	-0.184181148	3.158091110	-1.716218325
C	-1.668457671	3.211415939	-2.110390677
H	-2.163375572	2.248358365	-1.932284852
H	-1.736513546	3.442530215	-3.183632703
H	-2.208886266	3.998304085	-1.571750582
C	0.442591305	4.556096130	-1.806405067
H	0.019598449	5.253911206	-1.074974949
H	0.251152712	4.966882567	-2.809014994
H	1.529528765	4.516413334	-1.669523726
C	0.531568327	2.222205216	-2.712640461
H	1.597843994	2.109555812	-2.490349684
H	0.435249357	2.646721546	-3.723281693
H	0.068047668	1.226467455	-2.719029503
C	-1.432972055	-2.844064363	1.580416841
C	-0.807844262	-2.335033354	2.895911671
H	0.075659955	-2.911255079	3.192867638
H	-0.526263865	-1.274119252	2.826469519
C	-2.793320428	-2.143071310	1.420810508
H	-2.677114137	-1.057600243	1.343580095
H	-3.337276392	-2.477401821	0.534010959
C	-1.631813263	-4.362550080	1.683860069
H	-2.174837693	-4.764730058	0.821430168
H	-0.678480527	-4.898486328	1.776465862
C	-0.653538177	-3.187871020	-1.466900749
C	-2.162257402	-3.109092477	-1.746374570
H	-2.736940141	-3.779843585	-1.097024941
H	-2.342274607	-3.423045448	-2.785304511
H	-2.530974065	-2.083602917	-1.624810294
C	0.070336026	-2.374730567	-2.559676972
H	-0.311223874	-1.345416687	-2.596037909
H	-0.126322530	-2.842534292	-3.536342572
H	1.155086272	-2.343695551	-2.407833434
C	-0.172799330	-4.644287729	-1.528511569
H	0.920884559	-4.706904175	-1.481861357
H	-0.487053458	-5.079291901	-2.489105997
H	-0.596522429	-5.263847340	-0.729850874
H	-3.408520513	-2.374555600	2.302923431
H	-1.550917717	-2.432875965	3.700007113
H	-2.227144629	-4.586621549	2.581808083

4 r^{2}scan-3c/def2-mSVP/D(4)

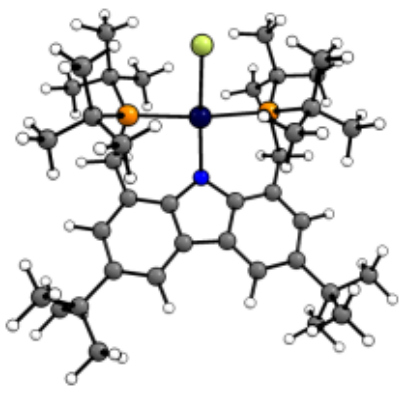

Ru	0.000000000	0.000000000	0.000000000
Br	-2.346560485	0.057528353	-0.925221005
P	0.000000000	2.378362848	0.000000000
P	-0.187316971	-2.346736739	0.339318046

N	2.058254895	-0.087700739	-0.043029467
C	1.669025140	2.910864220	0.626245989
H	1.760305781	4.001261553	0.693611429
H	1.745905245	2.503963717	1.646513289
C	2.766811349	2.350424002	-0.222207074
C	3.738870245	3.194532514	-0.763901798
H	3.652505208	4.258108466	-0.544029084
C	4.820698622	2.749273465	-1.552637270
C	4.942613822	1.374152281	-1.760188212
H	5.773897622	0.962856368	-2.327189030
C	3.997739109	0.502919937	-1.210381984
C	2.879646457	0.971994246	-0.476144178
C	2.779211576	-1.247712227	-0.394101711
C	3.931048752	-0.933863697	-1.163017214
C	4.782918184	-1.925955128	-1.645590717
H	5.649027787	-1.641630448	-2.241704673
C	4.540063118	-3.269964308	-1.337164729
C	3.442533224	-3.556801094	-0.504577846
H	3.267768155	-4.582039388	-0.187614988
C	2.559572437	-2.582262703	-0.020694162
C	1.467624179	-2.948529174	0.934717819
H	1.643468065	-2.418757196	1.881441553
H	1.475607667	-4.025796732	1.143220572
C	5.800529459	3.775150503	-2.129108426
C	5.034720827	4.733234755	-3.064954182
H	4.565961898	4.175344189	-3.885671742
H	5.720319526	5.476321558	-3.495408862
H	4.244222380	5.272861749	-2.528684621
C	6.447100773	4.582596000	-0.985662503
H	5.698715552	5.133447549	-0.403402777
H	7.159692173	5.312598095	-1.393763096
H	6.986715105	3.916538508	-0.300464788
C	6.923276619	3.109608211	-2.937731117
H	7.519553013	2.431580861	-2.313546285
H	7.596191563	3.881090750	-3.333363047
H	6.525078672	2.542653130	-3.789166347
C	5.477101028	-4.358211401	-1.872298351
C	5.037859391	-5.769267106	-1.454391760
H	5.726575091	-6.507475065	-1.885009899
H	4.027411610	-6.001882800	-1.816115409
H	5.057280255	-5.893206145	-0.363824304
C	6.902048495	-4.119693094	-1.332755149
H	6.908340003	-4.154451549	-0.235886489
H	7.291125550	-3.142206582	-1.642066767
H	7.586109499	-4.892650182	-1.709740082
C	5.496435236	-4.299510187	-3.412965619
H	6.169538957	-5.069751451	-3.814312152
H	5.843856408	-3.324134933	-3.773742537
H	4.491013749	-4.471012168	-3.818497940
C	-1.124655937	3.059751766	1.401604610
C	-2.611268472	3.152413817	1.022743551
H	-2.820132865	4.019639073	0.390280133
H	-3.191230544	3.276580553	1.949580315
H	-2.965880440	2.250105207	0.514770893
C	-0.666189833	4.431784678	1.919396456
H	0.335619356	4.403618348	2.362080995
H	-1.363693902	4.761174943	2.703661841
H	-0.679600444	5.192072255	1.129016924
C	-1.001876169	2.019922776	2.534546338
H	-1.456406544	1.065397527	2.232875320
H	-1.546760232	2.383864061	3.418516731
H	0.036516516	1.833299787	2.838960792
C	-0.180057505	3.312844618	-1.663723493
C	-1.648949694	3.548523632	-2.049541142
H	-2.258141027	2.646256380	-1.934368371
H	-1.677523776	3.848062652	-3.107611395
H	-2.094099722	4.362766203	-1.468380792
C	0.550327884	4.664180792	-1.672094080
H	0.186709585	5.338313241	-0.886700419
H	0.361563148	5.153685266	-2.639018784
H	1.633880534	4.546106301	-1.570908512
C	0.453080548	2.385471804	-2.721338490
H	1.503030163	2.158314694	-2.504433211
H	0.408347203	2.888472545	-3.699028528
H	-0.103542061	1.442585629	-2.796099856
C	-1.299461120	-2.841072783	1.820182798
C	-0.688828219	-2.184437939	3.075807306
H	0.227952614	-2.684507962	3.407815710
H	-0.466315221	-1.120262915	2.912771807
C	-2.724284855	-2.287480490	1.661426312
H	-2.725712373	-1.193708140	1.622004864
H	-3.224423162	-2.647974870	0.758622606
C	-1.366039415	-4.359078795	2.046133556
H	-1.902797104	-4.872573417	1.241167725
H	-0.372237152	-4.811768724	2.149928678
C	-0.545368965	-3.433417239	-1.198066633
C	-2.052843672	-3.588245177	-1.455719134
H	-2.540927138	-4.221298663	-0.705589986
H	-2.182551274	-4.079964548	-2.431422301
H	-2.560795225	-2.619185339	-1.494322589
C	0.073663998	-2.682325345	-2.394872656
H	-0.412570752	-1.710014557	-2.541876401
H	-0.080128880	-3.282998570	-3.304050509
H	1.151608913	-2.524351364	-2.271645232
C	0.092540940	-4.828770212	-1.106493097
H	1.185963171	-4.774671546	-1.094476395
H	-0.197849445	-5.400130340	-2.000613142
H	-0.246215229	-5.393516114	-0.230664759
H	-3.317173201	-2.605536653	2.532084828
H	-1.416521344	-2.251583247	3.897076035
H	-1.913686677	-4.550992488	2.980932273

5 r^{2}scan-3c/def2-mSVP/D(4)

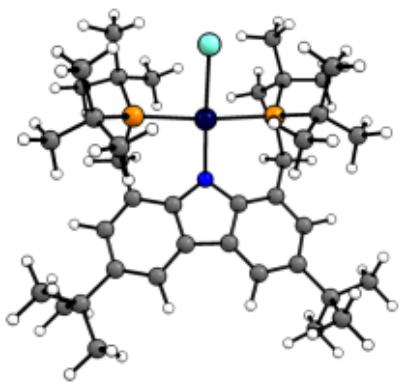

Ru	0.000000000	0.000000000	0.000000000
I	-2.443182058	-0.096266000	-1.175456970
P	-0.081575524	2.389076667	-0.027869972
P	-0.068573053	-2.343676895	0.466908463
N	2.062942012	0.000000000	0.000000000
C	1.564513699	2.962199233	0.633833711
H	1.618316854	4.053140162	0.723631597
H	1.643071924	2.534623776	1.645377880
C	2.683762720	2.450724061	-0.216824654
C	3.626231330	3.323165105	-0.765822947
H	3.512979744	4.383827755	-0.544248338
C	4.712183507	2.906180592	-1.564895780
C	4.879707207	1.533763670	-1.762573447
H	5.721971223	1.147520759	-2.330843868
C	3.971911599	0.635072339	-1.194942926
C	2.841199020	1.077468299	-0.464345628

C	2.835242544	-1.136776691	-0.312360268
C	3.966594736	-0.802260638	-1.101833798
C	4.862557215	-1.775893846	-1.540192328
H	5.710218320	-1.482000158	-2.157813430
C	4.690556454	-3.112006548	-1.155391886
C	3.616792256	-3.408847473	-0.295240826
H	3.501942202	-4.420672074	0.085145754
C	2.686149654	-2.455383634	0.137255386
C	1.612912119	-2.804394578	1.118533673
H	1.754523650	-2.190716684	2.019106820
H	1.675733074	-3.859706599	1.411803712
C	5.654251694	3.958573720	-2.157336951
C	4.845183313	4.910168149	-3.062629205
H	4.365734554	4.351903567	-3.876977893
H	5.505754266	5.670018965	-3.502575179
H	4.059146587	5.430204779	-2.501143228
C	6.312259229	4.766871720	-1.021165493
H	5.565426267	5.286959525	-0.409353712
H	6.992481420	5.522200562	-1.438318114
H	6.889207190	4.105497664	-0.362336276
C	6.768445331	3.326116331	-3.003259530
H	7.404423631	2.663547080	-2.402021844
H	7.406609203	4.116759256	-3.418323202
H	6.358010965	2.750279601	-3.842894440
C	5.673519196	-4.180882354	-1.646010914
C	5.319904025	-5.583307763	-1.129891110
H	6.040840417	-6.310608290	-1.524713346
H	4.317856421	-5.892272075	-1.456138056
H	5.362734774	-5.633417872	-0.034083955
C	7.096747352	-3.835352794	-1.163684093
H	7.134769295	-3.801462067	-0.067397062
H	7.424431893	-2.860284841	-1.543349355
H	7.811108894	-4.593280659	-1.513792282
C	5.649717844	-4.216826973	-3.187468798
H	6.349686869	-4.976662183	-3.561831255
H	5.938284888	-3.249320899	-3.615291905
H	4.643633418	-4.460731345	-3.552082884
C	-1.244375160	3.027118459	1.367664007
C	-2.727487539	3.088365341	0.968978521
H	-2.948349917	3.957908063	0.344133228
H	-3.324391514	3.185134311	1.888170315
H	-3.056474952	2.186301119	0.443427183
C	-0.831885478	4.404572137	1.909042520
H	0.160247328	4.397559480	2.374014661
H	-1.554725256	4.708002044	2.680775520
H	-0.847264931	5.172644308	1.126552153
C	-1.110603144	1.978360075	2.489494353
H	-1.516587405	1.009562744	2.163278842
H	-1.694413613	2.305289653	3.363201152
H	-0.074128397	1.830814279	2.821031415
C	-0.254060992	3.366873239	-1.671067232
C	-1.718767874	3.602178962	-2.072520735
H	-2.328543381	2.696785711	-1.994100820
H	-1.732164381	3.929990265	-3.122463709
H	-2.177704739	4.398776589	-1.477186760
C	0.452892064	4.730411535	-1.631988221
H	0.069834301	5.378476551	-0.833981329
H	0.266143715	5.241538957	-2.588080348
H	1.536408245	4.629487476	-1.522881372
C	0.413076288	2.483037557	-2.745097868
H	1.467768044	2.283936986	-2.522966536
H	0.361917950	3.009080571	-3.710133794
H	-0.112017524	1.525386179	-2.848473251
C	-1.159084063	-2.790903983	1.981705719
C	-0.586808435	-2.023850031	3.192076914
H	0.347019890	-2.462107847	3.562077100
H	-0.405295876	-0.965080612	2.960839536
C	-2.609310484	-2.325404574	1.781334940
H	-2.669797215	-1.238056112	1.668068823
H	-3.082371583	-2.773177350	0.902776326
C	-1.152467475	-4.290856350	2.315527144
H	-1.668268193	-4.885198832	1.554155862
H	-0.138420589	-4.688570225	2.443807563
C	-0.337670591	-3.590856703	-0.969444076
C	-1.827823370	-3.868430362	-1.223212678
H	-2.286693825	-4.448262215	-0.413960633
H	-1.911089048	-4.467525651	-2.142187914
H	-2.397827605	-2.945727105	-1.369389687
C	0.259491316	-2.925693900	-2.225966531
H	-0.257910737	-1.989090879	-2.465552646
H	0.137261825	-3.612301925	-3.077164143
H	1.329468783	-2.716677961	-2.110159580
C	0.378464306	-4.931991329	-0.739837525
H	1.466318756	-4.815775632	-0.721732646
H	0.134756082	-5.596486421	-1.581878468
H	0.056895114	-5.435103464	0.178337172
H	-3.193242045	-2.612851888	2.668599648
H	-1.317936375	-2.065552215	4.012001506
H	-1.686732267	-4.440050481	3.265772867

Cartesian coordinates (reported in Å) of complexes **2-5** S = 0

2 r^{2}scan-3c/def2-mSVP/D(4)

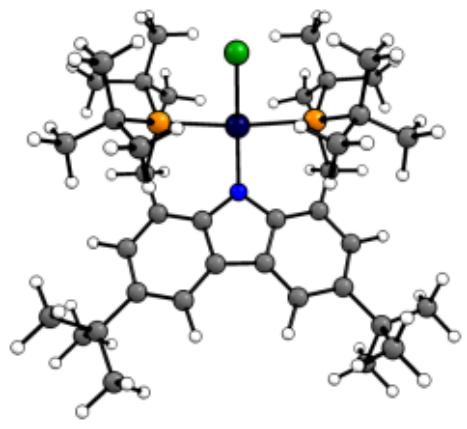

Ru	0.000000000	0.000000000	0.000000000
I	-2.289541239	-0.047406776	-1.410487217
P	0.000000000	2.381070314	0.000000000
P	-0.170313092	-2.333585612	0.460756546
N	2.028437117	-0.073661083	0.000473207
C	1.666052419	2.888314757	0.661891823
H	1.763431445	3.973945933	0.784923410
H	1.743768003	2.421694472	1.654135390
C	2.757674665	2.348525338	-0.207665931
C	3.742051702	3.180501674	-0.746016988
H	3.680928821	4.243081016	-0.514391434
C	4.809738138	2.717411910	-1.543747486
C	4.916575164	1.338817149	-1.748570984
H	5.745630160	0.918824973	-2.312048111
C	3.967862308	0.480411330	-1.187556132
C	2.855496732	0.972358597	-0.465263021
C	2.768077044	-1.242599113	-0.287885484
C	3.910122076	-0.957728992	-1.077663660
C	4.774352087	-1.966889767	-1.495861525
H	5.632197997	-1.712939432	-2.116658974
C	4.556889835	-3.289701964	-1.084481289
C	3.475635230	-3.534767596	-0.218531785
H	3.328705458	-4.534832452	0.181015085
C	2.573862056	-2.543535196	0.191486024
C	1.476999710	-2.838089041	1.165566890
H	1.620857552	-2.206382930	2.053498773

H	1.501320965	-3.887036180	1.485409961
C	5.811374058	3.726147851	-2.113876614
C	5.059961677	4.759286217	-2.977607069
H	4.534735607	4.263472746	-3.804037784
H	5.766925718	5.486240753	-3.400558023
H	4.317765208	5.313580780	-2.390310983
C	6.529033780	4.448516532	-0.955839885
H	5.821725607	4.994872631	-0.320242272
H	7.256562043	5.170990885	-1.350941111
H	7.063313073	3.726886639	-0.324968550
C	6.874810451	3.051448511	-2.992068519
H	7.480380129	2.338129275	-2.418126333
H	7.552701163	3.814102068	-3.396058256
H	6.419855833	2.522260402	-3.839459718
C	5.501610745	-4.399759324	-1.556267039
C	5.093171701	-5.782633986	-1.028171055
H	5.785067230	-6.539859563	-1.418473172
H	4.079827111	-6.055700886	-1.351559254
H	5.135282311	-5.827924373	0.067927762
C	6.933742868	-4.102542952	-1.068483101
H	6.966109539	-4.057643644	0.027595198
H	7.302017372	-3.145416625	-1.456700057
H	7.620589868	-4.891576064	-1.404626749
C	5.484612379	-4.452508332	-3.097683709
H	6.156072771	-5.243866802	-3.458368244
H	5.813084671	-3.502946130	-3.536747756
H	4.471671124	-4.661517055	-3.464997190
C	-1.158758068	3.111295747	1.345755999
C	-2.618602940	2.691022215	1.112803914
H	-3.013315355	3.024107755	0.149103685
H	-3.238282451	3.133705598	1.907030371
H	-2.730570296	1.602457506	1.156613798
C	-1.079138741	4.640977728	1.460128773
H	-0.052376354	4.994411172	1.614662562
H	-1.670145041	4.958722457	2.332206416
H	-1.495469126	5.145835541	0.582074297
C	-0.719763211	2.498586644	2.690679919
H	-0.636729585	1.404828639	2.622320944
H	-1.479522001	2.732071782	3.450320520
H	0.236055504	2.899746527	3.046512187
C	-0.137146214	3.386396635	-1.633855646
C	-1.598181994	3.678840243	-2.013851603
H	-2.205762936	2.769274340	-2.050015653
H	-1.602930145	4.127840064	-3.018237829
H	-2.067764962	4.398872852	-1.333905202
C	0.623751019	4.721811551	-1.579559231
H	0.281455372	5.376116725	-0.770264517
H	0.450900534	5.252051008	-2.527811817
H	1.703457791	4.572199172	-1.485646091
C	0.488935464	2.509828621	-2.737227124
H	1.543371215	2.287010585	-2.538362547
H	0.430983752	3.056002131	-3.690908042
H	-0.052899007	1.563930168	-2.848559386
C	-1.325731058	-2.682271889	1.951272477
C	-0.767964548	-1.886664570	3.148636834
H	0.144527564	-2.331657361	3.562070495
H	-0.554496760	-0.843795375	2.874845818
C	-2.742510990	-2.156334271	1.673427898
H	-2.738006398	-1.072011519	1.516325036
H	-3.204856556	-2.614447265	0.794390600
C	-1.395480198	-4.164035559	2.349060549
H	-1.902505451	-4.773903283	1.594347528
H	-0.403048634	-4.591800140	2.538049898
C	-0.450876270	-3.624801674	-0.937006509
C	-1.944206392	-3.848742883	-1.227705553
H	-2.451345122	-4.375420417	-0.410994133
H	-2.024512606	-4.481526501	-2.124114416
H	-2.469625929	-2.910655053	-1.430718700
C	0.214378431	-3.042453004	-2.200272429
H	-0.235530907	-2.083496730	-2.480019964
H	0.066887815	-3.749374198	-3.030772576
H	1.292308837	-2.896961680	-2.066011839
C	0.191200542	-4.989131503	-0.632246833
H	1.282667427	-4.927186089	-0.584669270
H	-0.059777668	-5.673993434	-1.455686870
H	-0.181823206	-5.438984632	0.293977043
H	-3.374938425	-2.375376863	2.546788950
H	-1.522570400	-1.877951967	3.948168999
H	-1.972999640	-4.249707797	3.281763012

3 r^{2}scan-3c/def2-mSVP/D(4)

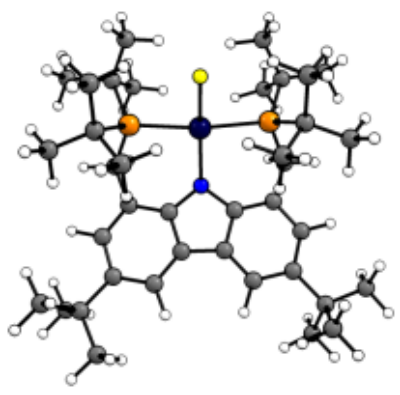

Ru	0.000000000	0.000000000	0.000000000
F	-1.792952078	0.058335206	-0.801237347
P	0.000000000	2.341816913	0.000000000
P	-0.273295829	-2.312227806	0.205485425
N	2.008780721	-0.117396750	0.000670559
C	1.635895014	2.908189493	0.667482356
H	1.729116516	4.000737767	0.725413724
H	1.688912519	2.505712533	1.689136639
C	2.753891175	2.323618723	-0.144396069
C	3.747500359	3.156289532	-0.666300425
H	3.659905373	4.222287532	-0.458905347
C	4.847447888	2.697300609	-1.420766686
C	4.964979185	1.319737509	-1.616094922
H	5.806713812	0.900140226	-2.160974314
C	3.997253233	0.460606667	-1.088872222
C	2.865289331	0.940892754	-0.388796300
C	2.728133329	-1.289735826	-0.341437918
C	3.908156713	-0.978313450	-1.063228044
C	4.754618135	-1.970172287	-1.554524084
H	5.641481227	-1.683466236	-2.117825251
C	4.470676532	-3.318896700	-1.305434291
C	3.334865052	-3.609625095	-0.528536656
H	3.114560878	-4.642737008	-0.271235256
C	2.459154552	-2.633832870	-0.032361980
C	1.308837232	-3.036881546	0.841471765
H	1.448141947	-2.585501043	1.834135870
H	1.272908759	-4.127336194	0.966150150
C	5.860386089	3.708876116	-1.965064591
C	5.134249024	4.726479852	-2.867768554
H	4.642194154	4.216843416	-3.706162582
H	5.851660110	5.452376697	-3.274776214
H	4.368206694	5.284109759	-2.315216693
C	6.529343341	4.449288070	-0.789572223
H	5.793863420	4.992299118	-0.183796807
H	7.262367626	5.176691672	-1.165163336
H	7.049064894	3.739040427	-0.134065568
C	6.960512800	3.033872265	-2.796588778
H	7.546435436	2.326976349	-2.194936236

H	7.649963346	3.797195414	-3.179305629
H	6.541186046	2.497027666	-3.657470255
C	5.390899289	-4.409778967	-1.863268082
C	4.947506117	-5.820617053	-1.449427794
H	5.637721137	-6.559360295	-1.876443800
H	3.939817512	-6.051813970	-1.819081589
H	4.958183458	-5.943389242	-0.358597683
C	6.827862550	-4.188539322	-1.349423988
H	6.857181506	-4.231168672	-0.253183503
H	7.222446284	-3.213939670	-1.660237885
H	7.496117840	-4.965389399	-1.745891069
C	5.380327200	-4.334982431	-3.403614796
H	6.039983864	-5.105200765	-3.826923198
H	5.725059121	-3.356813975	-3.759971675
H	4.365349820	-4.496256811	-3.789027310
C	-1.249983138	3.068578890	1.250490317
C	-2.672613951	2.562811509	0.947806552
H	-3.101371227	3.028405777	0.056657870
H	-3.321294551	2.814508975	1.799785899
H	-2.690119322	1.478790183	0.796165694
C	-1.242816565	4.601394590	1.314702057
H	-0.243273294	5.001971164	1.527800005
H	-1.911179581	4.930651107	2.124640079
H	-1.608136239	5.051906246	0.384836926
C	-0.852691604	2.506176018	2.630572787
H	-0.779265523	1.409263590	2.605337357
H	-1.629319382	2.779723821	3.359052020
H	0.097631501	2.911502035	2.996784553
C	-0.150849947	3.171088581	-1.711637355
C	-1.628419133	3.238629521	-2.127739635
H	-2.123800577	2.270280450	-1.985316746
H	-1.679912420	3.499001700	-3.195409222
H	-2.177627851	4.010736818	-1.576484516
C	0.488092118	4.565056035	-1.777540049
H	0.065436835	5.259576418	-1.042686666
H	0.312733902	4.989290602	-2.777584656
H	1.572997827	4.511671738	-1.629545163
C	0.571150415	2.241947770	-2.709455767
H	1.637088911	2.131763486	-2.482829709
H	0.478689252	2.673246721	-3.717809910
H	0.111752252	1.245144472	-2.717014801
C	-1.534448631	-2.791019365	1.554928878
C	-0.945044436	-2.331229861	2.903827827
H	-0.115361730	-2.965357112	3.237660662
H	-0.595992551	-1.289576238	2.856189802
C	-2.842997408	-2.010286065	1.341411960
H	-2.662283125	-0.930134982	1.353867795
H	-3.333040849	-2.251011158	0.394697376
C	-1.822575455	-4.295918071	1.634843961
H	-2.349632983	-4.663726975	0.748015312
H	-0.905424615	-4.885000424	1.765794382
C	-0.620512052	-3.218798556	-1.437386973
C	-2.110076548	-3.107643810	-1.801025511
H	-2.746174747	-3.721933873	-1.153125361
H	-2.243811427	-3.470131761	-2.831179164
H	-2.445257195	-2.065076738	-1.754766944
C	0.176520892	-2.460360150	-2.519601198
H	-0.153657271	-1.415675013	-2.584189822
H	-0.007574085	-2.941719304	-3.492231442
H	1.255674098	-2.473045030	-2.331776691
C	-0.178650009	-4.688760845	-1.432804818
H	0.906628009	-4.780400846	-1.304464464
H	-0.433536459	-5.136197019	-2.405242115
H	-0.677439499	-5.279328366	-0.656336352
H	-3.535519146	-2.258992563	2.159244685
H	-1.732542028	-2.389495042	3.668852193
H	-2.467183607	-4.490217322	2.505365601

4 r^{2}scan-3c/def2-mSVP/D(4)

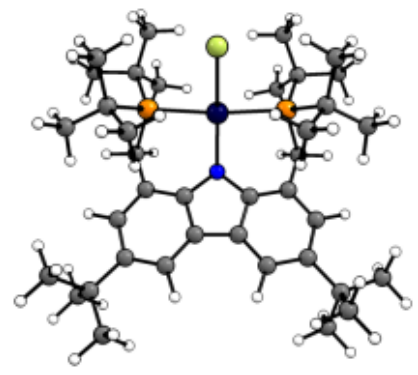

Ru	0.000000000	0.000000000	0.000000000
Br	-2.156696579	0.002838771	-1.263370589
P	0.000000000	2.370129644	0.000000000
P	-0.207400355	-2.328125108	0.391496865
N	2.021542664	-0.090396876	0.001392406
C	1.660928583	2.892720903	0.656476499
H	1.759610804	3.980660077	0.759387616
H	1.735188223	2.445543437	1.657887187
C	2.755341963	2.336640834	-0.201014183
C	3.738601951	3.166663231	-0.745059435
H	3.670299921	4.231319959	-0.525102669
C	4.810230826	2.701390279	-1.535878440
C	4.918995776	1.322170747	-1.733093543
H	5.747838541	0.900211304	-2.295471880
C	3.968202531	0.465971806	-1.172351981
C	2.855494308	0.957740141	-0.450330625
C	2.750611129	-1.262768502	-0.305365895
C	3.899364755	-0.972800154	-1.083417258
C	4.752056721	-1.980591562	-1.527837869
H	5.614515172	-1.719759753	-2.139351616
C	4.513610384	-3.312074601	-1.159507351
C	3.421615172	-3.568209204	-0.310609704
H	3.252871539	-4.579252330	0.051107998
C	2.532725831	-2.577404654	0.128131035
C	1.422981170	-2.899293396	1.080086033
H	1.572446896	-2.315799541	1.999427278
H	1.426771616	-3.963442167	1.347735380
C	5.807989140	3.709212647	-2.114319039
C	5.053926819	4.718476675	-3.003551550
H	4.542149790	4.201902228	-3.825703837
H	5.756879925	5.445434609	-3.433219776
H	4.299903439	5.274772567	-2.433349334
C	6.508308477	4.459986985	-0.963900261
H	5.790217692	5.009867751	-0.343768878
H	7.230928181	5.183317616	-1.366339854
H	7.045640658	3.755916482	-0.315978662
C	6.885930266	3.029405762	-2.970517310
H	7.489182341	2.328059321	-2.379532888
H	7.563405688	3.790874315	-3.377427451
H	6.444848355	2.485420563	-3.815869520
C	5.442533833	-4.420645840	-1.665067166
C	5.031050042	-5.809812534	-1.156073169
H	5.717700470	-6.563800544	-1.561565066
H	4.015186002	-6.073572126	-1.478916966
H	5.078192028	-5.870601396	-0.060961450
C	6.884447420	-4.144113329	-1.193941614
H	6.934331358	-4.120024008	-0.097867204
H	7.255542501	-3.183222539	-1.569922478
H	7.559370856	-4.932176946	-1.555458356
C	5.401618928	-4.443760290	-3.206686824

H	6.064217277	-5.230629395	-3.592887809
H	5.725895781	-3.486498034	-3.632135587
H	4.382096716	-4.642372272	-3.561405107
C	-1.176540142	3.100915075	1.326423492
C	-2.628305732	2.653717370	1.089336203
H	-3.027571234	2.981186380	0.125795423
H	-3.256685816	3.084685394	1.883209703
H	-2.719750588	1.563443393	1.130216015
C	-1.118830600	4.632838756	1.418469340
H	-0.098113974	5.002440895	1.576823108
H	-1.723013081	4.957691121	2.278852809
H	-1.532055554	5.117249296	0.527201857
C	-0.738594971	2.509798087	2.681575907
H	-0.652977115	1.414930311	2.629337309
H	-1.501176443	2.753127715	3.435345602
H	0.215049430	2.918373105	3.034922498
C	-0.149398097	3.326769961	-1.656114208
C	-1.617193308	3.575538212	-2.041014152
H	-2.200733738	2.649318222	-2.044678535
H	-1.634605420	3.992793729	-3.058891573
H	-2.100738613	4.306018142	-1.382164364
C	0.590131918	4.674170354	-1.638110548
H	0.235702105	5.343614146	-0.846171427
H	0.413024255	5.177241619	-2.600344865
H	1.672080064	4.542941533	-1.535612541
C	0.485643528	2.425497900	-2.734698291
H	1.544516824	2.225122207	-2.535871822
H	0.413530804	2.939546070	-3.705203981
H	-0.044171255	1.468913001	-2.809591304
C	-1.388453606	-2.707198681	1.851765578
C	-0.804883252	-2.010388129	3.097852208
H	0.079854987	-2.525710543	3.488627614
H	-0.538601500	-0.964374617	2.889103528
C	-2.773857232	-2.094728166	1.590523005
H	-2.710236830	-1.005904732	1.486546573
H	-3.248262664	-2.483839720	0.685475373
C	-1.532797922	-4.204574231	2.160637641
H	-2.059899637	-4.743544816	1.366550613
H	-0.562784791	-4.688769227	2.329873417
C	-0.517834821	-3.525188760	-1.076605791
C	-2.016313716	-3.667510676	-1.391331496
H	-2.556495546	-4.211260119	-0.607874436
H	-2.114942775	-4.249512576	-2.319768512
H	-2.492085529	-2.694414308	-1.548652746
C	0.174980946	-2.893285208	-2.301073744
H	-0.250683857	-1.910508983	-2.533204894
H	0.018028248	-3.551912505	-3.168849278
H	1.254790256	-2.781461197	-2.151747366
C	0.076188605	-4.923919654	-0.843783036
H	1.168475230	-4.895204877	-0.776882601
H	-0.180966736	-5.554972539	-1.707511911
H	-0.323281159	-5.412413562	0.051484668
H	-3.425390570	-2.323995656	2.447027888
H	-1.566282688	-2.013302622	3.890949042
H	-2.122886718	-4.319118371	3.082366114

5 r²scan-3c/def2-mSVP/D(4)

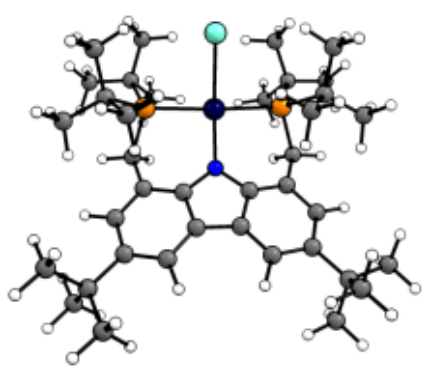

Ru	0.000000000	0.000000000	0.000000000
I	-2.289541239	-0.047406776	-1.410487217
P	0.000000000	2.381070314	0.000000000
P	-0.170313092	-2.333585612	0.460756546
N	2.028437117	-0.073661083	0.000473207
C	1.666052419	2.888314757	0.661891823
H	1.763431445	3.973945933	0.784923410
H	1.743768003	2.421694472	1.654135390
C	2.757674665	2.348525338	-0.207665931
C	3.742051702	3.180501674	-0.746016988
H	3.680928821	4.243081016	-0.514391434
C	4.809738138	2.717411910	-1.543747486
C	4.916575164	1.338817149	-1.748570984
H	5.745630160	0.918824973	-2.312048111
C	3.967862308	0.480411330	-1.187556132
C	2.855496732	0.972358597	-0.465263021
C	2.768077044	-1.242599113	-0.287885484
C	3.910122076	-0.957728992	-1.077663660
C	4.774352087	-1.966889767	-1.495861525
H	5.632197997	-1.712939432	-2.116658974
C	4.556889835	-3.289701964	-1.084481289
C	3.475635230	-3.534767596	-0.218531785
H	3.328705458	-4.534832452	0.181015085
C	2.573862056	-2.543535196	0.191486024
C	1.476999710	-2.838089041	1.165566890
H	1.620857552	-2.206382930	2.053498773
H	1.501320965	-3.887036180	1.485409961
C	5.811374058	3.726147851	-2.113876614
C	5.059961677	4.759286217	-2.977607069
H	4.534735607	4.263472746	-3.804037784
H	5.766925718	5.486240753	-3.400558023
H	4.317765208	5.313580780	-2.390310983
C	6.529033780	4.448516532	-0.955839885
H	5.821725607	4.994872631	-0.320242272
H	7.256562043	5.170990885	-1.350941111
H	7.063313073	3.726886639	-0.324968550
C	6.874810451	3.051448511	-2.992068519
H	7.480380129	2.338129275	-2.418126333
H	7.552701163	3.814102068	-3.396058256
H	6.419855833	2.522260402	-3.839459718
C	5.501610745	-4.399759324	-1.556267039
C	5.093171701	-5.782633986	-1.028171055
H	5.785067230	-6.539859563	-1.418473172
H	4.079827111	-6.055700886	-1.351559254
H	5.135282311	-5.827924373	0.067927762
C	6.933742868	-4.102542952	-1.068483101
H	6.966109539	-4.057643644	0.027595198
H	7.302017372	-3.145416625	-1.456700057
H	7.620589868	-4.891576064	-1.404626749
C	5.484612379	-4.452508332	-3.097683709
H	6.156072771	-5.243866802	-3.458368244
H	5.813084671	-3.502946130	-3.536747756
H	4.471671124	-4.661517055	-3.464997190
C	-1.158758068	3.111295747	1.345755999
C	-2.618602940	2.691022215	1.112803914
H	-3.013315355	3.024107755	0.149103685
H	-3.238282451	3.133705598	1.907030371
H	-2.730570296	1.602457506	1.156613798
C	-1.079138741	4.640977728	1.460128773
H	-0.052376354	4.994411172	1.614662562
H	-1.670145041	4.958722457	2.332206416
H	-1.495469126	5.145835541	0.582074297
C	-0.719763211	2.498586644	2.690679919

H	-0.636729585	1.404828639	2.622320944
H	-1.479522001	2.732071782	3.450320520
H	0.236055504	2.899746527	3.046512187
C	-0.137146214	3.386396635	-1.633855646
C	-1.598181994	3.678840243	-2.013851603
H	-2.205762936	2.769274340	-2.050015653
H	-1.602930145	4.127840064	-3.018237829
H	-2.067764962	4.398872852	-1.333905202
C	0.623751019	4.721811551	-1.579559231
H	0.281455372	5.376116725	-0.770264517
H	0.450900534	5.252051008	-2.527811817
H	1.703457791	4.572199172	-1.485646091
C	0.488935464	2.509828621	-2.737227124
H	1.543371215	2.287010585	-2.538362547
H	0.430983752	3.056002131	-3.690908042
H	-0.052899007	1.563930168	-2.848559386
C	-1.325731058	-2.682271889	1.951272477
C	-0.767964548	-1.886664570	3.148636834
H	0.144527564	-2.331657361	3.562070495
H	-0.554496760	-0.843795375	2.874845818
C	-2.742510990	-2.156334271	1.673427898
H	-2.738006398	-1.072011519	1.516325036
H	-3.204856556	-2.614447265	0.794390600
C	-1.395480198	-4.164035559	2.349060549
H	-1.902505451	-4.773903283	1.594347528
H	-0.403048634	-4.591800140	2.538049898
C	-0.450876270	-3.624801674	-0.937006509
C	-1.944206392	-3.848742883	-1.227705553
H	-2.451345122	-4.375420417	-0.410994133
H	-2.024512606	-4.481526501	-2.124114416
H	-2.469625929	-2.910655053	-1.430718700
C	0.214378431	-3.042453004	-2.200272429
H	-0.235530907	-2.083496730	-2.480019964
H	0.066887815	-3.749374198	-3.030772576
H	1.292308837	-2.896961680	-2.066011839
C	0.191200542	-4.989131503	-0.632246833
H	1.282667427	-4.927186089	-0.584669270
H	-0.059777668	-5.673993434	-1.455686870
H	-0.181823206	-5.438984632	0.293977043
H	-3.374938425	-2.375376863	2.546788950
H	-1.522570400	-1.877951967	3.948168999
H	-1.972999640	-4.249707797	3.281763012

Cartesian coordinates (reported in Å) of bent complexes of **2**

2 with N-Ru-Cl angle: 136° r[2]scan-3c/def2-mSVP/D(4)

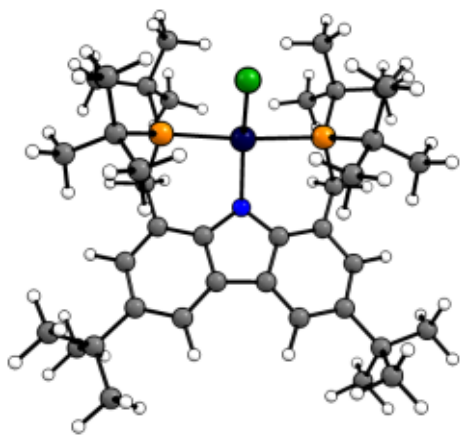

Ru	0.001077453	0.002572025	0.010946800
Cl	-2.090219482	0.068774709	-1.139982189
P	0.085769180	2.366002696	0.090318769
P	-0.182278352	-2.346858698	0.237603908
N	1.966790407	-0.124067281	-0.522350003
C	1.864336990	2.826080537	0.365579184
H	2.013204383	3.905422924	0.499370218
H	2.156984648	2.322074063	1.297745221
C	2.710754623	2.296709753	-0.754419659
C	3.567394535	3.135324919	-1.469260296
H	3.575202187	4.190345517	-1.197168825
C	4.428390328	2.689025262	-2.495972600
C	4.468897645	1.317684876	-2.757169115
H	5.150441782	0.907407860	-3.497807360
C	3.643918480	0.448040596	-2.036794956
C	2.712081523	0.928119925	-1.082801726
C	2.592815603	-1.292324606	-0.990423419
C	3.569804773	-0.990398007	-1.976844510
C	4.296598784	-1.997587994	-2.610184297
H	5.026946437	-1.730053953	-3.372709093
C	4.112055112	-3.335887105	-2.239750333
C	3.206647443	-3.605269969	-1.195956419
H	3.094248898	-4.625717486	-0.838243305
C	2.442277824	-2.619573674	-0.558333755
C	1.533521547	-2.972309095	0.583416332
H	1.869033957	-2.438848546	1.484381043
H	1.565958877	-4.049204902	0.792441382
C	5.283502711	3.710089322	-3.251611005
C	4.359668243	4.751856648	-3.916377272
H	3.666261722	4.261582689	-4.611658722
H	4.954542872	5.485945832	-4.477317533
H	3.763932792	5.297205172	-3.173871929
C	6.236930603	4.420853669	-2.270233316
H	5.686234121	4.954158926	-1.485891205
H	6.856170909	5.153645947	-2.805999969
H	6.900554270	3.694339863	-1.784197630
C	6.129367388	3.054434942	-4.352511218
H	6.843342369	2.330711070	-3.938434584
H	6.704748119	3.826169253	-4.879859961
H	5.498837648	2.541562769	-5.090357019
C	4.908957518	-4.440113806	-2.943339576
C	4.545343940	-5.842367759	-2.432994727
H	5.120876787	-6.593374380	-2.989203312
H	3.479030380	-6.060454023	-2.579542337
H	4.784522292	-5.961935748	-1.368257489
C	6.416310365	-4.217757481	-2.707096928
H	6.646542531	-4.245901539	-1.634380155
H	6.744481751	-3.246850452	-3.097163218
H	7.000675402	-5.001244776	-3.209444271
C	4.615280697	-4.392179987	-4.456551803
H	5.178741896	-5.178193594	-4.978210142
H	4.899973639	-3.426551068	-4.890844687
H	3.545362190	-4.546166044	-4.646727676
C	-0.772280746	2.992127321	1.675730971
C	-2.204524781	2.431702392	1.726125290
H	-2.820520607	2.760035735	0.884404524
H	-2.685088267	2.767696411	2.656941700
H	-2.198571674	1.334654691	1.719797448
C	-0.795896624	4.518311960	1.830445267
H	0.210854074	4.952801355	1.781155650
H	-1.214938731	4.768442184	2.816853184
H	-1.423080218	5.003202738	1.075697923
C	0.001689259	2.398953119	2.869653336
H	0.169328887	1.318041026	2.753383625
H	-0.589897710	2.548191710	3.784270458
H	0.972605433	2.884958274	3.020244671
C	-0.423391818	3.408080484	-1.434958364
C	-1.946444952	3.617182100	-1.489249134
H	-2.484310556	2.665653712	-1.439116862
H	-2.189526484	4.095007099	-2.449873174
H	-2.306805621	4.282375599	-0.695901575
C	0.276215970	4.776766387	-1.471282549

H	0.077906907	5.383361072	-0.580751553
H	-0.102065705	5.332645164	-2.341884062
H	1.359081920	4.672720237	-1.598272254
C	-0.001991759	2.609845909	-2.686484384
H	1.080509687	2.449224839	-2.729840022
H	-0.293746863	3.188344345	-3.576049255
H	-0.507565736	1.639627007	-2.726487692
C	-1.131976348	-2.758649462	1.843735732
C	-0.366028547	-2.070939784	2.991159942
H	0.603810394	-2.539896248	3.193150211
H	-0.199481301	-1.003359063	2.782125570
C	-2.537235847	-2.134522842	1.794247173
H	-2.487023311	-1.056705808	1.600307554
H	-3.171406655	-2.577201250	1.022192787
C	-1.230711738	-4.258638603	2.150729728
H	-1.861171166	-4.786673870	1.427323775
H	-0.244474255	-4.739624426	2.171355594
C	-0.774205118	-3.437499926	-1.221994722
C	-2.308634596	-3.513741503	-1.285537613
H	-2.727717755	-4.106703783	-0.464081953
H	-2.583460963	-4.018225788	-2.223662062
H	-2.766138406	-2.519541105	-1.288823513
C	-0.268573768	-2.769334013	-2.517089352
H	-0.674841936	-1.758460954	-2.626710159
H	-0.610198964	-3.373668102	-3.371074081
H	0.824758776	-2.718878800	-2.556144337
C	-0.208224963	-4.865740751	-1.153398727
H	0.879380798	-4.878082413	-1.279035110
H	-0.639602021	-5.445786803	-1.982605409
H	-0.464449805	-5.381926155	-0.221437369
H	-3.026963296	-2.288794671	2.767242976
H	-0.966047130	-2.139938819	3.909906619
H	-1.685003422	-4.392907444	3.144032474

2 with N-Ru-Cl angle: 180° r^{2}scan-3c/def2-mSVP/D(4)

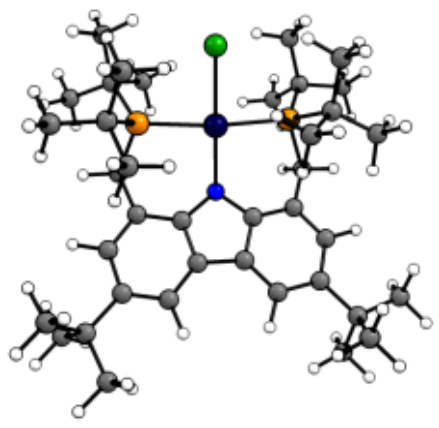

Ru	-0.005633000	-0.010908000	0.004811000
Cl	-2.343232000	0.081239000	-0.469699000
P	-0.001473000	2.369598000	-0.066085000
P	-0.295461000	-2.349506000	0.342061000
N	2.026213000	-0.091002000	0.417256000
C	1.507081000	2.958559000	0.845734000
H	1.586736000	4.051710000	0.843343000
H	1.339088000	2.642250000	1.887813000
C	2.767911000	2.353415000	0.327361000
C	3.832354000	3.189425000	-0.020842000
H	3.684491000	4.260511000	0.117219000
C	5.058143000	2.731947000	-0.545210000
C	5.214426000	1.353238000	-0.691418000
H	6.139654000	0.929193000	-1.073271000
C	4.171438000	0.494119000	-0.332942000
C	2.927717000	0.967601000	0.155376000
C	2.794886000	-1.253847000	0.176779000
C	4.082992000	-0.939286000	-0.332206000
C	5.002584000	-1.925392000	-0.686787000
H	5.971917000	-1.630828000	-1.086907000
C	4.683704000	-3.276377000	-0.512866000
C	3.435658000	-3.573963000	0.062772000
H	3.180438000	-4.608986000	0.277195000
C	2.490690000	-2.602491000	0.421050000
C	1.245774000	-3.010458000	1.138862000
H	1.263337000	-2.559101000	2.140478000
H	1.217086000	-4.099600000	1.261494000
C	6.137405000	3.746092000	-0.932849000
C	5.583891000	4.682326000	-2.027143000
H	5.286287000	4.105991000	-2.912727000
H	6.348837000	5.411831000	-2.327161000
H	4.706421000	5.238109000	-1.674446000
C	6.536530000	4.580291000	0.300985000
H	5.681402000	5.130411000	0.711640000
H	7.310137000	5.311640000	0.029112000
H	6.933766000	3.932216000	1.092504000
C	7.400721000	3.065669000	-1.478736000
H	7.854549000	2.401087000	-0.732111000
H	8.142721000	3.830466000	-1.741762000
H	7.186367000	2.481256000	-2.382974000
C	5.686159000	-4.361025000	-0.920507000
C	5.159555000	-5.778079000	-0.650499000
H	5.906113000	-6.513089000	-0.977626000
H	4.230292000	-5.976060000	-1.201169000
H	4.974616000	-5.943489000	0.418925000
C	6.996988000	-4.177896000	-0.129339000
H	6.810546000	-4.255636000	0.949358000
H	7.451418000	-3.199112000	-0.323694000
H	7.723669000	-4.951552000	-0.413687000
C	5.978395000	-4.237520000	-2.429688000
H	6.702285000	-5.003215000	-2.741403000
H	6.395476000	-3.254291000	-2.678370000
H	5.058212000	-4.371254000	-3.012912000
C	-1.342983000	3.214385000	1.033864000
C	-2.672924000	3.454779000	0.302966000
H	-2.588586000	4.263759000	-0.432830000
H	-3.420063000	3.768758000	1.047343000
H	-3.040718000	2.551533000	-0.189669000
C	-0.872582000	4.566109000	1.606422000
H	-0.052122000	4.458992000	2.324008000
H	-1.717983000	5.016154000	2.146960000
H	-0.566753000	5.274167000	0.828139000
C	-1.587969000	2.255156000	2.216450000
H	-2.075546000	1.332917000	1.884188000
H	-2.247360000	2.750977000	2.944650000
H	-0.657367000	1.994389000	2.740453000
C	0.085736000	3.152844000	-1.800815000
C	-1.150842000	2.732635000	-2.614192000
H	-1.252824000	1.642137000	-2.648187000
H	-1.026542000	3.100781000	-3.643333000
H	-2.083099000	3.144692000	-2.220210000
C	0.213326000	4.681000000	-1.782498000
H	-0.685086000	5.167169000	-1.384910000
H	0.352327000	5.038492000	-2.813646000
H	1.083610000	5.013887000	-1.201716000
C	1.318739000	2.552670000	-2.507123000
H	2.262716000	2.901521000	-2.078214000
H	1.288196000	2.852517000	-3.564959000
H	1.310443000	1.454286000	-2.467464000
C	-1.596351000	-2.958487000	1.628331000
C	-1.214631000	-2.310813000	2.976405000
H	-0.309367000	-2.740390000	3.419368000
H	-1.076437000	-1.224796000	2.875246000
C	-3.043059000	-2.528219000	1.329369000

H	-3.166741000	-1.447884000	1.421762000
H	-3.388011000	-2.820173000	0.334757000
C	-1.575520000	-4.489369000	1.778726000
H	-1.964533000	-4.989481000	0.884184000
H	-0.579199000	-4.888822000	1.999365000
C	-0.430456000	-3.298097000	-1.311271000
C	-1.890802000	-3.399824000	-1.777023000
H	-2.464198000	-4.110201000	-1.169861000
H	-1.899697000	-3.770615000	-2.812757000
H	-2.392951000	-2.425478000	-1.757782000
C	0.345252000	-2.439696000	-2.331728000
H	-0.126738000	-1.453312000	-2.456241000
H	0.328204000	-2.944024000	-3.310033000
H	1.393163000	-2.295746000	-2.040769000
C	0.195344000	-4.699859000	-1.267603000
H	1.274641000	-4.652579000	-1.086621000
H	0.044724000	-5.182727000	-2.244704000
H	-0.264299000	-5.341568000	-0.507729000
H	-3.692257000	-3.017817000	2.071177000
H	-2.036505000	-2.476876000	3.687270000
H	-2.231052000	-4.764936000	2.618005000

2 with N-Ru-Cl angle: 202° r[2]scan-3c/def2-mSVP/D(4)

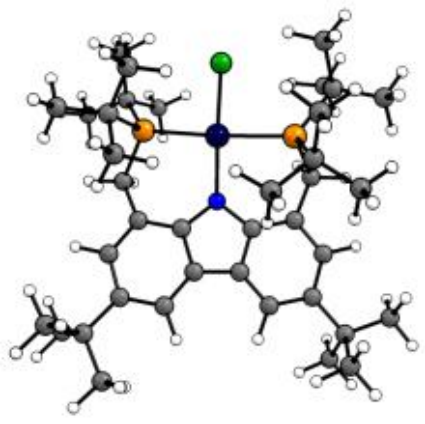

Ru	-0.158140000	0.125339000	0.299695000
Cl	-2.505172000	0.285180000	0.656819000
P	-0.136640000	2.499366000	0.356166000
P	-0.302325000	-2.266383000	0.038374000
N	1.858572000	0.000777000	0.779756000
C	1.502090000	3.153422000	0.905121000
H	1.607116000	4.217671000	0.658565000
H	1.469084000	3.085620000	2.004135000
C	2.709478000	2.400478000	0.442921000
C	3.839200000	3.136417000	0.072419000
H	3.722850000	4.218479000	0.011266000
C	5.107699000	2.575228000	-0.178861000
C	5.239416000	1.200447000	0.010361000
H	6.198978000	0.703495000	-0.107811000
C	4.121162000	0.439716000	0.362568000
C	2.824926000	0.998211000	0.511240000
C	2.613023000	-1.183729000	0.918311000
C	3.993073000	-0.956346000	0.664128000
C	4.943459000	-1.973976000	0.723952000
H	5.986434000	-1.739864000	0.514781000
C	4.554834000	-3.275859000	1.055015000
C	3.188309000	-3.492468000	1.300649000
H	2.844413000	-4.493261000	1.550786000
C	2.206942000	-2.492909000	1.225035000
C	0.777400000	-2.877304000	1.427822000
H	0.370883000	-2.384766000	2.324321000
H	0.698740000	-3.962457000	1.574100000
C	6.266190000	3.480824000	-0.604488000
C	5.900700000	4.183729000	-1.927728000
H	5.711417000	3.444812000	-2.716926000
H	6.722478000	4.837277000	-2.251743000
H	4.999954000	4.800092000	-1.818587000
C	6.526180000	4.542007000	0.483976000
H	5.645154000	5.173037000	0.651339000
H	7.358067000	5.195394000	0.186555000
H	6.784954000	4.061678000	1.436202000
C	7.564699000	2.691371000	-0.822478000
H	7.894237000	2.195704000	0.099842000
H	8.361901000	3.377350000	-1.136352000
H	7.447558000	1.930916000	-1.605524000
C	5.596910000	-4.396731000	1.124038000
C	4.974284000	-5.756727000	1.472356000
H	5.761340000	-6.521080000	1.500704000
H	4.235103000	-6.066320000	0.721597000
H	4.488535000	-5.739914000	2.456640000
C	6.643733000	-4.055586000	2.203096000
H	6.165594000	-3.957351000	3.185965000
H	7.156580000	-3.111851000	1.981551000
H	7.401914000	-4.848790000	2.262352000
C	6.294994000	-4.527931000	-0.244819000
H	7.046466000	-5.328908000	-0.213000000
H	6.802315000	-3.598415000	-0.529158000
H	5.564248000	-4.767695000	-1.028120000
C	-1.265640000	3.496933000	1.559389000
C	-2.702807000	3.670133000	1.040478000
H	-2.746495000	4.344355000	0.177006000
H	-3.301600000	4.129393000	1.841213000
H	-3.161823000	2.715649000	0.776005000
C	-0.708253000	4.902638000	1.863353000
H	0.240212000	4.875240000	2.409944000
H	-1.435801000	5.418534000	2.506592000
H	-0.575962000	5.512893000	0.963946000
C	-1.292818000	2.700294000	2.878144000
H	-1.790666000	1.735131000	2.749631000
H	-1.844561000	3.282939000	3.630732000
H	-0.283344000	2.522780000	3.274095000
C	-0.325010000	3.127487000	-1.434300000
C	-1.590276000	2.506597000	-2.050786000
H	-1.537572000	1.410187000	-2.044100000
H	-1.675099000	2.839855000	-3.095839000
H	-2.504879000	2.793809000	-1.523178000
C	-0.367156000	4.654037000	-1.580884000
H	-1.270358000	5.092922000	-1.143896000
H	-0.366260000	4.904027000	-2.652512000
H	0.511896000	5.137326000	-1.135361000
C	0.895709000	2.601740000	-2.213685000
H	1.828308000	3.087133000	-1.906236000
H	0.741274000	2.798449000	-3.284970000
H	1.028588000	1.518288000	-2.085249000
C	-1.904979000	-3.274627000	0.397627000
C	-2.270528000	-2.999424000	1.872266000
H	-1.561930000	-3.468617000	2.564656000
H	-2.333947000	-1.927592000	2.084899000
C	-3.093925000	-2.856773000	-0.487400000
H	-3.288218000	-1.784480000	-0.427880000
H	-2.955691000	-3.136980000	-1.534619000
C	-1.703190000	-4.791554000	0.237870000
H	-1.598081000	-5.090203000	-0.810688000
H	-0.842880000	-5.174198000	0.799867000
C	0.479813000	-2.876907000	-1.609061000
C	-0.609558000	-2.930488000	-2.693516000
H	-1.296903000	-3.770320000	-2.553728000
H	-0.121658000	-3.064784000	-3.669654000
H	-1.193207000	-2.000513000	-2.737340000
C	1.514435000	-1.830231000	-2.066669000
H	1.069598000	-0.827304000	-2.136040000

H	1.870573000	-2.109173000	-3.069418000
H	2.382013000	-1.774159000	-1.404244000
C	1.179879000	-4.240762000	-1.510586000
H	2.019178000	-4.208665000	-0.808057000
H	1.584674000	-4.498170000	-2.501260000
H	0.500472000	-5.045114000	-1.213895000
H	-3.987179000	-3.387166000	-0.124770000
H	-3.258259000	-3.440836000	2.067930000
H	-2.599281000	-5.295267000	0.629859000

7. X-Ray Crystal Structure Determinations

Crystal data and details of the structure determinations are compiled in Table S 51+52. Full shells of intensity data were collected at 120(1) K with an Agilent Technologies Supernova-E CCD diffractometer (Cu-K_α radiation, microfocus X-ray tube, multilayer mirror optics). Detector frames (typically ω- and φ-scans, scan width 1.0°) were integrated by profile fitting.[49] Data were corrected for air and detector absorption, Lorentz and polarization effects[50] and scaled essentially by application of appropriate spherical harmonic functions.[50-52] Absorption by the crystal was treated numerically (Gaussian grid). [52-53] An illumination correction was performed as part of the numerical absorption correction.[50,52]

Using OLEX2,[54] the structures were solved with SHELXT[55] (intrinsic phasing) and refined with SHELXL[56] by full-matrix least squares methods based on F^2 against all unique reflections. All non-hydrogen atoms were given anisotropic displacement parameters. Hydrogen atoms were generally input at calculated positions and refined with a riding model.[57-60] Split atom models were used to refine disordered groups and/or solvent molecules. When found necessary, suitable geometry and adp restraints were applied.[57-61]

CCDC 2557324-2557327 contains the supplementary crystallographic data for this paper. These data can be obtained free of charge from the Cambridge Crystallographic Data Centre's and FIZ Karlsruhe’s joint Access Service via https://www.ccdc.cam.ac.uk

Table S 57 Crystal data and details of the structure determinations for **2**, **3** and **4**.

Compound	**2**	**3**	**4**
Empirical formula	$C_{38}H_{62}NP_2ClRu$	$C_{38}H_{62}NFP_2Ru$	$C_{38}H_{62}NP_2BrRu$
Formula weight	731.34	714.89	775.80
Temperature [K]	120(1)	120(1)	120(1)
Crystal system	monoclinic	monoclinic	orthorhombic
Space group (number)	*C*2/*c* (15)	*I*2/*a* (15)	$P2_12_12_1$ (19)
a [Å]	29.3110(4)	25.4104(2)	11.60770(10)
b [Å]	13.06860(10)	13.04370(10)	14.7265(2)
c [Å]	25.3645(3)	23.4617(3)	22.9069(3)
α [°]	90	90	90
β [°]	128.880(2)	106.3130(10)	90
γ [°]	90	90	90
Volume [$Å^3$]	7563.5(2)	7463.22(13)	3915.72(8)
Z	8	8	4
ρ_{calc} [gcm^{-3}]	1.285	1.272	1.316
μ [mm^{-1}]	4.985	4.436	5.382
Transmission factors (max, min)	1.000, 0.636	1.000, 0.777	1.000, 0.767
F(000)	3104.0	3040.0	1624.0
Radiation	Cu Kα (λ = 1.54184)	Cu Kα (λ = 1.54184)	Cu Kα (λ = 1.54184)
2θ range [°]	6.908 to 143.138	7.25 to 142.192	7.136 to 142.296
Index ranges *h*,*k*,*l*	±35, ±15, ±31	±31, ±15, −27-28	±14, ±18, −28-26
Reflections collected	61906	70551	62076
Independent reflections	7244 (R_{int} = 0.0707)	7159 (R_{int} = 0.0517)	7542 (R_{int} = 0.0709)
Observed [$I \geq 2\sigma(I)$]	6870	6797	7270
Data Completeness	0.994	0.992	1.78/1.00
Data / Restraints / Parameters	7244/21/437	7159/63/437	7542/0/406
Goodness-of-fit on F^2	1.017	1.069	1.019
Final *R* indexes [$I \geq 2\sigma(I)$] R_1, wR_2	0.0361, 0.0962	0.0633, 0.1825	0.0381, 0.1003
Final *R* indexes [all data] R_1, wR_2	0.0376, 0.0976	0.0653, 0.1848	0.0398, 0.1018
Largest peak/hole [$eÅ^{-3}$]	1.04/-0.81	1.79/-1.17	1.04/-0.91
CCDC number	2557324	2557326	2557327

Table S 58: Crystal data and details of the structure determinations for **5**.

Compound	**5**
Empirical formula	$C_{38}H_{62}NP_2RuI$
Formula weight	822.79
Temperature [K]	120(1)
Crystal system	monoclinic
Space group (number)	$P2_1/n$ (14)
a [Å]	16.84200(10)
b [Å]	12.39940(10)
c [Å]	18.8344(2)
α [°]	90
β [°]	94.6120(10)
γ [°]	90
Volume [$Å^3$]	3920.47(6)
Z	4
ρ_{calc} [gcm^{-3}]	1.394
μ [mm^{-1}]	10.365
Transmission factors (max, min)	0.904, 0.498
F(000)	1696.0
Radiation	Cu Kα (λ = 1.54184)
2θ range [°]	6.776 to 142.086
Index ranges *h*,*k*,*l*	±20, ±15, −23-22
Reflections collected	77126
Independent reflections	7534 (R_{int} = 0.0831)
Observed [$I \geq 2\sigma(I)$]	6914
Data Completeness	0.993
Data / Restraints / Parameters	7534/0/406
Goodness-of-fit on F^2	1.044
Final *R* indexes [$I \geq 2\sigma(I)$] R_1, wR_2	0.0642, 0.1670
Final *R* indexes [all data] R_1, wR_2	0.0670, 0.1718
Largest peak/hole [$eÅ^{-3}$]	2.34/-1.91
CCDC number	2557326

References

1 J. Higuchi, S. Kuriyama, A. Eizawa, K. Arashiba, K. Nakajima, Y. Nishibayashi, *Preparation and reactivity of iron complexes bearing anionic carbazole-based PNP-type pincer ligands toward catalytic nitrogen fixation*, *Dalton Trans.* **2018**, *47*, 1117-1121.

2 J. Guschlbauer, T. Vollgraff, X. Xie, A. Fetoh, J. Sundermeyer, *Heavy silylchalcogenido lanthanates synthesis $Ph_4P[Cp_3La–ESiMe_3]$ (E = S, Se, and Te) via fluoride-induced demethylation of dimethylcarbonate to $Ph_4P[OCO_2Me]$ key intermediate*, *Dalton Trans.* **2021**, *50*, 13103-13111.

3 G. A. Bain, J. F. Berry, *Diamagnetic Corrections and Pascal's Constants*, *J. Chem. Educ.* **2008**, *85*, 532.

4 S. Stoll, A. Schweiger, *EasySpin, a comprehensive software package for spectral simulation and analysis in EPR*, *J. Magn. Reson.* **2006**, *178*, 42-55.

5 B. Martin, J. Autschbach, *Temperature dependence of contact and dipolar NMR chemical shifts in paramagnetic molecules*, *J. Chem. Phys.* **2015**, *142*.

6 F. Neese, *The ORCA program system*, *WIREs Comput. Mol. Sci.* **2012**, *2*, 73-78.

7 F. Neese, *Software Update: The ORCA Program System—Version 6.0*, *WIREs Comput. Mol. Sci.* **2025**, *15*.

8 J. W. Furness, A. D. Kaplan, J. Ning, J. P. Perdew, J. Sun, *Accurate and Numerically Efficient r^2SCAN Meta-Generalized Gradient Approximation*, *J. Phys. Chem. Lett.* **2020**, *11*, 8208-8215.

9 S. Ehlert, U. Huniar, J. Ning, J. W. Furness, J. Sun, A. D. Kaplan, J. P. Perdew, J. G. Brandenburg, *r2SCAN-D4: Dispersion corrected meta-generalized gradient approximation for general chemical applications*, *J. Chem. Phys.* **2021**, *154*, 061101.

10 S. Grimme, A. Hansen, S. Ehlert, J.-M. Mewes, *r2SCAN-3c: A “Swiss army knife” composite electronic-structure method*, *J. Chem. Phys.* **2021**, *154*, 064103.

11 E. Caldeweyher, J.-M. Mewes, S. Ehlert, S. Grimme, *Extension and evaluation of the D4 London-dispersion model for periodic systems*, *Phys. Chem. Chem. Phys.* **2020**, *22*, 8499-8512.

12 F. Weigend, R. Ahlrichs, *Balanced basis sets of split valence, triple zeta valence and quadruple zeta valence quality for H to Rn: Design and assessment of accuracy*, *Phys. Chem. Chem. Phys.* **2005**, *7*, 3297.

13 S. Grimme, J. G. Brandenburg, C. Bannwarth, A. Hansen, *Consistent structures and interactions by density functional theory with small atomic orbital basis sets*, *J. Chem. Phys.* **2015**, *143*.

14 F. Neese, *An improvement of the resolution of the identity approximation for the formation of the Coulomb matrix*, *J. Comput. Chem.* **2003**, *24*, 1740-1747.

15 B. Helmich-Paris, B. de Souza, F. Neese, R. Izsák, *An improved chain of spheres for exchange algorithm*, *J. Chem. Phys.* **2021**, *155*.

16 F. Weigend, *Accurate Coulomb-fitting basis sets for H to Rn*, *Phys. Chem. Chem. Phys.* **2006**, *8*, 1057.

17 A. V. Marenich, C. J. Cramer, D. G. Truhlar, *Universal Solvation Model Based on Solute Electron Density and on a Continuum Model of the Solvent Defined by the Bulk Dielectric Constant and Atomic Surface Tensions*, *J. Phys. Chem. B* **2009**, *113*, 6378-6396.

18 L. Goerigk, S. Grimme, *Efficient and Accurate Double-Hybrid-Meta-GGA Density Functionals—Evaluation with the Extended GMTKN30 Database for General Main Group Thermochemistry, Kinetics, and Noncovalent Interactions*, *J. Chem. Theory Comput.* **2011**, *7*, 291-309.

19 S. Grimme, *Accurate description of van der Waals complexes by density functional theory including empirical corrections*, *J. Comput. Chem.* **2004**, *25*, 1463-1473.

20 S. Grimme, J. Antony, S. Ehrlich, H. Krieg, *A consistent and accurate ab initio parametrization of density functional dispersion correction (DFT-D) for the 94 elements H-Pu*, *J. Chem. Phys.* **2010**, *132*.

21 A. D. Becke, E. R. Johnson, *Exchange-hole dipole moment and the dispersion interaction*, *J. Chem. Phys.* **2005**, *122*.

22 E. R. Johnson, A. D. Becke, *A post-Hartree–Fock model of intermolecular interactions*, *J. Chem. Phys.* **2005**, *123*.

23 S. Kossmann, F. Neese, *Efficient Structure Optimization with Second-Order Many-Body Perturbation Theory: The RIJCOSX-MP2 Method*, *J. Chem. Theory Comput.* **2010**, *6*, 2325-2338.

24 A. Hellweg, C. Hättig, S. Höfener, W. Klopper, *Optimized accurate auxiliary basis sets for RI-MP2 and RI-CC2 calculations for the atoms Rb to Rn*, *Theor. Chem. Acc.* **2007**, *117*, 587-597.

25 E. v. Lenthe, E. J. Baerends, J. G. Snijders, *Relativistic regular two‐component Hamiltonians*, *J. Chem. Phys.* **1993**, *99*, 4597-4610.

26 E. van Lenthe, E. J. Baerends, J. G. Snijders, *Relativistic total energy using regular approximations*, *J. Chem. Phys.* **1994**, *101*, 9783-9792.

27 C. van Wüllen, *Molecular density functional calculations in the regular relativistic approximation: Method, application to coinage metal diatomics, hydrides, fluorides and chlorides, and comparison with first-order relativistic calculations*, *J. Chem. Phys.* **1998**, *109*, 392-399.

28 J. D. Rolfes, F. Neese, D. A. Pantazis, *All‐electron scalar relativistic basis sets for the elements Rb–Xe*, *J. Comput. Chem.* **2020**, *41*, 1842-1849.

29 J. Tao, J. P. Perdew, V. N. Staroverov, G. E. Scuseria, *Climbing the Density Functional Ladder: Nonempirical Meta–Generalized Gradient Approximation Designed for Molecules and Solids*, *Phys. Rev. Lett.* **2003**, *91*.

30 V. N. Staroverov, G. E. Scuseria, J. Tao, J. P. Perdew, *Comparative assessment of a new nonempirical density functional: Molecules and hydrogen-bonded complexes*, *J. Chem. Phys.* **2003**, *119*, 12129-12137.

31 R. L. Martin, *Natural transition orbitals*, *J. Chem. Phys.* **2003**, *118*, 4775-4777.

32 J.-D. Chai, M. Head-Gordon, *Systematic optimization of long-range corrected hybrid density functionals*, *J. Chem. Phys.* **2008**, *128*.

33 A. D. Becke, *Density‐functional thermochemistry. III. The role of exact exchange*, *J. Chem. Phys.* **1993**, *98*, 5648-5652.

34 C. Lee, W. Yang, R. G. Parr, *Development of the Colle-Salvetti correlation-energy formula into a functional of the electron density*, *Phys. Rev. B* **1988**, *37*, 785-789.

35 B. O. Roos, P. R. Taylor, P. E. M. Sigbahn, *A complete active space SCF method (CASSCF) using a density matrix formulated super-CI approach*, *Chem. Phys.* **1980**, *48*, 157-173.

36 S. Per, H. Anders, R. Björn, L. Bernard, *A Comparison of the Super-CI and the Newton-Raphson Scheme in the Complete Active Space SCF Method*, *Phys. Scr.* **1980**, *21*, 323.

37 L. Lang, M. Atanasov, F. Neese, *Improvement of Ab Initio Ligand Field Theory by Means of Multistate Perturbation Theory*, *J. Phys. Chem. A* **2020**, *124*, 1025-1037.

38 C. Angeli, R. Cimiraglia, S. Evangelisti, T. Leininger, J.-P. Malrieu, *Introduction of n-electron valence states for multireference perturbation theory*, *J. Chem. Phys.* **2001**, *114*, 10252-10264.

39 C. Angeli, R. Cimiraglia, J.-P. Malrieu, *N-electron valence state perturbation theory: a fast implementation of the strongly contracted variant*, *Chem. Phys. Lett.* **2001**, *350*, 297-305.

40 C. Angeli, R. Cimiraglia, J.-P. Malrieu, *n-electron valence state perturbation theory: A spinless formulation and an efficient implementation of the strongly contracted and of the partially contracted variants*, *J. Chem. Phys.* **2002**, *117*, 9138-9153.

41 G. L. Stoychev, A. A. Auer, F. Neese, *Automatic Generation of Auxiliary Basis Sets*, *J. Chem. Theory Comput.* **2017**, *13*, 554-562.

42 F. Neese, *Efficient and accurate approximations to the molecular spin-orbit coupling operator and their use in molecular g-tensor calculations*, *J. Chem. Phys.* **2005**, *122*.

43 B. A. Heß, C. M. Marian, U. Wahlgren, O. Gropen, *A mean-field spin-orbit method applicable to correlated wavefunctions*, *Chem. Phys. Lett.* **1996**, *251*, 365-371.

44 Z. G. A, *Chemcraft - graphical program for visualization of quantum chemistry computations* (www.chemcraftprog.com), Ivanovo, Russia, **2005**.

45 T. Bruckhoff, *CUVNMR – A paramagnetic NMR simulation program*, *ChemRxiv* **2026**, *2026*.

46 I. Bertini, C. Luchinat, G. Parigi, E. Ravera, *NMR of paramagnetic molecules: applications to metallobiomolecules and models, Vol. 2*Elsevier, **2016**.

47 G. T. P. Charnock, I. Kuprov, *A partial differential equation for pseudocontact shift*, *Phys. Chem. Chem. Phys.* **2014**, *16*, 20184-20189.

48 J. C. Ott, E. A. Suturina, I. Kuprov, J. Nehrkorn, A. Schnegg, M. Enders, L. H. Gade, *Observability of Paramagnetic NMR Signals at over 10 000 ppm Chemical Shifts*, *Angew. Chem. Int. Ed.* **2021**, *60*, 22856-22864.

49 W. Kabsch, *International Tables for Crystallography Vol. F Ch. 11.3* (Eds.: E. Arnold, M. G. Rossmann), Kluwer Academic, Dordrecht, The Netherlands, **2006**.

50 *CrysAlisPro Diffraction*, Agilent Technologies UK Ltd. and Rigaku Oxford Diffraction, Rigaku Polska Sp.z o.o., Oxford, UK and Wrocław, Poland, **2011-2014 and 2015-2020**

51 R. Blessing, *An empirical correction for absorption anisotropy*, *Acta Crystallogr., Sect. A* **1995**, *51*, 33-38.

52 *SCALE3 ABSPACK, CrysAlisPro*, Agilent Technologies UK Ltd. and Rigaku Oxford Diffraction, Rigaku Polska Sp.z o.o., Oxford, UK and Wrocław, Poland, **2011-2014 and 2015-2020**

53 W. R. Busing, H. A. Levy, *High-speed computation of the absorption correction for single-crystal diffraction measurements*, *Acta Crystallogr.* **1957**, *10*, 180-182.

54 O. V. Dolomanov, L. J. Bourhis, R. J. Gildea, J. A. K. Howard, H. Puschmann, *OLEX2: a complete structure solution, refinement and analysis program*, *J. Appl. Crystallogr.* **2009**, *42*, 339-341.

55 G. Sheldrick, *SHELXT - Integrated space-group and crystal-structure determination*, *Acta Crystallogr. Sect. A* **2015**, *71*, 3-8.

56 G. Sheldrick, *Crystal structure refinement with SHELXL*, *Acta Crystallogr. Sect. C* **2015**, *71*, 3-8.

57 J. S. Rollett, *Crystallographic Computing* (Ed.: M. R. T. N. W. Isaaks), IUCr and Oxford University Press, Oxford, UK, **1970**.

58 D. Watkin, *Crystallographic Computing 4* (Eds.: N. W. Isaaks, M. R. Taylor), IUCr and Oxford University Press, Oxford, UK, **1988**.

59 P. Müller, R. Herbst-Irmer, A. L. Spek, T. R. Schneider, M. R. Sawaya, *Crystal Structure Refinement* (Ed.: P. Müller), Ch. 5, Oxford University Press, Oxford, UK, **2006**.

60 D. Watkin, *Structure refinement: some background theory and practical strategies*, *J. Appl. Crystallogr.* **2008**, *41*, 491-522.

61 A. Thorn, B. Dittrich, G. M. Sheldrick, *Enhanced rigid-bond restraints*, *Acta Crystallographica Section A* **2012**, *68*, 448-451.
